# SARS-CoV-2 Variants Associated with Vaccine Breakthrough in the Delaware Valley through Summer 2021

**DOI:** 10.1128/mbio.03788-21

**Published:** 2022-02-08

**Authors:** Andrew D. Marques, Scott Sherrill-Mix, John K. Everett, Shantan Reddy, Pascha Hokama, Aoife M. Roche, Young Hwang, Abigail Glascock, Samantha A. Whiteside, Jevon Graham-Wooten, Layla A. Khatib, Ayannah S. Fitzgerald, Ahmed M. Moustafa, Colleen Bianco, Swetha Rajagopal, Jenna Helton, Regan Deming, Lidiya Denu, Azad Ahmed, Eimear Kitt, Susan E. Coffin, Claire Newbern, Josh Chang Mell, Paul J. Planet, Nitika Badjatia, Bonnie Richards, Zi-Xuan Wang, Carolyn C. Cannuscio, Katherine M. Strelau, Anne Jaskowiak-Barr, Leigh Cressman, Sean Loughrey, Arupa Ganguly, Michael D. Feldman, Ronald G. Collman, Kyle G. Rodino, Brendan J. Kelly, Frederic D. Bushman

**Affiliations:** a Department of Microbiology, Perelman School of Medicine, University of Pennsylvania, Philadelphia, Pennsylvania, USA; b Pulmonary, Allergy and Critical Care Division, Department of Medicine, University of Pennsylvania Perelman School of Medicine, Philadelphia, Pennsylvania, USA; c Division of Infectious Diseases, Children’s Hospital of Philadelphia, Philadelphia, Pennsylvania, USA; d Division of Gastroenterology, Hepatology & Nutrition, Children’s Hospital of Philadelphia, Philadelphia, Pennsylvania, USA; e Division of COVID-19 Containment, Philadelphia Department of Public Health, Philadelphia, Pennsylvania, USA; f Department of Microbiology & Immunology, Center for Genomic Sciences, Drexel University College of Medicine, Philadelphia, Pennsylvania, USA; g Department of Pediatrics, Perelman School of Medicine, University of Pennsylvania, Philadelphia, Pennsylvania, USA; h Sackler Institute for Comparative Genomics, American Museum of Natural History, New York, New York, USA; i Molecular & Genomic Pathology Laboratory, Thomas Jefferson University Hospital, Philadelphia, Pennsylvania, USA; j Jefferson Occupational Health Network for Employees and Students (JOHN), Thomas Jefferson University, Philadelphia, Pennsylvania, USA; k Department of Anatomy, Pathology, and Cell Biology, Thomas Jefferson University Hospital, Philadelphia, Pennsylvania, USA; l Leonard Davis Institute of Health Economics, University of Pennsylvania, Philadelphia, Pennsylvania, USA; m Department of Family Medicine and Community Health, University of Pennsylvania, Philadelphia, Pennsylvania, USA; n Division of Infectious Diseases, Department of Medicine & Department of Biostatistics, Epidemiology, and Informatics, Perelman School of Medicine, University of Pennsylvania, Philadelphia, Pennsylvania, USA; o Department of Genetics, Perelman School of Medicine, University of Pennsylvania, Philadelphia, Pennsylvania, USA; p Department of Pathology and Laboratory Medicine, Perelman School of Medicine, University of Pennsylvania, Philadelphia, Pennsylvania, USA; Columbia University/HHMI

**Keywords:** SARS-CoV-2, COVID-19, coronavirus, genome sequencing, Philadelphia

## Abstract

The severe acute respiratory coronavirus-2 (SARS-CoV-2) is the cause of the global outbreak of COVID-19. Evidence suggests that the virus is evolving to allow efficient spread through the human population, including vaccinated individuals. Here, we report a study of viral variants from surveillance of the Delaware Valley, including the city of Philadelphia, and variants infecting vaccinated subjects. We sequenced and analyzed complete viral genomes from 2621 surveillance samples from March 2020 to September 2021 and compared them to genome sequences from 159 vaccine breakthroughs. In the early spring of 2020, all detected variants were of the B.1 and closely related lineages. A mixture of lineages followed, notably including B.1.243 followed by B.1.1.7 (alpha), with other lineages present at lower levels. Later isolations were dominated by B.1.617.2 (delta) and other delta lineages; delta was the exclusive variant present by the last time sampled. To investigate whether any variants appeared preferentially in vaccine breakthroughs, we devised a model based on Bayesian autoregressive moving average logistic multinomial regression to allow rigorous comparison. This revealed that B.1.617.2 (delta) showed 3-fold enrichment in vaccine breakthrough cases (odds ratio of 3; 95% credible interval 0.89-11). Viral point substitutions could also be associated with vaccine breakthroughs, notably the N501Y substitution found in the alpha, beta and gamma variants (odds ratio 2.04; 95% credible interval of1.25–3.18). This study thus overviews viral evolution and vaccine breakthroughs in the Delaware Valley and introduces a rigorous statistical approach to interrogating enrichment of breakthrough variants against a changing background.

## INTRODUCTION

The global COVID-19 pandemic is caused by infection with the virus SARS-CoV-2 (1). Analysis of whole-genome sequences from global viral samples shows ongoing changes in the composition of viral populations. RNA viruses have high mutation rates, so sequence change is expected in the viral genome due to random genetic drift (2). However, selection for efficient immune evasion and transmission between humans now seem likely to be major drivers of SARS-CoV-2 diversification (3–5).

Widespread vaccination against SARS-CoV-2 was introduced in the United States in the winter of 2020–2021. First to be implemented were vaccines based on modified mRNAs, followed by adenovirus vector delivery. Vaccines are highly protective against infection, severe disease, and death. However, infection of vaccinated individuals has been widely detected, albeit typically with much milder disease course compared to that experienced by unvaccinated individuals (6–8). Thus, interest turns to the question of which viral features are associated with vaccine breakthrough infections (7–9).

Several criteria can be applied to assessing whether sequence changes in a new variant have likely evolved to promote infection and vaccine breakthrough. Substitutions found in viral spike proteins can be tested in laboratory experiments to determine whether they promote more efficient replication in human cells or reduce binding of human antibodies (10–18). Other substitutions may alter epitopes targeted by the cellular immune system (6, 19, 20). Viral lineages with diverse combinations of these substitution have been identified and designated variants being monitored and variants of concern (VBM/VOC) by the Centers for Disease Control and Prevention (CDC). Some of the substitutions in these variants have been detected arising independently on multiple genetic backgrounds, such as the spike substitutions N501Y, E484K or the 69–70 deletion (4, 21–24), supporting a model of convergent evolution.

One indication of selection for increased transmission in humans is that several new variants have spread globally and rapidly displaced preexisting strains. This was first documented for the D614G substitution, which spread around the world in the Spring of 2020 and displaced most strains lacking this substitution (25–28). More recently, variants first identified in the UK (B.1.1.7 or alpha) (29, 30), South Africa (B.1.351 or beta), Brazil (P.1 or gamma), California (B.1.427 and B.1.429 or epsilon), New York (B.1.526 or iota) (31), and India (B.1.617.2 or delta) (8) have been suggested to be spreading at the expense of preexisting viral types. Against this background, intense interest focuses on whether particular variants are more efficient at infecting vaccinated individuals.

We have investigated viral genomic evolution in the Delaware Valley, which encompasses the city of Philadelphia, in a sample that includes vaccine breakthrough cases. Our initial report on the first wave of infection in this area (32) revealed that lineages in Philadelphia most closely matched sequences derived from New York City, approximately 100 miles away, which is larger than Philadelphia and had an earlier peak in infection. We also found that in some cases different viral sequence polymorphisms could be found in the same patients from different body sites or in longitudinal samples, suggestive of ongoing evolution within infected individuals (32).

In this study, samples were collected from 2621 surveillance samples from infected individuals and 159 vaccine breakthrough cases through September 2021, and the representation of different variants compared. We found that several waves of variants rose and fell in prevalence over the course of our sampling period, by the end of sampling all genomes were identified as VOC delta lineages, and the delta lineage B.1.617.2 was potentially 3-fold enriched among vaccine breakthrough samples compared to surveillance samples. The amino acid substitution N501Y, found in the alpha, beta, and gamma variants, was also notably enriched in vaccine breakthrough samples. We introduce a rigorous statistical approach, Bayesian autoregressive moving average logistic multinomial regression, that should be widely useful for assessing enrichment of viral variants while controlling for the changing background of circulating strains.

## RESULTS

### The COVID-19 epidemic in the Delaware Valley.

**FIG 1:**
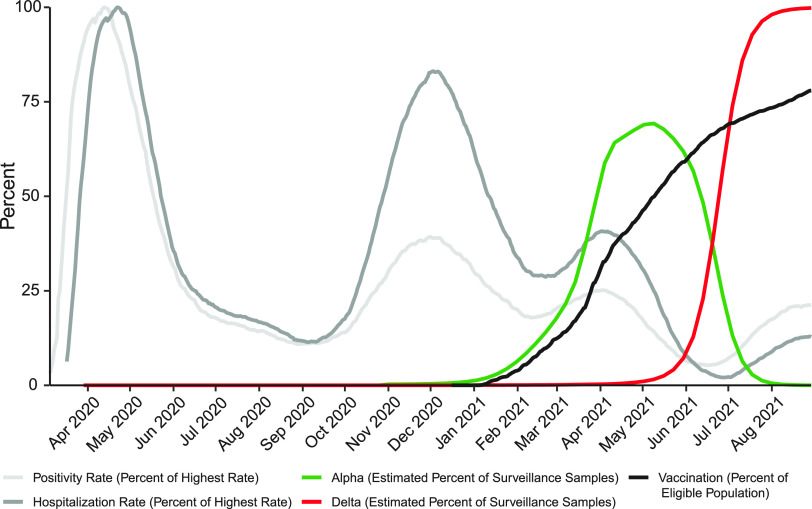
Longitudinal data from the COVID-19 pandemic in the city of Philadelphia. The *y* axis shows the daily test positivity rate (light gray) as a percentage of the highest value (26.57% positivity on 4/13/2020), the hospitalization rate (dark gray) as a percentage of the highest value (87 hospitalizations per day on 4/22/2020), the vaccination rate in adults 18 years old and older (black). The estimated percentage of surveillance samples classified as alpha (green) or delta (red) variant was estimated from the sequence data presented in this paper. Other data are from the city of Philadelphia “Testing Data: Programs and Initiatives.”

Sampling was carried out from March 2020 to September 2021. Over the course of the study, several waves of infection are evident as increased test positivity rates (Fig. 1, light gray curve) and COVID-19-attributed hospitalizations in the city of Philadelphia (Fig. 1, dark gray curve). Widespread vaccination was introduced in winter 2020–2021, reaching over 70% (in adults 18 years old and older) in Philadelphia by September 2021 (Fig. 1, black curve). As is described below, during this period, SARS-CoV-2 variants alpha and delta, designated VBM/VOC, became the overwhelming majority of genomes identified by our sequence surveys (Fig. 1, green and red curves).

### Patient populations.

Patient samples (nasal or nasopharyngeal swab and saliva) used for SARS-CoV-2 whole-genome sequence analysis are listed in Table S1. To preserve patient confidentiality, samples were deidentified except for collection date and rationale for collection. Surveillance samples (*n* = 2621) were defined as those acquired from clinical diagnostic laboratories across the Delaware Valley (*n* = 2485), from hospitalized subjects (*n* = 116), and asymptomatic subjects testing positive in a university screening program (*n* = 20) (33). Vaccine breakthrough cases (*n* = 159) were included only if detected at least 2 weeks after the final vaccine dose (second dose for Moderna and Pfizer-BioNTech mRNA vaccines or single dose for the Johnson & Johnson adenovirus vector platform) and the subject tested positive by clinical laboratory assay. Data were not available on subject immune responses following vaccination.

10.1128/mbio.03788-21.1 TABLE S1 Human subjects and SARS-CoV-2 genome sequences analyzed in this work, including genome quality metrics, viral variant designation, and accession numbers. Download Table S1, PDF file, 0.3 MB. Copyright © 2022 Marques et al. 2022 Marques et al. https://creativecommons.org/licenses/by/4.0/ This content is distributed under the terms of the Creative Commons Attribution 4.0 International license.

As a positive control, S gene target failure samples (*n* = 172) were also compared. The TaqPath COVID-19 Combo kit by Thermo Fisher Scientific targets three regions of the SARS-CoV-2 genome for viral detection; the ORF1ab, nucleocapsid (N), and spike (S) genes. The region of the S gene interrogated by the assay overlies a characteristic deletion in the alpha variant (del 69–70), so samples containing alpha lineage virus are selectively negative for the spike amplicon, while the other two targets are detected. Samples with these characteristics were targeted for sequencing early during the wave of alpha infections to track the variant; here these spike target gene failures serve as positive controls for the statistical model.

### Sequencing strategies.

Several sequencing strategies were used to acquire whole-genome sequences. The POLAR protocol with Artic primers and Illumina sequencing was used for most samples (34). Smaller numbers of samples were acquired using the Paragon, Illumina RPIP, and Illumina CovidSeq methods. Viral genome sequences were judged to be high quality and included in the study if 95% of the viral genome was covered by at least 5 reads. In all, 2952 high-quality sequences were generated and analyzed. Viral variants were assigned using Pangolin lineage software (35, 36).

### Variants detected in pooled surveillance data.

**FIG 2:**
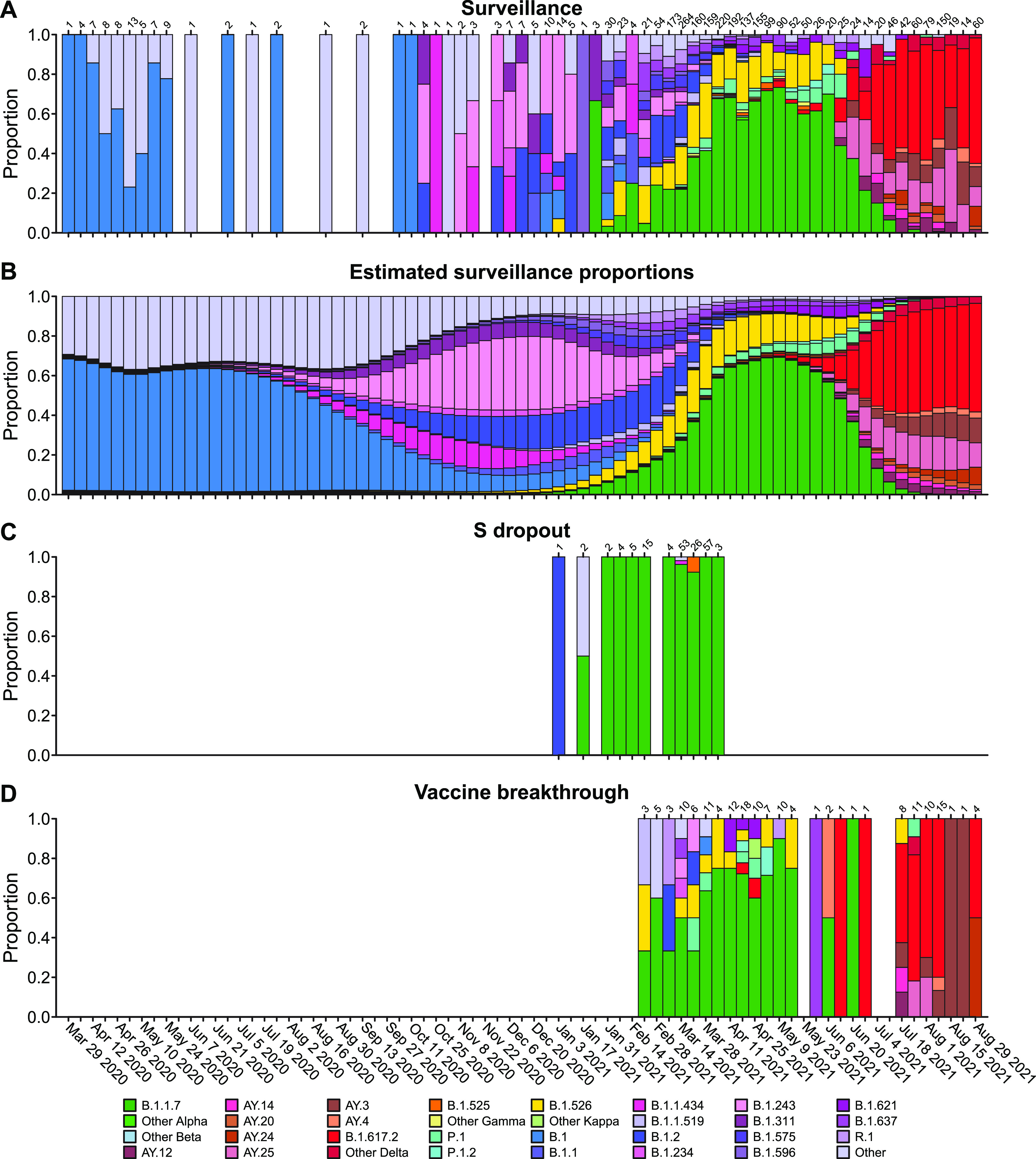
Comparison of viral genome sequence data from surveillance samples (A, B) to spike target gene failures (C) and vaccine breakthrough samples (D). (A) Longitudinal stacked bar graph depicting the SARS-CoV-2 variants present in surveillance samples from the Delaware Valley, shown as the proportion of genomes classified as each variant lineage within each week. The numbers of genomes sampled each week are shown above the graph. Variants are colored according to the key at the bottom of the figure. (B) Markings are the same as in (A), but showing the proportions of variants estimated from the count data in (A) using Bayesian autoregressive moving average multinomial logistic regression. (C) Markings as in (A), but showing counts of spike target gene failures samples. (D) Markings as in (A), but showing the counts of vaccine breakthrough samples. Designation of lineages as variants of concern and variants being monitored is presented in Table S2. For vaccine breakthroughs, the time window compared was from the introduction of widespread vaccination (March 1, 2021) to the end of our sampling period (September 3, 2021).

Figure 2A shows the proportions of variants detected in surveillance samples over the course of the study from March 2020 to September 2021. Variants detected are summarized by the color code on the bar graph (variant designations used are summarized in Table S2). The numbers of genomes analyzed per week are indicated above each column. Numbers varied both as surveillance sequencing efforts accelerated and as the availability of samples varied.

10.1128/mbio.03788-21.2 TABLE S2 Nomenclature of VBM/VOC used in this study. Download Table S2, PDF file, 0.1 MB. Copyright © 2022 Marques et al. 2022 Marques et al. https://creativecommons.org/licenses/by/4.0/ This content is distributed under the terms of the Creative Commons Attribution 4.0 International license.

Members of the B.1 lineage predominated March 2020 until fall 2020, at which time B.1.2 and B.1.243 became predominant. Later the B.1.526 and B.1.1.7 (alpha) variants emerged, with B.1.1.7 becoming predominant by spring 2021. Delta (B.1.617.2 and AY lineages) became detectable in late spring and predominant in early summer. By late summer, delta lineages were the only variants detected.

### Assessing variant abundance in the surveillance population.

Wide-spread vaccination was introduced in midwinter 2020–2021, but nevertheless breakthrough infections were detected in some vaccinated individuals, raising the question of whether specific viral features identifiable in sequence data might be associated with vaccine evasion. We sequenced 159 vaccine breakthrough samples collected between February 22 and September 3, 2021, and compared the distributions of lineages or genomic variations to those observed in surveillance samples from the same community (*n* = 2621).

Challenges in the analysis include the facts that: (i) the distribution of variants in the surveillance samples is changing over time; (ii) sampling is uneven over time; and 3 sampling is subject to stochastic fluctuations. To address these issues, we developed a model based on Bayesian analysis that combines an autoregressive moving average model with multinomial logistic regression. The underlying variant proportions at each week of the study were estimated from the counts of SARS-CoV-2 variants assuming relatively smooth changes over time and accounting for stochastic fluctuations during sampling (Fig. 2B). These estimated surveillance proportions were compared to the variant counts observed in spike gene target failure samples (Fig. 2C) and vaccine breakthrough samples (Fig. 2D). Since the surveillance population proportions were estimated for each week, the weekly vaccine breakthrough or spike gene target failure variant counts could be compared to the corresponding time-matched surveillance estimates.

**FIG 3:**
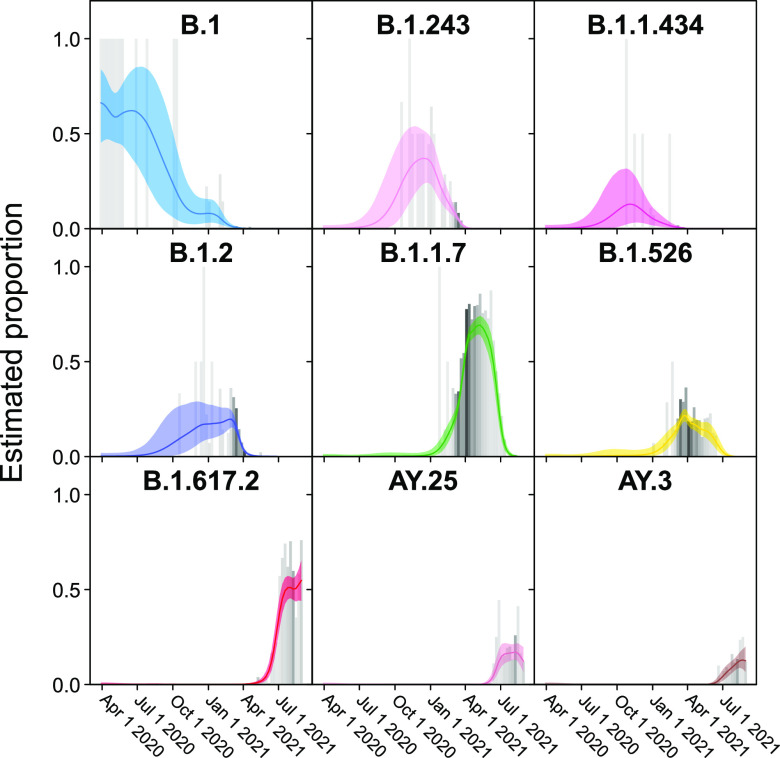
Frequencies of individual variants estimated using Bayesian autoregressive moving average multinomial logistic regression. Time is shown along the *x* axis and estimated proportions of the surveillance population along the *y* axis. The gray bars indicate raw proportions from the count data shaded by the number of observations observed in a given week (darker indicating more samples) while the colored lines indicate the proportion estimated by the Bayesian model. The light-colored envelopes around each line show the 95% credible intervals for the proportion. Only lineages achieving an estimated proportion of >10% in any given week are shown.

Examples of the temporal profiles of the modeled lineage succession are shown in more detail in Fig. 3. One benefit of the model is that uncertainty in the surveillance estimates can be included. For example, note that time points with fewer surveillance samples (lighter gray bars) have larger 95% credible intervals for the proportion estimate (colored shading). From this view, it is evident that several further lineages waxed and waned notably over the sampling period, including B.1.1.434 and B.1.526.

### Analyzing lineages in spike gene target failures.

**FIG 4:**
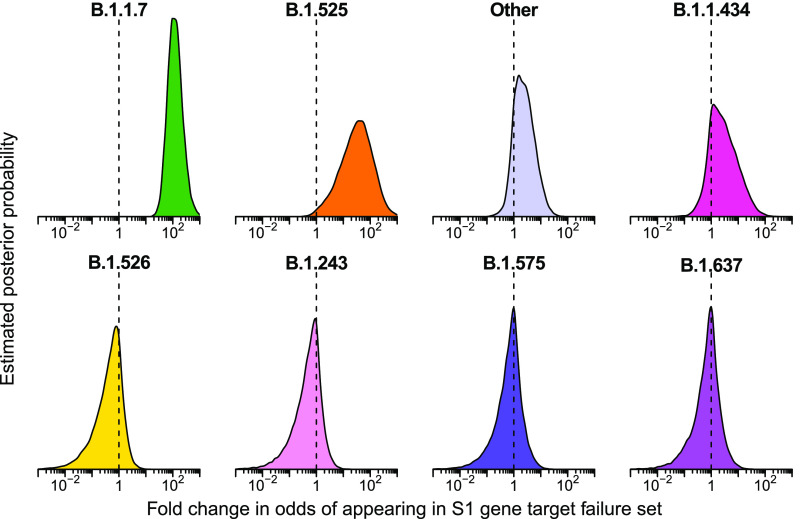
Estimated posterior probability densities for the enrichment of variants among spike gene target failures produced by Bayesian autoregressive moving average multinomial logistic regression. The *x* axis shows the fold enrichment/depletion in the odds of a variant (labeled above each plot) appearing in the spike gene target failure set relative to the proportions estimated in the surveillance population and the *y* axis shows the posterior probability. No enrichment (fold change of 1) is indicated by the dashed vertical line. Increased density to the right indicates greater likelihood of inclusion among spike gene target failures, increased density to the left indicates decreased likelihood. The four lineages with highest posterior probability of enrichment are shown on the top and four lineages with high posterior probability of depletion are shown on the bottom. Color coding indicates the variant queried with colors as in earlier figures.

As a control, we assessed the variants associated with spike (S) gene target failure samples (Fig. 2C and 4 and Table S3), where an amplicon overlapping the 69–70 deletion in spike failed selectively. To track the spread and variation of B.1.1.7 during the alpha wave of infection, we sequenced 172 S gene target failure samples in the Delaware Valley from January to April 2021. Upon variant assignment, genomes from 96.1% of spike target gene failure samples corresponded to B.1.1.7. Rarely, other lineages were seen that share the spike deletion with B.1.1.7 (B.1.375 and B.1.525) or were likely stochastic failures of S amplification or genomes with novel combinations of mutations.

10.1128/mbio.03788-21.3 TABLE S3 Estimated fold enrichment in odds of appearing in the spike gene target failure set for each SARS-CoV-2 lineage. Download Table S3, PDF file, 0.03 MB. Copyright © 2022 Marques et al. 2022 Marques et al. https://creativecommons.org/licenses/by/4.0/ This content is distributed under the terms of the Creative Commons Attribution 4.0 International license.

As expected, we found that the B.1.1.7 lineage was estimated to be highly enriched in the S gene target failure set over what would be expected from its frequency in the surveillance lineage counts (Fig. 4) (odds ratio of 120; 95% credible interval 38–470). The B.1.525 lineage, which also contains the S69-70 deletion, was estimated to be enriched as well (odds ratio 33; 95% credible interval 1.7–380). Note that this enrichment was detected based on counts of only two B.1.525 genomes in the S gene target failure set and 12 surveillance genomes, both emphasizing the sensitivity of the model and highlighting that the detection of enrichment can be easier in lineages rare in the surveillance population. Other lineages showed little association with spike target gene failures, as expected (note that only a single B.1.375 lineage sample was observed and thus it did not reach the threshold for lineage-specific analysis and was grouped in “Other”). Thus, the analysis of S target failures confirmed that the Bayesian autoregressive moving average categorical regression model was effective at identifying overrepresented lineages relative to the time-adjusted expectation from surveillance.

### Enrichment of delta variant B.1.617.2 in vaccine breakthrough samples.

**FIG 5:**
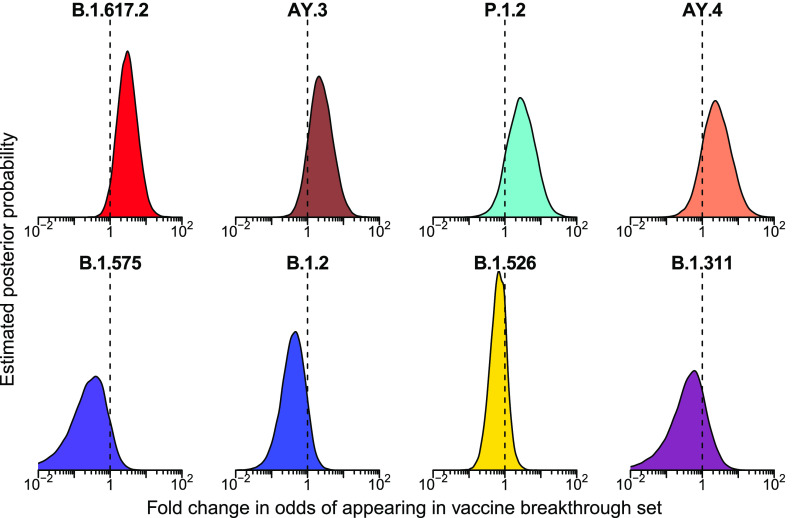
Posterior probability densities for enrichment of variants among vaccine breakthrough samples compared to the surveillance population as estimated by Bayesian autoregressive moving average multinomial logistic regression. Markings as in Fig. 4

We then applied the Bayesian model to analyzing vaccine breakthrough samples (Fig. 2D and 5 and Table S4). There was less clear enrichment in vaccine breakthroughs than in the S gene target failure set. The delta lineage B.1.617.2 did show signs of enrichment in breakthrough samples, with mean odds ratio of 3 (95% credible interval 0.89 to 11). The one-sided posterior probability of any enrichment for B.1.617.2 in vaccine breakthrough was estimated at 96% with a 73% probability of more than a 2-fold enrichment in odds of appearing in vaccine breakthrough. This enrichment was not observed for all lineages grouped within delta, with both a group-wise estimate for all delta lineages and the specific estimates for the other delta AY lineages, including AY.3, AY.4, AY.14, and AY.24, showing little enrichment over surveillance. These alternative delta lineages also had fewer samples observed, so a lack of power may be a partial explanation. Note that the power to detect enrichment of delta is limited by its rapid spread–observation of a delta vaccine breakthrough case at a time point when almost all surveillance population infections were also delta provides little information. Similarly, lineages that had already waned by the time of widespread vaccination are difficult to assess since there was little chance for them to appear in vaccine breakthrough cases.

10.1128/mbio.03788-21.4 TABLE S4 Estimated fold enrichment in odds of appearing in vaccine breakthrough samples for each SARS-CoV-2 lineage. Download Table S4, PDF file, 0.03 MB. Copyright © 2022 Marques et al. 2022 Marques et al. https://creativecommons.org/licenses/by/4.0/ This content is distributed under the terms of the Creative Commons Attribution 4.0 International license.

### Assessing possible enrichment of specific mutations in spike gene target failures and vaccine breakthroughs.

**FIG 6:**
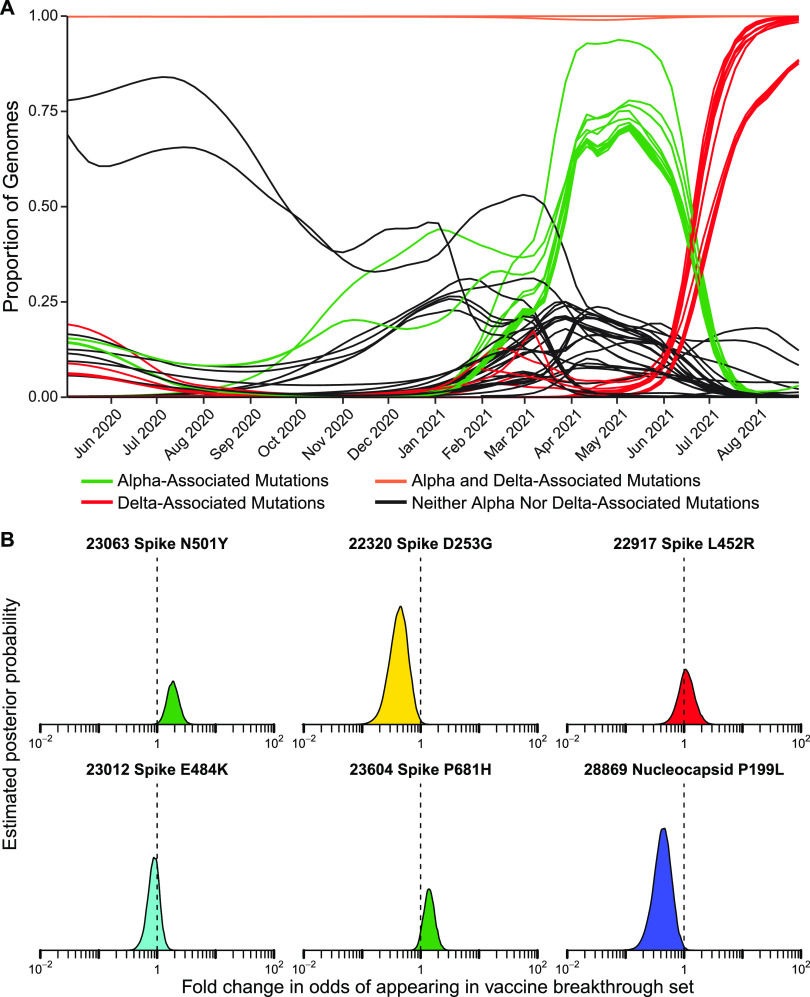
Assessment of enrichment of specific base substitutions and deletions in vaccine breakthrough samples. (A) Longitudinal frequencies of individual mutations estimated using Bayesian autoregressive moving average logistic regression models. Red indicates mutations commonly found in delta lineages. Green indicates mutations commonly found in alpha lineages. Orange indicates mutations shared by almost all lineages in the study. Black indicates mutations found in other subsets of lineages. (B) Estimated posterior probability densities summarizing the fold enrichment/depletion in odds of a mutation appearing in the vaccine breakthrough samples over its proportions estimated from surveillance samples. The mutations estimated as most enriched and most depleted are shown along with other mutations of interest. Markings as in Fig. 4 and 5.

We next investigated whether any individual base substitutions in the viral genome were selectively associated with spike gene target failure and vaccine breakthrough samples. A summary of mutations detected is presented in Table S5. We adapted the Bayesian autoregressive moving average logistic regression method to assess the behavior of single mutations and amino acid substitutions. Fig. 6 and Table S6 summarizes results. Many mutations varied in frequency over the course of the study often paralleling the profile of the most abundant lineages containing them (Fig. 6A).

10.1128/mbio.03788-21.5 TABLE S5 The percentage of genomes within each SARS-CoV-2 lineage containing each substitution or deletion. Download Table S5, PDF file, 1.9 MB. Copyright © 2022 Marques et al. 2022 Marques et al. https://creativecommons.org/licenses/by/4.0/ This content is distributed under the terms of the Creative Commons Attribution 4.0 International license.

10.1128/mbio.03788-21.6 TABLE S6 Estimated fold enrichment in odds of appearing in the spike gene target failure set for each substitution or deletion studied. Download Table S6, PDF file, 0.04 MB. Copyright © 2022 Marques et al. 2022 Marques et al. https://creativecommons.org/licenses/by/4.0/ This content is distributed under the terms of the Creative Commons Attribution 4.0 International license.

As expected, the 69–70 deletion was found to be highly enriched in the S gene target failure set (odds ratio of 205, 95% credible interval of 65–581). Enrichment among S gene target failures was also seen for another 28 mutations (Table S6), where all the most strongly enriched substitutions were characteristic of the alpha variant and thus due to “hitchhiker” effects. Underrepresentation in spike target gene failures was also seen in mutations in multiple additional open reading frames, reflecting mutations found in variants lacking the 69–70 deletion. Each mutation was analyzed separately without accounting for genomic linkage so this is as expected.

For vaccine breakthroughs (Fig. 6B and Table S7), the most notably enriched substitution was N501Y, which showed an odds ratio of 2.04 (95% credible interval of 1.25–3.18). The N501Y substitution is found in multiple VBM, including alpha, beta, and gamma, and is reported to increase the affinity of spike protein binding to the ACE2 receptor (23, 37, 38) and to diminish binding of some human antibodies to spike (24). The spike substitution P681H was also slightly enriched; this substitution is near the furin cleavage site and may promote efficient proteolysis. Several additional substitutions in spike were potentially enriched (D614G, P681R, D950N), but the 95% credible interval included one, so the evidence for enrichment was weaker (Table S7). Two other closely studied spike substitutions, E484K and L452R, were not notably enriched among vaccine breakthrough cases, and the D253G substitution was modestly depleted. Among non-spike substitutions, the P199L substitution in the nucleocapsid showed notable depletion (Table S7). Stepping back, these findings together emphasize the potential importance of the N501Y substitution in vaccine breakthrough.

10.1128/mbio.03788-21.7 TABLE S7 Estimated fold enrichment in odds of appearing in the vaccine breakthrough samples for each substitution or deletion studied. Download Table S7, PDF file, 0.04 MB. Copyright © 2022 Marques et al. 2022 Marques et al. https://creativecommons.org/licenses/by/4.0/ This content is distributed under the terms of the Creative Commons Attribution 4.0 International license.

## DISCUSSION

Vaccination against SARS-CoV-2 has been highly effective at preventing infection, hospitalization and death. However, infections occasionally take place despite vaccination. It has been suggested that certain lineages of SARS-CoV-2 are more prone to evade vaccination, however, interpretation of counts of vaccine breakthrough is difficult due to variation over time in circulating lineages, the numbers of vaccinated individuals, times since vaccination and the application of nonpharmaceutical interventions. Here, we introduce modeling based on Bayesian autoregressive moving average multinomial logistic regression, which estimates the proportions of lineages in the surveillance data over time, allowing comparison of lineage counts in populations of interest to time-matched surveillance estimates. Multiple studies have investigated whether certain lineages are more likely to appear in vaccine breakthrough infections (7, 8, 39–43). However, few studies have as large numbers of sequenced samples for both background surveillance and for vaccine breakthrough cases within a matched geographical region as reported here. Thus, our contribution provides targeted data on the nature of breakthroughs at nucleotide resolution.

Using this method, we found that there was evidence for enrichment in the odds of variant lineages appearing in the vaccine breakthrough population relative to the general surveillance population, with the delta variant B.1.617.2 showing the strongest signal with an estimated enrichment of 3-fold (95% credible interval 0.89 to 11). Several previous studies have also noted delta variants to be enriched among vaccine breakthroughs (7, 8); our data provides further support based on careful statistical analysis.

Using a similar Bayesian autoregressive moving average logistic regression model, we interrogated enrichment of point substitutions among vaccine breakthrough cases. The N501Y substitution stood out as particularly associated with vaccine breakthroughs. N501Y is found in several of the VBM, and this substitution is well known to increase affinity of binding for the viral spike protein to the ACE2 receptor (23, 37, 38), as well as reduce antibody binding to promote immune evasion (24). Recently the N501Y substitution was suggested to be a central feature in a meta-signature of up to 35 mutations which recur in alpha, beta, gamma and other lineages, and which mark a viral fitness peak reflecting optimization for spread in humans (44). In contrast, several substitutions were estimated to be less likely to appear in vaccine breakthrough samples, including nucleocapsid mutation P199L (detected in B.1.2, B.1.526, and B.1.596; odds ratio of 0.5; 95% credible interval of 0.24–0.88), and spike mutation D253G (detected in B.1.526; odds ratio of 0.5; 95% credible interval of 0.23–0.89). The spike D253G substitution has been implicated in MAb evasion (45), and nucleocapsid P199L may alter the assembly of SARS-CoV-2 VLPs (46), functions possibly linked to their depletion in vaccine breakthrough.

This study has several limitations. For the vaccine breakthrough samples, we did not have paired immunological data, so it is unknown whether the vaccinations provoked protective immune responses. Our surveillance data were from a mixture of hospitalized patients, symptomatic subjects tested in clinical diagnostic laboratories, and asymptomatic subjects tested weekly at an academic institution, and so our sampling may not be entirely representative of viruses circulating in the community. Several different amplification and sequencing methods were used to acquire data. Documentation of vaccination status may be incomplete. For all the viral genome sequencing, only samples achieving a threshold level of viral RNA could be sequenced (roughly Ct <28 for swab-based testing, Ct <20 for saliva), so it is possible that other variants predominate in subjects with lower viral loads. Studies have been initiated to address some of these concerns.

In summary, similar to other regions in the US and around the world, VBM/VOC and other lineages waxed and waned in the Delaware Valley, with delta lineages ultimately comprising all recent samples in the fall of 2021. Widespread vaccination was introduced in the region in winter 2020–2021, and increasing numbers of breakthrough infections have since been detected. To compare the lineages found in breakthrough with those observed in general surveillance, we introduce analysis based on Bayesian autoregressive moving average multinomial logistic regression and show that delta variant B.1.617.2 showed 3-fold enrichment in vaccine breakthroughs. The N501Y substitution stood out among point substitutions for enrichment in vaccine breakthroughs. We expect that these modeling methods will be useful in monitoring the effectiveness of vaccination programs going forward as novel variants of SARS-CoV-2 continue to emerge.

## MATERIALS AND METHODS

### Human subjects.

The University of Pennsylvania Institutional Review Board (IRB) reviewed the research protocol and deemed the limited data elements extracted with positive SARS-CoV-2 specimens to be exempt from human subject research per 45 CFR 46.104, category 4 (IRB #848605). For hospitalized subjects at the University of Pennsylvania, following informed consent (IRB protocol #823392), patients were sampled by collection of saliva, oropharyngeal and/or nasopharyngeal swabs, or endotracheal aspirates if intubated, as previously described (32). Clinical data were extracted from the electronic medical record. Further samples were collected from asymptomatic subjects detected in a screening program at the Perelman School of Medicine at the University of Pennsylvania and symptomatic subjects tested throughout the PennMedicine clinical network under IRB protocols #843565 and #848608. Human samples were collected at Children’s Hospital of Philadelphia under protocol # 21-018726 approved by the Children’s Hospital of Philadelphia IRB. Human samples were collected at Thomas Jefferson University under protocol number IRB Control # 21E.441 approved by the Thomas Jefferson University IRB. Vaccine breakthrough cases were identified by either report to HUP Infection and Control, Jefferson Infection and Control, CHOP Department of Infection Control and Prevention, the Philadelphia Department of Public Health, or, where applicable, chart review.

### Sequencing methods.

Several sequencing methods were used to acquire viral whole-genome sequences.

The POLAR protocol was used to acquire the majority of viral genome sequences (47). Illumina’s NextSeq instrument was used to gather sequence data. In detail, 5 μl of viral RNA, 0.5 μl of 50 μM Random Hexamers (Thermo Fisher, N8080127), 0.5 μl of 10 mM dNTPs Mix (Thermo Fisher, 18427013), and 1 μl nuclease-free water was incubated at for 5 min at 65°C proceeded by a 1 min incubation at 4°C. To perform reverse transcription, 6.5 μl from the previous reaction, 0.5 μl SuperScript III Reverse Transcriptase (Thermo Fisher, 18080085), 0.5 μl of 0.1M DTT (Thermo Fisher, 18080085), 0.5 μl of RNaseOut (Thermo Fisher, 18080051), and 2 μl of 5X First-Strand Buffer (Thermo Fisher, 18080085) was incubated for 50 min at 42°C, followed by an incubation for 10 min at 70°C, and then held at 4°C. To amplify the cDNA, artic-ncov2019 version 3 primers were used (IDT). To perform PCR amplification of the viral cDNA, the following reagents were added to 2.5 μl of the previous mixture: 0.25 μl Q5 Hot Start DNA polymerase (NEB, M0493S), 5 μl of 5X Q5 Reaction Buffer (NEB, M0493S), 0.5 μl of 10 mM dNTPs Mix (NEB, N0447S), either 4.0 μl of pooled primer set 1 or 3.98 μl of pooled primer set 2, and nuclease-free water to bring to a final volume of 25 μl. This PCR amplification of the viral cDNA used the following conditions: 98°C for 30 s for 1 cycle, 25 cycles at 98°C for 15 s and 65°C for 5 min, and then held at 4°C. Amplicons generated by the two primer sets from the same sample were pooled then diluted to a concentration of 0.25 ng/μl. The Nextera library was prepared using the Nextera XT Library Preparation kit (Illumina, FC-131-1096) and the IDT for Illumina DNA/RNA UD Indexes Set A and B (Illumina, 20027213, 20027214, 20027215, 20027216). The Quant-iT PicoGreen dsDNA quantitation assay kit was used to quantify the DNA of each sample (Invitrogen, P7589). The samples were pooled in equal quantities, and the pooled library was quantified using the Qubit1X dsDNA HS assay kit (Invitrogen, Q33230). The library was sequenced on an Illumina NextSeq.

Another sequencing method, used at CHOP, was Paragon. Paragon sequencing was carried out as in (22). Briefly, RNA was extracted from nasopharyngeal swab samples using QIAamp Viral RNA Mini (Qiagen). Whole-genome sequencing was carried out by the Genomics Core Facility at Drexel University. Amplification was performed using Paragon Genomics CleanPlex SARS-CoV-2 Research and Surveillance NGS Panel 1 and 2. Libraries were quantified using the Qubit dsDNA HS (High Sensitivity) assay kit (Invitrogen) with the Qubit Fluorometer (Invitrogen). Library quality was assessed using Agilent High Sensitivity DNA kit and the 2100 Bioanalyzer instrument (Agilent). Libraries were normalized to 5 nM and pooled in equimolar concentrations. The resulting pool was quantified again using the Qubit dsDNA HS (High Sensitivity) assay kit (Invitrogen) and diluted to a final concentration of 4 nM; libraries were denatured and diluted according to Illumina protocols and loaded on the MiSeq at 10pM. Paired-end and dual-indexed 2x150bp sequencing was carried out using MiSeq Reagent Kits v3 (300 cycles).

The Thomas Jefferson University site carried out sequencing using the Illumina RPIP and CovidSeq methods essentially as per the manufacturer’s instructions. A nasopharyngeal swab specimen that was tested positive by PCR for SARS-CoV-2 was used for this study. Vaccine breakthrough specimens were identified by Jefferson Occupational Health Network for Employees and Students (JOHN) and were sequenced with a designation of VBT. Randomly selected residual positive specimens from the Molecular & Genomic Pathology Laboratory at Jefferson during the same period were sequenced for the purpose of epidemiology surveillance and labeled as SURV. RNA was extracted from 200 μl of the specimen and eluted in 110 μl using the bioMérieux EasyMag Extraction System, following EasyMag’s Generic protocol. Whole-genome sequencing for SARS-CoV-2 was subsequently performed at the Molecular & Genomic Pathology Laboratory at Thomas Jefferson University Hospital. With the exception of a few samples, samples were sequenced using Illumina COVIDSeq protocol (Illumina) following manufacturer’s guideline. Remaining specimens were sequenced using the Swift Normalase Amplicon Panels (SNAP) Core kit along with the SARS-CoV-2 Additional Genome Coverage Primer Panel following the manufacturer’s protocol. For data analysis, Illumina’s Local Run Manager’s GenerateFASTQ module was used to generate the fastq files for all specimens. The fastq files were transferred to UPenn’s secure data server for further processing to obtain QC metrics and lineage data.

Samples with VSP numbers lower than VSP00256 (Table S1) were previously described in Everett et al. (32).

### Data analysis.

To process sequence data, sequence reads are trimmed to remove low quality base calls (< Q20) and aligned to the original Wuhan reference sequence (NC_045512.2) with the BWA aligner tool (v0.7.17) (48), after which alignments are filtered with the Samtools package (v1.10) (49). Sequencing depth is determined for each position in the viral genome and genomes are accepted for analysis when ≥ 95% of genome positions are covered with a read depth of ≥ 5 reads. Variant positions are called with the Bcftools package (v1.10.2-34) (50) requiring PHRED scores ≥ 20 and variant read frequencies ≥ 50% of the total reads. The nature of substitutions is determined by retrieving reading frames from the reference GENBANK record, translating, and determining the native and mutant residues.

Variants were assigned using the Pangolin lineage software (Pangolin version 3.1.11 with the PangoLEARN 2021-08-24 release). Note that these lineages are updated regularly and so are expected to change over time. Point mutations were assigned using a previously described bioinformatics pipeline (31).

Statistical analysis of variant enrichment and mutation enrichment in subsets of the data were separately assessed using a Bayesian model and Markov chain Monte Carlo sampling implemented in Stan (51). The model takes the vector of counts of the variants or mutations seen in the surveillance sampling for a given week, counts_i_, and assumes they are multinomially distributed where:

$${\text{counts}}_{\text{w}}\sim \text{Multinomial(}p_{*,w}\text{)}$$

with probabilities modeled as multinomial logistic:

$$p_{i,w}\;=\;\frac{e^{x_{i,w}}}{\sum_{j=1}^{n}e^{x_{j,w}}}$$

where p_i,w_ is the true proportion of variant or mutation *i* on week w. p_*,w_ indicates the vector of all lineage probabilities for week *w* and *n* is the total number of variants or mutations observed. The underlying proportions for a given week are assumed to be centered around the proportions observed in the prior week (autoregressive) plus a change term that is itself correlated with the change observed in the prior week (moving average):

$$x_{i,w}\;=x_{i,w-1}\;+\;\mathrm{chang}e_{i,w}$$

$${\text{change}}_{\text{i,w}}\;\sim \;\text{Normal}\left(\mathrm{chang}e_{i,w-1},\text{σ}\right)$$

where *chang*e*_i,w_* is the change in log odds of variant or mutation *i* on week *w*. The initial starting proportions are given flat priors:

$$x_{i,1}\;\text{=}\;\{\begin{array}{cc} 0, & i=1\\ \text{Normal}(0,10), & i\;>1 \end{array}$$

The standard deviation for changes, σ, was given a *Gamma*(1, 2) prior distribution.

To assess if there's enrichment of a particular variant or mutation in the vaccine breakthrough, counts of vaccine breakthrough samples for a given week, vaccineCounts_w_, were modeled as:

$${\text{vaccineCounts}}_{\text{w}}\;\sim \;\text{Multinomial}\left(v_{*,w}\right)$$

where:

$$v_{i,w}=\frac{e^{y_{i,w}}}{\sum_{j=1}^{n}e^{y_{j,w}}}$$

and

$$y_{i,w}=x_{i,w}\;+\;\beta_{i}\;+\;\delta_{\mathrm{grou}p_{i}}$$

where *β_i_* measures the log odds enrichment/depletion of variant or mutation *i* in the vaccine breakthrough population over the surveillance population, *group_i_* indicates the WHO classification for Pango lineage *i* and *δ_group__i_* indicates the enrichment or depletion for a CDC VBM/VOC classification containing more than one Pango lineage, e.g., Delta containing B.1.617.2 and other variants with an AY prefix. In these data, Delta, Gamma and Non-VBM/VOC groupings contained more than one lineage above the abundance threshold. For WHO classifications containing only a single lineage, *δ_group__i_* was set to 0. The *β* and remaining *δ* were given DoubleExponential(0,1) priors.

This was repeated equivalently in the samples collected as S gene target failures, with the counts, SFailCounts_w_, modeled as:

$${\text{SFailCounts}}_{\text{w}}\;\sim \;\text{Multinomial}\left({\text{u}}_{*\text{,w}}\right)$$

where:

$$u_{i,w}=\frac{e^{z_{i,w}}}{\sum_{j=1}^{n}e^{z_{j,w}}}$$

and

$$z_{i,w}=x_{i,w}\;+\;\alpha_{i}$$

The α were given DoubleExponential(0,1) priors.

For the assessment of lineage enrichment, genomes from lineages that had more than 10 genomes assigned to them were included as their individual lineages, genomes from lineages with 10 or fewer assignments that were listed within VBM/VOC were included as miscellaneous classification for their particular WHO category, e.g., “Other delta” and all other genomes were grouped into an Other category. For the assessment of mutations, each mutation that was found in greater than 5% of genomes was independently modeled as above but replacing the Multinomial distributions with Binomial and removing the inapplicable lineage group *δ* terms.

### Data availability.

All viral genome sequences acquired in this study have been deposited in GISAID and at NCBI under accession numbers listed in Table S1. Analysis code is archived on Zenodo at https://doi.org/10.5281/zenodo.5888338. Sequence processing software and intermediate files used in this study are available at https://doi.org/10.5281/zenodo.5559699. A list of key reagents used in this study is in Table S8.

10.1128/mbio.03788-21.8 TABLE S8 Key reagents. Download Table S8, PDF file, 0.04 MB. Copyright © 2022 Marques et al. 2022 Marques et al. https://creativecommons.org/licenses/by/4.0/ This content is distributed under the terms of the Creative Commons Attribution 4.0 International license.
