# Supplementary material for: SARS-CoV-2 Variants Associated with Vaccine Breakthrough in the Delaware Valley through Summer 2021
Source: mBio. 2022 Feb 8;13(1):e03788-21. doi: 10.1128/mbio.03788-21 (PMC8942461; doi:10.1128/mbio.03788-21)
Supplement: TABLE S1 [file mbio.03788-21-st001.pdf]

Table S1 Human subjects and SARS-CoV-2 genome sequences analyzed in this work, including genome quality metrics, viral variant designation, and accession numb

| VSP | Pango_Lineage | Collection_Date | Rationale | Percent_5x_Coverage | Mean_Coverage | GISAID_ID | NCBI_Accession |
|---|---|---|---|---|---|---|---|
| VSP0009 | B.1 | 3/30/20 | hospitalized | 99.7 | 1276 | hCoV-19/USA/VSP0009/2020 | MW001232 |
| VSP0010 | B.1 | 4/8/20 | hospitalized | 95.1 | 306 | hCoV-19/USA/VSP0010/2020 | MW001233 |
| VSP0011 | B.1 | 4/8/20 | hospitalized | 99.4 | 3682 | hCoV-19/USA/VSP0011/2020 | MW001234 |
| VSP0012 | B.1 | 4/8/20 | hospitalized | 99.7 | 2208 | hCoV-19/USA/VSP0012/2020 | MW001235 |
| VSP0013 | B.1 | 4/13/20 | hospitalized | 99.7 | 2249 | hCoV-19/USA/VSP0013/2020 | MW001236 |
| VSP0014 | B.1.564 | 4/15/20 | hospitalized | 99.6 | 900 | hCoV-19/USA/VSP0014/2020 | MW001240 |
| VSP0020 | B.1.520 | 4/22/20 | hospitalized | 99.7 | 3499 | hCoV-19/USA/VSP0020/2020 | MW001245 |
| VSP0021 | B.1 | 4/22/20 | hospitalized | 99.7 | 6690 | hCoV-19/USA/VSP0021/2020 | MW001246 |
| VSP0022 | B.1 | 4/22/20 | hospitalized | 98.9 | 499 | hCoV-19/USA/VSP0022/2020 | MW001248 |
| VSP0039 | B.1.564 | 4/29/20 | hospitalized | 99.7 | 5425 | hCoV-19/USA/VSP0039/2020 | MW001254 |
| VSP0041 | B.1.369 | 4/29/20 | hospitalized | 99.7 | 7099 | hCoV-19/USA/VSP0041/2020 | MW001255 |
| VSP0042 | B.1.369 | 4/29/20 | hospitalized | 64.5 | 3803 | hCoV-19/USA/VSP0042/2020 | MW001256 |
| VSP0044 | B.1 | 4/29/20 | hospitalized | 99.7 | 3408 | hCoV-19/USA/VSP0044/2020 | MW001259 |
| VSP0045 | B.1 | 4/29/20 | hospitalized | 99.1 | 3866 | hCoV-19/USA/VSP0045/2020 | MW001261 |
| VSP0046 | B.1 | 5/1/20 | hospitalized | 99.7 | 3686 | hCoV-19/USA/VSP0046/2020 | MW001257 |
| VSP0047 | B.1 | 5/1/20 | hospitalized | 99.7 | 4438 | hCoV-19/USA/VSP0047/2020 | MW001260 |
| VSP0050 | B.1 | 5/1/20 | hospitalized | 96.6 | 3443 | hCoV-19/USA/VSP0050/2020 | MW001262 |
| VSP0052 | B.1 | 5/4/20 | hospitalized | 99.6 | 4519 | hCoV-19/USA/VSP0052/2020 | MW001258 |
| VSP0065 | B.1.439 | 5/4/20 | hospitalized | 99.7 | 5980 | hCoV-19/USA/VSP0065/2020 | MW001264 |
| VSP0080 | B.1.520 | 4/24/20 | hospitalized | 99.7 | 5775 | hCoV-19/USA/VSP0080/2020 | MW001250 |
| VSP0088 | B.1.439 | 5/6/20 | hospitalized | 99.7 | 3435 | hCoV-19/USA/VSP0088/2020 | MW001263 |
| VSP0089 | B.1.439 | 5/6/20 | hospitalized | 99.6 | 5869 | hCoV-19/USA/VSP0089/2020 | MW001265 |
| VSP0100 | B.1.520 | 5/8/20 | hospitalized | 99.6 | 3781 | hCoV-19/USA/VSP0100/2020 | MW001266 |
| VSP0102 | B.1 | 5/8/20 | hospitalized | 99.7 | 6335 | hCoV-19/USA/VSP0102/2020 | MW001269 |
| VSP0107 | B.1.520 | 5/8/20 | hospitalized | 99.7 | 8815 | hCoV-19/USA/VSP0107/2020 | MW001268 |
| VSP0123 | B.1.520 | 5/11/20 | hospitalized | 99.7 | 4433 | hCoV-19/USA/VSP0123/2020 | MW001267 |
| VSP0140 | B.1.564 | 5/15/20 | hospitalized | 96 | 77 | hCoV-19/USA/VSP0140/2020 | MW001270 |
| VSP0141 | B.1.564 | 5/15/20 | hospitalized | 99.7 | 1278 | hCoV-19/USA/VSP0141/2020 | MW001271 |
| VSP0142 | B.1 | 5/15/20 | hospitalized | 99.6 | 1369 | hCoV-19/USA/VSP0142/2020 | MW001272 |
| VSP0143 | B.1 | 5/15/20 | hospitalized | 99.7 | 5818 | hCoV-19/USA/VSP0143/2020 | MW001273 |
| VSP0155 | B.1 | 5/20/20 | hospitalized | 98.5 | 624 | hCoV-19/USA/VSP0155/2020 | MW001274 |
| VSP0163 | B.1 | 5/22/20 | hospitalized | 99.7 | 8029 | hCoV-19/USA/VSP0163/2020 | MW001275 |
| VSP0166 | B.1.564 | 5/22/20 | hospitalized | 99.5 | 1908 | hCoV-19/USA/VSP0166/2020 | MW001280 |
| VSP0176 | B.1.564 | 5/27/20 | hospitalized | 99.7 | 1921 | hCoV-19/USA/VSP0176/2020 | MW001278 |
| VSP0179 | B.1 | 5/27/20 | hospitalized | 99.7 | 4022 | hCoV-19/USA/VSP0179/2020 | MW001282 |
| VSP0180 | B.1 | 4/17/20 | hospitalized | 99.7 | 1671 | hCoV-19/USA/VSP0180/2020 | MW001241 |
| VSP0181 | B.1 | 4/17/20 | hospitalized | 99.7 | 4741 | hCoV-19/USA/VSP0181/2020 | MW001242 |
| VSP0182 | B.1 | 4/20/20 | hospitalized | 99.7 | 2751 | hCoV-19/USA/VSP0182/2020 | MW001243 |
| VSP0183 | B.1 | 4/17/20 | hospitalized | 99.8 | 2270 | hCoV-19/USA/VSP0183/2020 | MW001244 |
| VSP0184 | B.1 | 4/8/20 | hospitalized | 99.7 | 2000 | hCoV-19/USA/VSP0184/2020 | MW001238 |
| VSP0185 | B.1 | 4/13/20 | hospitalized | 99.7 | 2015 | hCoV-19/USA/VSP0185/2020 | MW001239 |
| VSP0186 | B.1 | 4/13/20 | hospitalized | 99.7 | 1296 | hCoV-19/USA/VSP0186/2020 | MW001237 |
| VSP0188 | B.1 | 5/29/20 | hospitalized | 99.7 | 11443 | hCoV-19/USA/VSP0188/2020 | MW001247 |
| VSP0189 | B.1 | 5/29/20 | hospitalized | 99.7 | 1229 | hCoV-19/USA/VSP0189/2020 | MW001249 |
| VSP0195 | B.1 | 5/29/20 | hospitalized | 98.8 | 1847 | hCoV-19/USA/VSP0195/2020 | MW001281 |

| | | | | | | | |
|---|---|---|---|---|---|---|---|
| VSP0196 | B.1 | 5/29/20 | hospitalized | 98.3 | 66 | hCoV-19/USA/VSP0196/2020 | MW001283 |
| VSP0202 | B.1.564 | 6/12/20 | hospitalized | 99.7 | 3801 | hCoV-19/USA/VSP0202/2020 | MW001279 |
| VSP0230 | B.1 | 6/29/20 | hospitalized | 99.7 | 7948 | hCoV-19/USA/VSP0230/2020 | MW001284 |
| VSP0233 | B.1 | 7/1/20 | hospitalized | 99.7 | 7982 | hCoV-19/USA/VSP0233/2020 | MW001285 |
| VSP0256 | B.1.240 | 7/17/20 | hospitalized | 99 | 14869 | hCoV-19/USA/VSP0256/2020 | MW001286 |
| VSP0262 | B.1 | 5/20/20 | hospitalized | 99.7 | 5006 | hCoV-19/USA/VSP0262/2020 | MW001276 |
| VSP0263 | B.1 | 5/22/20 | hospitalized | 99.5 | 1365 | hCoV-19/USA/VSP0263/2020 | MW001277 |
| VSP0266 | B.1 | 7/27/20 | hospitalized | 99.7 | 16111 | hCoV-19/USA/VSP0266/2020 | OK245577 |
| VSP0271 | B.1 | 7/29/20 | hospitalized | 99.7 | 10930 | hCoV-19/USA/VSP0271/2020 | OK245578 |
| VSP0318 | B.1.439 | 5/6/20 | hospitalized | 99 | 661 | hCoV-19/USA/PA-VSP0318/2020 | OK245579 |
| VSP0319 | B.1 | 5/20/20 | hospitalized | 98.5 | 452 | hCoV-19/USA/PA-VSP0319/2020 | OK245580 |
| VSP0320 | B.1 | 5/22/20 | hospitalized | 99.7 | 2633 | hCoV-19/USA/PA-VSP0320/2020 | OK245581 |
| VSP0327 | B.1.340 | 8/26/20 | hospitalized | 99.7 | 7863 | hCoV-19/USA/VSP0327/2020 | OK245582 |
| VSP0356 | B.1.543 | 9/16/20 | hospitalized | 99.7 | 13848 | hCoV-19/USA/VSP0356/2020 | OK245583 |
| VSP0359 | B.1.240 | 9/16/20 | hospitalized | 97.1 | 10757 | hCoV-19/USA/VSP0359/2020 | OK245584 |
| VSP0393 | B.1 | 10/9/20 | hospitalized | 99.7 | 2796 | hCoV-19/USA/VSP0393/2020 | OK245585 |
| VSP0399 | B.1 | 10/12/20 | hospitalized | 99.1 | 1480 | hCoV-19/USA/VSP0399/2020 | OK245586 |
| VSP0415 | B.1.243 | 10/19/20 | hospitalized | 99.6 | 2599 | hCoV-19/USA/VSP0415/2020 | OK245587 |
| VSP0421 | B.1.311 | 10/21/20 | hospitalized | 99.5 | 4966 | hCoV-19/USA/VSP0421/2020 | OK245588 |
| VSP0424 | B.1.2 | 10/21/20 | hospitalized | 99.7 | 5440 | hCoV-19/USA/VSP0424/2020 | OK245589 |
| VSP0425 | B.1.243 | 10/23/20 | hospitalized | 99.5 | 1635 | hCoV-19/USA/VSP0425/2020 | OK245590 |
| VSP0429 | B.1.1.434 | 10/26/20 | hospitalized | 99.7 | 1404 | hCoV-19/USA/VSP0429/2020 | OK245591 |
| VSP0448 | B.1.1.317 | 11/4/20 | hospitalized | 99.6 | 6319 | hCoV-19/USA/VSP0448/2020 | OK245592 |
| VSP0460 | B.1.243 | 11/11/20 | hospitalized | 99.7 | 6801 | hCoV-19/USA/VSP0460/2020 | OK245593 |
| VSP0470 | B.1.591 | 11/13/20 | hospitalized | 99.7 | 5740 | hCoV-19/USA/VSP0470/2020 | OK245594 |
| VSP0479 | B.1.1.434 | 11/16/20 | hospitalized | 99.6 | 6537 | hCoV-19/USA/VSP0479/2020 | OK245595 |
| VSP0483 | B.1.243 | 11/18/20 | hospitalized | 99.7 | 5644 | hCoV-19/USA/VSP0483/2020 | OK245596 |
| VSP0491 | B.1.543 | 11/20/20 | hospitalized | 99.4 | 5847 | hCoV-19/USA/VSP0491/2020 | OK245597 |
| VSP0503 | B.1.243 | 12/2/20 | hospitalized | 99.7 | 6003 | hCoV-19/USA/VSP0503/2020 | OK245598 |
| VSP0505 | B.1.2 | 12/3/20 | hospitalized | 99.6 | 5947 | hCoV-19/USA/VSP0505/2020 | OK245599 |
| VSP0510 | B.1.234 | 12/4/20 | hospitalized | 99.6 | 8236 | hCoV-19/USA/VSP0510/2020 | OK245600 |
| VSP0511 | B.1.369 | 12/7/20 | hospitalized | 96 | 80 | hCoV-19/USA/VSP0511/2020 | OK245601 |
| VSP0514 | B.1.243 | 12/7/20 | hospitalized | 99.7 | 5998 | hCoV-19/USA/VSP0514/2020 | OK245602 |
| VSP0517 | B.1.243 | 12/9/20 | hospitalized | 99.7 | 6525 | hCoV-19/USA/VSP0517/2020 | OK245603 |
| VSP0550 | B.1.243 | 12/14/20 | hospitalized | 99.6 | 13979 | hCoV-19/USA/VSP0550/2020 | OK245613 |
| VSP0554 | B.1.311 | 12/14/20 | hospitalized | 99.7 | 5812 | hCoV-19/USA/VSP0554/2020 | OK245614 |
| VSP0560 | B.1.243 | 12/18/20 | hospitalized | 99.7 | 11400 | hCoV-19/USA/VSP0560/2020 | OK245615 |
| VSP0571 | B.1.2 | 1/3/21 | s drop | 99.8 | 14881 | hCoV-19/USA/VSP0571/2021 | OK245617 |
| VSP0576 | B.1.243 | 1/8/21 | hospitalized | 99.7 | 8390 | hCoV-19/USA/VSP0576/2021 | OK245618 |
| VSP0586 | B.1.240 | 1/11/21 | hospitalized | 99.7 | 1671 | hCoV-19/USA/VSP0586/2021 | OK245619 |
| VSP0588 | B.1.243 | 1/11/21 | hospitalized | 99.7 | 22053 | hCoV-19/USA/VSP0588/2021 | OK245620 |
| VSP0596 | B.1.2 | 1/15/21 | hospitalized | 99.7 | 7544 | hCoV-19/USA/VSP0596/2021 | OK245621 |
| VSP0600 | B.1.243 | 1/15/21 | hospitalized | 99.7 | 7386 | hCoV-19/USA/VSP0600/2021 | OK245622 |
| VSP0601 | B.1.2 | 1/15/21 | hospitalized | 99.7 | 8719 | hCoV-19/USA/VSP0601/2021 | OK245623 |
| VSP0603 | B.1.234 | 12/9/20 | asymptomatic | 99.7 | 4502 | hCoV-19/USA/VSP0603/2021 | OK245624 |
| VSP0604 | B.1.311 | 12/9/20 | asymptomatic | 99.7 | 5314 | hCoV-19/USA/VSP0604/2021 | OK245625 |
| VSP0605 | B.1.1.434 | 12/9/20 | asymptomatic | 99.7 | 1480 | hCoV-19/USA/VSP0605/2021 | OK245626 |

| | | | | | | | |
|---|---|---|---|---|---|---|---|
| VSP0606 | B.1.1.434 | 12/7/20 | asymptomatic | 99.7 | 3798 | hCoV-19/USA/VSP0606/2021 | OK245627 |
| VSP0607 | B.1.2 | 12/15/20 | asymptomatic | 99.7 | 4786 | hCoV-19/USA/VSP0607/2021 | OK245628 |
| VSP0608 | B.1.243 | 12/15/20 | asymptomatic | 99.7 | 5034 | hCoV-19/USA/VSP0608/2021 | OK245629 |
| VSP0609 | B.1.2 | 12/15/20 | asymptomatic | 99.7 | 4664 | hCoV-19/USA/VSP0609/2021 | OK245630 |
| VSP0611 | B.1.2 | 12/15/20 | asymptomatic | 99.7 | 4822 | hCoV-19/USA/VSP0611/2021 | OK245631 |
| VSP0612 | B.1.311 | 12/22/20 | asymptomatic | 98.9 | 678 | hCoV-19/USA/VSP0612/2021 | OK245632 |
| VSP0613 | B.1.243 | 12/29/20 | asymptomatic | 99.7 | 3295 | hCoV-19/USA/VSP0613/2021 | OK245633 |
| VSP0614 | B.1.2 | 12/29/20 | asymptomatic | 99.7 | 4656 | hCoV-19/USA/VSP0614/2021 | OK245634 |
| VSP0615 | B.1.243 | 1/4/21 | asymptomatic | 99.7 | 4204 | hCoV-19/USA/VSP0615/2021 | OK245635 |
| VSP0616 | B.1.243 | 1/4/21 | asymptomatic | 96.9 | 135 | hCoV-19/USA/VSP0616/2021 | OK245636 |
| VSP0617 | B.1.243 | 1/4/21 | asymptomatic | 99.7 | 5117 | hCoV-19/USA/VSP0617/2021 | OK245637 |
| VSP0619 | B.1.2 | 12/23/20 | hospitalized | 98.1 | 2519 | hCoV-19/USA/PA-VSP0619/2020 | OK245638 |
| VSP0622 | B.1.375 | 1/20/21 | s drop | 99.7 | 13155 | hCoV-19/USA/VSP0622/2021 | OK245639 |
| VSP0625 | B.1.1.7 | 1/22/21 | s drop | 99.1 | 11175 | hCoV-19/USA/VSP0625/2021 | OK245641 |
| VSP0654 | B.1.1.7 | 1/27/21 | hospitalized | 99.7 | 12051 | hCoV-19/USA/VSP0654/2020 | OK245656 |
| VSP0657 | B.1.311 | 1/27/21 | hospitalized | 99 | 376 | hCoV-19/USA/VSP0657/2020 | OK245657 |
| VSP0672 | B.1.243 | 3/1/21 | surveillance | 99.7 | 2436 | hCoV-19/USA/PA-VSP0672/2021 | OK245671 |
| VSP0673 | B.1.243 | 3/1/21 | surveillance | 99 | 162 | hCoV-19/USA/PA-VSP0673/2021 | OK245672 |
| VSP0674 | B.1.1.7 | 3/1/21 | surveillance | 99.1 | 2377 | hCoV-19/USA/PA-VSP0674/2021 | OK245673 |
| VSP0676 | R.1 | 3/1/21 | surveillance | 98.9 | 7268 | hCoV-19/USA/PA-VSP0676/2021 | OK245674 |
| VSP0677 | B.1.628 | 3/1/21 | surveillance | 98.8 | 2671 | hCoV-19/USA/PA-VSP0677/2021 | OK245675 |
| VSP0678 | B.1.2 | 3/1/21 | surveillance | 99.7 | 5666 | hCoV-19/USA/PA-VSP0678/2021 | OK245676 |
| VSP0679 | B.1.2 | 3/1/21 | surveillance | 99.7 | 7679 | hCoV-19/USA/PA-VSP0679/2021 | OK245677 |
| VSP0680 | B.1.637 | 3/1/21 | surveillance | 99.7 | 6952 | hCoV-19/USA/PA-VSP0680/2021 | OK245678 |
| VSP0681 | B.1.526 | 3/1/21 | surveillance | 99.7 | 8527 | hCoV-19/USA/PA-VSP0681/2021 | OK245679 |
| VSP0682 | B.1.2 | 3/1/21 | surveillance | 99.7 | 8384 | hCoV-19/USA/PA-VSP0682/2021 | OK245680 |
| VSP0683 | B.1.243 | 3/1/21 | surveillance | 96.5 | 201 | hCoV-19/USA/PA-VSP0683/2021 | OK245681 |
| VSP0684 | B.1.243 | 3/1/21 | surveillance | 99.7 | 9417 | hCoV-19/USA/PA-VSP0684/2021 | OK245682 |
| VSP0685 | B.1.575 | 3/1/21 | surveillance | 99.7 | 7995 | hCoV-19/USA/PA-VSP0685/2021 | OK245683 |
| VSP0695 | B.1.575 | 3/1/21 | surveillance | 99.7 | 5846 | hCoV-19/USA/PA-VSP0695/2021 | OK245684 |
| VSP0696 | B.1.637 | 3/1/21 | surveillance | 99.7 | 5060 | hCoV-19/USA/PA-VSP0696/2021 | OK245685 |
| VSP0697 | B.1.2 | 3/1/21 | surveillance | 99.7 | 3674 | hCoV-19/USA/PA-VSP0697/2021 | OK245686 |
| VSP0698 | B.1.243 | 3/1/21 | surveillance | 99.7 | 5793 | hCoV-19/USA/PA-VSP0698/2021 | OK245687 |
| VSP0707 | B.1.243 | 3/22/21 | surveillance | 99.7 | 5501 | hCoV-19/USA/PA-VSP0707/2021 | OK245692 |
| VSP0709 | B.1.2 | 3/22/21 | surveillance | 99.7 | 4990 | hCoV-19/USA/PA-VSP0709/2021 | OK245693 |
| VSP0711 | B.1.1.7 | 3/22/21 | surveillance | 99.7 | 5425 | hCoV-19/USA/PA-VSP0711/2021 | OK245694 |
| VSP0790 | B.1.1.7 | 1/29/21 | hospitalized | 99.7 | 953 | hCoV-19/USA/PA-VSP0790/2021 | OK245713 |
| VSP0794 | B.1.2 | 2/5/21 | hospitalized | 99.6 | 337 | hCoV-19/USA/PA-VSP0794/2021 | OK245714 |
| VSP0796 | B.1.2 | 2/5/21 | hospitalized | 99.7 | 1125 | hCoV-19/USA/PA-VSP0796/2021 | OK245715 |
| VSP0811 | B.1.1.7 | 2/11/21 | s drop | 99.7 | 13760 | hCoV-19/USA/VSP0811/2021 | OK245721 |
| VSP0812 | B.1.1.7 | 2/11/21 | s drop | 99.7 | 10880 | hCoV-19/USA/VSP0812/2021 | OK245722 |
| VSP0813 | B.1.1.7 | 2/11/21 | s drop | 99.7 | 10718 | hCoV-19/USA/VSP0813/2021 | OK245723 |
| VSP0814 | B.1.1.7 | 2/12/21 | s drop | 99 | 10785 | hCoV-19/USA/VSP0814/2021 | OK245724 |
| VSP0815 | B.1.1.7 | 2/12/21 | s drop | 99.7 | 9042 | hCoV-19/USA/VSP0815/2021 | OK245725 |
| VSP0832 | B.1.243 | 2/12/21 | hospitalized | 98.4 | 2230 | hCoV-19/USA/PA-VSP0832/2021 | |
| VSP0840 | B.1.1.434 | 2/15/21 | hospitalized | 99.6 | 580 | hCoV-19/USA/PA-VSP0840/2021 | OK245735 |
| VSP0859 | B.1.526 | 2/22/21 | hospitalized | 99.5 | 488 | hCoV-19/USA/PA-VSP0859/2021 | OK245739 |

| | | | | | | | |
|---|---|---|---|---|---|---|---|
| VSP0866 | B.1.480 | 2/22/21 | hospitalized | 97.3 | 641 | hCoV-19/USA/VSP0866/2021 | OK245745 |
| VSP0868 | B.1.1.7 | 2/22/21 | s drop | 99.7 | 13767 | hCoV-19/USA/VSP0868/2021 | OK245747 |
| VSP0869 | B.1.1.7 | 2/22/21 | s drop | 99.7 | 14937 | hCoV-19/USA/VSP0869/2021 | OK245748 |
| VSP0870 | B.1.1.7 | 2/22/21 | s drop | 99.7 | 15409 | hCoV-19/USA/VSP0870/2021 | OK245749 |
| VSP0871 | B.1.1.7 | 2/22/21 | s drop | 99.7 | 13761 | hCoV-19/USA/VSP0871/2021 | OK245750 |
| VSP0872 | B.1.1.7 | 2/22/21 | s drop | 99.7 | 16372 | hCoV-19/USA/VSP0872/2021 | OK245751 |
| VSP0873 | B.1.1.7 | 2/22/21 | s drop | 99.1 | 13141 | hCoV-19/USA/VSP0873/2021 | OK245752 |
| VSP0875 | B.1.637 | 2/24/21 | hospitalized | 99.7 | 765 | hCoV-19/USA/PA-VSP0875/2021 | OK245753 |
| VSP0879 | B.1.1.7 | 3/1/21 | s drop | 99.7 | 6725 | hCoV-19/USA/PA-VSP0879/2021 | OK245754 |
| VSP0880 | B.1.1.7 | 3/1/21 | s drop | 99.7 | 6484 | hCoV-19/USA/PA-VSP0880/2021 | OK245755 |
| VSP0881 | B.1.1.519 | 3/1/21 | accine breakthroug | 99.7 | 7493 | hCoV-19/USA/PA-VSP0881/2021 | OK245756 |
| VSP0882 | B.1.1.7 | 3/1/21 | s drop | 99.7 | 7511 | hCoV-19/USA/NJ-VSP0882/2021 | OK245757 |
| VSP0884 | B.1.1.7 | 3/1/21 | s drop | 99.7 | 6615 | hCoV-19/USA/PA-VSP0884/2021 | OK245758 |
| VSP0885 | B.1.1.7 | 3/1/21 | s drop | 99.7 | 7926 | hCoV-19/USA/PA-VSP0885/2021 | OK245759 |
| VSP0886 | B.1.1.7 | 3/1/21 | s drop | 99.7 | 7279 | hCoV-19/USA/PA-VSP0886/2021 | OK245760 |
| VSP0887 | B.1.1.7 | 3/1/21 | s drop | 99.7 | 4291 | hCoV-19/USA/PA-VSP0887/2021 | OK245761 |
| VSP0888 | B.1.1.7 | 3/1/21 | s drop | 99.7 | 7254 | hCoV-19/USA/PA-VSP0888/2021 | OK245762 |
| VSP0889 | B.1.526 | 3/1/21 | accine breakthroug | 99.7 | 7570 | hCoV-19/USA/NJ-VSP0889/2021 | OK245763 |
| VSP0890 | B.1.1.7 | 3/1/21 | accine breakthroug | 97.7 | 44 | hCoV-19/USA/PA-VSP0890/2021 | OK245764 |
| VSP0891 | B.1.1.7 | 3/1/21 | s drop | 99.7 | 6487 | hCoV-19/USA/PA-VSP0891/2021 | OK245765 |
| VSP0892 | B.1.1.7 | 3/1/21 | s drop | 99.7 | 7782 | hCoV-19/USA/PA-VSP0892/2021 | OK245766 |
| VSP0893 | B.1.1.7 | 3/1/21 | s drop | 99.7 | 6528 | hCoV-19/USA/NJ-VSP0893/2021 | OK245767 |
| VSP0894 | B.1.1.7 | 3/1/21 | s drop | 99.7 | 6624 | hCoV-19/USA/NJ-VSP0894/2021 | OK245768 |
| VSP0895 | B.1.1.7 | 3/1/21 | s drop | 99.7 | 6834 | hCoV-19/USA/DE-VSP0895/2021 | OK245769 |
| VSP0896 | B.1.1.7 | 3/1/21 | s drop | 99.7 | 6555 | hCoV-19/USA/PA-VSP0896/2021 | OK245770 |
| VSP0897 | B.1.1.7 | 3/1/21 | s drop | 99.7 | 7130 | hCoV-19/USA/PA-VSP0897/2021 | OK245771 |
| VSP0912 | B.1.526 | 3/5/21 | hospitalized | 99.7 | 1529 | hCoV-19/USA/PA-VSP0912/2021 | OK245776 |
| VSP0915 | B.1.311 | 3/5/21 | hospitalized | 99.7 | 1239 | hCoV-19/USA/PA-VSP0915/2021 | OK245777 |
| VSP0918 | B.1.243 | 3/8/21 | hospitalized | 99.7 | 1581 | hCoV-19/USA/PA-VSP0918/2021 | OK245778 |
| VSP0923 | B.1.311 | 2/26/21 | hospitalized | 99.7 | 1402 | hCoV-19/USA/PA-VSP0923/2021 | OK245779 |
| VSP0926 | B.1.1.519 | 2/26/21 | hospitalized | 99.7 | 1440 | hCoV-19/USA/PA-VSP0926/2021 | OK245780 |
| VSP0934 | B.1.234 | 3/8/21 | surveillance | 99.7 | 7838 | hCoV-19/USA/VSP0934/2021 | MW881990 |
| VSP0935 | B.1.1.7 | 3/8/21 | surveillance | 99.7 | 12075 | hCoV-19/USA/VSP0935/2021 | MW881991 |
| VSP0936 | B.1.2 | 3/8/21 | surveillance | 99.7 | 9415 | hCoV-19/USA/VSP0936/2021 | MW881992 |
| VSP0937 | B.1.110.3 | 3/8/21 | surveillance | 99.7 | 8914 | hCoV-19/USA/VSP0937/2021 | MW881993 |
| VSP0938 | B.1.2 | 3/8/21 | surveillance | 99.1 | 8775 | hCoV-19/USA/VSP0938/2021 | MW881994 |
| VSP0939 | B.1.596 | 3/8/21 | surveillance | 99.7 | 9304 | hCoV-19/USA/VSP0939/2021 | MW881995 |
| VSP0941 | B.1.637 | 3/8/21 | surveillance | 99.7 | 8605 | hCoV-19/USA/VSP0941/2021 | MW881996 |
| VSP0942 | B.1.1.7 | 3/8/21 | surveillance | 99.7 | 5652 | hCoV-19/USA/VSP0942/2021 | MW881997 |
| VSP0943 | B.1.243 | 3/8/21 | surveillance | 99.6 | 2929 | hCoV-19/USA/VSP0943/2021 | MW881998 |
| VSP0944 | B.1.243 | 3/8/21 | surveillance | 95.7 | 6330 | hCoV-19/USA/VSP0944/2021 | |
| VSP0950 | B.1.311 | 3/8/21 | surveillance | 99.7 | 7960 | hCoV-19/USA/VSP0950/2021 | MW881999 |
| VSP0951 | R.1 | 3/8/21 | surveillance | 99.7 | 7159 | hCoV-19/USA/VSP0951/2021 | MW882000 |
| VSP0952 | B.1.596 | 3/8/21 | surveillance | 99.7 | 9300 | hCoV-19/USA/VSP0952/2021 | MW882001 |
| VSP0953 | B.1.526 | 3/8/21 | surveillance | 99.7 | 8070 | hCoV-19/USA/VSP0953/2021 | MW882002 |
| VSP0954 | B.1.637 | 3/8/21 | surveillance | 99.7 | 8555 | hCoV-19/USA/VSP0954/2021 | MW882003 |
| VSP0956 | B.1.409 | 3/8/21 | surveillance | 96.4 | 9541 | hCoV-19/USA/VSP0956/2021 | |

| | | | | | | | |
|---|---|---|---|---|---|---|---|
| VSP0957 | B.1.575 | 3/8/21 | surveillance | 99.6 | 8620 | hCoV-19/USA/VSP0957/2021 | MW882004 |
| VSP0959 | B.1.1.519 | 3/8/21 | surveillance | 99.7 | 8973 | hCoV-19/USA/VSP0959/2021 | MW882005 |
| VSP0960 | B.1.433 | 3/8/21 | surveillance | 99.6 | 7002 | hCoV-19/USA/VSP0960/2021 | MW882006 |
| VSP0961 | B.1.234 | 3/8/21 | surveillance | 98.9 | 8209 | hCoV-19/USA/VSP0961/2021 | MW882007 |
| VSP0964 | B.1.575 | 3/8/21 | surveillance | 99.7 | 7995 | hCoV-19/USA/VSP0964/2021 | MW882008 |
| VSP0966 | R.1 | 3/8/21 | surveillance | 99.6 | 11400 | hCoV-19/USA/VSP0966/2021 | MW882009 |
| VSP0967 | B.1.526 | 3/8/21 | surveillance | 99.7 | 9455 | hCoV-19/USA/VSP0967/2021 | MW882010 |
| VSP0970 | B.1.243 | 3/8/21 | surveillance | 99.7 | 28014 | hCoV-19/USA/VSP0970/2021 | MW882011 |
| VSP0971 | B.1.526 | 3/8/21 | surveillance | 99.6 | 11272 | hCoV-19/USA/VSP0971/2021 | MW882012 |
| VSP0972 | B.1.409 | 3/8/21 | surveillance | 99.7 | 10106 | hCoV-19/USA/VSP0972/2021 | MW882013 |
| VSP0973 | B.1.596 | 3/8/21 | surveillance | 99.7 | 20243 | hCoV-19/USA/VSP0973/2021 | OK245781 |
| VSP0974 | B.1.526 | 3/8/21 | surveillance | 99.7 | 8910 | hCoV-19/USA/VSP0974/2021 | MW882014 |
| VSP0975 | B.1.526 | 3/8/21 | surveillance | 98.9 | 7494 | hCoV-19/USA/VSP0975/2021 | MW882015 |
| VSP0977 | B.1.234 | 3/8/21 | surveillance | 99.7 | 13621 | hCoV-19/USA/VSP0977/2021 | MW882016 |
| VSP0978 | B.1.526 | 3/8/21 | surveillance | 99.5 | 11418 | hCoV-19/USA/VSP0978/2021 | MW882017 |
| VSP0979 | B.1.243 | 3/8/21 | surveillance | 99.7 | 10735 | hCoV-19/USA/VSP0979/2021 | MW882018 |
| VSP0980 | B.1.2 | 3/8/21 | surveillance | 99.7 | 7451 | hCoV-19/USA/VSP0980/2021 | MW882019 |
| VSP0981 | B.1.2 | 3/8/21 | surveillance | 99.7 | 6689 | hCoV-19/USA/VSP0981/2021 | MW882020 |
| VSP0982 | B.1.637 | 3/8/21 | surveillance | 99.7 | 7333 | hCoV-19/USA/VSP0982/2021 | MW882021 |
| VSP0983 | B.1.243 | 3/8/21 | surveillance | 99.7 | 5643 | hCoV-19/USA/VSP0983/2021 | MW882022 |
| VSP0984 | B.1.243 | 3/8/21 | surveillance | 99.7 | 5930 | hCoV-19/USA/VSP0984/2021 | MW882023 |
| VSP0985 | B.1.1.434 | 3/8/21 | surveillance | 99.7 | 7871 | hCoV-19/USA/VSP0985/2021 | MW882024 |
| VSP0986 | B.1.526 | 3/8/21 | surveillance | 99.7 | 7071 | hCoV-19/USA/VSP0986/2021 | MW882025 |
| VSP0987 | B.1.2 | 3/8/21 | surveillance | 99.7 | 4616 | hCoV-19/USA/VSP0987/2021 | MW882026 |
| VSP0988 | B.1.2 | 3/8/21 | surveillance | 99.7 | 7081 | hCoV-19/USA/VSP0988/2021 | MW882027 |
| VSP0989 | B.1.1.7 | 3/8/21 | surveillance | 99.7 | 5906 | hCoV-19/USA/VSP0989/2021 | MW882028 |
| VSP0990 | B.1.1.7 | 3/8/21 | surveillance | 99.7 | 6581 | hCoV-19/USA/VSP0990/2021 | MW882029 |
| VSP0992 | B.1.526 | 3/8/21 | surveillance | 99.7 | 6525 | hCoV-19/USA/VSP0992/2021 | MW882030 |
| VSP0993 | B.1.526 | 3/8/21 | surveillance | 99.7 | 5136 | hCoV-19/USA/VSP0993/2021 | MW882031 |
| VSP0994 | B.1.526 | 3/8/21 | surveillance | 99.7 | 7398 | hCoV-19/USA/VSP0994/2021 | MW882032 |
| VSP0995 | B.1.637 | 3/8/21 | surveillance | 99.7 | 6177 | hCoV-19/USA/VSP0995/2021 | MW882033 |
| VSP0996 | B.1.306 | 3/8/21 | surveillance | 99.5 | 6285 | hCoV-19/USA/VSP0996/2021 | MW882034 |
| VSP0997 | B.1.637 | 3/8/21 | surveillance | 99.7 | 7365 | hCoV-19/USA/VSP0997/2021 | MW882035 |
| VSP0998 | B.1.234 | 3/8/21 | surveillance | 99.7 | 7635 | hCoV-19/USA/VSP0998/2021 | OK245782 |
| VSP0999 | R.1 | 3/8/21 | surveillance | 99.7 | 7978 | hCoV-19/USA/VSP0999/2021 | MW882036 |
| VSP1000 | B.1.575 | 3/8/21 | surveillance | 99.7 | 7050 | hCoV-19/USA/VSP1000/2021 | MW882037 |
| VSP1001 | B.1.2 | 3/8/21 | surveillance | 99.7 | 6802 | hCoV-19/USA/VSP1001/2021 | MW882038 |
| VSP1002 | B.1.575 | 3/8/21 | surveillance | 99.1 | 6671 | hCoV-19/USA/VSP1002/2021 | MW882039 |
| VSP1003 | B.1.526 | 3/8/21 | surveillance | 99.7 | 8415 | hCoV-19/USA/VSP1003/2021 | MW882040 |
| VSP1004 | B.1.243 | 3/8/21 | surveillance | 99.7 | 7955 | hCoV-19/USA/VSP1004/2021 | MW882041 |
| VSP1005 | B.1.526 | 3/8/21 | surveillance | 99.7 | 6994 | hCoV-19/USA/VSP1005/2021 | MW882042 |
| VSP1006 | B.1.595 | 3/8/21 | surveillance | 99.7 | 7433 | hCoV-19/USA/VSP1006/2021 | MW882043 |
| VSP1007 | B.1.1.434 | 3/8/21 | surveillance | 99.7 | 5998 | hCoV-19/USA/VSP1007/2021 | MW882044 |
| VSP1008 | R.1 | 3/8/21 | surveillance | 99.7 | 6809 | hCoV-19/USA/VSP1008/2021 | MW882045 |
| VSP1009 | B.1.575 | 3/8/21 | surveillance | 99.7 | 7231 | hCoV-19/USA/VSP1009/2021 | MW882046 |
| VSP1010 | B.1.526 | 3/8/21 | surveillance | 99.7 | 7234 | hCoV-19/USA/VSP1010/2021 | MW882047 |
| VSP1011 | B.1.409 | 3/8/21 | surveillance | 99.7 | 5339 | hCoV-19/USA/VSP1011/2021 | MW882048 |

| | | | | | | | |
|---|---|---|---|---|---|---|---|
| VSP1012 | B.1.1.7 | 3/8/21 | surveillance | 99.7 | 7106 | hCoV-19/USA/VSP1012/2021 | MW882049 |
| VSP1013 | B.1.2 | 3/8/21 | surveillance | 99.7 | 6488 | hCoV-19/USA/VSP1013/2021 | MW882050 |
| VSP1014 | B.1.110.3 | 3/8/21 | surveillance | 99.7 | 6592 | hCoV-19/USA/VSP1014/2021 | MW882051 |
| VSP1015 | B.1.170 | 3/8/21 | surveillance | 99.7 | 8843 | hCoV-19/USA/VSP1015/2021 | MW882052 |
| VSP1016 | B.1.2 | 3/8/21 | surveillance | 99.7 | 8536 | hCoV-19/USA/VSP1016/2021 | MW882053 |
| VSP1017 | B.1.2 | 3/8/21 | surveillance | 99.7 | 5550 | hCoV-19/USA/VSP1017/2021 | MW882054 |
| VSP1018 | B.1.433 | 3/8/21 | surveillance | 99.7 | 8305 | hCoV-19/USA/VSP1018/2021 | MW882055 |
| VSP1019 | B.1.526 | 3/8/21 | surveillance | 99.7 | 7763 | hCoV-19/USA/VSP1019/2021 | MW882056 |
| VSP1020 | B.1.429 | 3/8/21 | surveillance | 99.7 | 8050 | hCoV-19/USA/VSP1020/2021 | MW882057 |
| VSP1021 | B.1.2 | 3/8/21 | surveillance | 99.7 | 8227 | hCoV-19/USA/VSP1021/2021 | MW882058 |
| VSP1022 | B.1.1.7 | 3/8/21 | surveillance | 99.7 | 7311 | hCoV-19/USA/VSP1022/2021 | MW882059 |
| VSP1023 | B.1.427 | 3/8/21 | surveillance | 99.7 | 7749 | hCoV-19/USA/VSP1023/2021 | MW882060 |
| VSP1024 | B.1.2 | 3/8/21 | surveillance | 99.7 | 9755 | hCoV-19/USA/VSP1024/2021 | MW882061 |
| VSP1025 | B.1.1.7 | 3/8/21 | surveillance | 99.7 | 7888 | hCoV-19/USA/VSP1025/2021 | MW882062 |
| VSP1027 | B.1.1.519 | 3/8/21 | surveillance | 98.8 | 3176 | hCoV-19/USA/VSP1027/2021 | MW935669 |
| VSP1029 | B.1.1.7 | 3/8/21 | surveillance | 99.7 | 3248 | hCoV-19/USA/VSP1029/2021 | MW935670 |
| VSP1031 | B.1.1.7 | 3/8/21 | surveillance | 99.7 | 7549 | hCoV-19/USA/VSP1031/2021 | MW935671 |
| VSP1033 | B.1.1.7 | 3/8/21 | s drop | 95.1 | 2988 | hCoV-19/USA/PA-VSP1033/2021 | |
| VSP1034 | B.1.1.7 | 3/8/21 | s drop | 99.7 | 5231 | hCoV-19/USA/PA-VSP1034/2021 | OK245783 |
| VSP1035 | B.1.1.7 | 3/8/21 | s drop | 99.7 | 6428 | hCoV-19/USA/PA-VSP1035/2021 | OK245784 |
| VSP1036 | B.1.1.7 | 3/8/21 | s drop | 99.7 | 6819 | hCoV-19/USA/PA-VSP1036/2021 | OK245785 |
| VSP1044 | B.1.234 | 3/15/21 | hospitalized | 99.7 | 869 | hCoV-19/USA/PA-VSP1044/2021 | OK245787 |
| VSP1045 | B.1.311 | 3/17/21 | hospitalized | 99.6 | 2672 | hCoV-19/USA/PA-VSP1045/2021 | OK245788 |
| VSP1048 | B.1.311 | 3/17/21 | asymptomatic | 99.7 | 1442 | hCoV-19/USA/PA-VSP1048/2021 | OK245789 |
| VSP1050 | B.1.526 | 3/17/21 | asymptomatic | 99.7 | 3926 | hCoV-19/USA/PA-VSP1050/2021 | OK245790 |
| VSP1053 | B.1.2 | 3/17/21 | asymptomatic | 99.7 | 1771 | hCoV-19/USA/PA-VSP1053/2021 | OK245791 |
| VSP1058 | B.1.1.7 | 3/17/21 | asymptomatic | 99.7 | 5137 | hCoV-19/USA/PA-VSP1058/2021 | OK245792 |
| VSP1059 | B.1.396 | 3/17/21 | asymptomatic | 99.7 | 4519 | hCoV-19/USA/PA-VSP1059/2021 | OK245793 |
| VSP1060 | B.1.2 | 3/17/21 | asymptomatic | 99.7 | 4489 | hCoV-19/USA/PA-VSP1060/2021 | OK245794 |
| VSP1068 | B.1.1.7 | 3/15/21 | s drop | 99.7 | 5997 | hCoV-19/USA/PA-VSP1068/2021 | OK245795 |
| VSP1069 | B.1.1.7 | 3/15/21 | s drop | 99.7 | 5138 | hCoV-19/USA/PA-VSP1069/2021 | OK245796 |
| VSP1070 | B.1.1.7 | 3/15/21 | s drop | 99.7 | 995 | hCoV-19/USA/PA-VSP1070/2021 | OK245797 |
| VSP1072 | B.1.1.7 | 3/15/21 | accine breakthroug | 99.7 | 5149 | hCoV-19/USA/PA-VSP1072/2021 | OK245798 |
| VSP1073 | B.1.1.7 | 3/15/21 | s drop | 99.1 | 5430 | hCoV-19/USA/PA-VSP1073/2021 | OK245799 |
| VSP1074 | B.1.1.7 | 3/15/21 | s drop | 99.7 | 5258 | hCoV-19/USA/PA-VSP1074/2021 | OK245800 |
| VSP1075 | B.1.1.7 | 3/15/21 | s drop | 97 | 6252 | hCoV-19/USA/NJ-VSP1075/2021 | |
| VSP1076 | B.1.1.7 | 3/15/21 | s drop | 99.7 | 4127 | hCoV-19/USA/PA-VSP1076/2021 | OK245801 |
| VSP1078 | B.1.1.7 | 3/15/21 | s drop | 99.7 | 4493 | hCoV-19/USA/PA-VSP1078/2021 | OK245802 |
| VSP1080 | B.1.1.7 | 3/15/21 | s drop | 99.7 | 7527 | hCoV-19/USA/PA-VSP1080/2021 | OK245803 |
| VSP1081 | B.1.1.7 | 3/15/21 | s drop | 99.7 | 3837 | hCoV-19/USA/PA-VSP1081/2021 | OK245804 |
| VSP1083 | B.1.1.7 | 3/15/21 | accine breakthroug | 99.7 | 11797 | hCoV-19/USA/NJ-VSP1083/2021 | OK245805 |
| VSP1084 | B.1.1.7 | 3/15/21 | s drop | 99.4 | 3852 | hCoV-19/USA/PA-VSP1084/2021 | OK245806 |
| VSP1085 | B.1.1.7 | 3/15/21 | s drop | 99.4 | 3877 | hCoV-19/USA/PA-VSP1085/2021 | OK245807 |
| VSP1086 | B.1.596 | 3/15/21 | surveillance | 99.7 | 4943 | hCoV-19/USA/VSP1086/2021 | MW935672 |
| VSP1088 | B.1.1.519 | 3/15/21 | surveillance | 99.7 | 4171 | hCoV-19/USA/VSP1088/2021 | MW935673 |
| VSP1090 | B.1.575 | 3/15/21 | surveillance | 96.1 | 4413 | hCoV-19/USA/VSP1090/2021 | |
| VSP1092 | B.1.526 | 3/15/21 | surveillance | 99.7 | 3664 | hCoV-19/USA/VSP1092/2021 | MW935674 |

| | | | | | | | |
|---|---|---|---|---|---|---|---|
| VSP1093 | B.1.351 | 3/15/21 | surveillance | 98.7 | 4825 | hCoV-19/USA/VSP1093/2021 | OK245808 |
| VSP1094 | B.1.1.7 | 3/15/21 | surveillance | 99.7 | 3530 | hCoV-19/USA/VSP1094/2021 | MW935675 |
| VSP1095 | B.1.1.7 | 3/15/21 | surveillance | 99.7 | 5023 | hCoV-19/USA/VSP1095/2021 | MW935676 |
| VSP1096 | B.1.1.7 | 3/15/21 | surveillance | 98.3 | 3767 | hCoV-19/USA/VSP1096/2021 | MW935677 |
| VSP1097 | B.1.243 | 3/15/21 | surveillance | 99.7 | 4336 | hCoV-19/USA/VSP1097/2021 | MW935678 |
| VSP1099 | B.1.588 | 3/15/21 | surveillance | 99 | 5803 | hCoV-19/USA/VSP1099/2021 | MW935679 |
| VSP1100 | B.1.234 | 3/15/21 | surveillance | 99.7 | 4554 | hCoV-19/USA/VSP1100/2021 | MW935680 |
| VSP1101 | B.1.526 | 3/15/21 | surveillance | 99.7 | 6143 | hCoV-19/USA/VSP1101/2021 | MW935681 |
| VSP1102 | B.1.526 | 3/15/21 | surveillance | 99.7 | 4188 | hCoV-19/USA/VSP1102/2021 | MW935682 |
| VSP1103 | B.1.2 | 3/15/21 | surveillance | 99.7 | 3754 | hCoV-19/USA/VSP1103/2021 | MW935683 |
| VSP1104 | B.1.526 | 3/15/21 | surveillance | 99.7 | 4193 | hCoV-19/USA/VSP1104/2021 | MW935684 |
| VSP1105 | B.1.526 | 3/15/21 | surveillance | 99.7 | 464 | hCoV-19/USA/VSP1105/2021 | MW935685 |
| VSP1106 | B.1.637 | 3/15/21 | surveillance | 95.8 | 100 | hCoV-19/USA/VSP1106/2021 | MW935686 |
| VSP1107 | B.1.1.7 | 3/15/21 | surveillance | 95.6 | 27 | hCoV-19/USA/VSP1107/2021 | MW935687 |
| VSP1108 | B.1.526 | 3/15/21 | surveillance | 99.7 | 3405 | hCoV-19/USA/VSP1108/2021 | MW935688 |
| VSP1110 | B.1.1.7 | 3/15/21 | surveillance | 99.7 | 642 | hCoV-19/USA/VSP1110/2021 | MW935689 |
| VSP1111 | B.1.1.7 | 3/15/21 | surveillance | 99.7 | 2585 | hCoV-19/USA/VSP1111/2021 | MW935690 |
| VSP1112 | R.1 | 3/15/21 | surveillance | 98 | 229 | hCoV-19/USA/VSP1112/2021 | MW935691 |
| VSP1113 | B.1.110.3 | 3/15/21 | surveillance | 98.2 | 271 | hCoV-19/USA/VSP1113/2021 | MW935692 |
| VSP1114 | R.1 | 3/15/21 | surveillance | 99.7 | 4750 | hCoV-19/USA/VSP1114/2021 | MW935693 |
| VSP1115 | B.1.2 | 3/15/21 | surveillance | 99.7 | 5675 | hCoV-19/USA/VSP1115/2021 | MW935694 |
| VSP1116 | B.1.2 | 3/15/21 | surveillance | 99.7 | 3010 | hCoV-19/USA/VSP1116/2021 | MW935695 |
| VSP1117 | B.1.2 | 3/15/21 | surveillance | 99.7 | 4771 | hCoV-19/USA/VSP1117/2021 | MW935696 |
| VSP1118 | B.1.575 | 3/15/21 | surveillance | 98.2 | 1761 | hCoV-19/USA/VSP1118/2021 | MW935697 |
| VSP1119 | B.1.243 | 3/15/21 | surveillance | 99.7 | 7447 | hCoV-19/USA/VSP1119/2021 | MW935698 |
| VSP1120 | B.1.2 | 3/15/21 | surveillance | 99.7 | 7320 | hCoV-19/USA/VSP1120/2021 | MW935699 |
| VSP1121 | B.1.2 | 3/15/21 | surveillance | 99.7 | 8636 | hCoV-19/USA/VSP1121/2021 | MW935700 |
| VSP1122 | B.1.526 | 3/15/21 | surveillance | 99.7 | 6147 | hCoV-19/USA/VSP1122/2021 | MW935701 |
| VSP1123 | R.1 | 3/15/21 | surveillance | 99.7 | 6818 | hCoV-19/USA/VSP1123/2021 | MW935702 |
| VSP1124 | B.1.575 | 3/15/21 | surveillance | 99.7 | 6960 | hCoV-19/USA/VSP1124/2021 | MW935703 |
| VSP1126 | B.1.110.3 | 3/15/21 | surveillance | 99.7 | 7232 | hCoV-19/USA/VSP1126/2021 | MW935704 |
| VSP1127 | R.1 | 3/15/21 | surveillance | 99.7 | 6500 | hCoV-19/USA/VSP1127/2021 | MW935705 |
| VSP1128 | B.1.433 | 3/15/21 | surveillance | 99.7 | 5817 | hCoV-19/USA/VSP1128/2021 | MW935706 |
| VSP1129 | B.1.575 | 3/15/21 | surveillance | 99.7 | 6548 | hCoV-19/USA/VSP1129/2021 | MW935707 |
| VSP1130 | B.1.575 | 3/15/21 | surveillance | 99.7 | 8157 | hCoV-19/USA/VSP1130/2021 | OK245809 |
| VSP1131 | B.1.637 | 3/15/21 | surveillance | 99.7 | 7199 | hCoV-19/USA/VSP1131/2021 | MW935708 |
| VSP1132 | B.1.2 | 3/15/21 | surveillance | 99.7 | 8899 | hCoV-19/USA/VSP1132/2021 | MW935709 |
| VSP1133 | R.1 | 3/15/21 | surveillance | 99.7 | 5946 | hCoV-19/USA/VSP1133/2021 | MW935710 |
| VSP1134 | B.1.243 | 3/15/21 | surveillance | 99.7 | 5174 | hCoV-19/USA/VSP1134/2021 | MW935711 |
| VSP1135 | B.1.240 | 3/15/21 | surveillance | 99.7 | 8171 | hCoV-19/USA/VSP1135/2021 | MW935712 |
| VSP1136 | B.1.526 | 3/15/21 | surveillance | 99.7 | 7438 | hCoV-19/USA/VSP1136/2021 | MW935713 |
| VSP1137 | B.1.2 | 3/15/21 | surveillance | 99.7 | 7316 | hCoV-19/USA/VSP1137/2021 | MW935714 |
| VSP1138 | B.1.1.7 | 3/15/21 | accine breakthroug | 99.7 | 6398 | hCoV-19/USA/VSP1138/2021 | OK245810 |
| VSP1139 | B.1.1.7 | 3/15/21 | surveillance | 99.7 | 5940 | hCoV-19/USA/VSP1139/2021 | MW935715 |
| VSP1140 | R.1 | 3/15/21 | surveillance | 99.1 | 4612 | hCoV-19/USA/VSP1140/2021 | MW935716 |
| VSP1141 | B.1.575 | 3/15/21 | surveillance | 99.7 | 6592 | hCoV-19/USA/VSP1141/2021 | MW935717 |
| VSP1142 | B.1.2 | 3/15/21 | surveillance | 99.7 | 8844 | hCoV-19/USA/VSP1142/2021 | MW935718 |

| | | | | | | | |
|---|---|---|---|---|---|---|---|
| VSP1143 | B.1.2 | 3/15/21 | surveillance | 99.7 | 7059 | hCoV-19/USA/VSP1143/2021 | MW935719 |
| VSP1144 | B.1.2 | 3/15/21 | surveillance | 99.7 | 5322 | hCoV-19/USA/VSP1144/2021 | MW935720 |
| VSP1145 | B.1.575 | 3/15/21 | surveillance | 99.2 | 7279 | hCoV-19/USA/VSP1145/2021 | MW935721 |
| VSP1146 | R.1 | 3/15/21 | surveillance | 99.1 | 3967 | hCoV-19/USA/VSP1146/2021 | MW935722 |
| VSP1147 | B.1.311 | 3/15/21 | surveillance | 99.7 | 8133 | hCoV-19/USA/VSP1147/2021 | MW935723 |
| VSP1149 | B.1.243 | 3/15/21 | surveillance | 99.7 | 7596 | hCoV-19/USA/VSP1149/2021 | MW935724 |
| VSP1150 | B.1.637 | 3/15/21 | surveillance | 99.7 | 6363 | hCoV-19/USA/VSP1150/2021 | OK245811 |
| VSP1151 | B.1.2 | 3/15/21 | surveillance | 99.7 | 6088 | hCoV-19/USA/VSP1151/2021 | MW935725 |
| VSP1153 | R.1 | 3/15/21 | surveillance | 99.7 | 5946 | hCoV-19/USA/VSP1153/2021 | MW935726 |
| VSP1154 | B.1.1.7 | 3/15/21 | surveillance | 99.7 | 8266 | hCoV-19/USA/VSP1154/2021 | MW935727 |
| VSP1155 | B.1.2 | 3/15/21 | surveillance | 99.7 | 7425 | hCoV-19/USA/VSP1155/2021 | MW935728 |
| VSP1156 | B.1.2 | 3/15/21 | surveillance | 99.7 | 8822 | hCoV-19/USA/VSP1156/2021 | MW935729 |
| VSP1157 | B.1.2 | 3/15/21 | surveillance | 98.4 | 4086 | hCoV-19/USA/VSP1157/2021 | MW935730 |
| VSP1158 | B.1.2 | 3/15/21 | surveillance | 99.7 | 7266 | hCoV-19/USA/VSP1158/2021 | MW935731 |
| VSP1159 | B.1.2 | 3/15/21 | surveillance | 99.1 | 6003 | hCoV-19/USA/VSP1159/2021 | MW935732 |
| VSP1160 | B.1.2 | 3/15/21 | surveillance | 99.7 | 5299 | hCoV-19/USA/VSP1160/2021 | MW935733 |
| VSP1161 | R.1 | 3/15/21 | surveillance | 99.7 | 6677 | hCoV-19/USA/VSP1161/2021 | MW935734 |
| VSP1162 | B.1.1.7 | 3/15/21 | surveillance | 99.7 | 7591 | hCoV-19/USA/VSP1162/2021 | MW935735 |
| VSP1164 | B.1.1.7 | 3/15/21 | surveillance | 99.7 | 7664 | hCoV-19/USA/VSP1164/2021 | MW935736 |
| VSP1165 | B.1.2 | 3/15/21 | surveillance | 95.1 | 54 | hCoV-19/USA/PA-VSP1165/2021 | OK245812 |
| VSP1166 | B.1.1.7 | 3/15/21 | surveillance | 98.6 | 170 | hCoV-19/USA/PA-VSP1166/2021 | OK245813 |
| VSP1167 | B.1.637 | 3/15/21 | surveillance | 98.4 | 144 | hCoV-19/USA/PA-VSP1167/2021 | OK245814 |
| VSP1168 | B.1.2 | 3/15/21 | surveillance | 98.3 | 130 | hCoV-19/USA/PA-VSP1168/2021 | OK245815 |
| VSP1172 | B.1.311 | 3/15/21 | surveillance | 98.9 | 168 | hCoV-19/USA/PA-VSP1172/2021 | OK245816 |
| VSP1173 | B.1.1.7 | 3/15/21 | surveillance | 97.3 | 59 | hCoV-19/USA/PA-VSP1173/2021 | OK245817 |
| VSP1174 | B.1.243 | 3/15/21 | surveillance | 99 | 188 | hCoV-19/USA/PA-VSP1174/2021 | OK245818 |
| VSP1175 | R.1 | 3/15/21 | surveillance | 99.4 | 186 | hCoV-19/USA/PA-VSP1175/2021 | OK245819 |
| VSP1176 | B.1.1.519 | 3/15/21 | surveillance | 98.8 | 169 | hCoV-19/USA/PA-VSP1176/2021 | OK245820 |
| VSP1177 | B.1.526 | 3/15/21 | surveillance | 97.3 | 176 | hCoV-19/USA/PA-VSP1177/2021 | OK245821 |
| VSP1178 | B.1.526 | 3/15/21 | surveillance | 95.9 | 193 | hCoV-19/USA/PA-VSP1178/2021 | OK245822 |
| VSP1180 | B.1.2 | 3/15/21 | surveillance | 98.9 | 156 | hCoV-19/USA/NJ-VSP1180/2021 | OK245823 |
| VSP1181 | B.1.526 | 3/15/21 | surveillance | 99.3 | 164 | hCoV-19/USA/PA-VSP1181/2021 | OK245824 |
| VSP1182 | B.1.526 | 3/15/21 | surveillance | 97.8 | 133 | hCoV-19/USA/PA-VSP1182/2021 | OK245825 |
| VSP1184 | B.1.243 | 3/15/21 | surveillance | 97.7 | 180 | hCoV-19/USA/PA-VSP1184/2021 | OK245826 |
| VSP1186 | B.1.596 | 3/15/21 | surveillance | 99 | 176 | hCoV-19/USA/NJ-VSP1186/2021 | OK245827 |
| VSP1188 | B.1.526 | 3/15/21 | surveillance | 99 | 197 | hCoV-19/USA/PA-VSP1188/2021 | OK245828 |
| VSP1189 | B.1.588 | 3/15/21 | surveillance | 98.2 | 216 | hCoV-19/USA/PA-VSP1189/2021 | OK245829 |
| VSP1190 | B.1.2 | 3/15/21 | surveillance | 98.9 | 185 | hCoV-19/USA/PA-VSP1190/2021 | OK245830 |
| VSP1191 | B.1.243 | 3/15/21 | surveillance | 97.9 | 137 | hCoV-19/USA/PA-VSP1191/2021 | OK245831 |
| VSP1192 | B.1.243 | 3/15/21 | surveillance | 98.2 | 166 | hCoV-19/USA/PA-VSP1192/2021 | OK245832 |
| VSP1193 | B.1.575 | 3/15/21 | surveillance | 98.1 | 201 | hCoV-19/USA/PA-VSP1193/2021 | OK245833 |
| VSP1194 | B.1.526 | 3/15/21 | surveillance | 99.6 | 436 | hCoV-19/USA/PA-VSP1194/2021 | OK245834 |
| VSP1195 | B.1.575 | 3/15/21 | surveillance | 98.7 | 186 | hCoV-19/USA/PA-VSP1195/2021 | |
| VSP1196 | B.1.526 | 3/15/21 | surveillance | 96.5 | 222 | hCoV-19/USA/PA-VSP1196/2021 | OK245835 |
| VSP1197 | B.1.596 | 3/15/21 | surveillance | 98.7 | 183 | hCoV-19/USA/PA-VSP1197/2021 | OK245836 |
| VSP1198 | B.1.2 | 3/15/21 | surveillance | 99.2 | 176 | hCoV-19/USA/NJ-VSP1198/2021 | OK245837 |
| VSP1199 | B.1.361 | 3/15/21 | surveillance | 99 | 226 | hCoV-19/USA/PA-VSP1199/2021 | OK245838 |

| | | | | | | | |
|---|---|---|---|---|---|---|---|
| VSP1200 | B.1.1.348 | 3/15/21 | surveillance | 98.3 | 163 | hCoV-19/USA/PA-VSP1200/2021 | OK245839 |
| VSP1202 | B.1.526 | 3/15/21 | surveillance | 99.5 | 242 | hCoV-19/USA/PA-VSP1202/2021 | OK245840 |
| VSP1203 | B.1.526 | 3/15/21 | surveillance | 99.7 | 1069 | hCoV-19/USA/NJ-VSP1203/2021 | OK245841 |
| VSP1204 | B.1.234 | 3/15/21 | surveillance | 98.7 | 166 | hCoV-19/USA/PA-VSP1204/2021 | OK245842 |
| VSP1205 | B.1.1.192 | 3/15/21 | surveillance | 99.7 | 1638 | hCoV-19/USA/PA-VSP1205/2021 | OK245843 |
| VSP1207 | B.1.526 | 3/15/21 | surveillance | 99.7 | 292 | hCoV-19/USA/PA-VSP1207/2021 | OK245844 |
| VSP1212 | B.1.1.7 | 3/22/21 | hospitalized | 99.1 | 2009 | hCoV-19/USA/PA-VSP1212/2021 | OK245845 |
| VSP1217 | R.1 | 3/22/21 | accine breakthroug | 99.7 | 8860 | hCoV-19/USA/PA-VSP1217/2021 | OK245846 |
| VSP1218 | B.1.1.7 | 3/22/21 | s drop | 99.6 | 346 | hCoV-19/USA/NJ-VSP1218/2021 | OK245847 |
| VSP1220 | B.1.243 | 3/22/21 | accine breakthroug | 99.7 | 3222 | hCoV-19/USA/PA-VSP1220/2021 | OK245848 |
| VSP1221 | B.1.637 | 3/22/21 | accine breakthroug | 99.7 | 4107 | hCoV-19/USA/NJ-VSP1221/2021 | OK245849 |
| VSP1222 | B.1.1.7 | 3/22/21 | s drop | 99.7 | 4414 | hCoV-19/USA/PA-VSP1222/2021 | OK245850 |
| VSP1223 | B.1.1.7 | 3/22/21 | s drop | 99.7 | 897 | hCoV-19/USA/PA-VSP1223/2021 | OK245851 |
| VSP1224 | B.1.1.7 | 3/22/21 | s drop | 99.7 | 4065 | hCoV-19/USA/PA-VSP1224/2021 | OK245852 |
| VSP1225 | B.1.1.434 | 3/22/21 | s drop | 99 | 375 | hCoV-19/USA/PA-VSP1225/2021 | |
| VSP1226 | B.1.1.7 | 3/22/21 | s drop | 99.7 | 1858 | hCoV-19/USA/PA-VSP1226/2021 | OK245853 |
| VSP1227 | B.1.1.7 | 3/22/21 | s drop | 99.7 | 3360 | hCoV-19/USA/PA-VSP1227/2021 | OK245854 |
| VSP1228 | B.1.1.7 | 3/22/21 | s drop | 99.7 | 4800 | hCoV-19/USA/PA-VSP1228/2021 | OK245855 |
| VSP1229 | B.1.1.7 | 3/22/21 | s drop | 99.7 | 3156 | hCoV-19/USA/PA-VSP1229/2021 | OK245856 |
| VSP1230 | B.1.1.7 | 3/22/21 | s drop | 99.7 | 3427 | hCoV-19/USA/PA-VSP1230/2021 | OK245857 |
| VSP1231 | B.1.1.7 | 3/22/21 | s drop | 99.7 | 4394 | hCoV-19/USA/PA-VSP1231/2021 | OK245858 |
| VSP1232 | B.1.1.7 | 3/22/21 | s drop | 99.7 | 5754 | hCoV-19/USA/PA-VSP1232/2021 | OK245859 |
| VSP1233 | B.1.1.7 | 3/22/21 | s drop | 99.7 | 5852 | hCoV-19/USA/PA-VSP1233/2021 | OK245860 |
| VSP1236 | B.1.1.7 | 3/22/21 | s drop | 99.7 | 1716 | hCoV-19/USA/NJ-VSP1236/2021 | OK245861 |
| VSP1237 | B.1.1.7 | 3/22/21 | s drop | 99.7 | 664 | hCoV-19/USA/PA-VSP1237/2021 | OK245862 |
| VSP1238 | B.1.1.7 | 3/22/21 | s drop | 99.7 | 817 | hCoV-19/USA/PA-VSP1238/2021 | OK245863 |
| VSP1239 | B.1.234 | 3/22/21 | accine breakthroug | 99.7 | 1036 | hCoV-19/USA/PA-VSP1239/2021 | OK245864 |
| VSP1240 | B.1.1.7 | 3/22/21 | s drop | 99.7 | 5983 | hCoV-19/USA/PA-VSP1240/2021 | OK245865 |
| VSP1241 | B.1.1.7 | 3/22/21 | s drop | 99.7 | 2877 | hCoV-19/USA/PA-VSP1241/2021 | OK245866 |
| VSP1242 | B.1.1.7 | 3/22/21 | s drop | 99.7 | 4221 | hCoV-19/USA/PA-VSP1242/2021 | OK245867 |
| VSP1243 | B.1.1.7 | 3/22/21 | s drop | 99.7 | 5325 | hCoV-19/USA/PA-VSP1243/2021 | OK245868 |
| VSP1244 | B.1.1.7 | 3/22/21 | s drop | 99.7 | 4531 | hCoV-19/USA/NY-VSP1244/2021 | OK245869 |
| VSP1245 | B.1.1.7 | 3/22/21 | s drop | 99.7 | 3093 | hCoV-19/USA/PA-VSP1245/2021 | OK245870 |
| VSP1246 | B.1.1.7 | 3/22/21 | s drop | 99.7 | 1231 | hCoV-19/USA/PA-VSP1246/2021 | OK245871 |
| VSP1247 | B.1.1.7 | 3/22/21 | s drop | 99.7 | 4471 | hCoV-19/USA/PA-VSP1247/2021 | OK245872 |
| VSP1248 | B.1.1.7 | 3/22/21 | s drop | 99.7 | 5199 | hCoV-19/USA/PA-VSP1248/2021 | OK245873 |
| VSP1249 | B.1.1.7 | 3/22/21 | s drop | 99.7 | 2436 | hCoV-19/USA/PA-VSP1249/2021 | |
| VSP1250 | B.1.1.7 | 3/22/21 | s drop | 99.7 | 2495 | hCoV-19/USA/PA-VSP1250/2021 | OK245874 |
| VSP1251 | B.1.1.7 | 3/22/21 | s drop | 99.7 | 3774 | hCoV-19/USA/PA-VSP1251/2021 | OK245875 |
| VSP1252 | B.1.1.7 | 3/22/21 | s drop | 99.7 | 3445 | hCoV-19/USA/PA-VSP1252/2021 | OK245876 |
| VSP1253 | B.1.1.7 | 3/22/21 | s drop | 99.7 | 3011 | hCoV-19/USA/PA-VSP1253/2021 | OK245877 |
| VSP1254 | B.1.1.7 | 3/22/21 | s drop | 99.7 | 4854 | hCoV-19/USA/PA-VSP1254/2021 | OK245878 |
| VSP1255 | B.1.1.7 | 3/22/21 | s drop | 99.7 | 2804 | hCoV-19/USA/PA-VSP1255/2021 | OK245879 |
| VSP1256 | B.1.1.7 | 3/22/21 | s drop | 99.7 | 5849 | hCoV-19/USA/PA-VSP1256/2021 | OK245880 |
| VSP1257 | B.1.1.7 | 3/22/21 | s drop | 99.7 | 3615 | hCoV-19/USA/PA-VSP1257/2021 | OK245881 |
| VSP1258 | B.1.1.7 | 3/22/21 | s drop | 99.7 | 5094 | hCoV-19/USA/PA-VSP1258/2021 | OK245882 |
| VSP1259 | B.1.1.7 | 3/22/21 | s drop | 99.7 | 5721 | hCoV-19/USA/NJ-VSP1259/2021 | OK245883 |

| | | | | | | | |
|---|---|---|---|---|---|---|---|
| VSP1260 | B.1.1.7 | 3/22/21 | s drop | 99.7 | 4153 | hCoV-19/USA/PA-VSP1260/2021 | OK245884 |
| VSP1262 | B.1.1.7 | 3/22/21 | s drop | 99.7 | 1016 | hCoV-19/USA/PA-VSP1262/2021 | OK245885 |
| VSP1263 | B.1.1.7 | 3/22/21 | s drop | 99.7 | 1247 | hCoV-19/USA/NJ-VSP1263/2021 | OK245886 |
| VSP1267 | None | 3/22/21 | s drop | 97.4 | 804 | hCoV-19/USA/PA-VSP1267/2021 | |
| VSP1268 | B.1.1.7 | 3/22/21 | s drop | 99.7 | 4764 | hCoV-19/USA/PA-VSP1268/2021 | OK245887 |
| VSP1269 | B.1.1.7 | 3/22/21 | s drop | 99.7 | 4746 | hCoV-19/USA/PA-VSP1269/2021 | OK245888 |
| VSP1270 | B.1.1.7 | 3/22/21 | surveillance | 99.7 | 3794 | hCoV-19/USA/PA-VSP1270/2021 | OK245889 |
| VSP1271 | B.1.1.7 | 3/22/21 | surveillance | 99.7 | 4761 | hCoV-19/USA/PA-VSP1271/2021 | OK245890 |
| VSP1272 | B.1.526 | 3/22/21 | surveillance | 99.7 | 4227 | hCoV-19/USA/PA-VSP1272/2021 | OK245891 |
| VSP1273 | B.1.526 | 3/22/21 | surveillance | 99.7 | 5308 | hCoV-19/USA/PA-VSP1273/2021 | OK245892 |
| VSP1274 | B.1.575 | 3/22/21 | surveillance | 99.7 | 5590 | hCoV-19/USA/PA-VSP1274/2021 | OK245893 |
| VSP1275 | B.1.575 | 3/22/21 | surveillance | 99 | 258 | hCoV-19/USA/PA-VSP1275/2021 | OK245894 |
| VSP1276 | B.1.1.334 | 3/22/21 | surveillance | 99.7 | 2204 | hCoV-19/USA/PA-VSP1276/2021 | OK245895 |
| VSP1277 | B.1.1.519 | 3/22/21 | surveillance | 99.7 | 5276 | hCoV-19/USA/PA-VSP1277/2021 | OK245896 |
| VSP1279 | B.1.429 | 3/22/21 | surveillance | 99.7 | 3928 | hCoV-19/USA/GA-VSP1279/2021 | OK245897 |
| VSP1280 | B.1.2 | 3/22/21 | surveillance | 99.7 | 3925 | hCoV-19/USA/PA-VSP1280/2021 | OK245898 |
| VSP1281 | B.1.1.7 | 3/22/21 | surveillance | 99.7 | 3771 | hCoV-19/USA/PA-VSP1281/2021 | OK245899 |
| VSP1283 | B.1.1.7 | 3/22/21 | surveillance | 99.7 | 3765 | hCoV-19/USA/PA-VSP1283/2021 | OK245900 |
| VSP1284 | B.1.1.7 | 3/22/21 | surveillance | 99.7 | 4935 | hCoV-19/USA/PA-VSP1284/2021 | OK245901 |
| VSP1285 | B.1.1.7 | 3/22/21 | surveillance | 99.7 | 3643 | hCoV-19/USA/PA-VSP1285/2021 | OK245902 |
| VSP1286 | B.1.2 | 3/22/21 | surveillance | 99.7 | 2790 | hCoV-19/USA/PA-VSP1286/2021 | OK245903 |
| VSP1287 | B.1.526 | 3/22/21 | surveillance | 99.7 | 2501 | hCoV-19/USA/PA-VSP1287/2021 | OK245904 |
| VSP1288 | B.1.2 | 3/22/21 | surveillance | 99.7 | 5964 | hCoV-19/USA/PA-VSP1288/2021 | OK245905 |
| VSP1289 | B.1.243 | 3/22/21 | surveillance | 99.7 | 4793 | hCoV-19/USA/PA-VSP1289/2021 | OK245906 |
| VSP1290 | B.1.243 | 3/22/21 | surveillance | 99.7 | 6544 | hCoV-19/USA/PA-VSP1290/2021 | OK245907 |
| VSP1291 | B.1.526 | 3/22/21 | surveillance | 99.7 | 4139 | hCoV-19/USA/PA-VSP1291/2021 | OK245908 |
| VSP1292 | B.1.2 | 3/22/21 | surveillance | 99.7 | 3825 | hCoV-19/USA/PA-VSP1292/2021 | OK245909 |
| VSP1293 | B.1.1.7 | 3/22/21 | surveillance | 99.7 | 4358 | hCoV-19/USA/PA-VSP1293/2021 | OK245910 |
| VSP1294 | B.1.243 | 3/22/21 | surveillance | 99.7 | 3432 | hCoV-19/USA/PR-VSP1294/2021 | OK245911 |
| VSP1295 | B.1.1.7 | 3/22/21 | surveillance | 99.7 | 4988 | hCoV-19/USA/PA-VSP1295/2021 | OK245912 |
| VSP1297 | B.1.2 | 3/22/21 | surveillance | 99.7 | 3174 | hCoV-19/USA/PA-VSP1297/2021 | OK245913 |
| VSP1298 | B.1.1.265 | 3/22/21 | surveillance | 98.3 | 5608 | hCoV-19/USA/PA-VSP1298/2021 | OK245914 |
| VSP1299 | R.1 | 3/22/21 | surveillance | 99.6 | 4017 | hCoV-19/USA/PA-VSP1299/2021 | OK245915 |
| VSP1300 | B.1.1.231 | 3/22/21 | surveillance | 99.7 | 5384 | hCoV-19/USA/PA-VSP1300/2021 | OK245916 |
| VSP1301 | B.1.1.7 | 3/22/21 | surveillance | 99.7 | 7847 | hCoV-19/USA/PA-VSP1301/2021 | OK245917 |
| VSP1302 | B.1.2 | 3/22/21 | surveillance | 99.7 | 2855 | hCoV-19/USA/PA-VSP1302/2021 | OK245918 |
| VSP1303 | R.1 | 3/22/21 | surveillance | 99.7 | 1505 | hCoV-19/USA/PA-VSP1303/2021 | OK245919 |
| VSP1304 | B.1.2 | 3/22/21 | surveillance | 99.7 | 4856 | hCoV-19/USA/PA-VSP1304/2021 | OK245920 |
| VSP1306 | B.1.575 | 3/22/21 | surveillance | 99.7 | 6797 | hCoV-19/USA/PA-VSP1306/2021 | OK245921 |
| VSP1307 | B.1.110.3 | 3/22/21 | surveillance | 99.7 | 6233 | hCoV-19/USA/PA-VSP1307/2021 | OK245922 |
| VSP1308 | B.1.2 | 3/22/21 | surveillance | 99.7 | 7065 | hCoV-19/USA/PA-VSP1308/2021 | OK245923 |
| VSP1309 | B.1.243 | 3/22/21 | surveillance | 99.7 | 5990 | hCoV-19/USA/VSP1309/2021 | MW935737 |
| VSP1310 | B.1.2 | 3/22/21 | surveillance | 98.4 | 2272 | hCoV-19/USA/VSP1310/2021 | MW935738 |
| VSP1311 | B.1.2 | 3/22/21 | surveillance | 99.7 | 5440 | hCoV-19/USA/VSP1311/2021 | MW935739 |
| VSP1312 | R.1 | 3/22/21 | surveillance | 99.7 | 3733 | hCoV-19/USA/VSP1312/2021 | MW935740 |
| VSP1313 | R.1 | 3/22/21 | surveillance | 99.7 | 5949 | hCoV-19/USA/VSP1313/2021 | MW935741 |
| VSP1314 | B.1.110.3 | 3/22/21 | surveillance | 99.7 | 5028 | hCoV-19/USA/VSP1314/2021 | MW935742 |

| | | | | | | | |
|---|---|---|---|---|---|---|---|
| VSP1315 | B.1.311 | 3/22/21 | surveillance | 99.7 | 4550 | hCoV-19/USA/VSP1315/2021 | MW935743 |
| VSP1316 | B.1.526 | 3/22/21 | surveillance | 99.7 | 4039 | hCoV-19/USA/VSP1316/2021 | MW935744 |
| VSP1317 | B.1.2 | 3/22/21 | surveillance | 99.7 | 4280 | hCoV-19/USA/VSP1317/2021 | MW935745 |
| VSP1318 | B.1.2 | 3/22/21 | surveillance | 99.7 | 3494 | hCoV-19/USA/VSP1318/2021 | MW935746 |
| VSP1320 | B.1.2 | 3/22/21 | surveillance | 98.3 | 3144 | hCoV-19/USA/VSP1320/2021 | MW935747 |
| VSP1321 | B.1.2 | 3/22/21 | surveillance | 99.4 | 3896 | hCoV-19/USA/VSP1321/2021 | MW935748 |
| VSP1324 | B.1.526 | 3/22/21 | surveillance | 99.7 | 4229 | hCoV-19/USA/VSP1324/2021 | MW935749 |
| VSP1325 | B.1.526 | 3/22/21 | surveillance | 99.4 | 5083 | hCoV-19/USA/VSP1325/2021 | MW935750 |
| VSP1327 | B.1.2 | 3/22/21 | surveillance | 99.7 | 3359 | hCoV-19/USA/VSP1327/2021 | MW935751 |
| VSP1328 | B.1.2 | 3/22/21 | surveillance | 99.1 | 4561 | hCoV-19/USA/VSP1328/2021 | MW935752 |
| VSP1330 | B.1.2 | 3/22/21 | surveillance | 99.7 | 4926 | hCoV-19/USA/VSP1330/2021 | MW935753 |
| VSP1331 | B.1.2 | 3/22/21 | surveillance | 99.7 | 4968 | hCoV-19/USA/VSP1331/2021 | MW935754 |
| VSP1332 | B.1.2 | 3/22/21 | surveillance | 99.1 | 3097 | hCoV-19/USA/VSP1332/2021 | MW935755 |
| VSP1334 | B.1.1.7 | 3/22/21 | surveillance | 99.7 | 4665 | hCoV-19/USA/VSP1334/2021 | MW935756 |
| VSP1335 | B.1.2 | 3/22/21 | surveillance | 99.7 | 4305 | hCoV-19/USA/VSP1335/2021 | MW935757 |
| VSP1337 | B.1.1.7 | 3/22/21 | surveillance | 99.7 | 4358 | hCoV-19/USA/VSP1337/2021 | MW935758 |
| VSP1338 | B.1.1.7 | 3/22/21 | surveillance | 99.7 | 4526 | hCoV-19/USA/VSP1338/2021 | MW935759 |
| VSP1339 | B.1.1.7 | 3/22/21 | surveillance | 99.1 | 3180 | hCoV-19/USA/VSP1339/2021 | MW935760 |
| VSP1340 | B.1.1.7 | 3/22/21 | surveillance | 99.7 | 4947 | hCoV-19/USA/VSP1340/2021 | MW935761 |
| VSP1341 | B.1.637 | 3/22/21 | surveillance | 99.7 | 5606 | hCoV-19/USA/VSP1341/2021 | MW935762 |
| VSP1342 | B.1.575 | 3/22/21 | surveillance | 99.6 | 3496 | hCoV-19/USA/VSP1342/2021 | MW935763 |
| VSP1343 | B.1.243 | 3/22/21 | surveillance | 99.1 | 3898 | hCoV-19/USA/VSP1343/2021 | MW935764 |
| VSP1344 | B.1.2 | 3/22/21 | surveillance | 99.1 | 3841 | hCoV-19/USA/VSP1344/2021 | MW935765 |
| VSP1345 | B.1.2 | 3/22/21 | surveillance | 99.7 | 4075 | hCoV-19/USA/VSP1345/2021 | MW935766 |
| VSP1346 | B.1.1.7 | 3/22/21 | surveillance | 99.7 | 6344 | hCoV-19/USA/VSP1346/2021 | MW935767 |
| VSP1347 | B.1.525 | 3/22/21 | surveillance | 99.5 | 5388 | hCoV-19/USA/VSP1347/2021 | |
| VSP1348 | R.1 | 3/22/21 | surveillance | 99.1 | 2980 | hCoV-19/USA/VSP1348/2021 | MW935768 |
| VSP1350 | B.1.637 | 3/22/21 | surveillance | 98.8 | 2487 | hCoV-19/USA/VSP1350/2021 | MW935769 |
| VSP1351 | B.1.526 | 3/22/21 | surveillance | 99.7 | 2601 | hCoV-19/USA/VSP1351/2021 | MW935770 |
| VSP1352 | R.1 | 3/22/21 | surveillance | 99.1 | 2573 | hCoV-19/USA/VSP1352/2021 | MW935771 |
| VSP1353 | B.1.1.265 | 3/22/21 | surveillance | 96.7 | 2302 | hCoV-19/USA/VSP1353/2021 | MW935772 |
| VSP1354 | B.1.526 | 3/22/21 | surveillance | 99.7 | 3635 | hCoV-19/USA/VSP1354/2021 | MW935773 |
| VSP1356 | B.1.526 | 3/22/21 | surveillance | 99.7 | 3260 | hCoV-19/USA/VSP1356/2021 | MW935774 |
| VSP1357 | B.1.429 | 3/22/21 | surveillance | 99.7 | 3703 | hCoV-19/USA/VSP1357/2021 | MW935775 |
| VSP1358 | B.1.2 | 3/22/21 | surveillance | 99.7 | 3345 | hCoV-19/USA/VSP1358/2021 | MW935776 |
| VSP1359 | B.1.526 | 3/22/21 | surveillance | 99.7 | 3511 | hCoV-19/USA/VSP1359/2021 | MW935777 |
| VSP1360 | R.1 | 3/22/21 | surveillance | 99.7 | 3530 | hCoV-19/USA/VSP1360/2021 | MW935778 |
| VSP1361 | B.1.623 | 3/22/21 | surveillance | 99 | 2608 | hCoV-19/USA/VSP1361/2021 | MW935779 |
| VSP1362 | B.1.637 | 3/22/21 | surveillance | 99.7 | 4117 | hCoV-19/USA/VSP1362/2021 | MW935780 |
| VSP1363 | B.1.526 | 3/22/21 | surveillance | 99.7 | 3279 | hCoV-19/USA/VSP1363/2021 | MW935781 |
| VSP1364 | B.1.243 | 3/22/21 | surveillance | 99.7 | 3216 | hCoV-19/USA/VSP1364/2021 | MW935782 |
| VSP1365 | B.1.526 | 3/22/21 | surveillance | 99.7 | 3183 | hCoV-19/USA/VSP1365/2021 | MW935783 |
| VSP1366 | B.1.311 | 3/22/21 | surveillance | 99.7 | 4226 | hCoV-19/USA/VSP1366/2021 | OK245924 |
| VSP1367 | B.1.526 | 3/22/21 | surveillance | 99.7 | 4082 | hCoV-19/USA/VSP1367/2021 | MW935784 |
| VSP1368 | B.1.1.7 | 3/22/21 | surveillance | 99.7 | 3307 | hCoV-19/USA/VSP1368/2021 | MW935785 |
| VSP1369 | B.1.637 | 3/22/21 | surveillance | 99.6 | 2877 | hCoV-19/USA/VSP1369/2021 | MW935786 |
| VSP1370 | B.1.637 | 3/22/21 | surveillance | 99.7 | 3585 | hCoV-19/USA/VSP1370/2021 | MW935787 |

| | | | | | | | |
|---|---|---|---|---|---|---|---|
| VSP1371 | B.1.637 | 3/22/21 | surveillance | 99.7 | 3584 | hCoV-19/USA/VSP1371/2021 | MW935788 |
| VSP1372 | B.1.1.7 | 3/22/21 | surveillance | 99.7 | 3626 | hCoV-19/USA/VSP1372/2021 | OK245925 |
| VSP1373 | B.1.526 | 3/22/21 | surveillance | 99.7 | 3669 | hCoV-19/USA/VSP1373/2021 | MW935789 |
| VSP1374 | B.1.1.7 | 3/22/21 | surveillance | 99.7 | 3412 | hCoV-19/USA/VSP1374/2021 | MW935790 |
| VSP1375 | B.1.596 | 3/22/21 | surveillance | 99.7 | 3115 | hCoV-19/USA/VSP1375/2021 | MW935791 |
| VSP1376 | B.1.1.7 | 3/22/21 | surveillance | 99.7 | 2972 | hCoV-19/USA/VSP1376/2021 | MW935792 |
| VSP1377 | B.1.1.7 | 3/22/21 | surveillance | 99.7 | 3557 | hCoV-19/USA/VSP1377/2021 | MW935793 |
| VSP1378 | B.1.1.7 | 3/22/21 | surveillance | 99.7 | 2792 | hCoV-19/USA/VSP1378/2021 | MW935794 |
| VSP1379 | B.1.1.7 | 3/22/21 | surveillance | 99.7 | 3354 | hCoV-19/USA/VSP1379/2021 | MW935795 |
| VSP1380 | B.1.1.7 | 3/22/21 | surveillance | 99.7 | 1867 | hCoV-19/USA/VSP1380/2021 | MW935796 |
| VSP1381 | R.1 | 3/22/21 | surveillance | 97.6 | 182 | hCoV-19/USA/VSP1381/2021 | MW935797 |
| VSP1382 | B.1.1.7 | 3/22/21 | surveillance | 99.6 | 2488 | hCoV-19/USA/VSP1382/2021 | MW935798 |
| VSP1383 | B.1.2 | 3/22/21 | surveillance | 99.7 | 3512 | hCoV-19/USA/VSP1383/2021 | MW935799 |
| VSP1384 | B.1.596 | 3/22/21 | surveillance | 99.7 | 4059 | hCoV-19/USA/VSP1384/2021 | MW935800 |
| VSP1386 | B.1.526 | 3/22/21 | surveillance | 99.7 | 4042 | hCoV-19/USA/VSP1386/2021 | MW935801 |
| VSP1387 | P.1.10 | 3/22/21 | surveillance | 99.7 | 4144 | hCoV-19/USA/VSP1387/2021 | MW935802 |
| VSP1388 | B.1.2 | 3/22/21 | surveillance | 99.7 | 3750 | hCoV-19/USA/VSP1388/2021 | MW935803 |
| VSP1389 | R.1 | 3/22/21 | surveillance | 99.7 | 2745 | hCoV-19/USA/VSP1389/2021 | MW935804 |
| VSP1390 | B.1.526 | 3/22/21 | surveillance | 99.7 | 3824 | hCoV-19/USA/VSP1390/2021 | MW935805 |
| VSP1391 | B.1.1.7 | 3/22/21 | surveillance | 99.7 | 3754 | hCoV-19/USA/VSP1391/2021 | MW935806 |
| VSP1393 | B.1.2 | 3/22/21 | surveillance | 98.6 | 48 | hCoV-19/USA/VSP1393/2021 | MW935807 |
| VSP1398 | B.1.1.7 | 3/22/21 | surveillance | 99.4 | 279 | hCoV-19/USA/VSP1398/2021 | MW935809 |
| VSP1400 | B.1.234 | 3/22/21 | surveillance | 98.5 | 47 | hCoV-19/USA/VSP1400/2021 | MW935810 |
| VSP1402 | B.1.1.7 | 3/22/21 | surveillance | 97.3 | 32 | hCoV-19/USA/VSP1402/2021 | MW935811 |
| VSP1403 | B.1.2 | 3/22/21 | surveillance | 98.9 | 116 | hCoV-19/USA/VSP1403/2021 | MW935812 |
| VSP1405 | B.1.1.519 | 3/22/21 | surveillance | 96.1 | 27 | hCoV-19/USA/VSP1405/2021 | MW935813 |
| VSP1406 | B.1.526 | 3/22/21 | surveillance | 97.9 | 58 | hCoV-19/USA/VSP1406/2021 | MW935814 |
| VSP1408 | B.1.526 | 3/22/21 | accine breakthroug | 97.9 | 35 | hCoV-19/USA/VSP1408/2021 | MW935815 |
| VSP1411 | B.1 | 3/22/21 | surveillance | 95 | 22 | hCoV-19/USA/VSP1411/2021 | MW935816 |
| VSP1416 | B.1.1.519 | 3/22/21 | surveillance | 97.9 | 36 | hCoV-19/USA/VSP1416/2021 | MW935817 |
| VSP1421 | B.1.1.7 | 3/22/21 | surveillance | 95.5 | 24 | hCoV-19/USA/VSP1421/2021 | MW935818 |
| VSP1422 | B.1.1.434 | 3/22/21 | surveillance | 99 | 123 | hCoV-19/USA/VSP1422/2021 | MW935819 |
| VSP1423 | B.1.526 | 3/22/21 | surveillance | 98.8 | 66 | hCoV-19/USA/VSP1423/2021 | MW935820 |
| VSP1424 | B.1.526 | 3/22/21 | surveillance | 99.4 | 151 | hCoV-19/USA/VSP1424/2021 | MW935821 |
| VSP1426 | B.1.575 | 3/22/21 | surveillance | 96.8 | 39 | hCoV-19/USA/VSP1426/2021 | MW935822 |
| VSP1430 | B.1.2 | 3/22/21 | surveillance | 99.6 | 550 | hCoV-19/USA/VSP1430/2021 | MW935823 |
| VSP1433 | B.1.2 | 3/24/21 | accine breakthroug | 99.7 | 4858 | hCoV-19/USA/PA-VSP1433/2021 | MZ156590 |
| VSP1434 | B.1.2 | 3/26/21 | hospitalized | 99.7 | 1657 | hCoV-19/USA/PA-VSP1434/2021 | OK245926 |
| VSP1435 | B.1.2 | 3/26/21 | hospitalized | 99.6 | 850 | hCoV-19/USA/PA-VSP1435/2021 | OK245927 |
| VSP1439 | B.1.1.7 | 3/26/21 | hospitalized | 99.7 | 2672 | hCoV-19/USA/PA-VSP1439/2021 | OK245928 |
| VSP1447 | B.1.1.7 | 3/31/21 | s drop | 99 | 90 | hCoV-19/USA/NJ-VSP1447/2021 | MZ156591 |
| VSP1453 | B.1.1.7 | 3/31/21 | s drop | 99.7 | 4843 | hCoV-19/USA/PA-VSP1453/2021 | MZ156592 |
| VSP1454 | B.1.1.7 | 3/31/21 | s drop | 99.7 | 5032 | hCoV-19/USA/PA-VSP1454/2021 | MZ156593 |
| VSP1455 | B.1.1.7 | 3/31/21 | s drop | 99.7 | 4749 | hCoV-19/USA/PA-VSP1455/2021 | OK245929 |
| VSP1456 | B.1.1.7 | 3/31/21 | s drop | 99.7 | 6430 | hCoV-19/USA/PA-VSP1456/2021 | MZ156594 |
| VSP1458 | B.1.1.7 | 3/31/21 | s drop | 99.6 | 6730 | hCoV-19/USA/PA-VSP1458/2021 | OK245930 |
| VSP1459 | B.1.1.7 | 3/31/21 | s drop | 99.7 | 5158 | hCoV-19/USA/PA-VSP1459/2021 | MZ156595 |

| | | | | | | | |
|---|---|---|---|---|---|---|---|
| VSP1460 | B.1.525 | 3/31/21 | s drop | 99.7 | 8009 | hCoV-19/USA/NJ-VSP1460/2021 | |
| VSP1461 | B.1.1.7 | 3/31/21 | accine breakthroug | 99.7 | 722 | hCoV-19/USA/PA-VSP1461/2021 | MZ156596 |
| VSP1462 | B.1.1.7 | 3/31/21 | s drop | 99.7 | 5615 | hCoV-19/USA/PA-VSP1462/2021 | MZ156597 |
| VSP1463 | B.1.1.7 | 3/31/21 | s drop | 99.7 | 6295 | hCoV-19/USA/PA-VSP1463/2021 | MZ156598 |
| VSP1465 | B.1.1.7 | 3/31/21 | s drop | 99.7 | 4562 | hCoV-19/USA/PA-VSP1465/2021 | OK245931 |
| VSP1467 | B.1.1.7 | 3/31/21 | s drop | 99.3 | 4291 | hCoV-19/USA/PA-VSP1467/2021 | MZ156599 |
| VSP1468 | B.1.1.7 | 3/31/21 | s drop | 99.7 | 7311 | hCoV-19/USA/PA-VSP1468/2021 | MZ156600 |
| VSP1469 | B.1.1.7 | 3/31/21 | s drop | 99.7 | 7877 | hCoV-19/USA/PA-VSP1469/2021 | MZ156601 |
| VSP1470 | B.1.1.7 | 3/31/21 | s drop | 99.7 | 5540 | hCoV-19/USA/PA-VSP1470/2021 | MZ156602 |
| VSP1472 | B.1.1.7 | 3/31/21 | s drop | 99.7 | 7902 | hCoV-19/USA/PA-VSP1472/2021 | MZ156603 |
| VSP1473 | B.1.1.7 | 3/31/21 | s drop | 99.1 | 3322 | hCoV-19/USA/PA-VSP1473/2021 | MZ156604 |
| VSP1474 | B.1.1.7 | 3/31/21 | s drop | 99.7 | 7001 | hCoV-19/USA/PA-VSP1474/2021 | MZ156605 |
| VSP1475 | B.1.1.7 | 3/31/21 | s drop | 99 | 160 | hCoV-19/USA/PA-VSP1475/2021 | OK245932 |
| VSP1476 | B.1.1.7 | 3/31/21 | s drop | 99.7 | 3330 | hCoV-19/USA/PA-VSP1476/2021 | MZ156606 |
| VSP1477 | B.1.1.7 | 3/31/21 | s drop | 98.6 | 47 | hCoV-19/USA/PA-VSP1477/2021 | MZ156607 |
| VSP1478 | B.1.1.7 | 3/31/21 | s drop | 99.7 | 4048 | hCoV-19/USA/PA-VSP1478/2021 | MZ156608 |
| VSP1479 | B.1.1.7 | 3/31/21 | s drop | 99.7 | 2753 | hCoV-19/USA/DE-VSP1479/2021 | MZ156609 |
| VSP1480 | B.1.1.7 | 3/31/21 | s drop | 99.7 | 5151 | hCoV-19/USA/PA-VSP1480/2021 | MZ156610 |
| VSP1481 | B.1.525 | 3/31/21 | s drop | 99.5 | 8051 | hCoV-19/USA/NJ-VSP1481/2021 | |
| VSP1482 | B.1.1.7 | 3/31/21 | s drop | 99.7 | 8293 | hCoV-19/USA/PA-VSP1482/2021 | OK245933 |
| VSP1483 | B.1.1.7 | 3/31/21 | s drop | 98 | 47 | hCoV-19/USA/PA-VSP1483/2021 | MZ156611 |
| VSP1484 | B.1.1.7 | 3/31/21 | s drop | 99.7 | 7286 | hCoV-19/USA/PA-VSP1484/2021 | MZ156612 |
| VSP1485 | B.1.1.7 | 3/31/21 | s drop | 99.7 | 4137 | hCoV-19/USA/PA-VSP1485/2021 | MZ156613 |
| VSP1486 | B.1.1.7 | 3/31/21 | accine breakthroug | 99.7 | 7837 | hCoV-19/USA/PA-VSP1486/2021 | MZ156614 |
| VSP1487 | B.1.1.7 | 3/31/21 | s drop | 99.7 | 5635 | hCoV-19/USA/PA-VSP1487/2021 | MZ156615 |
| VSP1488 | B.1.1.7 | 3/31/21 | s drop | 99.7 | 6541 | hCoV-19/USA/PA-VSP1488/2021 | MZ156616 |
| VSP1489 | B.1.1.7 | 3/31/21 | s drop | 99.1 | 962 | hCoV-19/USA/PA-VSP1489/2021 | MZ156617 |
| VSP1490 | B.1.1.7 | 3/31/21 | s drop | 99.4 | 4388 | hCoV-19/USA/PA-VSP1490/2021 | MZ156618 |
| VSP1491 | B.1.1.7 | 3/31/21 | s drop | 99.7 | 8055 | hCoV-19/USA/PA-VSP1491/2021 | OK245934 |
| VSP1492 | B.1.1.7 | 3/31/21 | s drop | 99.7 | 4882 | hCoV-19/USA/PA-VSP1492/2021 | OK245935 |
| VSP1493 | B.1.1.7 | 3/31/21 | s drop | 99.7 | 8156 | hCoV-19/USA/PA-VSP1493/2021 | OK245936 |
| VSP1494 | B.1.1.7 | 3/31/21 | s drop | 99.7 | 7324 | hCoV-19/USA/PA-VSP1494/2021 | MZ156619 |
| VSP1495 | B.1.1.7 | 3/31/21 | s drop | 99.7 | 5401 | hCoV-19/USA/PA-VSP1495/2021 | MZ156620 |
| VSP1496 | B.1.1.7 | 3/31/21 | s drop | 99.7 | 3772 | hCoV-19/USA/PA-VSP1496/2021 | MZ156621 |
| VSP1498 | B.1.1.7 | 3/31/21 | s drop | 99.3 | 4247 | hCoV-19/USA/PA-VSP1498/2021 | OK245937 |
| VSP1499 | B.1.1.7 | 3/31/21 | s drop | 99.5 | 1984 | hCoV-19/USA/PA-VSP1499/2021 | OK245938 |
| VSP1500 | B.1.1.7 | 3/31/21 | s drop | 99.7 | 8771 | hCoV-19/USA/PA-VSP1500/2021 | MZ156622 |
| VSP1501 | B.1.1.7 | 3/31/21 | s drop | 99.7 | 4080 | hCoV-19/USA/PA-VSP1501/2021 | MZ156623 |
| VSP1502 | B.1.1.7 | 3/31/21 | s drop | 98 | 47 | hCoV-19/USA/PA-VSP1502/2021 | MZ156624 |
| VSP1503 | B.1.1.7 | 3/31/21 | s drop | 99.6 | 5616 | hCoV-19/USA/PA-VSP1503/2021 | OK245939 |
| VSP1505 | B.1.1.7 | 3/31/21 | s drop | 99.7 | 6529 | hCoV-19/USA/PA-VSP1505/2021 | MZ156625 |
| VSP1506 | B.1.1.7 | 3/31/21 | s drop | 99.6 | 6620 | hCoV-19/USA/PA-VSP1506/2021 | MZ156626 |
| VSP1507 | B.1.1.7 | 3/31/21 | s drop | 99.7 | 6198 | hCoV-19/USA/PA-VSP1507/2021 | MZ156627 |
| VSP1508 | B.1.1.7 | 3/31/21 | s drop | 99.7 | 8000 | hCoV-19/USA/PA-VSP1508/2021 | MZ156628 |
| VSP1509 | B.1.1.7 | 3/31/21 | s drop | 98.9 | 198 | hCoV-19/USA/PA-VSP1509/2021 | MZ156629 |
| VSP1514 | B.1.637 | 3/31/21 | surveillance | 99.7 | 3808 | hCoV-19/USA/NJ-VSP1514/2021 | MZ156633 |
| VSP1515 | B.1.2 | 3/31/21 | surveillance | 99.7 | 6187 | hCoV-19/USA/PA-VSP1515/2021 | MZ156634 |

| | | | | | | | |
|---|---|---|---|---|---|---|---|
| VSP1517 | B.1.637 | 3/31/21 | surveillance | 99 | 2141 | hCoV-19/USA/NJ-VSP1517/2021 | MZ156635 |
| VSP1518 | None | 3/31/21 | surveillance | 99 | 734 | hCoV-19/USA/NJ-VSP1518/2021 | MZ156636 |
| VSP1519 | R.1 | 3/31/21 | surveillance | 97.8 | 1672 | hCoV-19/USA/NJ-VSP1519/2021 | MZ156637 |
| VSP1520 | B.1.2 | 3/31/21 | surveillance | 99.1 | 5254 | hCoV-19/USA/PA-VSP1520/2021 | MZ156638 |
| VSP1521 | B.1.243 | 3/31/21 | surveillance | 99.7 | 7957 | hCoV-19/USA/PA-VSP1521/2021 | MZ156639 |
| VSP1522 | B.1.1.7 | 3/31/21 | surveillance | 99.7 | 4762 | hCoV-19/USA/PA-VSP1522/2021 | MZ156640 |
| VSP1523 | B.1.526 | 3/31/21 | surveillance | 99.7 | 5225 | hCoV-19/USA/PA-VSP1523/2021 | MZ156641 |
| VSP1524 | B.1.526 | 3/31/21 | surveillance | 99.5 | 4412 | hCoV-19/USA/PA-VSP1524/2021 | MZ156642 |
| VSP1526 | B.1.1.7 | 3/31/21 | surveillance | 99.1 | 3901 | hCoV-19/USA/PA-VSP1526/2021 | MZ156643 |
| VSP1527 | B.1.1.7 | 3/31/21 | surveillance | 99.2 | 4034 | hCoV-19/USA/PA-VSP1527/2021 | MZ156644 |
| VSP1528 | B.1.1.7 | 3/31/21 | surveillance | 99.7 | 8556 | hCoV-19/USA/PA-VSP1528/2021 | MZ156645 |
| VSP1529 | B.1.1.519 | 3/31/21 | surveillance | 99.6 | 8181 | hCoV-19/USA/PA-VSP1529/2021 | MZ156646 |
| VSP1530 | B.1.637 | 3/31/21 | surveillance | 99.5 | 4826 | hCoV-19/USA/PA-VSP1530/2021 | MZ156647 |
| VSP1531 | B.1.1.7 | 3/31/21 | surveillance | 99.3 | 4140 | hCoV-19/USA/PA-VSP1531/2021 | MZ156648 |
| VSP1532 | B.1.1.519 | 3/31/21 | surveillance | 99.7 | 4855 | hCoV-19/USA/PA-VSP1532/2021 | MZ156649 |
| VSP1533 | B.1.1.7 | 3/31/21 | surveillance | 98.9 | 1591 | hCoV-19/USA/PA-VSP1533/2021 | MZ156650 |
| VSP1534 | B.1.1.7 | 3/31/21 | surveillance | 99.7 | 8903 | hCoV-19/USA/PA-VSP1534/2021 | MZ156651 |
| VSP1536 | B.1.1.7 | 3/31/21 | surveillance | 99.1 | 2572 | hCoV-19/USA/PA-VSP1536/2021 | MZ156652 |
| VSP1537 | B.1.1.7 | 3/31/21 | surveillance | 99.3 | 4850 | hCoV-19/USA/PA-VSP1537/2021 | MZ156653 |
| VSP1538 | B.1.1.7 | 3/31/21 | surveillance | 99.3 | 1848 | hCoV-19/USA/PA-VSP1538/2021 | MZ156654 |
| VSP1539 | B.1.575 | 3/31/21 | surveillance | 99.4 | 6325 | hCoV-19/USA/PA-VSP1539/2021 | MZ156655 |
| VSP1541 | B.1.526 | 3/31/21 | surveillance | 99.1 | 319 | hCoV-19/USA/PA-VSP1541/2021 | MZ156656 |
| VSP1542 | B.1.637 | 3/31/21 | surveillance | 99.1 | 2225 | hCoV-19/USA/NJ-VSP1542/2021 | MZ156657 |
| VSP1543 | B.1.243 | 3/31/21 | surveillance | 99.7 | 6054 | hCoV-19/USA/VSP1543/2021 | MZ512576 |
| VSP1544 | B.1.526 | 3/31/21 | surveillance | 99.7 | 5876 | hCoV-19/USA/VSP1544/2021 | MZ512577 |
| VSP1545 | B.1.2 | 3/31/21 | surveillance | 99.7 | 5127 | hCoV-19/USA/VSP1545/2021 | MZ512578 |
| VSP1546 | B.1.427 | 3/31/21 | surveillance | 98.4 | 5611 | hCoV-19/USA/VSP1546/2021 | MZ512579 |
| VSP1547 | B.1.526 | 3/31/21 | surveillance | 99.5 | 5184 | hCoV-19/USA/VSP1547/2021 | MZ512580 |
| VSP1548 | B.1.1.7 | 3/31/21 | surveillance | 98.6 | 5560 | hCoV-19/USA/VSP1548/2021 | MZ512581 |
| VSP1549 | B.1.2 | 3/31/21 | surveillance | 99.7 | 4718 | hCoV-19/USA/VSP1549/2021 | MZ512582 |
| VSP1550 | B.1.2 | 3/31/21 | surveillance | 99.7 | 3517 | hCoV-19/USA/VSP1550/2021 | MZ512583 |
| VSP1551 | R.1 | 3/31/21 | surveillance | 99.7 | 3633 | hCoV-19/USA/VSP1551/2021 | MZ512584 |
| VSP1552 | B.1.1.7 | 3/31/21 | surveillance | 99.7 | 5530 | hCoV-19/USA/VSP1552/2021 | MZ512585 |
| VSP1553 | R.1 | 3/31/21 | surveillance | 99.7 | 4381 | hCoV-19/USA/VSP1553/2021 | MZ512586 |
| VSP1554 | B.1.243 | 3/31/21 | surveillance | 99.7 | 3694 | hCoV-19/USA/VSP1554/2021 | MZ512587 |
| VSP1555 | B.1.2 | 3/31/21 | surveillance | 99.7 | 4788 | hCoV-19/USA/VSP1555/2021 | OK245940 |
| VSP1556 | B.1.2 | 3/31/21 | surveillance | 99.7 | 4779 | hCoV-19/USA/VSP1556/2021 | MZ512588 |
| VSP1557 | B.1.1.7 | 3/31/21 | surveillance | 99.7 | 4538 | hCoV-19/USA/VSP1557/2021 | MZ512589 |
| VSP1558 | R.1 | 3/31/21 | surveillance | 98.9 | 4797 | hCoV-19/USA/VSP1558/2021 | MZ512590 |
| VSP1559 | B.1.1.7 | 3/31/21 | surveillance | 99.7 | 6275 | hCoV-19/USA/VSP1559/2021 | MZ512591 |
| VSP1560 | B.1.1 | 3/31/21 | surveillance | 99.7 | 5551 | hCoV-19/USA/VSP1560/2021 | MZ512592 |
| VSP1561 | B.1.243 | 3/31/21 | surveillance | 98.3 | 4720 | hCoV-19/USA/VSP1561/2021 | MZ512593 |
| VSP1562 | B.1.1.7 | 3/31/21 | surveillance | 99.7 | 4209 | hCoV-19/USA/VSP1562/2021 | MZ512594 |
| VSP1563 | R.1 | 3/31/21 | surveillance | 98.9 | 4622 | hCoV-19/USA/VSP1563/2021 | MZ512595 |
| VSP1564 | B.1.110.3 | 3/31/21 | surveillance | 99.7 | 4814 | hCoV-19/USA/VSP1564/2021 | MZ512596 |
| VSP1565 | B.1.526 | 3/31/21 | surveillance | 99.7 | 4539 | hCoV-19/USA/VSP1565/2021 | MZ512597 |
| VSP1566 | B.1.311 | 3/31/21 | surveillance | 99.7 | 4469 | hCoV-19/USA/VSP1566/2021 | MZ512598 |

| | | | | | | | |
|---|---|---|---|---|---|---|---|
| VSP1567 | B.1.1.7 | 3/31/21 | surveillance | 99.7 | 4889 | hCoV-19/USA/VSP1567/2021 | MZ512599 |
| VSP1568 | B.1.1.7 | 3/31/21 | surveillance | 99.7 | 4545 | hCoV-19/USA/VSP1568/2021 | MZ512600 |
| VSP1569 | B.1.1.7 | 3/31/21 | surveillance | 99.7 | 4289 | hCoV-19/USA/VSP1569/2021 | MZ512601 |
| VSP1570 | B.1.110.3 | 3/31/21 | surveillance | 99.7 | 5420 | hCoV-19/USA/VSP1570/2021 | MZ512602 |
| VSP1571 | B.1.1.7 | 3/31/21 | surveillance | 99.7 | 4422 | hCoV-19/USA/VSP1571/2021 | MZ512603 |
| VSP1572 | B.1.110.3 | 3/31/21 | surveillance | 99.7 | 4791 | hCoV-19/USA/VSP1572/2021 | MZ512604 |
| VSP1573 | B.1.1.7 | 3/31/21 | surveillance | 99.7 | 4524 | hCoV-19/USA/VSP1573/2021 | MZ512605 |
| VSP1574 | B.1.2 | 3/31/21 | surveillance | 99.7 | 3435 | hCoV-19/USA/VSP1574/2021 | MZ512606 |
| VSP1575 | R.1 | 3/31/21 | surveillance | 98.9 | 5460 | hCoV-19/USA/VSP1575/2021 | MZ512607 |
| VSP1576 | B.1.526 | 3/31/21 | surveillance | 99.7 | 4007 | hCoV-19/USA/VSP1576/2021 | MZ512608 |
| VSP1577 | B.1.2 | 3/31/21 | surveillance | 99.7 | 3027 | hCoV-19/USA/VSP1577/2021 | MZ512609 |
| VSP1578 | B.1.311 | 3/31/21 | surveillance | 99.7 | 1311 | hCoV-19/USA/VSP1578/2021 | MZ512610 |
| VSP1579 | B.1.2 | 3/31/21 | surveillance | 99.7 | 3520 | hCoV-19/USA/VSP1579/2021 | MZ512611 |
| VSP1580 | B.1.1.7 | 3/31/21 | surveillance | 99.7 | 3486 | hCoV-19/USA/VSP1580/2021 | MZ512612 |
| VSP1581 | B.1.637 | 3/31/21 | surveillance | 99.3 | 2002 | hCoV-19/USA/VSP1581/2021 | MZ512613 |
| VSP1582 | B.1.1.519 | 3/31/21 | surveillance | 99.7 | 4939 | hCoV-19/USA/VSP1582/2021 | MZ512614 |
| VSP1583 | B.1.1.7 | 3/31/21 | surveillance | 99.7 | 5156 | hCoV-19/USA/VSP1583/2021 | MZ512615 |
| VSP1584 | B.1.2 | 3/31/21 | surveillance | 99.7 | 5498 | hCoV-19/USA/VSP1584/2021 | MZ512616 |
| VSP1585 | B.1.1.7 | 3/31/21 | surveillance | 99.7 | 4611 | hCoV-19/USA/VSP1585/2021 | MZ512617 |
| VSP1586 | B.1 | 3/31/21 | surveillance | 95.7 | 389 | hCoV-19/USA/VSP1586/2021 | MZ512618 |
| VSP1587 | R.1 | 3/31/21 | surveillance | 98.9 | 3618 | hCoV-19/USA/VSP1587/2021 | MZ512619 |
| VSP1588 | B.1.2 | 3/31/21 | surveillance | 99.7 | 5652 | hCoV-19/USA/VSP1588/2021 | MZ512620 |
| VSP1589 | B.1.2 | 3/31/21 | surveillance | 99.7 | 5249 | hCoV-19/USA/VSP1589/2021 | MZ512621 |
| VSP1590 | B.1.1.7 | 3/31/21 | surveillance | 99.7 | 4834 | hCoV-19/USA/VSP1590/2021 | MZ512622 |
| VSP1591 | B.1.1.7 | 3/31/21 | surveillance | 99.7 | 5256 | hCoV-19/USA/VSP1591/2021 | MZ512623 |
| VSP1592 | B.1.2 | 3/31/21 | surveillance | 99.7 | 4861 | hCoV-19/USA/VSP1592/2021 | MZ512624 |
| VSP1594 | P.1 | 3/31/21 | surveillance | 99.7 | 5406 | hCoV-19/USA/VSP1594/2021 | MZ512625 |
| VSP1595 | B.1.575 | 3/31/21 | surveillance | 99.7 | 5614 | hCoV-19/USA/VSP1595/2021 | MZ512626 |
| VSP1596 | B.1.1.7 | 3/31/21 | surveillance | 99.7 | 5705 | hCoV-19/USA/VSP1596/2021 | MZ512627 |
| VSP1597 | B.1.575 | 3/31/21 | surveillance | 99.5 | 3699 | hCoV-19/USA/VSP1597/2021 | MZ512628 |
| VSP1598 | B.1.243 | 3/31/21 | surveillance | 99.6 | 3865 | hCoV-19/USA/VSP1598/2021 | MZ512629 |
| VSP1599 | B.1.2 | 3/31/21 | surveillance | 99.7 | 5309 | hCoV-19/USA/VSP1599/2021 | MZ512630 |
| VSP1600 | B.1.1.7 | 3/31/21 | surveillance | 99.7 | 4583 | hCoV-19/USA/VSP1600/2021 | MZ512631 |
| VSP1601 | B.1.2 | 3/31/21 | surveillance | 99.7 | 4550 | hCoV-19/USA/VSP1601/2021 | MZ512632 |
| VSP1602 | B.1.1.7 | 3/31/21 | surveillance | 99.7 | 4400 | hCoV-19/USA/VSP1602/2021 | MZ512633 |
| VSP1603 | B.1.1.7 | 3/31/21 | surveillance | 99.3 | 5007 | hCoV-19/USA/VSP1603/2021 | MZ512634 |
| VSP1604 | B.1.526 | 3/31/21 | surveillance | 99.4 | 1634 | hCoV-19/USA/VSP1604/2021 | MZ512635 |
| VSP1605 | B.1.1.7 | 3/31/21 | surveillance | 99.7 | 6208 | hCoV-19/USA/VSP1605/2021 | MZ512636 |
| VSP1606 | B.1.1.7 | 3/31/21 | surveillance | 99.2 | 6614 | hCoV-19/USA/VSP1606/2021 | MZ512637 |
| VSP1607 | B.1.2 | 3/31/21 | surveillance | 99.7 | 5535 | hCoV-19/USA/VSP1607/2021 | MZ512638 |
| VSP1608 | B.1.575 | 3/31/21 | surveillance | 99.5 | 7714 | hCoV-19/USA/VSP1608/2021 | MZ512639 |
| VSP1609 | B.1.637 | 3/31/21 | surveillance | 99.7 | 2472 | hCoV-19/USA/VSP1609/2021 | MZ512640 |
| VSP1610 | B.1.1.519 | 3/31/21 | surveillance | 99.7 | 6531 | hCoV-19/USA/VSP1610/2021 | MZ512641 |
| VSP1611 | B.1.575 | 3/31/21 | surveillance | 99.4 | 3801 | hCoV-19/USA/VSP1611/2021 | |
| VSP1612 | B.1.1.7 | 3/31/21 | surveillance | 99.7 | 6004 | hCoV-19/USA/VSP1612/2021 | MZ512642 |
| VSP1613 | B.1.243 | 3/31/21 | surveillance | 99.7 | 6381 | hCoV-19/USA/VSP1613/2021 | MZ512643 |
| VSP1614 | B.1.575 | 3/31/21 | surveillance | 99.4 | 5877 | hCoV-19/USA/VSP1614/2021 | MZ512644 |

| | | | | | | | |
|---|---|---|---|---|---|---|---|
| VSP1615 | B.1.1.7 | 3/31/21 | surveillance | 99.7 | 5165 | hCoV-19/USA/VSP1615/2021 | MZ512645 |
| VSP1616 | B.1.526 | 3/31/21 | surveillance | 99 | 363 | hCoV-19/USA/VSP1616/2021 | MZ512646 |
| VSP1617 | B.1.526 | 3/31/21 | surveillance | 99.7 | 6009 | hCoV-19/USA/VSP1617/2021 | MZ512647 |
| VSP1618 | B.1.526 | 3/31/21 | surveillance | 98.8 | 5407 | hCoV-19/USA/VSP1618/2021 | MZ512648 |
| VSP1619 | B.1.243 | 3/31/21 | surveillance | 99.7 | 4637 | hCoV-19/USA/VSP1619/2021 | MZ512649 |
| VSP1620 | B.1.1.7 | 3/31/21 | surveillance | 99.7 | 6507 | hCoV-19/USA/VSP1620/2021 | MZ512650 |
| VSP1621 | B.1.1.7 | 3/31/21 | surveillance | 99.7 | 4693 | hCoV-19/USA/VSP1621/2021 | MZ512651 |
| VSP1622 | B.1.1.7 | 3/31/21 | surveillance | 99.7 | 6782 | hCoV-19/USA/VSP1622/2021 | MZ512652 |
| VSP1623 | B.1.2 | 3/31/21 | surveillance | 99.7 | 6332 | hCoV-19/USA/VSP1623/2021 | MZ512653 |
| VSP1624 | R.1 | 3/31/21 | surveillance | 99.6 | 7737 | hCoV-19/USA/VSP1624/2021 | MZ512654 |
| VSP1625 | B.1.623 | 3/31/21 | surveillance | 99.6 | 5940 | hCoV-19/USA/VSP1625/2021 | MZ512655 |
| VSP1626 | B.1.2 | 3/31/21 | surveillance | 99.7 | 5022 | hCoV-19/USA/VSP1626/2021 | MZ512656 |
| VSP1627 | B.1.1.7 | 3/31/21 | surveillance | 99.7 | 4738 | hCoV-19/USA/VSP1627/2021 | MZ512657 |
| VSP1628 | B.1.1.7 | 3/31/21 | surveillance | 99.7 | 4508 | hCoV-19/USA/VSP1628/2021 | MZ512658 |
| VSP1629 | B.1.1.7 | 3/31/21 | surveillance | 99.7 | 3169 | hCoV-19/USA/VSP1629/2021 | MZ512659 |
| VSP1630 | P.1 | 3/31/21 | surveillance | 99.7 | 1709 | hCoV-19/USA/VSP1630/2021 | MZ512660 |
| VSP1631 | B.1.526 | 3/31/21 | surveillance | 99.7 | 4774 | hCoV-19/USA/VSP1631/2021 | MZ512661 |
| VSP1632 | B.1.1.434 | 3/31/21 | surveillance | 98.4 | 792 | hCoV-19/USA/VSP1632/2021 | |
| VSP1633 | B.1.575 | 3/31/21 | surveillance | 99.7 | 4941 | hCoV-19/USA/VSP1633/2021 | MZ512662 |
| VSP1634 | B.1.526 | 3/31/21 | surveillance | 99.7 | 11610 | hCoV-19/USA/VSP1634/2021 | MZ512663 |
| VSP1635 | B.1.2 | 3/31/21 | surveillance | 99.7 | 4380 | hCoV-19/USA/VSP1635/2021 | MZ512664 |
| VSP1636 | B.1.596 | 3/31/21 | surveillance | 99.6 | 564 | hCoV-19/USA/VSP1636/2021 | MZ512665 |
| VSP1637 | B.1.575 | 3/31/21 | surveillance | 99.7 | 7490 | hCoV-19/USA/VSP1637/2021 | MZ512666 |
| VSP1638 | B.1.2 | 3/31/21 | surveillance | 99.7 | 1998 | hCoV-19/USA/VSP1638/2021 | MZ512667 |
| VSP1639 | B.1.526 | 3/31/21 | surveillance | 99.6 | 486 | hCoV-19/USA/VSP1639/2021 | MZ512668 |
| VSP1640 | B.1.1.7 | 3/31/21 | surveillance | 99.7 | 1094 | hCoV-19/USA/VSP1640/2021 | MZ512669 |
| VSP1641 | B.1.2 | 3/31/21 | surveillance | 99.7 | 1280 | hCoV-19/USA/VSP1641/2021 | MZ512670 |
| VSP1642 | R.1 | 3/31/21 | surveillance | 99.7 | 6403 | hCoV-19/USA/VSP1642/2021 | MZ512671 |
| VSP1643 | B.1.1.519 | 3/31/21 | surveillance | 99.7 | 864 | hCoV-19/USA/VSP1643/2021 | MZ512672 |
| VSP1644 | B.1.526 | 3/31/21 | surveillance | 99.7 | 487 | hCoV-19/USA/VSP1644/2021 | MZ512673 |
| VSP1645 | B.1.1.519 | 3/31/21 | surveillance | 99.7 | 575 | hCoV-19/USA/VSP1645/2021 | MZ512674 |
| VSP1646 | B.1.1.7 | 3/31/21 | surveillance | 99.7 | 4304 | hCoV-19/USA/VSP1646/2021 | MZ512675 |
| VSP1647 | B.1.526 | 3/31/21 | surveillance | 99.7 | 1295 | hCoV-19/USA/VSP1647/2021 | MZ512676 |
| VSP1648 | B.1.1.7 | 3/31/21 | surveillance | 99.7 | 370 | hCoV-19/USA/VSP1648/2021 | MZ512677 |
| VSP1649 | B.1.1.7 | 3/31/21 | surveillance | 99.6 | 286 | hCoV-19/USA/VSP1649/2021 | MZ512678 |
| VSP1650 | B.1.1.7 | 3/31/21 | surveillance | 99.7 | 400 | hCoV-19/USA/VSP1650/2021 | MZ512679 |
| VSP1651 | B.1.1.519 | 3/31/21 | surveillance | 99.5 | 198 | hCoV-19/USA/VSP1651/2021 | MZ512680 |
| VSP1652 | B.1.526 | 3/31/21 | surveillance | 99.7 | 300 | hCoV-19/USA/VSP1652/2021 | MZ512681 |
| VSP1653 | B.1.234 | 3/31/21 | surveillance | 99.6 | 433 | hCoV-19/USA/VSP1653/2021 | MZ512682 |
| VSP1654 | B.1.1.519 | 3/31/21 | surveillance | 99.7 | 3324 | hCoV-19/USA/VSP1654/2021 | MZ512683 |
| VSP1655 | B.1.575 | 3/31/21 | surveillance | 99.7 | 783 | hCoV-19/USA/VSP1655/2021 | MZ512684 |
| VSP1656 | B.1.526 | 3/31/21 | surveillance | 99.3 | 120 | hCoV-19/USA/VSP1656/2021 | MZ512685 |
| VSP1657 | B.1.1.7 | 3/31/21 | surveillance | 99.7 | 2756 | hCoV-19/USA/VSP1657/2021 | MZ512686 |
| VSP1658 | B.1.1.7 | 3/31/21 | surveillance | 99.7 | 400 | hCoV-19/USA/VSP1658/2021 | MZ512687 |
| VSP1659 | B.1.526 | 3/31/21 | surveillance | 99.7 | 2361 | hCoV-19/USA/VSP1659/2021 | MZ512688 |
| VSP1660 | B.1.588 | 3/31/21 | surveillance | 99.5 | 1133 | hCoV-19/USA/VSP1660/2021 | MZ512689 |
| VSP1661 | B.1.1.519 | 3/31/21 | surveillance | 99.7 | 4758 | hCoV-19/USA/VSP1661/2021 | MZ512690 |

| | | | | | | | |
|---|---|---|---|---|---|---|---|
| VSP1662 | B.1.1.7 | 3/31/21 | surveillance | 99.6 | 480 | hCoV-19/USA/VSP1662/2021 | MZ512691 |
| VSP1663 | B.1.2 | 3/31/21 | surveillance | 99.3 | 117 | hCoV-19/USA/VSP1663/2021 | MZ512692 |
| VSP1664 | B.1.1.7 | 3/31/21 | surveillance | 99.5 | 290 | hCoV-19/USA/VSP1664/2021 | MZ512693 |
| VSP1665 | B.1.526 | 3/31/21 | surveillance | 99.7 | 1637 | hCoV-19/USA/VSP1665/2021 | MZ512694 |
| VSP1666 | B.1.575 | 3/31/21 | surveillance | 99.7 | 1239 | hCoV-19/USA/VSP1666/2021 | MZ512695 |
| VSP1667 | B.1.526 | 3/31/21 | surveillance | 99.7 | 482 | hCoV-19/USA/VSP1667/2021 | MZ512696 |
| VSP1668 | B.1.1.7 | 3/31/21 | surveillance | 99.7 | 3403 | hCoV-19/USA/VSP1668/2021 | MZ512697 |
| VSP1669 | B.1.1.7 | 3/31/21 | surveillance | 99.6 | 194 | hCoV-19/USA/VSP1669/2021 | MZ512698 |
| VSP1670 | B.1.1.7 | 3/31/21 | surveillance | 99.7 | 317 | hCoV-19/USA/VSP1670/2021 | MZ512699 |
| VSP1671 | B.1.526 | 3/31/21 | surveillance | 99.7 | 2108 | hCoV-19/USA/VSP1671/2021 | MZ512700 |
| VSP1672 | P.1.10 | 3/31/21 | surveillance | 99.6 | 264 | hCoV-19/USA/VSP1672/2021 | MZ512701 |
| VSP1673 | B.1.1.7 | 3/31/21 | surveillance | 99.7 | 3217 | hCoV-19/USA/VSP1673/2021 | MZ512702 |
| VSP1674 | B.1.526 | 3/31/21 | surveillance | 99.1 | 9195 | hCoV-19/USA/VSP1674/2021 | MZ512703 |
| VSP1675 | B.1.526 | 3/31/21 | surveillance | 99.7 | 2260 | hCoV-19/USA/VSP1675/2021 | MZ512704 |
| VSP1676 | B.1.526 | 3/31/21 | surveillance | 99.7 | 1106 | hCoV-19/USA/VSP1676/2021 | MZ512705 |
| VSP1677 | B.1.526 | 3/31/21 | surveillance | 99.6 | 938 | hCoV-19/USA/VSP1677/2021 | MZ512706 |
| VSP1678 | B.1.526 | 3/31/21 | surveillance | 98.9 | 159 | hCoV-19/USA/VSP1678/2021 | MZ512707 |
| VSP1679 | B.1.526 | 3/31/21 | surveillance | 99.7 | 2074 | hCoV-19/USA/VSP1679/2021 | MZ512708 |
| VSP1680 | B.1.2 | 3/31/21 | surveillance | 99.7 | 8549 | hCoV-19/USA/VSP1680/2021 | MZ512709 |
| VSP1681 | B.1.1.519 | 3/31/21 | surveillance | 99.7 | 473 | hCoV-19/USA/VSP1681/2021 | MZ512710 |
| VSP1683 | B.1.1.7 | 3/31/21 | surveillance | 99.7 | 2775 | hCoV-19/USA/VSP1683/2021 | MZ512711 |
| VSP1684 | B.1.637 | 3/31/21 | surveillance | 99.7 | 3133 | hCoV-19/USA/VSP1684/2021 | MZ512712 |
| VSP1685 | B.1.1.7 | 3/31/21 | surveillance | 99.7 | 615 | hCoV-19/USA/VSP1685/2021 | MZ512713 |
| VSP1686 | B.1.1.7 | 3/31/21 | surveillance | 99.7 | 1200 | hCoV-19/USA/VSP1686/2021 | MZ512714 |
| VSP1687 | B.1.526 | 3/31/21 | surveillance | 99.6 | 497 | hCoV-19/USA/VSP1687/2021 | MZ512715 |
| VSP1688 | B.1.526 | 3/31/21 | surveillance | 99.7 | 5578 | hCoV-19/USA/VSP1688/2021 | MZ512716 |
| VSP1689 | B.1.1.7 | 3/31/21 | surveillance | 99.7 | 1737 | hCoV-19/USA/VSP1689/2021 | MZ512717 |
| VSP1690 | B.1.526 | 3/31/21 | surveillance | 99.7 | 729 | hCoV-19/USA/VSP1690/2021 | MZ512718 |
| VSP1691 | B.1.1.7 | 3/31/21 | surveillance | 99.7 | 17752 | hCoV-19/USA/VSP1691/2021 | MZ512719 |
| VSP1692 | B.1.1.7 | 3/31/21 | surveillance | 99.6 | 462 | hCoV-19/USA/VSP1692/2021 | MZ512720 |
| VSP1693 | B.1.526 | 3/31/21 | surveillance | 99.7 | 3653 | hCoV-19/USA/VSP1693/2021 | MZ512721 |
| VSP1695 | B.1.1.7 | 3/31/21 | surveillance | 99.7 | 5356 | hCoV-19/USA/VSP1695/2021 | MZ512722 |
| VSP1696 | B.1.1.519 | 3/31/21 | surveillance | 99.7 | 4405 | hCoV-19/USA/VSP1696/2021 | MZ512723 |
| VSP1697 | B.1.526 | 3/31/21 | surveillance | 99.7 | 3351 | hCoV-19/USA/VSP1697/2021 | MZ512724 |
| VSP1698 | B.1.526 | 3/31/21 | surveillance | 99.7 | 1907 | hCoV-19/USA/VSP1698/2021 | MZ512725 |
| VSP1699 | B.1.1.519 | 3/31/21 | surveillance | 99.7 | 8812 | hCoV-19/USA/VSP1699/2021 | MZ512726 |
| VSP1700 | B.1.526 | 3/31/21 | surveillance | 99.7 | 12498 | hCoV-19/USA/VSP1700/2021 | MZ512727 |
| VSP1701 | B.1.1.7 | 3/31/21 | surveillance | 99.7 | 3592 | hCoV-19/USA/VSP1701/2021 | MZ512728 |
| VSP1702 | B.1.1.7 | 3/31/21 | surveillance | 99.7 | 1037 | hCoV-19/USA/VSP1702/2021 | MZ512729 |
| VSP1703 | B.1.243 | 3/31/21 | surveillance | 99.1 | 6070 | hCoV-19/USA/VSP1703/2021 | MZ512730 |
| VSP1704 | B.1.2 | 3/31/21 | surveillance | 99.7 | 630 | hCoV-19/USA/VSP1704/2021 | MZ512731 |
| VSP1705 | B.1.1.7 | 3/31/21 | surveillance | 99.7 | 5918 | hCoV-19/USA/VSP1705/2021 | MZ512732 |
| VSP1706 | B.1.1.7 | 3/31/21 | surveillance | 99.7 | 803 | hCoV-19/USA/VSP1706/2021 | MZ512733 |
| VSP1707 | B.1.1.7 | 3/31/21 | surveillance | 99.7 | 1171 | hCoV-19/USA/VSP1707/2021 | MZ512734 |
| VSP1708 | B.1.526 | 3/31/21 | surveillance | 97.8 | 2276 | hCoV-19/USA/VSP1708/2021 | MZ512735 |
| VSP1709 | B.1.637 | 3/31/21 | surveillance | 98.9 | 362 | hCoV-19/USA/VSP1709/2021 | MZ512736 |
| VSP1710 | B.1.1.519 | 3/31/21 | surveillance | 99.7 | 5507 | hCoV-19/USA/VSP1710/2021 | MZ512737 |

| | | | | | | | |
|---|---|---|---|---|---|---|---|
| VSP1711 | B.1.526 | 3/31/21 | surveillance | 99.7 | 4793 | hCoV-19/USA/VSP1711/2021 | MZ512738 |
| VSP1712 | B.1.526 | 3/31/21 | surveillance | 99.7 | 658 | hCoV-19/USA/VSP1712/2021 | MZ512739 |
| VSP1713 | B.1.526 | 3/31/21 | surveillance | 99.7 | 4171 | hCoV-19/USA/VSP1713/2021 | MZ512740 |
| VSP1714 | B.1.526 | 3/31/21 | surveillance | 99.7 | 4457 | hCoV-19/USA/VSP1714/2021 | MZ512741 |
| VSP1715 | B.1.2 | 3/31/21 | surveillance | 99.7 | 2311 | hCoV-19/USA/VSP1715/2021 | MZ512742 |
| VSP1716 | B.1.234 | 3/31/21 | surveillance | 99.7 | 423 | hCoV-19/USA/VSP1716/2021 | MZ512743 |
| VSP1717 | B.1.1.519 | 3/31/21 | surveillance | 99.7 | 11856 | hCoV-19/USA/VSP1717/2021 | MZ512744 |
| VSP1718 | B.1.526 | 3/31/21 | surveillance | 99.6 | 404 | hCoV-19/USA/VSP1718/2021 | MZ512745 |
| VSP1719 | B.1.526 | 3/31/21 | surveillance | 99.7 | 7563 | hCoV-19/USA/VSP1719/2021 | MZ512746 |
| VSP1721 | B.1.1.7 | 3/31/21 | hospitalized | 97.6 | 719 | hCoV-19/USA/PA-VSP1721/2021 | OK245941 |
| VSP1738 | B.1.1.7 | 4/5/21 | s drop | 99.7 | 3681 | hCoV-19/USA/PA-VSP1738/2021 | OK245942 |
| VSP1739 | B.1.1.7 | 4/5/21 | s drop | 99.7 | 3065 | hCoV-19/USA/PA-VSP1739/2021 | OK245943 |
| VSP1740 | B.1.1.7 | 4/5/21 | s drop | 99.7 | 1905 | hCoV-19/USA/PA-VSP1740/2021 | OK245944 |
| VSP1741 | B.1.1.7 | 4/5/21 | s drop | 99.7 | 2105 | hCoV-19/USA/PA-VSP1741/2021 | OK245945 |
| VSP1742 | B.1.1.7 | 4/5/21 | s drop | 99.7 | 1845 | hCoV-19/USA/PA-VSP1742/2021 | MZ156658 |
| VSP1743 | B.1.1.7 | 4/5/21 | s drop | 99.7 | 2540 | hCoV-19/USA/PA-VSP1743/2021 | MZ156659 |
| VSP1744 | B.1.1.7 | 4/5/21 | s drop | 99.7 | 2240 | hCoV-19/USA/NJ-VSP1744/2021 | MZ156660 |
| VSP1746 | B.1.1.7 | 4/5/21 | s drop | 99.7 | 2647 | hCoV-19/USA/NJ-VSP1746/2021 | MZ156661 |
| VSP1748 | B.1.1.7 | 4/5/21 | s drop | 99.7 | 2321 | hCoV-19/USA/PA-VSP1748/2021 | MZ156662 |
| VSP1749 | B.1.1.7 | 4/5/21 | s drop | 99.7 | 2601 | hCoV-19/USA/PA-VSP1749/2021 | MZ156663 |
| VSP1750 | B.1.1.7 | 4/5/21 | s drop | 99.7 | 2410 | hCoV-19/USA/PA-VSP1750/2021 | MZ156664 |
| VSP1751 | B.1.1.7 | 4/5/21 | s drop | 99.7 | 2239 | hCoV-19/USA/PA-VSP1751/2021 | MZ156665 |
| VSP1752 | B.1.1.7 | 4/5/21 | s drop | 99.7 | 2034 | hCoV-19/USA/PA-VSP1752/2021 | MZ156666 |
| VSP1755 | B.1.1.7 | 4/5/21 | s drop | 99.7 | 2404 | hCoV-19/USA/PA-VSP1755/2021 | MZ156667 |
| VSP1756 | B.1.1.7 | 4/5/21 | s drop | 97.9 | 2258 | hCoV-19/USA/PA-VSP1756/2021 | MZ156668 |
| VSP1758 | B.1.1.7 | 4/5/21 | s drop | 96.6 | 1472 | hCoV-19/USA/DE-VSP1758/2021 | |
| VSP1760 | B.1.1.7 | 4/5/21 | s drop | 99.2 | 2377 | hCoV-19/USA/PA-VSP1760/2021 | MZ156669 |
| VSP1761 | B.1.1.7 | 4/5/21 | s drop | 99.7 | 1980 | hCoV-19/USA/PA-VSP1761/2021 | MZ156670 |
| VSP1762 | B.1.1.7 | 4/5/21 | s drop | 99.7 | 1611 | hCoV-19/USA/PA-VSP1762/2021 | MZ156671 |
| VSP1764 | B.1.1.7 | 4/5/21 | s drop | 99.7 | 1642 | hCoV-19/USA/PA-VSP1764/2021 | MZ156672 |
| VSP1765 | B.1.1.7 | 4/5/21 | s drop | 99.7 | 2535 | hCoV-19/USA/PA-VSP1765/2021 | MZ156673 |
| VSP1766 | B.1.1.7 | 4/5/21 | s drop | 99.7 | 1916 | hCoV-19/USA/PA-VSP1766/2021 | MZ156674 |
| VSP1767 | B.1.1.7 | 4/5/21 | s drop | 99.7 | 2038 | hCoV-19/USA/PA-VSP1767/2021 | MZ156675 |
| VSP1768 | B.1.1.7 | 4/5/21 | s drop | 99.7 | 1936 | hCoV-19/USA/PA-VSP1768/2021 | MZ156676 |
| VSP1769 | B.1.1.7 | 4/5/21 | s drop | 99.7 | 1542 | hCoV-19/USA/PA-VSP1769/2021 | MZ156677 |
| VSP1770 | B.1.1.7 | 4/5/21 | s drop | 99.7 | 1847 | hCoV-19/USA/PA-VSP1770/2021 | MZ156678 |
| VSP1771 | B.1.1.7 | 4/5/21 | s drop | 99.7 | 1383 | hCoV-19/USA/PA-VSP1771/2021 | MZ156679 |
| VSP1772 | B.1.1.7 | 4/5/21 | s drop | 99.7 | 2332 | hCoV-19/USA/PA-VSP1772/2021 | MZ156680 |
| VSP1773 | B.1.1.7 | 4/5/21 | s drop | 99.7 | 2034 | hCoV-19/USA/PA-VSP1773/2021 | MZ156681 |
| VSP1774 | B.1.1.7 | 4/5/21 | s drop | 99.7 | 1861 | hCoV-19/USA/PA-VSP1774/2021 | MZ156682 |
| VSP1775 | B.1.1.7 | 4/5/21 | s drop | 99.7 | 1785 | hCoV-19/USA/PA-VSP1775/2021 | MZ156683 |
| VSP1776 | B.1.1.7 | 4/5/21 | s drop | 99.7 | 1913 | hCoV-19/USA/PA-VSP1776/2021 | MZ156684 |
| VSP1777 | B.1.1.7 | 4/5/21 | s drop | 99.7 | 2938 | hCoV-19/USA/PA-VSP1777/2021 | MZ156685 |
| VSP1778 | B.1.1.7 | 4/5/21 | s drop | 99.7 | 1518 | hCoV-19/USA/PA-VSP1778/2021 | MZ156686 |
| VSP1779 | B.1.1.7 | 4/5/21 | s drop | 99.7 | 2394 | hCoV-19/USA/PA-VSP1779/2021 | MZ156687 |
| VSP1780 | B.1.575 | 4/5/21 | surveillance | 99.7 | 2431 | hCoV-19/USA/PA-VSP1780/2021 | MZ156688 |
| VSP1781 | B.1.637 | 4/5/21 | surveillance | 98.9 | 1119 | hCoV-19/USA/PA-VSP1781/2021 | MZ156689 |

| | | | | | | | |
|---|---|---|---|---|---|---|---|
| VSP1782 | B.1.526 | 4/5/21 | surveillance | 99.7 | 2205 | hCoV-19/USA/PA-VSP1782/2021 | MZ156690 |
| VSP1783 | B.1.526 | 4/5/21 | surveillance | 99.7 | 2377 | hCoV-19/USA/PA-VSP1783/2021 | MZ156691 |
| VSP1784 | B.1.575 | 4/5/21 | surveillance | 99.7 | 1962 | hCoV-19/USA/PA-VSP1784/2021 | MZ156692 |
| VSP1785 | B.1.526 | 4/5/21 | surveillance | 99.7 | 1783 | hCoV-19/USA/PA-VSP1785/2021 | MZ156693 |
| VSP1786 | R.1 | 4/5/21 | surveillance | 99.7 | 2070 | hCoV-19/USA/PA-VSP1786/2021 | MZ156694 |
| VSP1787 | B.1.637 | 4/5/21 | surveillance | 99.6 | 160 | hCoV-19/USA/VSP1787/2021 | MZ512747 |
| VSP1788 | B.1.2 | 4/5/21 | surveillance | 99.6 | 219 | hCoV-19/USA/VSP1788/2021 | MZ512748 |
| VSP1789 | B.1.526 | 4/5/21 | surveillance | 99.7 | 198 | hCoV-19/USA/VSP1789/2021 | MZ512749 |
| VSP1791 | B.1.526 | 4/5/21 | surveillance | 95.8 | 45 | hCoV-19/USA/VSP1791/2021 | MZ512750 |
| VSP1792 | B.1.575 | 4/5/21 | surveillance | 96 | 66 | hCoV-19/USA/VSP1792/2021 | MZ512751 |
| VSP1793 | B.1.1.7 | 4/5/21 | surveillance | 99.5 | 192 | hCoV-19/USA/VSP1793/2021 | MZ512752 |
| VSP1794 | B.1.429 | 4/5/21 | surveillance | 99.6 | 236 | hCoV-19/USA/VSP1794/2021 | MZ512753 |
| VSP1797 | B.1.2 | 4/5/21 | surveillance | 99.5 | 183 | hCoV-19/USA/VSP1797/2021 | MZ512754 |
| VSP1798 | B.1.2 | 4/5/21 | surveillance | 99.6 | 116 | hCoV-19/USA/VSP1798/2021 | MZ512755 |
| VSP1799 | B.1.243 | 4/5/21 | surveillance | 98.1 | 258 | hCoV-19/USA/VSP1799/2021 | MZ512756 |
| VSP1800 | B.1.2 | 4/5/21 | surveillance | 96.4 | 30 | hCoV-19/USA/VSP1800/2021 | MZ512757 |
| VSP1801 | B.1.1.7 | 4/5/21 | surveillance | 99 | 111 | hCoV-19/USA/VSP1801/2021 | MZ512758 |
| VSP1802 | B.1.2 | 4/5/21 | surveillance | 99.5 | 57 | hCoV-19/USA/VSP1802/2021 | MZ512759 |
| VSP1803 | B.1.1.519 | 4/5/21 | surveillance | 99.7 | 165 | hCoV-19/USA/VSP1803/2021 | MZ512760 |
| VSP1804 | B.1.1.7 | 4/5/21 | surveillance | 99.6 | 208 | hCoV-19/USA/VSP1804/2021 | MZ512761 |
| VSP1805 | B.1.1.7 | 4/5/21 | surveillance | 99.6 | 228 | hCoV-19/USA/VSP1805/2021 | MZ512762 |
| VSP1806 | B.1.1.7 | 4/5/21 | surveillance | 96.7 | 46 | hCoV-19/USA/VSP1806/2021 | MZ512763 |
| VSP1807 | B.1.637 | 4/5/21 | surveillance | 96.9 | 66 | hCoV-19/USA/VSP1807/2021 | MZ512764 |
| VSP1808 | B.1.1.7 | 4/5/21 | surveillance | 96.8 | 64 | hCoV-19/USA/VSP1808/2021 | MZ512765 |
| VSP1810 | B.1.351 | 4/5/21 | surveillance | 99 | 227 | hCoV-19/USA/VSP1810/2021 | MZ512766 |
| VSP1811 | B.1.1.7 | 4/5/21 | surveillance | 99.3 | 216 | hCoV-19/USA/VSP1811/2021 | MZ512767 |
| VSP1812 | B.1.526 | 4/5/21 | surveillance | 98.9 | 123 | hCoV-19/USA/VSP1812/2021 | MZ512768 |
| VSP1813 | B.1.1.7 | 4/5/21 | surveillance | 96.5 | 60 | hCoV-19/USA/VSP1813/2021 | MZ512769 |
| VSP1815 | R.1 | 4/5/21 | surveillance | 96 | 48 | hCoV-19/USA/VSP1815/2021 | MZ512770 |
| VSP1816 | B.1.526 | 4/5/21 | surveillance | 99.2 | 166 | hCoV-19/USA/VSP1816/2021 | MZ512771 |
| VSP1818 | B.1.526 | 4/5/21 | surveillance | 99.6 | 252 | hCoV-19/USA/VSP1818/2021 | MZ512772 |
| VSP1819 | B.1.1.7 | 4/5/21 | surveillance | 98.6 | 153 | hCoV-19/USA/VSP1819/2021 | MZ512773 |
| VSP1820 | B.1.1.7 | 4/5/21 | surveillance | 99.3 | 202 | hCoV-19/USA/VSP1820/2021 | MZ512774 |
| VSP1821 | B.1.2 | 4/5/21 | surveillance | 99.6 | 204 | hCoV-19/USA/VSP1821/2021 | MZ512775 |
| VSP1822 | B.1.526 | 4/5/21 | surveillance | 98.6 | 98 | hCoV-19/USA/VSP1822/2021 | MZ512776 |
| VSP1823 | B.1.2 | 4/5/21 | surveillance | 99.6 | 214 | hCoV-19/USA/VSP1823/2021 | MZ512777 |
| VSP1824 | B.1.1.7 | 4/5/21 | surveillance | 99.3 | 149 | hCoV-19/USA/VSP1824/2021 | MZ512778 |
| VSP1825 | B.1.1.7 | 4/5/21 | surveillance | 99.5 | 144 | hCoV-19/USA/VSP1825/2021 | MZ512779 |
| VSP1827 | B.1.1.7 | 4/5/21 | surveillance | 99.5 | 211 | hCoV-19/USA/VSP1827/2021 | MZ512780 |
| VSP1828 | B.1.110.3 | 4/5/21 | surveillance | 95.9 | 51 | hCoV-19/USA/VSP1828/2021 | MZ512781 |
| VSP1830 | B.1.1.7 | 4/5/21 | surveillance | 99.3 | 162 | hCoV-19/USA/VSP1830/2021 | MZ512782 |
| VSP1832 | B.1.2 | 4/5/21 | surveillance | 99.7 | 7417 | hCoV-19/USA/VSP1832/2021 | MZ512783 |
| VSP1833 | B.1.311 | 4/5/21 | surveillance | 99.7 | 10172 | hCoV-19/USA/VSP1833/2021 | MZ512784 |
| VSP1834 | B.1.1.7 | 4/5/21 | surveillance | 99.7 | 5496 | hCoV-19/USA/VSP1834/2021 | MZ512785 |
| VSP1835 | B.1.526 | 4/5/21 | surveillance | 99.7 | 6898 | hCoV-19/USA/VSP1835/2021 | MZ512786 |
| VSP1836 | B.1.1.7 | 4/5/21 | surveillance | 99.7 | 624 | hCoV-19/USA/VSP1836/2021 | MZ512787 |
| VSP1837 | B.1.526 | 4/5/21 | surveillance | 99.7 | 21704 | hCoV-19/USA/VSP1837/2021 | MZ512788 |

| | | | | | | | |
|---|---|---|---|---|---|---|---|
| VSP1838 | P.1 | 4/5/21 | surveillance | 99.7 | 11479 | hCoV-19/USA/VSP1838/2021 | MZ512789 |
| VSP1839 | B.1.1.7 | 4/5/21 | surveillance | 99.7 | 450 | hCoV-19/USA/VSP1839/2021 | MZ512790 |
| VSP1840 | B.1.526 | 4/5/21 | surveillance | 99.1 | 989 | hCoV-19/USA/DE-VSP1840/2021 | OK245946 |
| VSP1841 | B.1.1.7 | 4/5/21 | surveillance | 99.7 | 2109 | hCoV-19/USA/PA-VSP1841/2021 | OK245947 |
| VSP1842 | B.1.637 | 4/5/21 | surveillance | 99.6 | 1507 | hCoV-19/USA/NJ-VSP1842/2021 | OK245948 |
| VSP1843 | B.1.526 | 4/5/21 | surveillance | 99.7 | 2045 | hCoV-19/USA/PA-VSP1843/2021 | OK245949 |
| VSP1844 | B.1.351 | 4/5/21 | surveillance | 98.6 | 1206 | hCoV-19/USA/PA-VSP1844/2021 | OK245950 |
| VSP1845 | B.1.1.7 | 4/5/21 | surveillance | 99.7 | 3077 | hCoV-19/USA/PA-VSP1845/2021 | OK245951 |
| VSP1846 | B.1.1.7 | 4/5/21 | surveillance | 99.7 | 1321 | hCoV-19/USA/PA-VSP1846/2021 | OK245952 |
| VSP1847 | B.1.1.7 | 4/5/21 | surveillance | 99.7 | 2081 | hCoV-19/USA/PA-VSP1847/2021 | OK245953 |
| VSP1848 | B.1.1.7 | 4/5/21 | surveillance | 99.7 | 4055 | hCoV-19/USA/PA-VSP1848/2021 | OK245954 |
| VSP1849 | B.1.1.7 | 4/5/21 | surveillance | 99.7 | 2201 | hCoV-19/USA/PA-VSP1849/2021 | OK245955 |
| VSP1850 | B.1.1.7 | 4/5/21 | surveillance | 99.7 | 1987 | hCoV-19/USA/PA-VSP1850/2021 | OK245956 |
| VSP1851 | B.1.1.7 | 4/5/21 | surveillance | 99.1 | 524 | hCoV-19/USA/PA-VSP1851/2021 | OK245957 |
| VSP1852 | B.1.1.7 | 4/5/21 | surveillance | 99.7 | 1360 | hCoV-19/USA/PA-VSP1852/2021 | OK245958 |
| VSP1853 | B.1.526 | 4/5/21 | surveillance | 99.5 | 1753 | hCoV-19/USA/PA-VSP1853/2021 | OK245959 |
| VSP1854 | B.1.1.7 | 4/5/21 | surveillance | 99.1 | 406 | hCoV-19/USA/PA-VSP1854/2021 | OK245960 |
| VSP1855 | B.1.526 | 4/5/21 | surveillance | 95.3 | 77 | hCoV-19/USA/PA-VSP1855/2021 | OK245961 |
| VSP1856 | B.1.526 | 4/5/21 | surveillance | 99.6 | 1997 | hCoV-19/USA/PA-VSP1856/2021 | OK245962 |
| VSP1857 | B.1.1.7 | 4/5/21 | surveillance | 99.5 | 2176 | hCoV-19/USA/PA-VSP1857/2021 | OK245963 |
| VSP1858 | B.1.1.7 | 4/5/21 | surveillance | 99.5 | 1491 | hCoV-19/USA/PA-VSP1858/2021 | OK245964 |
| VSP1859 | B.1.526 | 4/5/21 | surveillance | 99.6 | 1858 | hCoV-19/USA/PA-VSP1859/2021 | OK245965 |
| VSP1860 | B.1.1.7 | 4/5/21 | surveillance | 99.7 | 2911 | hCoV-19/USA/PA-VSP1860/2021 | OK245966 |
| VSP1861 | B.1.1.7 | 4/5/21 | surveillance | 99.7 | 2509 | hCoV-19/USA/PA-VSP1861/2021 | OK245967 |
| VSP1862 | B.1.1.7 | 4/5/21 | surveillance | 98.2 | 233 | hCoV-19/USA/PA-VSP1862/2021 | OK245968 |
| VSP1863 | B.1.526 | 4/5/21 | surveillance | 97.6 | 252 | hCoV-19/USA/PA-VSP1863/2021 | OK245969 |
| VSP1864 | B.1.526 | 4/5/21 | surveillance | 99.7 | 2582 | hCoV-19/USA/PA-VSP1864/2021 | OK245970 |
| VSP1865 | B.1.637 | 4/5/21 | surveillance | 99.1 | 557 | hCoV-19/USA/PA-VSP1865/2021 | OK245971 |
| VSP1866 | B.1.526 | 4/5/21 | surveillance | 99.2 | 1543 | hCoV-19/USA/PA-VSP1866/2021 | OK245972 |
| VSP1867 | B.1.243 | 4/5/21 | surveillance | 99.7 | 889 | hCoV-19/USA/PA-VSP1867/2021 | OK245973 |
| VSP1868 | B.1.1.7 | 4/5/21 | surveillance | 99.7 | 1702 | hCoV-19/USA/NJ-VSP1868/2021 | OK245974 |
| VSP1869 | B.1.526 | 4/5/21 | surveillance | 99.6 | 849 | hCoV-19/USA/NJ-VSP1869/2021 | OK245975 |
| VSP1870 | B.1.1.7 | 4/5/21 | surveillance | 99.7 | 1046 | hCoV-19/USA/NJ-VSP1870/2021 | OK245976 |
| VSP1871 | B.1.1.7 | 4/5/21 | surveillance | 99.7 | 1481 | hCoV-19/USA/NJ-VSP1871/2021 | OK245977 |
| VSP1872 | B.1.526 | 4/5/21 | surveillance | 99.7 | 1015 | hCoV-19/USA/NJ-VSP1872/2021 | OK245978 |
| VSP1873 | B.1.1.7 | 4/5/21 | surveillance | 97.7 | 148 | hCoV-19/USA/NJ-VSP1873/2021 | OK245979 |
| VSP1874 | B.1.526 | 4/5/21 | surveillance | 99 | 251 | hCoV-19/USA/NJ-VSP1874/2021 | OK245980 |
| VSP1875 | B.1.1.7 | 4/5/21 | surveillance | 97.5 | 162 | hCoV-19/USA/NJ-VSP1875/2021 | OK245981 |
| VSP1876 | B.1.526 | 4/5/21 | surveillance | 99.7 | 1830 | hCoV-19/USA/NJ-VSP1876/2021 | OK245982 |
| VSP1877 | B.1.243 | 4/5/21 | surveillance | 99.7 | 9999 | hCoV-19/USA/PA-VSP1877/2021 | MZ156695 |
| VSP1878 | B.1.1.7 | 4/5/21 | surveillance | 99.7 | 8219 | hCoV-19/USA/PA-VSP1878/2021 | MZ156696 |
| VSP1879 | B.1.637 | 4/5/21 | surveillance | 99.7 | 8699 | hCoV-19/USA/PA-VSP1879/2021 | MZ156697 |
| VSP1880 | P.1 | 4/5/21 | surveillance | 99.7 | 8772 | hCoV-19/USA/PA-VSP1880/2021 | MZ156698 |
| VSP1881 | B.1.1.7 | 4/5/21 | surveillance | 99.7 | 8284 | hCoV-19/USA/PA-VSP1881/2021 | MZ156699 |
| VSP1882 | B.1.526 | 4/5/21 | surveillance | 99.7 | 10154 | hCoV-19/USA/PA-VSP1882/2021 | MZ156700 |
| VSP1883 | B.1.526 | 4/5/21 | surveillance | 99.7 | 8817 | hCoV-19/USA/PA-VSP1883/2021 | MZ156701 |
| VSP1884 | B.1.1.7 | 4/5/21 | surveillance | 99.7 | 10616 | hCoV-19/USA/PA-VSP1884/2021 | MZ156702 |

| | | | | | | | |
|---|---|---|---|---|---|---|---|
| VSP1885 | B.1.111 | 4/5/21 | surveillance | 99.7 | 9357 | hCoV-19/USA/PA-VSP1885/2021 | MZ156703 |
| VSP1886 | B.1.111 | 4/5/21 | surveillance | 99.7 | 7339 | hCoV-19/USA/PA-VSP1886/2021 | MZ156704 |
| VSP1887 | B.1.1.519 | 4/5/21 | surveillance | 99.7 | 8768 | hCoV-19/USA/PA-VSP1887/2021 | MZ156705 |
| VSP1888 | P.1 | 4/5/21 | surveillance | 99.7 | 9197 | hCoV-19/USA/PA-VSP1888/2021 | MZ156706 |
| VSP1889 | B.1.1.7 | 4/5/21 | surveillance | 99.7 | 9678 | hCoV-19/USA/PA-VSP1889/2021 | MZ156707 |
| VSP1890 | B.1.1.7 | 4/5/21 | surveillance | 99.7 | 9178 | hCoV-19/USA/NJ-VSP1890/2021 | MZ156708 |
| VSP1891 | B.1.1.7 | 4/5/21 | surveillance | 99.7 | 10319 | hCoV-19/USA/PA-VSP1891/2021 | MZ156709 |
| VSP1892 | B.1.1.7 | 4/5/21 | surveillance | 99.7 | 4912 | hCoV-19/USA/NY-VSP1892/2021 | MZ156710 |
| VSP1893 | B.1.526 | 4/5/21 | surveillance | 99.7 | 6143 | hCoV-19/USA/PA-VSP1893/2021 | MZ156711 |
| VSP1894 | B.1.234 | 4/5/21 | surveillance | 99.7 | 6969 | hCoV-19/USA/PA-VSP1894/2021 | MZ156712 |
| VSP1895 | B.1.1.7 | 4/5/21 | surveillance | 99.7 | 9740 | hCoV-19/USA/NJ-VSP1895/2021 | MZ156713 |
| VSP1897 | B.1.1.7 | 4/5/21 | surveillance | 99.7 | 5426 | hCoV-19/USA/PA-VSP1897/2021 | MZ156714 |
| VSP1898 | B.1.1.7 | 4/5/21 | surveillance | 99.7 | 8841 | hCoV-19/USA/PA-VSP1898/2021 | MZ156715 |
| VSP1899 | B.1.1.7 | 4/5/21 | surveillance | 99.7 | 7107 | hCoV-19/USA/PA-VSP1899/2021 | MZ156716 |
| VSP1900 | B.1.1.7 | 4/5/21 | surveillance | 99.7 | 7322 | hCoV-19/USA/PA-VSP1900/2021 | MZ156717 |
| VSP1901 | P.1.10 | 4/5/21 | surveillance | 99.7 | 4248 | hCoV-19/USA/PA-VSP1901/2021 | MZ156718 |
| VSP1902 | B.1.1.7 | 4/5/21 | surveillance | 99.7 | 8371 | hCoV-19/USA/PA-VSP1902/2021 | MZ156719 |
| VSP1903 | B.1.1.7 | 4/5/21 | surveillance | 98.4 | 260 | hCoV-19/USA/PA-VSP1903/2021 | MZ156720 |
| VSP1906 | B.1.526 | 4/5/21 | surveillance | 99.7 | 8563 | hCoV-19/USA/PA-VSP1906/2021 | MZ156722 |
| VSP1907 | R.1 | 4/5/21 | surveillance | 99.7 | 7977 | hCoV-19/USA/PA-VSP1907/2021 | MZ156723 |
| VSP1908 | B.1.526 | 4/5/21 | surveillance | 99.7 | 6786 | hCoV-19/USA/PA-VSP1908/2021 | MZ156724 |
| VSP1909 | B.1.429 | 4/5/21 | surveillance | 99.7 | 2127 | hCoV-19/USA/PA-VSP1909/2021 | MZ156725 |
| VSP1910 | B.1.526 | 4/5/21 | surveillance | 99.7 | 7944 | hCoV-19/USA/PA-VSP1910/2021 | MZ156726 |
| VSP1911 | B.1.1.7 | 4/5/21 | surveillance | 99.7 | 7038 | hCoV-19/USA/PA-VSP1911/2021 | MZ156727 |
| VSP1912 | B.1.111 | 4/5/21 | surveillance | 99.7 | 8535 | hCoV-19/USA/PA-VSP1912/2021 | MZ156728 |
| VSP1913 | B.1.243 | 4/5/21 | surveillance | 99.7 | 9482 | hCoV-19/USA/PA-VSP1913/2021 | MZ156729 |
| VSP1914 | B.1.1.7 | 4/5/21 | surveillance | 99.7 | 7868 | hCoV-19/USA/PA-VSP1914/2021 | MZ156730 |
| VSP1915 | B.1.1.519 | 4/5/21 | surveillance | 99.3 | 2629 | hCoV-19/USA/PA-VSP1915/2021 | |
| VSP1916 | B.1.1.7 | 4/5/21 | surveillance | 99.7 | 8306 | hCoV-19/USA/PA-VSP1916/2021 | OK245983 |
| VSP1917 | B.1.526 | 4/5/21 | surveillance | 96.6 | 2688 | hCoV-19/USA/PA-VSP1917/2021 | MZ156731 |
| VSP1918 | B.1.526 | 4/5/21 | surveillance | 99.7 | 6519 | hCoV-19/USA/PA-VSP1918/2021 | MZ156732 |
| VSP1919 | B.1.526 | 4/5/21 | surveillance | 99.7 | 4267 | hCoV-19/USA/PA-VSP1919/2021 | MZ156733 |
| VSP1922 | P.1 | 4/5/21 | surveillance | 99.7 | 6972 | hCoV-19/USA/PA-VSP1922/2021 | MZ156735 |
| VSP1923 | B.1.429 | 4/5/21 | surveillance | 99.1 | 1176 | hCoV-19/USA/PA-VSP1923/2021 | OK245984 |
| VSP1924 | B.1.637 | 4/5/21 | surveillance | 99.7 | 5342 | hCoV-19/USA/PA-VSP1924/2021 | MZ156736 |
| VSP1934 | B.1.1.7 | 4/5/21 | surveillance | 99.3 | 3893 | hCoV-19/USA/VA-VSP1934/2021 | MZ156737 |
| VSP1935 | B.1.526 | 4/5/21 | surveillance | 99.7 | 4442 | hCoV-19/USA/PA-VSP1935/2021 | MZ156738 |
| VSP1936 | B.1.243 | 4/5/21 | surveillance | 99.7 | 4644 | hCoV-19/USA/PA-VSP1936/2021 | MZ156739 |
| VSP1937 | B.1.1.7 | 4/5/21 | surveillance | 99.7 | 5423 | hCoV-19/USA/PA-VSP1937/2021 | MZ156740 |
| VSP1943 | B.1.1.7 | 4/5/21 | surveillance | 96.5 | 141 | hCoV-19/USA/PA-VSP1943/2021 | MZ156741 |
| VSP1945 | B.1.637 | 4/5/21 | surveillance | 97.8 | 264 | hCoV-19/USA/NJ-VSP1945/2021 | MZ156742 |
| VSP1946 | B.1.111 | 4/5/21 | surveillance | 99.7 | 5888 | hCoV-19/USA/PA-VSP1946/2021 | MZ156743 |
| VSP1947 | B.1.1.7 | 4/5/21 | surveillance | 99.1 | 3578 | hCoV-19/USA/PA-VSP1947/2021 | MZ156744 |
| VSP1948 | P.1 | 4/5/21 | surveillance | 99.7 | 1617 | hCoV-19/USA/PA-VSP1948/2021 | MZ156745 |
| VSP1949 | B.1.1.7 | 4/5/21 | surveillance | 99.1 | 4040 | hCoV-19/USA/PA-VSP1949/2021 | MZ156746 |
| VSP1952 | B.1.2 | 4/5/21 | surveillance | 99.1 | 3924 | hCoV-19/USA/PA-VSP1952/2021 | MZ156747 |
| VSP1953 | B.1.526 | 4/5/21 | surveillance | 99.1 | 1872 | hCoV-19/USA/PA-VSP1953/2021 | MZ156748 |

| | | | | | | | |
|---|---|---|---|---|---|---|---|
| VSP1955 | B.1.526 | 4/5/21 | surveillance | 99 | 1265 | hCoV-19/USA/PA-VSP1955/2021 | MZ156749 |
| VSP1957 | B.1.526 | 4/5/21 | surveillance | 99.1 | 3519 | hCoV-19/USA/PA-VSP1957/2021 | MZ156750 |
| VSP1960 | B.1.526 | 4/5/21 | surveillance | 99.1 | 2457 | hCoV-19/USA/NJ-VSP1960/2021 | MZ156751 |
| VSP1961 | B.1.526 | 4/5/21 | surveillance | 99.7 | 2330 | hCoV-19/USA/PA-VSP1961/2021 | MZ156752 |
| VSP1963 | B.1.2 | 4/5/21 | surveillance | 99.1 | 429 | hCoV-19/USA/PA-VSP1963/2021 | MZ156753 |
| VSP1965 | B.1.1.7 | 4/5/21 | surveillance | 99.1 | 2033 | hCoV-19/USA/PA-VSP1965/2021 | MZ156754 |
| VSP1966 | B.1.526 | 4/5/21 | surveillance | 96.7 | 1787 | hCoV-19/USA/PA-VSP1966/2021 | MZ156755 |
| VSP1969 | B.1.429 | 4/5/21 | surveillance | 99.7 | 3735 | hCoV-19/USA/PA-VSP1969/2021 | OK245985 |
| VSP1970 | B.1.596 | 4/5/21 | surveillance | 99.7 | 2936 | hCoV-19/USA/PA-VSP1970/2021 | OK245986 |
| VSP1971 | B.1.526 | 4/5/21 | surveillance | 99.7 | 3087 | hCoV-19/USA/PA-VSP1971/2021 | OK245987 |
| VSP1972 | B.1.526 | 4/5/21 | surveillance | 99.7 | 3054 | hCoV-19/USA/PA-VSP1972/2021 | OK245988 |
| VSP1980 | None | 4/10/21 | hospitalized | 99.7 | 2810 | hCoV-19/USA/PA-VSP1980/2021 | OK245989 |
| VSP1983 | B.1.1.7 | 4/12/21 | hospitalized | 99.7 | 1772 | hCoV-19/USA/PA-VSP1983/2021 | OK245990 |
| VSP1989 | B.1.1.7 | 4/16/21 | hospitalized | 99.7 | 3363 | hCoV-19/USA/PA-VSP1989/2021 | OK245991 |
| VSP1996 | B.1.1.7 | 4/19/21 | hospitalized | 95.4 | 76 | hCoV-19/USA/PA-VSP1996/2021 | OK245992 |
| VSP1999 | B.1.1.7 | 4/12/21 | accine breakthroug | 99.7 | 3732 | hCoV-19/USA/PA-VSP1999/2021 | OK245993 |
| VSP2000 | B.1.1.7 | 4/12/21 | accine breakthroug | 99.7 | 3000 | hCoV-19/USA/PA-VSP2000/2021 | OK245994 |
| VSP2003 | B.1.575 | 4/12/21 | surveillance | 99.7 | 3829 | hCoV-19/USA/PA-VSP2003/2021 | OK245997 |
| VSP2004 | B.1.526 | 4/12/21 | surveillance | 99.7 | 2955 | hCoV-19/USA/PA-VSP2004/2021 | OK245998 |
| VSP2005 | B.1.2 | 4/12/21 | surveillance | 99.7 | 4067 | hCoV-19/USA/PA-VSP2005/2021 | OK245999 |
| VSP2006 | B.1.1.7 | 4/12/21 | surveillance | 99.7 | 3908 | hCoV-19/USA/PA-VSP2006/2021 | OK246000 |
| VSP2007 | B.1.1.7 | 4/12/21 | surveillance | 99.6 | 2424 | hCoV-19/USA/PA-VSP2007/2021 | OK246001 |
| VSP2008 | B.1.1.7 | 4/12/21 | surveillance | 99.7 | 3711 | hCoV-19/USA/PA-VSP2008/2021 | OK246002 |
| VSP2009 | B.1.1.7 | 4/12/21 | surveillance | 99.7 | 2379 | hCoV-19/USA/PA-VSP2009/2021 | OK246003 |
| VSP2010 | B.1.1.7 | 4/12/21 | surveillance | 99.7 | 2876 | hCoV-19/USA/PA-VSP2010/2021 | OK246004 |
| VSP2011 | B.1.1.7 | 4/12/21 | surveillance | 99.7 | 3273 | hCoV-19/USA/PA-VSP2011/2021 | OK246005 |
| VSP2012 | B.1.637 | 4/12/21 | surveillance | 99.7 | 2920 | hCoV-19/USA/PA-VSP2012/2021 | OK246006 |
| VSP2013 | B.1.526 | 4/12/21 | surveillance | 99.7 | 2846 | hCoV-19/USA/PA-VSP2013/2021 | OK246007 |
| VSP2014 | B.1.1.7 | 4/12/21 | surveillance | 99.7 | 3032 | hCoV-19/USA/PA-VSP2014/2021 | OK246008 |
| VSP2015 | B.1.1.7 | 4/12/21 | surveillance | 99.7 | 3005 | hCoV-19/USA/PA-VSP2015/2021 | OK246009 |
| VSP2016 | B.1.1.7 | 4/12/21 | surveillance | 97.5 | 1666 | hCoV-19/USA/PA-VSP2016/2021 | OK246010 |
| VSP2017 | B.1.1.7 | 4/12/21 | accine breakthroug | 99.7 | 2607 | hCoV-19/USA/PA-VSP2017/2021 | OK246011 |
| VSP2018 | B.1.526 | 4/12/21 | surveillance | 99.7 | 3140 | hCoV-19/USA/PA-VSP2018/2021 | OK246012 |
| VSP2020 | P.1 | 4/12/21 | surveillance | 98.8 | 1741 | hCoV-19/USA/PA-VSP2020/2021 | OK246013 |
| VSP2021 | B.1.1.7 | 4/12/21 | surveillance | 99.1 | 2063 | hCoV-19/USA/PA-VSP2021/2021 | OK246014 |
| VSP2022 | B.1.1.207 | 4/12/21 | surveillance | 99.6 | 1936 | hCoV-19/USA/PA-VSP2022/2021 | OK246015 |
| VSP2023 | B.1.1.7 | 4/12/21 | surveillance | 99.7 | 3510 | hCoV-19/USA/PA-VSP2023/2021 | OK246016 |
| VSP2025 | B.1.1.7 | 4/12/21 | surveillance | 99.7 | 3101 | hCoV-19/USA/PA-VSP2025/2021 | OK246017 |
| VSP2026 | B.1.623 | 4/12/21 | surveillance | 99 | 1983 | hCoV-19/USA/PA-VSP2026/2021 | OK246018 |
| VSP2027 | B.1.525 | 4/12/21 | surveillance | 99.4 | 2225 | hCoV-19/USA/PA-VSP2027/2021 | |
| VSP2028 | P.1 | 4/12/21 | surveillance | 99.7 | 2637 | hCoV-19/USA/PA-VSP2028/2021 | OK246019 |
| VSP2030 | B.1.526 | 4/12/21 | surveillance | 99.7 | 3354 | hCoV-19/USA/PA-VSP2030/2021 | OK246020 |
| VSP2031 | B.1.575 | 4/12/21 | surveillance | 97 | 91 | hCoV-19/USA/PA-VSP2031/2021 | OK246021 |
| VSP2032 | B.1.2 | 4/12/21 | surveillance | 99.7 | 3372 | hCoV-19/USA/PA-VSP2032/2021 | OK246022 |
| VSP2034 | B.1.1.7 | 4/12/21 | surveillance | 99.7 | 3259 | hCoV-19/USA/PA-VSP2034/2021 | OK246023 |
| VSP2035 | B.1.1.7 | 4/12/21 | surveillance | 99.7 | 3360 | hCoV-19/USA/PA-VSP2035/2021 | OK246024 |
| VSP2036 | P.1 | 4/12/21 | surveillance | 99.1 | 714 | hCoV-19/USA/PA-VSP2036/2021 | |

| | | | | | | | |
|---|---|---|---|---|---|---|---|
| VSP2037 | P.1 | 4/12/21 | surveillance | 99.7 | 2812 | hCoV-19/USA/PA-VSP2037/2021 | OK246025 |
| VSP2038 | P.1 | 4/12/21 | surveillance | 99.7 | 3293 | hCoV-19/USA/PA-VSP2038/2021 | OK246026 |
| VSP2039 | B.1.1.7 | 4/12/21 | surveillance | 99.7 | 3352 | hCoV-19/USA/PA-VSP2039/2021 | OK246027 |
| VSP2042 | B.1.526 | 4/12/21 | accine breakthroug | 99.7 | 2970 | hCoV-19/USA/PA-VSP2042/2021 | OK246028 |
| VSP2043 | B.1.526 | 4/12/21 | surveillance | 99.7 | 2574 | hCoV-19/USA/PA-VSP2043/2021 | OK246029 |
| VSP2044 | B.1.1.7 | 4/12/21 | surveillance | 99.6 | 1131 | hCoV-19/USA/PA-VSP2044/2021 | OK246030 |
| VSP2047 | B.1.1.7 | 4/12/21 | surveillance | 99.1 | 728 | hCoV-19/USA/PA-VSP2047/2021 | OK246031 |
| VSP2048 | B.1.2 | 4/12/21 | surveillance | 99.6 | 925 | hCoV-19/USA/PA-VSP2048/2021 | OK246032 |
| VSP2051 | P.1 | 4/12/21 | surveillance | 99.7 | 2854 | hCoV-19/USA/PA-VSP2051/2021 | OK246033 |
| VSP2052 | B.1.575 | 4/12/21 | surveillance | 99.7 | 3284 | hCoV-19/USA/PA-VSP2052/2021 | OK246034 |
| VSP2053 | B.1.1.7 | 4/12/21 | surveillance | 99.6 | 2415 | hCoV-19/USA/PA-VSP2053/2021 | OK246035 |
| VSP2054 | P.1 | 4/12/21 | surveillance | 99.7 | 3443 | hCoV-19/USA/PA-VSP2054/2021 | OK246036 |
| VSP2055 | B.1.1.7 | 4/12/21 | surveillance | 99.7 | 3282 | hCoV-19/USA/PA-VSP2055/2021 | OK246037 |
| VSP2056 | B.1.526 | 4/12/21 | surveillance | 96.2 | 2066 | hCoV-19/USA/PA-VSP2056/2021 | |
| VSP2058 | B.1.1.7 | 4/12/21 | surveillance | 99.6 | 1590 | hCoV-19/USA/PA-VSP2058/2021 | OK246038 |
| VSP2059 | B.1.526 | 4/12/21 | surveillance | 99.7 | 1909 | hCoV-19/USA/PA-VSP2059/2021 | OK246039 |
| VSP2060 | B.1.526 | 4/12/21 | surveillance | 99.7 | 2578 | hCoV-19/USA/PA-VSP2060/2021 | OK246040 |
| VSP2061 | B.1.1.7 | 4/12/21 | surveillance | 99.7 | 2177 | hCoV-19/USA/PA-VSP2061/2021 | OK246041 |
| VSP2062 | B.1.1.7 | 4/12/21 | surveillance | 99.7 | 2719 | hCoV-19/USA/PA-VSP2062/2021 | OK246042 |
| VSP2063 | B.1.1.7 | 4/12/21 | surveillance | 99.7 | 3100 | hCoV-19/USA/PA-VSP2063/2021 | OK246043 |
| VSP2064 | B.1.526 | 4/12/21 | surveillance | 99.7 | 2546 | hCoV-19/USA/PA-VSP2064/2021 | OK246044 |
| VSP2065 | B.1.1.7 | 4/12/21 | surveillance | 99.7 | 2827 | hCoV-19/USA/PA-VSP2065/2021 | OK246045 |
| VSP2066 | B.1.1.7 | 4/12/21 | surveillance | 99.7 | 2010 | hCoV-19/USA/PA-VSP2066/2021 | OK246046 |
| VSP2067 | B.1.1.7 | 4/12/21 | surveillance | 99.7 | 2295 | hCoV-19/USA/PA-VSP2067/2021 | OK246047 |
| VSP2068 | B.1.1.7 | 4/12/21 | surveillance | 99.7 | 2805 | hCoV-19/USA/PA-VSP2068/2021 | OK246048 |
| VSP2069 | B.1.1.7 | 4/12/21 | surveillance | 99.6 | 2797 | hCoV-19/USA/PA-VSP2069/2021 | OK246049 |
| VSP2070 | B.1.1.7 | 4/12/21 | surveillance | 99.7 | 2545 | hCoV-19/USA/PA-VSP2070/2021 | OK246050 |
| VSP2071 | B.1.526 | 4/12/21 | surveillance | 99.7 | 2340 | hCoV-19/USA/PA-VSP2071/2021 | OK246051 |
| VSP2072 | B.1.1.7 | 4/12/21 | surveillance | 99.7 | 2096 | hCoV-19/USA/PA-VSP2072/2021 | OK246052 |
| VSP2073 | B.1.1.7 | 4/12/21 | surveillance | 99.7 | 2790 | hCoV-19/USA/PA-VSP2073/2021 | OK246053 |
| VSP2074 | B.1.1.7 | 4/12/21 | surveillance | 99.1 | 1729 | hCoV-19/USA/PA-VSP2074/2021 | |
| VSP2075 | B.1.1.7 | 4/12/21 | surveillance | 99.7 | 3258 | hCoV-19/USA/PA-VSP2075/2021 | OK246054 |
| VSP2076 | B.1.1.7 | 4/12/21 | surveillance | 99.7 | 2976 | hCoV-19/USA/PA-VSP2076/2021 | OK246055 |
| VSP2077 | B.1.526 | 4/12/21 | surveillance | 99.7 | 2259 | hCoV-19/USA/PA-VSP2077/2021 | OK246056 |
| VSP2078 | B.1.1.7 | 4/12/21 | surveillance | 99.7 | 1858 | hCoV-19/USA/PA-VSP2078/2021 | OK246057 |
| VSP2079 | B.1.526 | 4/12/21 | surveillance | 95.2 | 628 | hCoV-19/USA/PA-VSP2079/2021 | |
| VSP2080 | B.1.637 | 4/12/21 | surveillance | 99.7 | 2489 | hCoV-19/USA/PA-VSP2080/2021 | OK246058 |
| VSP2081 | B.1.1.7 | 4/12/21 | surveillance | 99.7 | 3282 | hCoV-19/USA/PA-VSP2081/2021 | OK246059 |
| VSP2082 | P.1.2 | 4/12/21 | surveillance | 99.7 | 2375 | hCoV-19/USA/PA-VSP2082/2021 | OK246060 |
| VSP2083 | B.1.1.7 | 4/12/21 | surveillance | 99.7 | 2634 | hCoV-19/USA/PA-VSP2083/2021 | OK246061 |
| VSP2084 | B.1.1.7 | 4/12/21 | surveillance | 99.7 | 2901 | hCoV-19/USA/PA-VSP2084/2021 | OK246062 |
| VSP2085 | B.1.1.7 | 4/12/21 | surveillance | 99.4 | 1128 | hCoV-19/USA/PA-VSP2085/2021 | |
| VSP2086 | B.1.1.7 | 4/12/21 | surveillance | 99.6 | 1457 | hCoV-19/USA/PA-VSP2086/2021 | OK246063 |
| VSP2087 | B.1.1 | 4/12/21 | surveillance | 97.5 | 511 | hCoV-19/USA/PA-VSP2087/2021 | OK246064 |
| VSP2088 | B.1.1.7 | 4/12/21 | surveillance | 99.7 | 528 | hCoV-19/USA/PA-VSP2088/2021 | OK246065 |
| VSP2089 | B.1.1.7 | 4/12/21 | surveillance | 99.6 | 771 | hCoV-19/USA/PA-VSP2089/2021 | OK246066 |
| VSP2090 | B.1.1.7 | 4/12/21 | surveillance | 99.6 | 1754 | hCoV-19/USA/PA-VSP2090/2021 | OK246067 |

| | | | | | | | |
|---|---|---|---|---|---|---|---|
| VSP2091 | P.1.10 | 4/12/21 | surveillance | 98.3 | 236 | hCoV-19/USA/PA-VSP2091/2021 | OK246068 |
| VSP2092 | B.1.1.7 | 4/12/21 | surveillance | 99.6 | 1025 | hCoV-19/USA/PA-VSP2092/2021 | OK246069 |
| VSP2093 | B.1.1.7 | 4/12/21 | surveillance | 99.7 | 1677 | hCoV-19/USA/PA-VSP2093/2021 | OK246070 |
| VSP2094 | B.1.1.7 | 4/12/21 | surveillance | 99.7 | 652 | hCoV-19/USA/PA-VSP2094/2021 | OK246071 |
| VSP2096 | B.1.1.7 | 4/12/21 | surveillance | 99.7 | 1160 | hCoV-19/USA/PA-VSP2096/2021 | OK246072 |
| VSP2097 | R.1 | 4/12/21 | surveillance | 99.6 | 1459 | hCoV-19/USA/PA-VSP2097/2021 | OK246073 |
| VSP2098 | None | 4/12/21 | surveillance | 96.4 | 1046 | hCoV-19/USA/PA-VSP2098/2021 | OK246074 |
| VSP2099 | B.1.1.7 | 4/12/21 | surveillance | 99.7 | 1725 | hCoV-19/USA/PA-VSP2099/2021 | OK246075 |
| VSP2100 | B.1.1.7 | 4/12/21 | surveillance | 97.8 | 227 | hCoV-19/USA/PA-VSP2100/2021 | OK246076 |
| VSP2101 | B.1.526 | 4/12/21 | surveillance | 98.8 | 655 | hCoV-19/USA/PA-VSP2101/2021 | OK246077 |
| VSP2102 | B.1.1.7 | 4/12/21 | surveillance | 99.7 | 743 | hCoV-19/USA/DE-VSP2102/2021 | OK246078 |
| VSP2103 | B.1.1.7 | 4/12/21 | surveillance | 97.2 | 111 | hCoV-19/USA/PA-VSP2103/2021 | OK246079 |
| VSP2104 | B.1.526 | 4/12/21 | surveillance | 99.7 | 2041 | hCoV-19/USA/PA-VSP2104/2021 | OK246080 |
| VSP2105 | B.1.1.7 | 4/12/21 | surveillance | 99.7 | 1485 | hCoV-19/USA/PA-VSP2105/2021 | OK246081 |
| VSP2106 | B.1.1.7 | 4/12/21 | surveillance | 99.1 | 915 | hCoV-19/USA/PA-VSP2106/2021 | OK246082 |
| VSP2107 | B.1.1.7 | 4/12/21 | surveillance | 99.6 | 1010 | hCoV-19/USA/PA-VSP2107/2021 | OK246083 |
| VSP2108 | B.1.1.7 | 4/12/21 | surveillance | 99.6 | 1825 | hCoV-19/USA/PA-VSP2108/2021 | OK246084 |
| VSP2109 | B.1.526 | 4/12/21 | surveillance | 99.7 | 1640 | hCoV-19/USA/PA-VSP2109/2021 | OK246085 |
| VSP2110 | B.1.1.7 | 4/12/21 | surveillance | 99.7 | 1602 | hCoV-19/USA/PA-VSP2110/2021 | OK246086 |
| VSP2111 | B.1.1.7 | 4/12/21 | surveillance | 99.7 | 964 | hCoV-19/USA/PA-VSP2111/2021 | OK246087 |
| VSP2112 | B.1.1.7 | 4/12/21 | surveillance | 99.5 | 481 | hCoV-19/USA/PA-VSP2112/2021 | OK246088 |
| VSP2113 | B.1.1.7 | 4/12/21 | surveillance | 99.7 | 2025 | hCoV-19/USA/PA-VSP2113/2021 | OK246089 |
| VSP2114 | B.1.1.7 | 4/12/21 | surveillance | 97.1 | 119 | hCoV-19/USA/PA-VSP2114/2021 | OK246090 |
| VSP2115 | B.1.1.7 | 4/12/21 | surveillance | 99.6 | 1581 | hCoV-19/USA/PA-VSP2115/2021 | OK246091 |
| VSP2116 | B.1.1.7 | 4/12/21 | surveillance | 99.1 | 1624 | hCoV-19/USA/PA-VSP2116/2021 | OK246092 |
| VSP2117 | B.1.1.7 | 4/12/21 | surveillance | 99.7 | 1684 | hCoV-19/USA/NJ-VSP2117/2021 | OK246093 |
| VSP2118 | B.1.1.7 | 4/12/21 | surveillance | 99.3 | 1045 | hCoV-19/USA/PA-VSP2118/2021 | OK246094 |
| VSP2119 | B.1.1.7 | 4/12/21 | surveillance | 99.1 | 631 | hCoV-19/USA/PA-VSP2119/2021 | OK246095 |
| VSP2120 | B.1.1.7 | 4/12/21 | surveillance | 98.9 | 676 | hCoV-19/USA/PA-VSP2120/2021 | OK246096 |
| VSP2121 | B.1.1.7 | 4/12/21 | surveillance | 99.2 | 1754 | hCoV-19/USA/PA-VSP2121/2021 | OK246097 |
| VSP2122 | B.1.526 | 4/12/21 | surveillance | 98.6 | 829 | hCoV-19/USA/PA-VSP2122/2021 | OK246098 |
| VSP2123 | B.1.1.7 | 4/12/21 | surveillance | 99.7 | 1355 | hCoV-19/USA/PA-VSP2123/2021 | OK246099 |
| VSP2124 | B.1.1.7 | 4/12/21 | surveillance | 99.6 | 1147 | hCoV-19/USA/PA-VSP2124/2021 | OK246100 |
| VSP2125 | B.1.526 | 4/12/21 | surveillance | 99.7 | 980 | hCoV-19/USA/PA-VSP2125/2021 | OK246101 |
| VSP2126 | B.1.1.7 | 4/12/21 | surveillance | 99.1 | 1308 | hCoV-19/USA/PA-VSP2126/2021 | OK246102 |
| VSP2127 | B.1.526 | 4/12/21 | surveillance | 99.1 | 501 | hCoV-19/USA/PR-VSP2127/2021 | OK246103 |
| VSP2128 | B.1.1.7 | 4/12/21 | surveillance | 99.6 | 2075 | hCoV-19/USA/PA-VSP2128/2021 | OK246104 |
| VSP2129 | B.1.1.7 | 4/12/21 | surveillance | 99.1 | 1169 | hCoV-19/USA/PA-VSP2129/2021 | OK246105 |
| VSP2130 | B.1.1.7 | 4/12/21 | surveillance | 97.4 | 101 | hCoV-19/USA/PA-VSP2130/2021 | OK246106 |
| VSP2131 | B.1.1.7 | 4/12/21 | surveillance | 97.3 | 411 | hCoV-19/USA/PA-VSP2131/2021 | OK246107 |
| VSP2132 | B.1.1.7 | 4/12/21 | surveillance | 99.6 | 887 | hCoV-19/USA/PA-VSP2132/2021 | OK246108 |
| VSP2133 | B.1.1.7 | 4/12/21 | surveillance | 99.1 | 651 | hCoV-19/USA/PA-VSP2133/2021 | |
| VSP2134 | B.1.1.7 | 4/12/21 | surveillance | 99.7 | 800 | hCoV-19/USA/PA-VSP2134/2021 | OK246109 |
| VSP2135 | B.1.1.7 | 4/12/21 | surveillance | 99.2 | 1025 | hCoV-19/USA/PA-VSP2135/2021 | OK246110 |
| VSP2136 | B.1.1.7 | 4/12/21 | surveillance | 99.7 | 787 | hCoV-19/USA/PA-VSP2136/2021 | OK246111 |
| VSP2137 | B.1.575 | 4/12/21 | surveillance | 99.6 | 1992 | hCoV-19/USA/PA-VSP2137/2021 | OK246112 |
| VSP2138 | B.1.1.7 | 4/12/21 | surveillance | 99.5 | 413 | hCoV-19/USA/PA-VSP2138/2021 | OK246113 |

| | | | | | | | |
|---|---|---|---|---|---|---|---|
| VSP2139 | B.1.1.7 | 4/12/21 | surveillance | 99.1 | 382 | hCoV-19/USA/PA-VSP2139/2021 | OK246114 |
| VSP2141 | B.1.525 | 4/12/21 | surveillance | 99.4 | 1493 | hCoV-19/USA/PA-VSP2141/2021 | |
| VSP2142 | B.1.1.7 | 4/12/21 | surveillance | 99.6 | 839 | hCoV-19/USA/PA-VSP2142/2021 | OK246115 |
| VSP2143 | B.1.1.7 | 4/12/21 | surveillance | 99.7 | 532 | hCoV-19/USA/PA-VSP2143/2021 | OK246116 |
| VSP2144 | B.1.1.7 | 4/12/21 | surveillance | 96.8 | 113 | hCoV-19/USA/PA-VSP2144/2021 | OK246117 |
| VSP2145 | B.1.1.7 | 4/12/21 | surveillance | 95.5 | 113 | hCoV-19/USA/PA-VSP2145/2021 | OK246118 |
| VSP2146 | B.1.1.7 | 4/12/21 | surveillance | 99.6 | 421 | hCoV-19/USA/PA-VSP2146/2021 | OK246119 |
| VSP2147 | B.1.1.7 | 4/12/21 | surveillance | 99.7 | 793 | hCoV-19/USA/PA-VSP2147/2021 | OK246120 |
| VSP2148 | B.1.1.7 | 4/12/21 | surveillance | 99.2 | 473 | hCoV-19/USA/PA-VSP2148/2021 | OK246121 |
| VSP2149 | B.1 | 4/12/21 | surveillance | 99.4 | 1812 | hCoV-19/USA/PA-VSP2149/2021 | OK246122 |
| VSP2151 | B.1.1.7 | 4/12/21 | surveillance | 99.1 | 903 | hCoV-19/USA/PA-VSP2151/2021 | OK246123 |
| VSP2152 | B.1.1.7 | 4/12/21 | surveillance | 99.4 | 991 | hCoV-19/USA/NJ-VSP2152/2021 | OK246124 |
| VSP2153 | B.1.1.7 | 4/12/21 | surveillance | 99.7 | 1833 | hCoV-19/USA/PA-VSP2153/2021 | OK246125 |
| VSP2154 | B.1.525 | 4/12/21 | surveillance | 99.5 | 3829 | hCoV-19/USA/PA-VSP2154/2021 | |
| VSP2155 | B.1.1.7 | 4/12/21 | surveillance | 99.7 | 2925 | hCoV-19/USA/NJ-VSP2155/2021 | OK246126 |
| VSP2156 | B.1.1.7 | 4/12/21 | surveillance | 99.7 | 4517 | hCoV-19/USA/PA-VSP2156/2021 | OK246127 |
| VSP2157 | B.1.1.7 | 4/12/21 | surveillance | 99.7 | 14443 | hCoV-19/USA/PA-VSP2157/2021 | OK246128 |
| VSP2158 | B.1.1.7 | 4/12/21 | surveillance | 99.7 | 6029 | hCoV-19/USA/PA-VSP2158/2021 | OK246129 |
| VSP2159 | B.1.1.7 | 4/12/21 | surveillance | 99.7 | 4527 | hCoV-19/USA/NJ-VSP2159/2021 | OK246130 |
| VSP2160 | B.1.1.7 | 4/12/21 | surveillance | 99.7 | 2772 | hCoV-19/USA/PA-VSP2160/2021 | OK246131 |
| VSP2161 | B.1.2 | 4/12/21 | surveillance | 99.5 | 2221 | hCoV-19/USA/PA-VSP2161/2021 | OK246132 |
| VSP2162 | B.1.588 | 4/12/21 | surveillance | 99.6 | 3549 | hCoV-19/USA/NJ-VSP2162/2021 | OK246133 |
| VSP2163 | B.1.2 | 4/12/21 | surveillance | 99.7 | 3195 | hCoV-19/USA/PA-VSP2163/2021 | OK246134 |
| VSP2164 | B.1.526 | 4/12/21 | surveillance | 99.1 | 1561 | hCoV-19/USA/PA-VSP2164/2021 | OK246135 |
| VSP2170 | B.1.526 | 4/12/21 | surveillance | 99.7 | 3769 | hCoV-19/USA/PA-VSP2170/2021 | OK246136 |
| VSP2171 | B.1.1.7 | 4/12/21 | surveillance | 99.7 | 3081 | hCoV-19/USA/PA-VSP2171/2021 | OK246137 |
| VSP2172 | B.1.1.7 | 4/12/21 | surveillance | 98.8 | 2994 | hCoV-19/USA/PA-VSP2172/2021 | OK246138 |
| VSP2174 | B.1.1.7 | 4/12/21 | surveillance | 99.2 | 954 | hCoV-19/USA/PA-VSP2174/2021 | OK246139 |
| VSP2175 | B.1.1.7 | 4/12/21 | surveillance | 99.5 | 1053 | hCoV-19/USA/PA-VSP2175/2021 | OK246140 |
| VSP2176 | B.1.1.7 | 4/12/21 | surveillance | 99.7 | 1442 | hCoV-19/USA/PA-VSP2176/2021 | OK246141 |
| VSP2177 | B.1.1.7 | 4/12/21 | surveillance | 99.6 | 471 | hCoV-19/USA/PA-VSP2177/2021 | OK246142 |
| VSP2178 | B.1.1.7 | 4/12/21 | surveillance | 99.3 | 1009 | hCoV-19/USA/PA-VSP2178/2021 | OK246143 |
| VSP2179 | B.1.1.7 | 4/12/21 | surveillance | 99.7 | 1270 | hCoV-19/USA/PA-VSP2179/2021 | OK246144 |
| VSP2180 | B.1.1.7 | 4/12/21 | surveillance | 99.2 | 815 | hCoV-19/USA/NJ-VSP2180/2021 | OK246145 |
| VSP2181 | B.1.1.7 | 4/12/21 | surveillance | 99.6 | 750 | hCoV-19/USA/NJ-VSP2181/2021 | OK246146 |
| VSP2183 | B.1.1.7 | 4/12/21 | surveillance | 99.1 | 2446 | hCoV-19/USA/NJ-VSP2183/2021 | OK246147 |
| VSP2184 | B.1.526 | 4/12/21 | surveillance | 98.3 | 2463 | hCoV-19/USA/NJ-VSP2184/2021 | OK246148 |
| VSP2185 | B.1.1.7 | 4/12/21 | surveillance | 98.9 | 4190 | hCoV-19/USA/NJ-VSP2185/2021 | OK246149 |
| VSP2186 | B.1.526 | 4/12/21 | surveillance | 99.7 | 3496 | hCoV-19/USA/NJ-VSP2186/2021 | OK246150 |
| VSP2187 | B.1.526 | 4/12/21 | surveillance | 99.3 | 2411 | hCoV-19/USA/NJ-VSP2187/2021 | OK246151 |
| VSP2189 | B.1.1.7 | 4/12/21 | surveillance | 99.1 | 1880 | hCoV-19/USA/PA-VSP2189/2021 | OK246152 |
| VSP2190 | B.1.1.7 | 4/12/21 | surveillance | 99.7 | 3485 | hCoV-19/USA/PA-VSP2190/2021 | OK246153 |
| VSP2191 | B.1.1.7 | 4/12/21 | surveillance | 99.7 | 1944 | hCoV-19/USA/PA-VSP2191/2021 | OK246154 |
| VSP2192 | B.1.1.7 | 4/12/21 | surveillance | 99.3 | 2430 | hCoV-19/USA/PA-VSP2192/2021 | OK246155 |
| VSP2193 | B.1.2 | 4/12/21 | surveillance | 99.7 | 1653 | hCoV-19/USA/PA-VSP2193/2021 | OK246156 |
| VSP2194 | B.1.1.7 | 4/12/21 | surveillance | 99.1 | 3767 | hCoV-19/USA/PA-VSP2194/2021 | OK246157 |
| VSP2197 | B.1.1.7 | 4/12/21 | surveillance | 99.2 | 2481 | hCoV-19/USA/PA-VSP2197/2021 | OK246158 |

| | | | | | | | |
|---|---|---|---|---|---|---|---|
| VSP2198 | B.1.1.7 | 4/12/21 | surveillance | 97.6 | 77 | hCoV-19/USA/PA-VSP2198/2021 | OK246159 |
| VSP2199 | B.1.1.7 | 4/12/21 | surveillance | 99.5 | 1900 | hCoV-19/USA/PA-VSP2199/2021 | OK246160 |
| VSP2200 | B.1.1.7 | 4/12/21 | surveillance | 99.7 | 3319 | hCoV-19/USA/PA-VSP2200/2021 | OK246161 |
| VSP2201 | B.1.1.7 | 4/12/21 | surveillance | 99.7 | 2908 | hCoV-19/USA/PA-VSP2201/2021 | OK246162 |
| VSP2202 | B.1.1.7 | 4/12/21 | surveillance | 99.7 | 2576 | hCoV-19/USA/PA-VSP2202/2021 | OK246163 |
| VSP2204 | B.1.429 | 4/12/21 | surveillance | 98.6 | 703 | hCoV-19/USA/PA-VSP2204/2021 | OK246164 |
| VSP2205 | B.1.526 | 4/12/21 | surveillance | 99.7 | 1865 | hCoV-19/USA/PA-VSP2205/2021 | OK246165 |
| VSP2206 | B.1.526 | 4/19/21 | surveillance | 99.7 | 7652 | hCoV-19/USA/VSP2206/2021 | MZ512791 |
| VSP2207 | B.1.1.7 | 4/19/21 | surveillance | 99.7 | 9090 | hCoV-19/USA/VSP2207/2021 | MZ512792 |
| VSP2208 | B.1.637 | 4/19/21 | surveillance | 99.7 | 7216 | hCoV-19/USA/VSP2208/2021 | MZ512793 |
| VSP2209 | B.1.1.7 | 4/19/21 | surveillance | 99.7 | 7928 | hCoV-19/USA/VSP2209/2021 | MZ512794 |
| VSP2210 | B.1.1.7 | 4/19/21 | surveillance | 99.7 | 6937 | hCoV-19/USA/VSP2210/2021 | MZ512795 |
| VSP2211 | B.1.1.7 | 4/19/21 | surveillance | 99.7 | 7423 | hCoV-19/USA/VSP2211/2021 | MZ512796 |
| VSP2212 | B.1.1.7 | 4/19/21 | surveillance | 99.7 | 3145 | hCoV-19/USA/VSP2212/2021 | MZ512797 |
| VSP2213 | Q.4 | 4/19/21 | surveillance | 99.7 | 6143 | hCoV-19/USA/VSP2213/2021 | MZ512798 |
| VSP2214 | B.1.1.7 | 4/19/21 | surveillance | 99.7 | 5949 | hCoV-19/USA/VSP2214/2021 | MZ512799 |
| VSP2215 | B.1.1.7 | 4/19/21 | surveillance | 99.7 | 7112 | hCoV-19/USA/VSP2215/2021 | MZ512800 |
| VSP2216 | B.1.1.7 | 4/19/21 | surveillance | 99.7 | 6492 | hCoV-19/USA/VSP2216/2021 | MZ512801 |
| VSP2218 | B.1.1.7 | 4/19/21 | surveillance | 99.7 | 7393 | hCoV-19/USA/VSP2218/2021 | MZ512802 |
| VSP2219 | B.1.1.7 | 4/19/21 | surveillance | 99.7 | 6484 | hCoV-19/USA/VSP2219/2021 | MZ512803 |
| VSP2220 | B.1.637 | 4/19/21 | surveillance | 99.7 | 7029 | hCoV-19/USA/VSP2220/2021 | MZ512804 |
| VSP2221 | B.1.1.7 | 4/19/21 | surveillance | 97.4 | 4786 | hCoV-19/USA/VSP2221/2021 | MZ512805 |
| VSP2222 | B.1.1.7 | 4/19/21 | surveillance | 99.7 | 5455 | hCoV-19/USA/VSP2222/2021 | MZ512806 |
| VSP2224 | B.1.637 | 4/19/21 | surveillance | 99.7 | 5596 | hCoV-19/USA/VSP2224/2021 | MZ512807 |
| VSP2225 | B.1.1.7 | 4/19/21 | surveillance | 99.7 | 6329 | hCoV-19/USA/VSP2225/2021 | MZ512808 |
| VSP2226 | B.1.1.7 | 4/19/21 | surveillance | 99.7 | 6796 | hCoV-19/USA/VSP2226/2021 | MZ512809 |
| VSP2227 | B.1.1.7 | 4/19/21 | surveillance | 97.5 | 4911 | hCoV-19/USA/VSP2227/2021 | |
| VSP2228 | P.1.2 | 4/19/21 | surveillance | 99.7 | 6531 | hCoV-19/USA/VSP2228/2021 | MZ512810 |
| VSP2229 | B.1.637 | 4/19/21 | surveillance | 99.7 | 7292 | hCoV-19/USA/VSP2229/2021 | MZ512811 |
| VSP2230 | B.1.1.7 | 4/19/21 | surveillance | 99.7 | 7355 | hCoV-19/USA/VSP2230/2021 | MZ512812 |
| VSP2231 | B.1.526 | 4/19/21 | surveillance | 99.7 | 7203 | hCoV-19/USA/VSP2231/2021 | MZ512813 |
| VSP2232 | B.1.1.7 | 4/19/21 | surveillance | 99.7 | 6712 | hCoV-19/USA/VSP2232/2021 | MZ512814 |
| VSP2233 | B.1.1.7 | 4/19/21 | surveillance | 99.7 | 5618 | hCoV-19/USA/VSP2233/2021 | MZ512815 |
| VSP2234 | P.1 | 4/19/21 | surveillance | 99.7 | 6536 | hCoV-19/USA/VSP2234/2021 | MZ512816 |
| VSP2235 | B.1.1.7 | 4/19/21 | surveillance | 99.7 | 6137 | hCoV-19/USA/VSP2235/2021 | MZ512817 |
| VSP2236 | B.1.1.7 | 4/19/21 | surveillance | 98.6 | 3717 | hCoV-19/USA/VSP2236/2021 | MZ512818 |
| VSP2237 | B.1.1.7 | 4/19/21 | surveillance | 99.7 | 5462 | hCoV-19/USA/VSP2237/2021 | MZ512819 |
| VSP2238 | B.1.1.7 | 4/19/21 | surveillance | 99.7 | 6546 | hCoV-19/USA/VSP2238/2021 | MZ512820 |
| VSP2239 | B.1.1.7 | 4/19/21 | surveillance | 99.7 | 6329 | hCoV-19/USA/VSP2239/2021 | MZ512821 |
| VSP2240 | B.1.1.7 | 4/19/21 | surveillance | 99.7 | 6830 | hCoV-19/USA/VSP2240/2021 | MZ512822 |
| VSP2241 | B.1.1.7 | 4/19/21 | surveillance | 99.7 | 6732 | hCoV-19/USA/VSP2241/2021 | MZ512823 |
| VSP2242 | B.1.1.7 | 4/19/21 | surveillance | 99.7 | 4980 | hCoV-19/USA/VSP2242/2021 | MZ512824 |
| VSP2243 | B.1.1.7 | 4/19/21 | surveillance | 99.7 | 6518 | hCoV-19/USA/VSP2243/2021 | MZ512825 |
| VSP2244 | B.1.1.7 | 4/19/21 | surveillance | 99.7 | 5222 | hCoV-19/USA/VSP2244/2021 | MZ512826 |
| VSP2245 | B.1.1.7 | 4/19/21 | surveillance | 99.7 | 4921 | hCoV-19/USA/VSP2245/2021 | MZ512827 |
| VSP2246 | P.1 | 4/19/21 | surveillance | 99.7 | 6082 | hCoV-19/USA/VSP2246/2021 | MZ512828 |
| VSP2247 | B.1.1.7 | 4/19/21 | surveillance | 99.1 | 545 | hCoV-19/USA/VSP2247/2021 | MZ512829 |

| | | | | | | | |
|---|---|---|---|---|---|---|---|
| VSP2248 | B.1.1.7 | 4/19/21 | surveillance | 99.7 | 5385 | hCoV-19/USA/VSP2248/2021 | MZ512830 |
| VSP2249 | B.1.1.7 | 4/19/21 | surveillance | 97.4 | 4965 | hCoV-19/USA/VSP2249/2021 | MZ512831 |
| VSP2250 | B.1.1.7 | 4/19/21 | surveillance | 99.7 | 4468 | hCoV-19/USA/VSP2250/2021 | MZ512832 |
| VSP2251 | B.1.1.7 | 4/19/21 | surveillance | 99.7 | 6502 | hCoV-19/USA/VSP2251/2021 | MZ512833 |
| VSP2252 | B.1.1.7 | 4/19/21 | surveillance | 99.7 | 6001 | hCoV-19/USA/VSP2252/2021 | MZ512834 |
| VSP2253 | B.1.526 | 4/19/21 | surveillance | 99.7 | 5949 | hCoV-19/USA/VSP2253/2021 | MZ512835 |
| VSP2254 | B.1.1.7 | 4/19/21 | surveillance | 99.7 | 6077 | hCoV-19/USA/VSP2254/2021 | MZ512836 |
| VSP2255 | B.1.526 | 4/19/21 | surveillance | 99.7 | 6125 | hCoV-19/USA/VSP2255/2021 | MZ512837 |
| VSP2256 | B.1.1.7 | 4/19/21 | surveillance | 97.2 | 2192 | hCoV-19/USA/VSP2256/2021 | MZ512838 |
| VSP2258 | B.1.1.7 | 4/19/21 | surveillance | 99.7 | 8045 | hCoV-19/USA/VSP2258/2021 | MZ512839 |
| VSP2259 | B.1.1.7 | 4/19/21 | surveillance | 99.7 | 6079 | hCoV-19/USA/VSP2259/2021 | MZ512840 |
| VSP2260 | B.1.1.7 | 4/19/21 | surveillance | 98.3 | 3821 | hCoV-19/USA/VSP2260/2021 | MZ512841 |
| VSP2261 | B.1.1.7 | 4/19/21 | surveillance | 99.7 | 4299 | hCoV-19/USA/VSP2261/2021 | MZ512842 |
| VSP2262 | B.1.1.7 | 4/19/21 | surveillance | 99.7 | 6387 | hCoV-19/USA/VSP2262/2021 | MZ512843 |
| VSP2263 | B.1.1.7 | 4/19/21 | surveillance | 99.7 | 6225 | hCoV-19/USA/VSP2263/2021 | MZ512844 |
| VSP2264 | B.1.1.7 | 4/19/21 | surveillance | 99.7 | 6072 | hCoV-19/USA/VSP2264/2021 | MZ512845 |
| VSP2265 | P.1 | 4/19/21 | surveillance | 99.7 | 6527 | hCoV-19/USA/VSP2265/2021 | MZ512846 |
| VSP2266 | B.1.1.7 | 4/19/21 | surveillance | 99.1 | 2705 | hCoV-19/USA/VSP2266/2021 | MZ512847 |
| VSP2267 | B.1.1.7 | 4/19/21 | surveillance | 99.7 | 5191 | hCoV-19/USA/VSP2267/2021 | MZ512848 |
| VSP2268 | B.1.1.7 | 4/19/21 | surveillance | 99.7 | 6064 | hCoV-19/USA/VSP2268/2021 | MZ512849 |
| VSP2269 | B.1.1.7 | 4/19/21 | surveillance | 99.7 | 6007 | hCoV-19/USA/VSP2269/2021 | MZ512850 |
| VSP2271 | P.1 | 4/19/21 | surveillance | 99.7 | 5026 | hCoV-19/USA/VSP2271/2021 | MZ512851 |
| VSP2272 | B.1.1.7 | 4/19/21 | surveillance | 99.7 | 5937 | hCoV-19/USA/VSP2272/2021 | MZ512852 |
| VSP2273 | B.1.234 | 4/19/21 | surveillance | 99.7 | 6278 | hCoV-19/USA/VSP2273/2021 | MZ512853 |
| VSP2274 | B.1.526 | 4/19/21 | surveillance | 99.7 | 6203 | hCoV-19/USA/VSP2274/2021 | MZ512854 |
| VSP2275 | B.1.1.519 | 4/19/21 | surveillance | 99.1 | 2083 | hCoV-19/USA/VSP2275/2021 | MZ512855 |
| VSP2276 | B.1.1.7 | 4/19/21 | surveillance | 99.7 | 6900 | hCoV-19/USA/VSP2276/2021 | MZ512856 |
| VSP2277 | B.1.1.7 | 4/19/21 | surveillance | 99.7 | 5162 | hCoV-19/USA/VSP2277/2021 | MZ512857 |
| VSP2278 | B.1.1.7 | 4/19/21 | surveillance | 99.7 | 6188 | hCoV-19/USA/VSP2278/2021 | MZ512858 |
| VSP2279 | R.1 | 4/19/21 | surveillance | 99.7 | 5900 | hCoV-19/USA/VSP2279/2021 | MZ512859 |
| VSP2280 | B.1.1.7 | 4/19/21 | surveillance | 99.7 | 5935 | hCoV-19/USA/VSP2280/2021 | MZ512860 |
| VSP2281 | B.1.526 | 4/19/21 | surveillance | 99.1 | 5078 | hCoV-19/USA/VSP2281/2021 | MZ512861 |
| VSP2282 | B.1.1.7 | 4/19/21 | surveillance | 99.7 | 6546 | hCoV-19/USA/VSP2282/2021 | MZ512862 |
| VSP2283 | B.1.1.7 | 4/19/21 | surveillance | 99.7 | 5538 | hCoV-19/USA/VSP2283/2021 | MZ512863 |
| VSP2284 | R.1 | 4/19/21 | surveillance | 99.7 | 5794 | hCoV-19/USA/VSP2284/2021 | MZ512864 |
| VSP2285 | B.1.1.7 | 4/19/21 | surveillance | 99.7 | 6472 | hCoV-19/USA/VSP2285/2021 | MZ512865 |
| VSP2286 | B.1.1.7 | 4/19/21 | surveillance | 99.7 | 5237 | hCoV-19/USA/VSP2286/2021 | MZ512866 |
| VSP2287 | B.1.351 | 4/19/21 | surveillance | 99.1 | 5049 | hCoV-19/USA/VSP2287/2021 | MZ512867 |
| VSP2288 | B.1.1.7 | 4/19/21 | surveillance | 99.7 | 6716 | hCoV-19/USA/VSP2288/2021 | MZ512868 |
| VSP2289 | B.1.1.7 | 4/19/21 | surveillance | 99.7 | 4803 | hCoV-19/USA/VSP2289/2021 | MZ512869 |
| VSP2290 | B.1.1.7 | 4/19/21 | surveillance | 99.7 | 6659 | hCoV-19/USA/VSP2290/2021 | MZ512870 |
| VSP2291 | B.1.1.7 | 4/19/21 | surveillance | 99.7 | 5873 | hCoV-19/USA/VSP2291/2021 | MZ512871 |
| VSP2292 | B.1.1.7 | 4/19/21 | surveillance | 99.7 | 6066 | hCoV-19/USA/VSP2292/2021 | MZ512872 |
| VSP2293 | B.1.526 | 4/19/21 | surveillance | 99.7 | 6213 | hCoV-19/USA/VSP2293/2021 | MZ512873 |
| VSP2294 | B.1.1.7 | 4/19/21 | surveillance | 99.7 | 3638 | hCoV-19/USA/VSP2294/2021 | MZ512874 |
| VSP2295 | B.1.1.7 | 4/19/21 | surveillance | 99.7 | 5836 | hCoV-19/USA/VSP2295/2021 | MZ512875 |
| VSP2296 | B.1.1.7 | 4/19/21 | surveillance | 99.7 | 1960 | hCoV-19/USA/PA-VSP2296/2021 | OK246166 |

| | | | | | | | |
|---|---|---|---|---|---|---|---|
| VSP2297 | B.1.526 | 4/19/21 | surveillance | 99.7 | 2037 | hCoV-19/USA/NY-VSP2297/2021 | OK246167 |
| VSP2298 | B.1.1.7 | 4/19/21 | surveillance | 99.7 | 2799 | hCoV-19/USA/PA-VSP2298/2021 | OK246168 |
| VSP2299 | B.1.351 | 4/19/21 | surveillance | 99.1 | 1991 | hCoV-19/USA/PA-VSP2299/2021 | OK246169 |
| VSP2300 | B.1.1.7 | 4/19/21 | surveillance | 99.7 | 3401 | hCoV-19/USA/PA-VSP2300/2021 | OK246170 |
| VSP2302 | B.1.1.7 | 4/19/21 | surveillance | 99.7 | 2733 | hCoV-19/USA/PA-VSP2302/2021 | OK246171 |
| VSP2303 | B.1.526 | 4/19/21 | surveillance | 99.7 | 2417 | hCoV-19/USA/PA-VSP2303/2021 | OK246172 |
| VSP2304 | B.1.1.7 | 4/19/21 | surveillance | 99.7 | 2898 | hCoV-19/USA/PA-VSP2304/2021 | OK246173 |
| VSP2306 | B.1.526 | 4/19/21 | surveillance | 99.7 | 2909 | hCoV-19/USA/PA-VSP2306/2021 | OK246174 |
| VSP2307 | B.1.526 | 4/19/21 | surveillance | 99.7 | 3784 | hCoV-19/USA/PA-VSP2307/2021 | OK246175 |
| VSP2308 | P.1 | 4/19/21 | surveillance | 99.7 | 2913 | hCoV-19/USA/PA-VSP2308/2021 | OK246176 |
| VSP2309 | P.1 | 4/19/21 | surveillance | 99.3 | 2628 | hCoV-19/USA/PA-VSP2309/2021 | OK246177 |
| VSP2310 | B.1.1.7 | 4/19/21 | surveillance | 99.3 | 670 | hCoV-19/USA/PA-VSP2310/2021 | OK246178 |
| VSP2311 | B.1.526 | 4/19/21 | surveillance | 98.1 | 736 | hCoV-19/USA/PA-VSP2311/2021 | OK246179 |
| VSP2312 | P.1 | 4/19/21 | surveillance | 99.6 | 1414 | hCoV-19/USA/PA-VSP2312/2021 | OK246180 |
| VSP2313 | P.1 | 4/19/21 | surveillance | 99.3 | 1813 | hCoV-19/USA/PA-VSP2313/2021 | OK246181 |
| VSP2314 | P.1 | 4/19/21 | surveillance | 99.7 | 2306 | hCoV-19/USA/PA-VSP2314/2021 | OK246182 |
| VSP2315 | B.1.526 | 4/19/21 | surveillance | 99.7 | 1669 | hCoV-19/USA/PA-VSP2315/2021 | OK246183 |
| VSP2316 | B.1.526 | 4/19/21 | surveillance | 98.8 | 3222 | hCoV-19/USA/PA-VSP2316/2021 | OK246184 |
| VSP2317 | B.1.1.7 | 4/19/21 | surveillance | 99.7 | 3529 | hCoV-19/USA/PA-VSP2317/2021 | OK246185 |
| VSP2318 | B.1.1.7 | 4/19/21 | surveillance | 98.6 | 803 | hCoV-19/USA/PA-VSP2318/2021 | OK246186 |
| VSP2319 | B.1.1.7 | 4/19/21 | surveillance | 99.7 | 946 | hCoV-19/USA/PA-VSP2319/2021 | OK246187 |
| VSP2320 | P.1 | 4/19/21 | surveillance | 99.7 | 1302 | hCoV-19/USA/PA-VSP2320/2021 | OK246188 |
| VSP2322 | B.1.526 | 4/19/21 | surveillance | 99.7 | 1393 | hCoV-19/USA/PA-VSP2322/2021 | OK246189 |
| VSP2323 | B.1.1.7 | 4/19/21 | surveillance | 99.7 | 1985 | hCoV-19/USA/PA-VSP2323/2021 | OK246190 |
| VSP2324 | B.1.637 | 4/19/21 | surveillance | 98.8 | 3425 | hCoV-19/USA/PA-VSP2324/2021 | OK246191 |
| VSP2325 | P.1 | 4/19/21 | surveillance | 99.7 | 2665 | hCoV-19/USA/PA-VSP2325/2021 | OK246192 |
| VSP2326 | B.1.526 | 4/19/21 | surveillance | 99.7 | 2953 | hCoV-19/USA/PA-VSP2326/2021 | OK246193 |
| VSP2327 | B.1.526 | 4/19/21 | surveillance | 99.7 | 2106 | hCoV-19/USA/PA-VSP2327/2021 | OK246194 |
| VSP2328 | B.1.526 | 4/19/21 | surveillance | 99.7 | 2324 | hCoV-19/USA/PA-VSP2328/2021 | OK246195 |
| VSP2329 | B.1.1.7 | 4/19/21 | surveillance | 99.7 | 2698 | hCoV-19/USA/PA-VSP2329/2021 | OK246196 |
| VSP2330 | B.1.1.7 | 4/19/21 | surveillance | 99.7 | 2516 | hCoV-19/USA/PA-VSP2330/2021 | OK246197 |
| VSP2331 | B.1.1.7 | 4/19/21 | surveillance | 99.7 | 2532 | hCoV-19/USA/PA-VSP2331/2021 | OK246198 |
| VSP2332 | B.1.1.7 | 4/19/21 | surveillance | 99.7 | 1919 | hCoV-19/USA/PA-VSP2332/2021 | OK246199 |
| VSP2333 | B.1.526 | 4/19/21 | surveillance | 99.7 | 3115 | hCoV-19/USA/PA-VSP2333/2021 | OK246200 |
| VSP2334 | B.1.526 | 4/19/21 | surveillance | 98.1 | 2732 | hCoV-19/USA/PA-VSP2334/2021 | OK246201 |
| VSP2335 | B.1.1.7 | 4/19/21 | surveillance | 99.6 | 3226 | hCoV-19/USA/PA-VSP2335/2021 | OK246202 |
| VSP2336 | B.1.526 | 4/19/21 | surveillance | 99.1 | 2376 | hCoV-19/USA/PA-VSP2336/2021 | OK246203 |
| VSP2337 | B.1.526 | 4/19/21 | surveillance | 97.6 | 997 | hCoV-19/USA/PA-VSP2337/2021 | |
| VSP2338 | B.1.1.7 | 4/19/21 | surveillance | 99.7 | 2715 | hCoV-19/USA/PA-VSP2338/2021 | OK246204 |
| VSP2339 | B.1.526 | 4/19/21 | surveillance | 99.7 | 2445 | hCoV-19/USA/PA-VSP2339/2021 | OK246205 |
| VSP2340 | B.1.1.7 | 4/19/21 | surveillance | 99.7 | 3699 | hCoV-19/USA/PA-VSP2340/2021 | OK246206 |
| VSP2341 | P.1 | 4/19/21 | surveillance | 99.4 | 3108 | hCoV-19/USA/PA-VSP2341/2021 | OK246207 |
| VSP2342 | P.1 | 4/19/21 | surveillance | 99.6 | 2383 | hCoV-19/USA/PA-VSP2342/2021 | OK246208 |
| VSP2343 | B.1.1.7 | 4/19/21 | surveillance | 95.7 | 27 | hCoV-19/USA/PA-VSP2343/2021 | OK246209 |
| VSP2344 | B.1.621 | 4/19/21 | surveillance | 99.7 | 3741 | hCoV-19/USA/PA-VSP2344/2021 | OK246210 |
| VSP2345 | B.1.1.7 | 4/19/21 | surveillance | 99.7 | 2930 | hCoV-19/USA/PA-VSP2345/2021 | OK246211 |
| VSP2346 | B.1.1.7 | 4/19/21 | surveillance | 99.7 | 2596 | hCoV-19/USA/PA-VSP2346/2021 | OK246212 |

| | | | | | | | |
|---|---|---|---|---|---|---|---|
| VSP2347 | B.1.1.7 | 4/19/21 | surveillance | 99.5 | 1928 | hCoV-19/USA/PA-VSP2347/2021 | OK246213 |
| VSP2348 | B.1.1.7 | 4/19/21 | surveillance | 98.2 | 99 | hCoV-19/USA/PA-VSP2348/2021 | OK246214 |
| VSP2349 | B.1.1.7 | 4/19/21 | surveillance | 99.1 | 323 | hCoV-19/USA/PA-VSP2349/2021 | OK246215 |
| VSP2350 | B.1.1.7 | 4/19/21 | surveillance | 99.3 | 1489 | hCoV-19/USA/PA-VSP2350/2021 | OK246216 |
| VSP2351 | B.1.1.7 | 4/19/21 | surveillance | 99.1 | 865 | hCoV-19/USA/PA-VSP2351/2021 | OK246217 |
| VSP2352 | B.1.1.7 | 4/19/21 | surveillance | 99.7 | 2748 | hCoV-19/USA/PA-VSP2352/2021 | OK246218 |
| VSP2353 | B.1.1.7 | 4/19/21 | surveillance | 99.6 | 1798 | hCoV-19/USA/PA-VSP2353/2021 | OK246219 |
| VSP2354 | B.1.1.7 | 4/19/21 | surveillance | 99 | 1627 | hCoV-19/USA/NJ-VSP2354/2021 | OK246220 |
| VSP2355 | B.1.526 | 4/19/21 | surveillance | 99.4 | 1363 | hCoV-19/USA/NJ-VSP2355/2021 | OK246221 |
| VSP2356 | B.1.1.7 | 4/19/21 | surveillance | 99.7 | 2194 | hCoV-19/USA/PA-VSP2356/2021 | OK246222 |
| VSP2357 | B.1.1.7 | 4/19/21 | surveillance | 99.7 | 2000 | hCoV-19/USA/NJ-VSP2357/2021 | OK246223 |
| VSP2358 | B.1.1.7 | 4/19/21 | surveillance | 99.7 | 2431 | hCoV-19/USA/NJ-VSP2358/2021 | OK246224 |
| VSP2359 | B.1.1.7 | 4/19/21 | surveillance | 99.6 | 1683 | hCoV-19/USA/NJ-VSP2359/2021 | OK246225 |
| VSP2360 | B.1.526 | 4/19/21 | surveillance | 99.7 | 2624 | hCoV-19/USA/NJ-VSP2360/2021 | OK246226 |
| VSP2361 | B.1.1.7 | 4/19/21 | surveillance | 99.7 | 2439 | hCoV-19/USA/NJ-VSP2361/2021 | OK246227 |
| VSP2369 | B.1.1.7 | 4/23/21 | hospitalized | 98.2 | 263 | hCoV-19/USA/PA-VSP2369/2021 | OK246228 |
| VSP2403 | B.1.1.7 | 4/26/21 | accine breakthroug | 99.7 | 1696 | hCoV-19/USA/PA-VSP2403/2021 | OK246229 |
| VSP2404 | B.1.621 | 4/26/21 | accine breakthroug | 99.7 | 2210 | hCoV-19/USA/FL-VSP2404/2021 | OK246230 |
| VSP2405 | B.1.621 | 4/26/21 | accine breakthroug | 99.7 | 2397 | hCoV-19/USA/NJ-VSP2405/2021 | OK246231 |
| VSP2406 | B.1.621 | 4/26/21 | accine breakthroug | 99.7 | 2248 | hCoV-19/USA/PA-VSP2406/2021 | OK246232 |
| VSP2407 | B.1.1.7 | 4/26/21 | accine breakthroug | 99.7 | 588 | hCoV-19/USA/PA-VSP2407/2021 | OK246233 |
| VSP2408 | B.1.1.7 | 4/26/21 | accine breakthroug | 99.7 | 1904 | hCoV-19/USA/NJ-VSP2408/2021 | OK246234 |
| VSP2409 | B.1.1.7 | 4/26/21 | accine breakthroug | 99.7 | 2001 | hCoV-19/USA/PA-VSP2409/2021 | OK246235 |
| VSP2410 | B.1.1.7 | 4/26/21 | accine breakthroug | 99.7 | 2415 | hCoV-19/USA/PA-VSP2410/2021 | OK246236 |
| VSP2411 | B.1.1.7 | 4/26/21 | surveillance | 99.7 | 3400 | hCoV-19/USA/PA-VSP2411/2021 | OK246237 |
| VSP2412 | B.1.1.7 | 4/26/21 | surveillance | 99.7 | 1490 | hCoV-19/USA/PA-VSP2412/2021 | OK246238 |
| VSP2413 | B.1.1.7 | 4/26/21 | surveillance | 99.7 | 1542 | hCoV-19/USA/PA-VSP2413/2021 | OK246239 |
| VSP2414 | B.1.526 | 4/26/21 | surveillance | 99.7 | 789 | hCoV-19/USA/PA-VSP2414/2021 | OK246240 |
| VSP2415 | B.1.1.7 | 4/26/21 | surveillance | 99.7 | 3574 | hCoV-19/USA/DE-VSP2415/2021 | OK246241 |
| VSP2416 | B.1.1.7 | 4/26/21 | surveillance | 99.7 | 3806 | hCoV-19/USA/PA-VSP2416/2021 | OK246242 |
| VSP2417 | B.1.526 | 4/26/21 | surveillance | 99.7 | 3443 | hCoV-19/USA/PA-VSP2417/2021 | OK246243 |
| VSP2418 | B.1.526 | 4/26/21 | surveillance | 99.7 | 3599 | hCoV-19/USA/PA-VSP2418/2021 | OK246244 |
| VSP2419 | B.1.2 | 4/26/21 | surveillance | 99.7 | 3538 | hCoV-19/USA/PA-VSP2419/2021 | OK246245 |
| VSP2420 | B.1.1.7 | 4/26/21 | surveillance | 99 | 177 | hCoV-19/USA/PA-VSP2420/2021 | OK246246 |
| VSP2422 | B.1.526 | 4/26/21 | surveillance | 99.7 | 3353 | hCoV-19/USA/PA-VSP2422/2021 | OK246247 |
| VSP2423 | B.1.1.7 | 4/26/21 | surveillance | 99.7 | 1326 | hCoV-19/USA/PA-VSP2423/2021 | OK246248 |
| VSP2424 | B.1.1.7 | 4/26/21 | surveillance | 99.7 | 1272 | hCoV-19/USA/PA-VSP2424/2021 | OK246249 |
| VSP2425 | B.1.1.7 | 4/26/21 | surveillance | 98.8 | 120 | hCoV-19/USA/PA-VSP2425/2021 | OK246250 |
| VSP2426 | B.1.1.7 | 4/26/21 | surveillance | 99.7 | 1809 | hCoV-19/USA/PA-VSP2426/2021 | OK246251 |
| VSP2427 | B.1.1.7 | 4/26/21 | surveillance | 99.7 | 3764 | hCoV-19/USA/PA-VSP2427/2021 | OK246252 |
| VSP2428 | B.1.1.7 | 4/26/21 | surveillance | 99.7 | 3649 | hCoV-19/USA/PA-VSP2428/2021 | OK246253 |
| VSP2429 | B.1.526 | 4/26/21 | surveillance | 98.7 | 92 | hCoV-19/USA/PA-VSP2429/2021 | OK246254 |
| VSP2430 | B.1.637 | 4/26/21 | surveillance | 99.7 | 3529 | hCoV-19/USA/PA-VSP2430/2021 | OK246255 |
| VSP2431 | B.1.1.7 | 4/26/21 | surveillance | 99.7 | 3580 | hCoV-19/USA/PA-VSP2431/2021 | OK246256 |
| VSP2432 | B.1.1.7 | 4/26/21 | surveillance | 99.7 | 2189 | hCoV-19/USA/PA-VSP2432/2021 | OK246257 |
| VSP2433 | B.1.526 | 4/26/21 | surveillance | 99.7 | 419 | hCoV-19/USA/PA-VSP2433/2021 | OK246258 |
| VSP2435 | P.1 | 4/26/21 | surveillance | 99.3 | 297 | hCoV-19/USA/PA-VSP2435/2021 | OK246259 |

| | | | | | | | |
|---|---|---|---|---|---|---|---|
| VSP2436 | B.1.526 | 4/26/21 | surveillance | 98.2 | 128 | hCoV-19/USA/PA-VSP2436/2021 | OK246260 |
| VSP2437 | B.1.1.7 | 4/26/21 | surveillance | 99.1 | 575 | hCoV-19/USA/PA-VSP2437/2021 | OK246261 |
| VSP2438 | B.1.1.7 | 4/26/21 | surveillance | 99.7 | 2881 | hCoV-19/USA/PA-VSP2438/2021 | OK246262 |
| VSP2439 | B.1.1.7 | 4/26/21 | surveillance | 99.7 | 1968 | hCoV-19/USA/NJ-VSP2439/2021 | OK246263 |
| VSP2440 | B.1.1.7 | 4/26/21 | surveillance | 99.7 | 2246 | hCoV-19/USA/PA-VSP2440/2021 | OK246264 |
| VSP2441 | B.1.1.7 | 4/26/21 | surveillance | 99.7 | 3847 | hCoV-19/USA/PA-VSP2441/2021 | OK246265 |
| VSP2442 | B.1.1.7 | 4/26/21 | surveillance | 99 | 119 | hCoV-19/USA/PA-VSP2442/2021 | OK246266 |
| VSP2443 | B.1.637 | 4/26/21 | surveillance | 99.7 | 4129 | hCoV-19/USA/PA-VSP2443/2021 | OK246267 |
| VSP2444 | B.1.621 | 4/26/21 | surveillance | 99.7 | 1440 | hCoV-19/USA/PA-VSP2444/2021 | OK246268 |
| VSP2445 | B.1.1.7 | 4/26/21 | surveillance | 99.7 | 1049 | hCoV-19/USA/PA-VSP2445/2021 | OK246269 |
| VSP2446 | B.1.526 | 4/26/21 | surveillance | 99.5 | 3214 | hCoV-19/USA/PA-VSP2446/2021 | OK246270 |
| VSP2447 | B.1.1.7 | 4/26/21 | surveillance | 99.7 | 2534 | hCoV-19/USA/PA-VSP2447/2021 | OK246271 |
| VSP2448 | B.1.1.7 | 4/26/21 | surveillance | 97.6 | 46 | hCoV-19/USA/PA-VSP2448/2021 | OK246272 |
| VSP2449 | B.1.1.7 | 4/26/21 | surveillance | 99.7 | 3411 | hCoV-19/USA/PA-VSP2449/2021 | OK246273 |
| VSP2450 | B.1.1.7 | 4/26/21 | surveillance | 99.7 | 949 | hCoV-19/USA/PA-VSP2450/2021 | OK246274 |
| VSP2451 | B.1.1.7 | 4/26/21 | surveillance | 99.7 | 3779 | hCoV-19/USA/PA-VSP2451/2021 | OK246275 |
| VSP2452 | B.1.1.7 | 4/26/21 | surveillance | 99.7 | 3680 | hCoV-19/USA/PA-VSP2452/2021 | OK246276 |
| VSP2453 | B.1.526 | 4/26/21 | surveillance | 99.7 | 3482 | hCoV-19/USA/PA-VSP2453/2021 | OK246277 |
| VSP2454 | B.1.526 | 4/26/21 | surveillance | 99.7 | 3751 | hCoV-19/USA/PA-VSP2454/2021 | OK246278 |
| VSP2455 | B.1.1.7 | 4/26/21 | surveillance | 99.7 | 3552 | hCoV-19/USA/PA-VSP2455/2021 | OK246279 |
| VSP2456 | B.1.1.7 | 4/26/21 | surveillance | 99.7 | 3178 | hCoV-19/USA/PA-VSP2456/2021 | OK246280 |
| VSP2457 | B.1.1.7 | 4/26/21 | surveillance | 96.5 | 320 | hCoV-19/USA/PA-VSP2457/2021 | |
| VSP2458 | B.1.1.7 | 4/26/21 | surveillance | 99.7 | 3606 | hCoV-19/USA/PA-VSP2458/2021 | OK246281 |
| VSP2459 | B.1.2 | 4/26/21 | surveillance | 96.4 | 30 | hCoV-19/USA/PA-VSP2459/2021 | OK246282 |
| VSP2461 | B.1.1.7 | 4/26/21 | surveillance | 99.7 | 3309 | hCoV-19/USA/PA-VSP2461/2021 | OK246283 |
| VSP2462 | B.1.1.7 | 4/26/21 | surveillance | 99.7 | 3550 | hCoV-19/USA/PA-VSP2462/2021 | OK246284 |
| VSP2463 | B.1.1.7 | 4/26/21 | surveillance | 99.7 | 1573 | hCoV-19/USA/PA-VSP2463/2021 | OK246285 |
| VSP2464 | B.1.1.7 | 4/26/21 | surveillance | 99.7 | 4116 | hCoV-19/USA/PA-VSP2464/2021 | OK246286 |
| VSP2465 | B.1.637 | 4/26/21 | surveillance | 98.8 | 3783 | hCoV-19/USA/PA-VSP2465/2021 | OK246287 |
| VSP2466 | B.1.1.7 | 4/26/21 | surveillance | 99.5 | 1369 | hCoV-19/USA/PA-VSP2466/2021 | OK246288 |
| VSP2467 | B.1.575 | 4/26/21 | surveillance | 99.1 | 683 | hCoV-19/USA/GA-VSP2467/2021 | OK246289 |
| VSP2468 | B.1.1.7 | 4/26/21 | surveillance | 99.7 | 3145 | hCoV-19/USA/PA-VSP2468/2021 | OK246290 |
| VSP2470 | P.1 | 4/26/21 | surveillance | 99.7 | 3532 | hCoV-19/USA/PA-VSP2470/2021 | OK246291 |
| VSP2471 | P.1 | 4/26/21 | surveillance | 99.7 | 4224 | hCoV-19/USA/PA-VSP2471/2021 | OK246292 |
| VSP2472 | B.1.526 | 4/26/21 | surveillance | 99.7 | 4048 | hCoV-19/USA/PA-VSP2472/2021 | OK246293 |
| VSP2474 | B.1.526 | 4/26/21 | surveillance | 99.7 | 3448 | hCoV-19/USA/PA-VSP2474/2021 | OK246294 |
| VSP2475 | B.1.1.7 | 4/26/21 | surveillance | 99.7 | 3724 | hCoV-19/USA/PA-VSP2475/2021 | OK246295 |
| VSP2476 | P.1 | 4/26/21 | surveillance | 99.2 | 495 | hCoV-19/USA/PA-VSP2476/2021 | OK246296 |
| VSP2477 | B.1.1.7 | 4/26/21 | surveillance | 99.7 | 1764 | hCoV-19/USA/PA-VSP2477/2021 | OK246297 |
| VSP2478 | B.1.1.7 | 4/26/21 | surveillance | 99.7 | 1455 | hCoV-19/USA/PA-VSP2478/2021 | OK246298 |
| VSP2479 | B.1.526 | 4/26/21 | surveillance | 99.1 | 1975 | hCoV-19/USA/PA-VSP2479/2021 | OK246299 |
| VSP2480 | B.1.1.7 | 4/26/21 | surveillance | 99.7 | 3520 | hCoV-19/USA/PA-VSP2480/2021 | OK246300 |
| VSP2481 | B.1.526 | 4/26/21 | surveillance | 99.7 | 2626 | hCoV-19/USA/PA-VSP2481/2021 | OK246301 |
| VSP2482 | B.1.1.7 | 4/26/21 | surveillance | 99.7 | 2857 | hCoV-19/USA/PA-VSP2482/2021 | OK246302 |
| VSP2483 | B.1.1.7 | 4/26/21 | surveillance | 99.7 | 2969 | hCoV-19/USA/PA-VSP2483/2021 | OK246303 |
| VSP2484 | B.1.526 | 4/26/21 | surveillance | 99.7 | 3105 | hCoV-19/USA/PA-VSP2484/2021 | OK246304 |
| VSP2486 | B.1.2 | 4/26/21 | surveillance | 99.7 | 3356 | hCoV-19/USA/PA-VSP2486/2021 | OK246305 |

| | | | | | | | |
|---|---|---|---|---|---|---|---|
| VSP2487 | P.1 | 4/26/21 | surveillance | 99.7 | 1785 | hCoV-19/USA/PA-VSP2487/2021 | OK246306 |
| VSP2488 | B.1.1.7 | 4/26/21 | surveillance | 99.7 | 3378 | hCoV-19/USA/PA-VSP2488/2021 | OK246307 |
| VSP2489 | B.1.526 | 4/26/21 | surveillance | 99.1 | 3522 | hCoV-19/USA/PA-VSP2489/2021 | OK246308 |
| VSP2490 | P.1 | 4/26/21 | surveillance | 99.7 | 3496 | hCoV-19/USA/PA-VSP2490/2021 | OK246309 |
| VSP2491 | B.1.1.7 | 4/26/21 | surveillance | 99.7 | 3513 | hCoV-19/USA/PA-VSP2491/2021 | OK246310 |
| VSP2492 | B.1.1.7 | 4/26/21 | surveillance | 99.7 | 3229 | hCoV-19/USA/AL-VSP2492/2021 | OK246311 |
| VSP2493 | B.1.1.7 | 4/26/21 | surveillance | 99.7 | 957 | hCoV-19/USA/PA-VSP2493/2021 | OK246312 |
| VSP2494 | B.1.1.7 | 4/26/21 | surveillance | 99.7 | 4079 | hCoV-19/USA/PA-VSP2494/2021 | OK246313 |
| VSP2495 | B.1.1.7 | 4/26/21 | surveillance | 99.7 | 3732 | hCoV-19/USA/PA-VSP2495/2021 | OK246314 |
| VSP2496 | B.1.526 | 4/26/21 | surveillance | 99.7 | 2861 | hCoV-19/USA/PA-VSP2496/2021 | OK246315 |
| VSP2497 | P.1 | 4/26/21 | surveillance | 99.7 | 1158 | hCoV-19/USA/PA-VSP2497/2021 | OK246316 |
| VSP2498 | B.1.1.7 | 4/26/21 | surveillance | 99.7 | 3492 | hCoV-19/USA/PA-VSP2498/2021 | OK246317 |
| VSP2499 | B.1.526 | 4/26/21 | surveillance | 98.8 | 53 | hCoV-19/USA/PA-VSP2499/2021 | OK246318 |
| VSP2500 | B.1.1.7 | 4/26/21 | surveillance | 99.7 | 2166 | hCoV-19/USA/PA-VSP2500/2021 | OK246319 |
| VSP2501 | B.1.1.7 | 4/26/21 | surveillance | 99 | 81 | hCoV-19/USA/PA-VSP2501/2021 | OK246320 |
| VSP2502 | B.1.526 | 4/26/21 | surveillance | 99.2 | 2155 | hCoV-19/USA/PA-VSP2502/2021 | OK246321 |
| VSP2503 | B.1.1.7 | 4/26/21 | surveillance | 99.6 | 1011 | hCoV-19/USA/PA-VSP2503/2021 | OK246322 |
| VSP2504 | B.1.575 | 4/26/21 | surveillance | 99.7 | 1428 | hCoV-19/USA/PA-VSP2504/2021 | |
| VSP2505 | B.1.617.2 | 4/26/21 | surveillance | 98.9 | 1271 | hCoV-19/USA/PA-VSP2505/2021 | OK246323 |
| VSP2506 | B.1.1.7 | 4/26/21 | surveillance | 99.6 | 1033 | hCoV-19/USA/PA-VSP2506/2021 | OK246324 |
| VSP2507 | P.1 | 4/26/21 | surveillance | 99.6 | 938 | hCoV-19/USA/PA-VSP2507/2021 | OK246325 |
| VSP2508 | B.1.1.7 | 4/26/21 | surveillance | 99.5 | 665 | hCoV-19/USA/PA-VSP2508/2021 | OK246326 |
| VSP2509 | B.1.526 | 4/26/21 | surveillance | 99.7 | 866 | hCoV-19/USA/PA-VSP2509/2021 | OK246327 |
| VSP2510 | R.1 | 4/26/21 | surveillance | 99.1 | 551 | hCoV-19/USA/PA-VSP2510/2021 | OK246328 |
| VSP2511 | B.1.526 | 4/26/21 | surveillance | 99.6 | 958 | hCoV-19/USA/PA-VSP2511/2021 | OK246329 |
| VSP2512 | B.1.1.7 | 4/26/21 | surveillance | 99.3 | 650 | hCoV-19/USA/PA-VSP2512/2021 | OK246330 |
| VSP2513 | B.1.526 | 4/26/21 | surveillance | 99.5 | 485 | hCoV-19/USA/PA-VSP2513/2021 | OK246331 |
| VSP2514 | B.1.1.7 | 4/26/21 | surveillance | 98.9 | 457 | hCoV-19/USA/PA-VSP2514/2021 | OK246332 |
| VSP2515 | B.1.1.7 | 4/26/21 | surveillance | 98.5 | 132 | hCoV-19/USA/PA-VSP2515/2021 | OK246333 |
| VSP2516 | B.1.526 | 4/26/21 | surveillance | 99.7 | 5937 | hCoV-19/USA/PA-VSP2516/2021 | OK246334 |
| VSP2517 | B.1.526 | 4/26/21 | surveillance | 99.7 | 3097 | hCoV-19/USA/PA-VSP2517/2021 | OK246335 |
| VSP2518 | B.1.526 | 4/26/21 | surveillance | 98.2 | 82 | hCoV-19/USA/PA-VSP2518/2021 | OK246336 |
| VSP2519 | B.1.1.7 | 4/26/21 | surveillance | 99.6 | 1049 | hCoV-19/USA/PA-VSP2519/2021 | OK246337 |
| VSP2520 | R.1 | 4/26/21 | surveillance | 99.7 | 1501 | hCoV-19/USA/PA-VSP2520/2021 | OK246338 |
| VSP2521 | B.1.1.7 | 4/26/21 | surveillance | 99.6 | 756 | hCoV-19/USA/LA-VSP2521/2021 | OK246339 |
| VSP2522 | B.1.1.7 | 4/26/21 | surveillance | 99.5 | 486 | hCoV-19/USA/PA-VSP2522/2021 | OK246340 |
| VSP2523 | B.1.637 | 4/26/21 | surveillance | 99.4 | 216 | hCoV-19/USA/PA-VSP2523/2021 | OK246341 |
| VSP2524 | B.1.1.7 | 4/26/21 | surveillance | 99.6 | 634 | hCoV-19/USA/NJ-VSP2524/2021 | OK246342 |
| VSP2525 | B.1.1.7 | 4/26/21 | surveillance | 99.6 | 1375 | hCoV-19/USA/PA-VSP2525/2021 | OK246343 |
| VSP2526 | B.1.1.7 | 4/26/21 | surveillance | 99.7 | 2001 | hCoV-19/USA/PA-VSP2526/2021 | OK246344 |
| VSP2527 | B.1.525 | 4/26/21 | surveillance | 99.3 | 485 | hCoV-19/USA/PA-VSP2527/2021 | |
| VSP2528 | B.1.1.7 | 4/26/21 | surveillance | 99.6 | 981 | hCoV-19/USA/PA-VSP2528/2021 | OK246345 |
| VSP2529 | B.1.1.7 | 4/26/21 | surveillance | 99.7 | 1401 | hCoV-19/USA/PA-VSP2529/2021 | OK246346 |
| VSP2530 | B.1.1.7 | 4/26/21 | surveillance | 99 | 95 | hCoV-19/USA/PA-VSP2530/2021 | OK246347 |
| VSP2534 | B.1.1.7 | 4/26/21 | surveillance | 99.3 | 169 | hCoV-19/USA/PA-VSP2534/2021 | OK246348 |
| VSP2535 | B.1.1.7 | 4/26/21 | surveillance | 99.6 | 564 | hCoV-19/USA/PA-VSP2535/2021 | OK246349 |
| VSP2536 | B.1.1.7 | 4/26/21 | surveillance | 99.7 | 2041 | hCoV-19/USA/PA-VSP2536/2021 | OK246350 |

| | | | | | | | |
|---|---|---|---|---|---|---|---|
| VSP2537 | B.1.1.7 | 4/26/21 | surveillance | 99.7 | 1315 | hCoV-19/USA/PA-VSP2537/2021 | OK246351 |
| VSP2538 | B.1.526 | 4/26/21 | surveillance | 99.3 | 415 | hCoV-19/USA/PA-VSP2538/2021 | OK246352 |
| VSP2539 | B.1.621 | 4/26/21 | surveillance | 99.7 | 904 | hCoV-19/USA/PA-VSP2539/2021 | OK246353 |
| VSP2540 | B.1.621 | 4/26/21 | surveillance | 99.7 | 1135 | hCoV-19/USA/PA-VSP2540/2021 | OK246354 |
| VSP2541 | B.1.1.7 | 4/26/21 | surveillance | 99.7 | 1272 | hCoV-19/USA/PA-VSP2541/2021 | OK246355 |
| VSP2542 | B.1.1.7 | 4/26/21 | surveillance | 99.6 | 682 | hCoV-19/USA/PA-VSP2542/2021 | OK246356 |
| VSP2544 | B.1.526 | 4/26/21 | surveillance | 99.6 | 3096 | hCoV-19/USA/NJ-VSP2544/2021 | OK246357 |
| VSP2545 | B.1.1.7 | 4/26/21 | surveillance | 99.3 | 605 | hCoV-19/USA/NJ-VSP2545/2021 | OK246358 |
| VSP2546 | B.1.637 | 4/26/21 | surveillance | 99.7 | 2327 | hCoV-19/USA/NJ-VSP2546/2021 | OK246359 |
| VSP2547 | B.1.1.7 | 4/26/21 | surveillance | 99.7 | 2779 | hCoV-19/USA/NJ-VSP2547/2021 | OK246360 |
| VSP2550 | B.1.1.519 | 4/26/21 | surveillance | 99.7 | 2458 | hCoV-19/USA/NJ-VSP2550/2021 | OK246361 |
| VSP2551 | C.37 | 4/26/21 | surveillance | 99.7 | 2114 | hCoV-19/USA/NJ-VSP2551/2021 | OK246362 |
| VSP2552 | B.1.1.7 | 4/26/21 | surveillance | 99.7 | 3765 | hCoV-19/USA/NJ-VSP2552/2021 | OK246363 |
| VSP2553 | B.1.1.7 | 4/26/21 | surveillance | 99.7 | 3061 | hCoV-19/USA/PA-VSP2553/2021 | OK246364 |
| VSP2554 | B.1.526 | 4/26/21 | surveillance | 99.7 | 2678 | hCoV-19/USA/PA-VSP2554/2021 | OK246365 |
| VSP2555 | B.1.1.7 | 4/26/21 | surveillance | 99.7 | 3003 | hCoV-19/USA/PA-VSP2555/2021 | OK246366 |
| VSP2556 | B.1.526 | 4/26/21 | surveillance | 99.6 | 3453 | hCoV-19/USA/PA-VSP2556/2021 | OK246367 |
| VSP2557 | B.1.1.7 | 4/26/21 | surveillance | 99.7 | 2258 | hCoV-19/USA/PA-VSP2557/2021 | OK246368 |
| VSP2558 | B.1.1.7 | 4/26/21 | surveillance | 99.7 | 1494 | hCoV-19/USA/PA-VSP2558/2021 | OK246369 |
| VSP2559 | B.1.1.7 | 4/26/21 | surveillance | 99.7 | 2633 | hCoV-19/USA/PA-VSP2559/2021 | OK246370 |
| VSP2560 | B.1.1.7 | 4/26/21 | surveillance | 99 | 679 | hCoV-19/USA/PA-VSP2560/2021 | OK246371 |
| VSP2561 | B.1.621 | 4/26/21 | surveillance | 99.7 | 3259 | hCoV-19/USA/PA-VSP2561/2021 | OK246372 |
| VSP2562 | B.1.1.7 | 4/26/21 | surveillance | 99.7 | 3268 | hCoV-19/USA/PA-VSP2562/2021 | OK246373 |
| VSP2563 | P.1 | 4/26/21 | surveillance | 99.7 | 2105 | hCoV-19/USA/PA-VSP2563/2021 | OK246374 |
| VSP2564 | B.1.526 | 4/26/21 | surveillance | 99.7 | 2761 | hCoV-19/USA/NJ-VSP2564/2021 | OK246375 |
| VSP2565 | B.1.621 | 4/26/21 | surveillance | 99.7 | 5354 | hCoV-19/USA/PA-VSP2565/2021 | OK246376 |
| VSP2567 | B.1.621 | 4/26/21 | surveillance | 99.7 | 1441 | hCoV-19/USA/VA-VSP2567/2021 | OK246377 |
| VSP2568 | Q.4 | 4/26/21 | surveillance | 99.7 | 1228 | hCoV-19/USA/NJ-VSP2568/2021 | OK246378 |
| VSP2570 | B.1.1.7 | 5/4/21 | accine breakthroug | 99.4 | 393 | hCoV-19/USA/NJ-VSP2570/2021 | OK246379 |
| VSP2571 | B.1.637 | 5/4/21 | surveillance | 99 | 1230 | hCoV-19/USA/PA-VSP2571/2021 | OK246380 |
| VSP2572 | B.1.1.7 | 5/4/21 | surveillance | 99.7 | 2182 | hCoV-19/USA/PA-VSP2572/2021 | OK246381 |
| VSP2573 | B.1.526 | 5/4/21 | surveillance | 99.7 | 1621 | hCoV-19/USA/PA-VSP2573/2021 | OK246382 |
| VSP2574 | B.1.526 | 5/4/21 | surveillance | 99.7 | 1867 | hCoV-19/USA/PA-VSP2574/2021 | OK246383 |
| VSP2575 | B.1.1.7 | 5/4/21 | surveillance | 99.7 | 772 | hCoV-19/USA/PA-VSP2575/2021 | OK246384 |
| VSP2576 | B.1.621 | 5/4/21 | surveillance | 99 | 153 | hCoV-19/USA/PA-VSP2576/2021 | OK246385 |
| VSP2577 | B.1.1.7 | 5/4/21 | surveillance | 99.7 | 1747 | hCoV-19/USA/PA-VSP2577/2021 | OK246386 |
| VSP2578 | P.1.10 | 5/4/21 | surveillance | 99.7 | 1615 | hCoV-19/USA/PA-VSP2578/2021 | OK246387 |
| VSP2579 | B.1.1.7 | 5/4/21 | surveillance | 99.7 | 1091 | hCoV-19/USA/PA-VSP2579/2021 | OK246388 |
| VSP2580 | B.1.526 | 5/4/21 | surveillance | 99.7 | 1507 | hCoV-19/USA/PA-VSP2580/2021 | OK246389 |
| VSP2581 | B.1.1.7 | 5/4/21 | surveillance | 99.7 | 1819 | hCoV-19/USA/PA-VSP2581/2021 | OK246390 |
| VSP2582 | B.1.1.7 | 5/4/21 | surveillance | 99.7 | 1465 | hCoV-19/USA/PA-VSP2582/2021 | OK246391 |
| VSP2583 | B.1.526 | 5/4/21 | surveillance | 99.6 | 525 | hCoV-19/USA/PA-VSP2583/2021 | OK246392 |
| VSP2584 | B.1.621 | 5/4/21 | surveillance | 99.7 | 1699 | hCoV-19/USA/PA-VSP2584/2021 | OK246393 |
| VSP2585 | B.1.1.7 | 5/4/21 | surveillance | 99.7 | 1540 | hCoV-19/USA/PA-VSP2585/2021 | OK246394 |
| VSP2586 | B.1.1.7 | 5/4/21 | surveillance | 99.7 | 1526 | hCoV-19/USA/PA-VSP2586/2021 | OK246395 |
| VSP2587 | B.1.1.7 | 5/4/21 | surveillance | 99.7 | 1646 | hCoV-19/USA/PA-VSP2587/2021 | OK246396 |
| VSP2588 | B.1.1.7 | 5/4/21 | surveillance | 99.7 | 3546 | hCoV-19/USA/PA-VSP2588/2021 | OK246397 |

| | | | | | | | |
|---|---|---|---|---|---|---|---|
| VSP2589 | B.1.1.7 | 5/4/21 | surveillance | 99.7 | 3589 | hCoV-19/USA/PA-VSP2589/2021 | OK246398 |
| VSP2590 | B.1.1.7 | 5/4/21 | surveillance | 99.7 | 2078 | hCoV-19/USA/PA-VSP2590/2021 | OK246399 |
| VSP2591 | B.1.1.7 | 5/4/21 | surveillance | 99.7 | 3258 | hCoV-19/USA/NJ-VSP2591/2021 | OK246400 |
| VSP2592 | B.1.526 | 5/4/21 | surveillance | 99.7 | 2193 | hCoV-19/USA/PA-VSP2592/2021 | OK246401 |
| VSP2593 | B.1.1.7 | 5/4/21 | surveillance | 99.7 | 3414 | hCoV-19/USA/PA-VSP2593/2021 | OK246402 |
| VSP2594 | B.1.637 | 5/4/21 | surveillance | 99.7 | 3435 | hCoV-19/USA/PA-VSP2594/2021 | OK246403 |
| VSP2595 | B.1.1.7 | 5/4/21 | surveillance | 99.7 | 4077 | hCoV-19/USA/PA-VSP2595/2021 | OK246404 |
| VSP2596 | B.1.1.7 | 5/4/21 | surveillance | 99.7 | 3116 | hCoV-19/USA/PA-VSP2596/2021 | OK246405 |
| VSP2597 | B.1.1.7 | 5/4/21 | surveillance | 99.7 | 3651 | hCoV-19/USA/PA-VSP2597/2021 | OK246406 |
| VSP2599 | B.1.1.7 | 5/4/21 | surveillance | 99.7 | 2337 | hCoV-19/USA/PA-VSP2599/2021 | OK246407 |
| VSP2600 | B.1.1.7 | 5/4/21 | surveillance | 99.7 | 4114 | hCoV-19/USA/PA-VSP2600/2021 | OK246408 |
| VSP2601 | B.1.1.7 | 5/4/21 | surveillance | 99.7 | 3460 | hCoV-19/USA/PA-VSP2601/2021 | OK246409 |
| VSP2602 | B.1.637 | 5/4/21 | surveillance | 99.7 | 3454 | hCoV-19/USA/PA-VSP2602/2021 | OK246410 |
| VSP2603 | R.1 | 5/4/21 | surveillance | 99.7 | 2619 | hCoV-19/USA/PA-VSP2603/2021 | OK246411 |
| VSP2604 | B.1.1.7 | 5/4/21 | surveillance | 99.7 | 2130 | hCoV-19/USA/PA-VSP2604/2021 | OK246412 |
| VSP2605 | B.1.621 | 5/4/21 | surveillance | 99.7 | 2801 | hCoV-19/USA/PA-VSP2605/2021 | OK246413 |
| VSP2606 | B.1.1.7 | 5/4/21 | surveillance | 99.7 | 3680 | hCoV-19/USA/PA-VSP2606/2021 | OK246414 |
| VSP2607 | B.1.1.7 | 5/4/21 | surveillance | 99.7 | 1655 | hCoV-19/USA/PA-VSP2607/2021 | OK246415 |
| VSP2608 | B.1.621 | 5/4/21 | surveillance | 99.7 | 2702 | hCoV-19/USA/PA-VSP2608/2021 | OK246416 |
| VSP2609 | B.1.1.519 | 5/4/21 | surveillance | 99.7 | 2935 | hCoV-19/USA/PA-VSP2609/2021 | OK246417 |
| VSP2610 | B.1.526 | 5/4/21 | surveillance | 99.7 | 2924 | hCoV-19/USA/PA-VSP2610/2021 | OK246418 |
| VSP2611 | B.1.1.7 | 5/4/21 | surveillance | 99.7 | 3342 | hCoV-19/USA/PA-VSP2611/2021 | OK246419 |
| VSP2612 | P.1 | 5/4/21 | surveillance | 99.7 | 4062 | hCoV-19/USA/PA-VSP2612/2021 | OK246420 |
| VSP2613 | B.1.1.7 | 5/4/21 | surveillance | 98.6 | 363 | hCoV-19/USA/PA-VSP2613/2021 | OK246421 |
| VSP2614 | B.1.526 | 5/4/21 | surveillance | 99.7 | 3072 | hCoV-19/USA/PA-VSP2614/2021 | OK246422 |
| VSP2615 | B.1.1.7 | 5/4/21 | surveillance | 99.7 | 3178 | hCoV-19/USA/PA-VSP2615/2021 | OK246423 |
| VSP2616 | B.1.1.7 | 5/4/21 | surveillance | 99.7 | 3592 | hCoV-19/USA/PA-VSP2616/2021 | OK246424 |
| VSP2617 | B.1.1.7 | 5/4/21 | surveillance | 99.7 | 3172 | hCoV-19/USA/PA-VSP2617/2021 | OK246425 |
| VSP2618 | B.1.1.7 | 5/4/21 | surveillance | 99.7 | 4175 | hCoV-19/USA/PA-VSP2618/2021 | OK246426 |
| VSP2619 | B.1.526 | 5/4/21 | surveillance | 99.7 | 4226 | hCoV-19/USA/PA-VSP2619/2021 | OK246427 |
| VSP2620 | B.1.1.7 | 5/4/21 | surveillance | 99.7 | 3207 | hCoV-19/USA/PA-VSP2620/2021 | OK246428 |
| VSP2621 | B.1.526 | 5/4/21 | surveillance | 99.7 | 3973 | hCoV-19/USA/PA-VSP2621/2021 | OK246429 |
| VSP2622 | B.1.1.7 | 5/4/21 | surveillance | 99.7 | 1260 | hCoV-19/USA/PA-VSP2622/2021 | OK246430 |
| VSP2623 | B.1.526 | 5/4/21 | surveillance | 99.7 | 3865 | hCoV-19/USA/PA-VSP2623/2021 | OK246431 |
| VSP2624 | B.1.1.7 | 5/4/21 | surveillance | 99.7 | 3724 | hCoV-19/USA/PA-VSP2624/2021 | OK246432 |
| VSP2625 | B.1.526 | 5/4/21 | surveillance | 99.7 | 4135 | hCoV-19/USA/PA-VSP2625/2021 | OK246433 |
| VSP2626 | B.1.1.7 | 5/4/21 | surveillance | 99.7 | 3956 | hCoV-19/USA/PA-VSP2626/2021 | OK246434 |
| VSP2627 | B.1.1.7 | 5/4/21 | surveillance | 99.7 | 3510 | hCoV-19/USA/PA-VSP2627/2021 | OK246435 |
| VSP2628 | B.1.1.7 | 5/4/21 | surveillance | 99.7 | 3631 | hCoV-19/USA/PA-VSP2628/2021 | OK246436 |
| VSP2629 | B.1.1.7 | 5/4/21 | surveillance | 99.7 | 4355 | hCoV-19/USA/PA-VSP2629/2021 | OK246437 |
| VSP2630 | B.1.1.7 | 5/4/21 | surveillance | 97.6 | 113 | hCoV-19/USA/PA-VSP2630/2021 | OK246438 |
| VSP2631 | P.1.10 | 5/4/21 | surveillance | 98.9 | 611 | hCoV-19/USA/DE-VSP2631/2021 | OK246439 |
| VSP2632 | B.1.1.7 | 5/4/21 | surveillance | 99.7 | 2995 | hCoV-19/USA/PA-VSP2632/2021 | OK246440 |
| VSP2634 | B.1.526 | 5/4/21 | surveillance | 99.7 | 2297 | hCoV-19/USA/VSP2634/2021 | MZ512878 |
| VSP2635 | B.1.526 | 5/4/21 | surveillance | 99.7 | 2008 | hCoV-19/USA/VSP2635/2021 | MZ512879 |
| VSP2636 | B.1.1.7 | 5/4/21 | surveillance | 99.7 | 2774 | hCoV-19/USA/VSP2636/2021 | MZ512880 |
| VSP2638 | B.1.1.7 | 5/4/21 | surveillance | 99.4 | 213 | hCoV-19/USA/VSP2638/2021 | MZ512881 |

| | | | | | | | |
|---|---|---|---|---|---|---|---|
| VSP2639 | P.1 | 5/4/21 | surveillance | 99.6 | 119 | hCoV-19/USA/VSP2639/2021 | MZ512882 |
| VSP2640 | B.1.1.7 | 5/4/21 | surveillance | 97.7 | 100 | hCoV-19/USA/VSP2640/2021 | MZ512883 |
| VSP2641 | B.1.1.7 | 5/4/21 | surveillance | 95.6 | 31 | hCoV-19/USA/VSP2641/2021 | MZ512884 |
| VSP2642 | B.1.637 | 5/4/21 | surveillance | 96.2 | 54 | hCoV-19/USA/VSP2642/2021 | MZ512885 |
| VSP2643 | B.1 | 5/4/21 | surveillance | 96.5 | 41 | hCoV-19/USA/VSP2643/2021 | MZ512886 |
| VSP2646 | B.1.351 | 5/4/21 | surveillance | 98.9 | 215 | hCoV-19/USA/VSP2646/2021 | MZ512887 |
| VSP2648 | B.1.1.7 | 5/4/21 | surveillance | 99.6 | 224 | hCoV-19/USA/VSP2648/2021 | MZ512888 |
| VSP2649 | P.1 | 5/4/21 | surveillance | 96.7 | 58 | hCoV-19/USA/VSP2649/2021 | MZ512889 |
| VSP2652 | B.1.526 | 5/4/21 | surveillance | 96.2 | 58 | hCoV-19/USA/VSP2652/2021 | MZ512890 |
| VSP2653 | B.1.526 | 5/4/21 | surveillance | 96.8 | 61 | hCoV-19/USA/VSP2653/2021 | MZ512891 |
| VSP2655 | B.1.526 | 5/4/21 | surveillance | 96 | 111 | hCoV-19/USA/VSP2655/2021 | MZ512892 |
| VSP2656 | B.1.526 | 5/4/21 | surveillance | 99.3 | 232 | hCoV-19/USA/VSP2656/2021 | MZ512893 |
| VSP2657 | B.1.1.7 | 5/4/21 | surveillance | 99 | 59 | hCoV-19/USA/VSP2657/2021 | MZ512894 |
| VSP2658 | B.1.1.7 | 5/4/21 | surveillance | 98 | 97 | hCoV-19/USA/VSP2658/2021 | MZ512895 |
| VSP2659 | B.1.1.7 | 5/4/21 | surveillance | 98.3 | 141 | hCoV-19/USA/VSP2659/2021 | MZ512896 |
| VSP2661 | B.1.1.7 | 5/4/21 | surveillance | 99.3 | 149 | hCoV-19/USA/VSP2661/2021 | MZ512897 |
| VSP2662 | B.1.1.519 | 5/4/21 | surveillance | 98.2 | 60 | hCoV-19/USA/VSP2662/2021 | MZ512898 |
| VSP2663 | B.1.1.7 | 5/4/21 | surveillance | 99.6 | 251 | hCoV-19/USA/VSP2663/2021 | MZ512899 |
| VSP2664 | B.1.526 | 5/4/21 | surveillance | 99.5 | 187 | hCoV-19/USA/VSP2664/2021 | MZ512900 |
| VSP2665 | B.1.1.7 | 5/4/21 | surveillance | 99 | 166 | hCoV-19/USA/VSP2665/2021 | MZ512901 |
| VSP2666 | B.1.1.7 | 5/4/21 | surveillance | 99 | 65 | hCoV-19/USA/VSP2666/2021 | MZ512902 |
| VSP2667 | B.1.1.7 | 5/4/21 | surveillance | 98.2 | 40 | hCoV-19/USA/VSP2667/2021 | MZ512903 |
| VSP2668 | B.1.1.7 | 5/4/21 | surveillance | 99.5 | 239 | hCoV-19/USA/VSP2668/2021 | MZ512904 |
| VSP2669 | B.1.1.7 | 5/4/21 | surveillance | 99.6 | 158 | hCoV-19/USA/VSP2669/2021 | MZ512905 |
| VSP2670 | B.1.637 | 5/4/21 | surveillance | 99.6 | 246 | hCoV-19/USA/VSP2670/2021 | MZ512906 |
| VSP2671 | B.1.1.7 | 5/4/21 | surveillance | 99.1 | 169 | hCoV-19/USA/VSP2671/2021 | MZ512907 |
| VSP2672 | B.1.1.7 | 5/4/21 | surveillance | 99.1 | 107 | hCoV-19/USA/VSP2672/2021 | MZ512908 |
| VSP2673 | B.1.1.7 | 5/4/21 | surveillance | 99.4 | 153 | hCoV-19/USA/VSP2673/2021 | MZ512909 |
| VSP2674 | B.1.1.7 | 5/4/21 | surveillance | 98.6 | 95 | hCoV-19/USA/VSP2674/2021 | MZ512910 |
| VSP2675 | B.1.1.7 | 5/4/21 | surveillance | 99.6 | 401 | hCoV-19/USA/VSP2675/2021 | MZ512911 |
| VSP2676 | B.1.1.7 | 5/4/21 | surveillance | 96.9 | 40 | hCoV-19/USA/VSP2676/2021 | MZ512912 |
| VSP2679 | B.1.1.7 | 5/4/21 | surveillance | 99.3 | 288 | hCoV-19/USA/VSP2679/2021 | MZ512913 |
| VSP2680 | B.1.1.7 | 5/4/21 | surveillance | 97.9 | 78 | hCoV-19/USA/VSP2680/2021 | MZ512914 |
| VSP2681 | B.1.1.7 | 5/4/21 | surveillance | 99.4 | 193 | hCoV-19/USA/VSP2681/2021 | MZ512915 |
| VSP2682 | B.1.621 | 5/4/21 | surveillance | 99.7 | 192 | hCoV-19/USA/VSP2682/2021 | MZ512916 |
| VSP2683 | B.1.1.7 | 5/4/21 | surveillance | 98.2 | 57 | hCoV-19/USA/VSP2683/2021 | MZ512917 |
| VSP2712 | B.1.1.7 | 5/10/21 | accine breakthroug | 99.7 | 1489 | hCoV-19/USA/PA-VSP2712/2021 | OK246441 |
| VSP2714 | B.1.1.7 | 5/10/21 | accine breakthroug | 99.7 | 1734 | hCoV-19/USA/PA-VSP2714/2021 | OK246442 |
| VSP2715 | P.1 | 5/10/21 | accine breakthroug | 99.7 | 1743 | hCoV-19/USA/NJ-VSP2715/2021 | OK246443 |
| VSP2716 | B.1.1.7 | 5/10/21 | accine breakthroug | 99.7 | 859 | hCoV-19/USA/PA-VSP2716/2021 | OK246444 |
| VSP2717 | B.1.1.7 | 5/10/21 | accine breakthroug | 99.6 | 1516 | hCoV-19/USA/PA-VSP2717/2021 | OK246445 |
| VSP2721 | B.1.1.7 | 5/10/21 | surveillance | 99.7 | 1530 | hCoV-19/USA/PA-VSP2721/2021 | OK246449 |
| VSP2722 | B.1.1.7 | 5/10/21 | surveillance | 99.7 | 1293 | hCoV-19/USA/PA-VSP2722/2021 | OK246450 |
| VSP2723 | B.1.1.7 | 5/10/21 | surveillance | 99.7 | 1935 | hCoV-19/USA/PA-VSP2723/2021 | OK246451 |
| VSP2724 | B.1.1.7 | 5/10/21 | surveillance | 99.6 | 1505 | hCoV-19/USA/PA-VSP2724/2021 | OK246452 |
| VSP2725 | B.1.1.7 | 5/10/21 | surveillance | 99.2 | 471 | hCoV-19/USA/PA-VSP2725/2021 | OK246453 |
| VSP2726 | B.1.1.7 | 5/10/21 | surveillance | 99.7 | 1724 | hCoV-19/USA/PA-VSP2726/2021 | OK246454 |

| | | | | | | | |
|---|---|---|---|---|---|---|---|
| VSP2727 | B.1.617.2 | 5/10/21 | surveillance | 99.7 | 1770 | hCoV-19/USA/PA-VSP2727/2021 | OK246455 |
| VSP2728 | B.1.1.7 | 5/10/21 | surveillance | 99.7 | 2234 | hCoV-19/USA/PA-VSP2728/2021 | OK246456 |
| VSP2729 | B.1.1.7 | 5/10/21 | surveillance | 99.7 | 2110 | hCoV-19/USA/PA-VSP2729/2021 | OK246457 |
| VSP2730 | B.1.1.7 | 5/10/21 | surveillance | 99.6 | 1785 | hCoV-19/USA/PA-VSP2730/2021 | OK246458 |
| VSP2731 | B.1.1.7 | 5/10/21 | surveillance | 99.5 | 804 | hCoV-19/USA/PA-VSP2731/2021 | OK246459 |
| VSP2732 | B.1.526 | 5/10/21 | surveillance | 99.4 | 2084 | hCoV-19/USA/PA-VSP2732/2021 | OK246460 |
| VSP2733 | B.1.1.7 | 5/10/21 | surveillance | 99.6 | 968 | hCoV-19/USA/PA-VSP2733/2021 | OK246461 |
| VSP2735 | B.1.525 | 5/10/21 | surveillance | 98.4 | 1792 | hCoV-19/USA/PA-VSP2735/2021 | |
| VSP2737 | B.1.526 | 5/10/21 | surveillance | 99 | 577 | hCoV-19/USA/PA-VSP2737/2021 | OK246462 |
| VSP2738 | B.1.1.7 | 5/10/21 | surveillance | 99.7 | 3225 | hCoV-19/USA/NJ-VSP2738/2021 | OK246463 |
| VSP2739 | P.1 | 5/10/21 | surveillance | 99.7 | 3006 | hCoV-19/USA/NJ-VSP2739/2021 | OK246464 |
| VSP2740 | B.1.1.7 | 5/10/21 | surveillance | 99.7 | 549 | hCoV-19/USA/NJ-VSP2740/2021 | OK246465 |
| VSP2741 | B.1.526 | 5/10/21 | surveillance | 99.7 | 2418 | hCoV-19/USA/NJ-VSP2741/2021 | OK246466 |
| VSP2742 | B.1.526 | 5/10/21 | surveillance | 99.7 | 2768 | hCoV-19/USA/NJ-VSP2742/2021 | OK246467 |
| VSP2743 | B.1.1.7 | 5/10/21 | surveillance | 99 | 645 | hCoV-19/USA/NJ-VSP2743/2021 | OK246468 |
| VSP2744 | B.1.1.7 | 5/10/21 | surveillance | 99.7 | 1712 | hCoV-19/USA/PA-VSP2744/2021 | OK246469 |
| VSP2745 | B.1.1.7 | 5/10/21 | surveillance | 99.1 | 1735 | hCoV-19/USA/PA-VSP2745/2021 | OK246470 |
| VSP2746 | B.1.1.7 | 5/10/21 | surveillance | 99.6 | 3044 | hCoV-19/USA/PA-VSP2746/2021 | OK246471 |
| VSP2747 | P.1 | 5/10/21 | surveillance | 99.7 | 2400 | hCoV-19/USA/PA-VSP2747/2021 | OK246472 |
| VSP2748 | B.1.1.7 | 5/10/21 | surveillance | 99.1 | 987 | hCoV-19/USA/PA-VSP2748/2021 | OK246473 |
| VSP2749 | B.1.1.7 | 5/10/21 | surveillance | 99.5 | 3288 | hCoV-19/USA/NJ-VSP2749/2021 | OK246474 |
| VSP2750 | B.1.1.7 | 5/10/21 | surveillance | 98.5 | 120 | hCoV-19/USA/PA-VSP2750/2021 | OK246475 |
| VSP2751 | B.1.1.7 | 5/10/21 | surveillance | 99.6 | 3355 | hCoV-19/USA/PA-VSP2751/2021 | OK246476 |
| VSP2752 | B.1.1.7 | 5/10/21 | surveillance | 99.7 | 2408 | hCoV-19/USA/NJ-VSP2752/2021 | OK246477 |
| VSP2753 | B.1.1.7 | 5/10/21 | surveillance | 99.7 | 3097 | hCoV-19/USA/PA-VSP2753/2021 | OK246478 |
| VSP2755 | B.1.526 | 5/10/21 | surveillance | 99.1 | 624 | hCoV-19/USA/PA-VSP2755/2021 | OK246479 |
| VSP2756 | B.1.1.7 | 5/10/21 | surveillance | 99.7 | 1626 | hCoV-19/USA/PA-VSP2756/2021 | OK246480 |
| VSP2757 | B.1.1.7 | 5/10/21 | surveillance | 99.7 | 3336 | hCoV-19/USA/NJ-VSP2757/2021 | OK246481 |
| VSP2759 | B.1.1.7 | 5/10/21 | surveillance | 99.7 | 2897 | hCoV-19/USA/PA-VSP2759/2021 | OK246482 |
| VSP2760 | B.1.1.7 | 5/10/21 | surveillance | 99.1 | 2109 | hCoV-19/USA/PA-VSP2760/2021 | OK246483 |
| VSP2761 | B.1.526 | 5/10/21 | surveillance | 99.6 | 1322 | hCoV-19/USA/PA-VSP2761/2021 | OK246484 |
| VSP2762 | B.1.526 | 5/10/21 | surveillance | 99.6 | 2361 | hCoV-19/USA/PA-VSP2762/2021 | OK246485 |
| VSP2763 | B.1.1.7 | 5/10/21 | surveillance | 99.7 | 2974 | hCoV-19/USA/PA-VSP2763/2021 | OK246486 |
| VSP2767 | B.1.526 | 5/10/21 | surveillance | 99.1 | 1862 | hCoV-19/USA/PA-VSP2767/2021 | OK246487 |
| VSP2768 | B.1.1.519 | 5/10/21 | surveillance | 99.7 | 1042 | hCoV-19/USA/PA-VSP2768/2021 | OK246488 |
| VSP2769 | B.1.1.7 | 5/10/21 | surveillance | 99.7 | 2195 | hCoV-19/USA/PA-VSP2769/2021 | OK246489 |
| VSP2770 | B.1.1.7 | 5/10/21 | surveillance | 99.1 | 284 | hCoV-19/USA/PA-VSP2770/2021 | OK246490 |
| VSP2771 | B.1.526 | 5/10/21 | surveillance | 99.7 | 2827 | hCoV-19/USA/PA-VSP2771/2021 | OK246491 |
| VSP2772 | B.1.1.7 | 5/10/21 | surveillance | 99.7 | 2787 | hCoV-19/USA/PA-VSP2772/2021 | OK246492 |
| VSP2773 | B.1.1.7 | 5/10/21 | surveillance | 99.7 | 3055 | hCoV-19/USA/PA-VSP2773/2021 | OK246493 |
| VSP2774 | B.1.526 | 5/10/21 | surveillance | 99.7 | 1047 | hCoV-19/USA/PA-VSP2774/2021 | OK246494 |
| VSP2776 | B.1.1.7 | 5/10/21 | surveillance | 99.7 | 2908 | hCoV-19/USA/PA-VSP2776/2021 | OK246495 |
| VSP2777 | B.1.1.7 | 5/10/21 | surveillance | 99.7 | 1406 | hCoV-19/USA/PA-VSP2777/2021 | OK246496 |
| VSP2778 | B.1.1.7 | 5/10/21 | surveillance | 99.7 | 2467 | hCoV-19/USA/PA-VSP2778/2021 | OK246497 |
| VSP2779 | B.1.526 | 5/10/21 | surveillance | 99.7 | 3023 | hCoV-19/USA/PA-VSP2779/2021 | OK246498 |
| VSP2780 | B.1.526 | 5/10/21 | surveillance | 99.7 | 3839 | hCoV-19/USA/PA-VSP2780/2021 | OK246499 |
| VSP2783 | B.1.526 | 5/10/21 | surveillance | 99.7 | 3065 | hCoV-19/USA/NJ-VSP2783/2021 | OK246500 |

| | | | | | | | |
|---|---|---|---|---|---|---|---|
| VSP2784 | B.1.637 | 5/10/21 | surveillance | 99.7 | 2792 | hCoV-19/USA/NJ-VSP2784/2021 | OK246501 |
| VSP2785 | B.1.1.7 | 5/10/21 | surveillance | 99.7 | 1060 | hCoV-19/USA/PA-VSP2785/2021 | OK246502 |
| VSP2786 | B.1.1.7 | 5/10/21 | surveillance | 99.1 | 676 | hCoV-19/USA/PA-VSP2786/2021 | OK246503 |
| VSP2787 | B.1.1.7 | 5/10/21 | surveillance | 99.5 | 1049 | hCoV-19/USA/PA-VSP2787/2021 | |
| VSP2788 | B.1.1.7 | 5/10/21 | surveillance | 99.7 | 2687 | hCoV-19/USA/PA-VSP2788/2021 | OK246504 |
| VSP2789 | B.1.1.7 | 5/10/21 | surveillance | 99.7 | 1970 | hCoV-19/USA/PA-VSP2789/2021 | OK246505 |
| VSP2790 | B.1.1.7 | 5/10/21 | surveillance | 99.6 | 906 | hCoV-19/USA/DE-VSP2790/2021 | OK246506 |
| VSP2791 | B.1.526 | 5/10/21 | surveillance | 99.7 | 2238 | hCoV-19/USA/PA-VSP2791/2021 | OK246507 |
| VSP2792 | B.1.1.7 | 5/10/21 | surveillance | 99.7 | 973 | hCoV-19/USA/PA-VSP2792/2021 | OK246508 |
| VSP2793 | B.1.1.7 | 5/10/21 | surveillance | 99.7 | 3365 | hCoV-19/USA/PA-VSP2793/2021 | OK246509 |
| VSP2794 | B.1.526 | 5/10/21 | surveillance | 99.7 | 1753 | hCoV-19/USA/PA-VSP2794/2021 | OK246510 |
| VSP2795 | B.1.526 | 5/10/21 | surveillance | 99.7 | 1025 | hCoV-19/USA/PA-VSP2795/2021 | OK246511 |
| VSP2796 | B.1.526 | 5/10/21 | surveillance | 99.7 | 4497 | hCoV-19/USA/PA-VSP2796/2021 | OK246512 |
| VSP2797 | B.1.1.7 | 5/10/21 | surveillance | 99.7 | 3643 | hCoV-19/USA/PA-VSP2797/2021 | OK246513 |
| VSP2798 | B.1.1.7 | 5/10/21 | surveillance | 99.7 | 4078 | hCoV-19/USA/PA-VSP2798/2021 | OK246514 |
| VSP2799 | B.1.1.7 | 5/10/21 | surveillance | 99.3 | 364 | hCoV-19/USA/PA-VSP2799/2021 | OK246515 |
| VSP2803 | B.1.1.7 | 5/18/21 | accine breakthroug | 99.7 | 64319 | hCoV-19/USA/PA-VSP2803/2021 | OK246516 |
| VSP2805 | B.1.1.7 | 5/18/21 | surveillance | 99.7 | 2215 | hCoV-19/USA/PA-VSP2805/2021 | OK246518 |
| VSP2806 | B.1.1.7 | 5/18/21 | surveillance | 99.7 | 5888 | hCoV-19/USA/DE-VSP2806/2021 | OK246519 |
| VSP2807 | B.1.1.7 | 5/18/21 | surveillance | 99.7 | 3251 | hCoV-19/USA/PA-VSP2807/2021 | OK246520 |
| VSP2808 | B.1.1.7 | 5/18/21 | surveillance | 99.7 | 4046 | hCoV-19/USA/PA-VSP2808/2021 | OK246521 |
| VSP2809 | B.1.621 | 5/18/21 | surveillance | 99.7 | 1616 | hCoV-19/USA/PA-VSP2809/2021 | OK246522 |
| VSP2811 | B.1.1.7 | 5/18/21 | surveillance | 99.7 | 15875 | hCoV-19/USA/PA-VSP2811/2021 | OK246523 |
| VSP2812 | B.1.621 | 5/18/21 | surveillance | 99.7 | 5967 | hCoV-19/USA/PA-VSP2812/2021 | OK246524 |
| VSP2813 | B.1.1.7 | 5/18/21 | surveillance | 99.7 | 14255 | hCoV-19/USA/PA-VSP2813/2021 | OK246525 |
| VSP2815 | B.1.1.7 | 5/18/21 | surveillance | 99.7 | 7964 | hCoV-19/USA/PA-VSP2815/2021 | OK246526 |
| VSP2816 | B.1.1.7 | 5/18/21 | surveillance | 99.7 | 17677 | hCoV-19/USA/PA-VSP2816/2021 | OK246527 |
| VSP2817 | B.1.1.7 | 5/18/21 | surveillance | 99 | 61634 | hCoV-19/USA/PA-VSP2817/2021 | OK246528 |
| VSP2818 | B.1.1.7 | 5/18/21 | surveillance | 99.5 | 661 | hCoV-19/USA/PA-VSP2818/2021 | OK246529 |
| VSP2819 | B.1.526 | 5/18/21 | surveillance | 99.7 | 3396 | hCoV-19/USA/NJ-VSP2819/2021 | OK246530 |
| VSP2820 | B.1.1.7 | 5/18/21 | surveillance | 99.7 | 2519 | hCoV-19/USA/PA-VSP2820/2021 | OK246531 |
| VSP2822 | B.1.1.7 | 5/18/21 | surveillance | 99.6 | 1278 | hCoV-19/USA/PA-VSP2822/2021 | OK246532 |
| VSP2823 | B.1.526 | 5/18/21 | surveillance | 99.7 | 4278 | hCoV-19/USA/PA-VSP2823/2021 | OK246533 |
| VSP2824 | B.1.526 | 5/18/21 | surveillance | 99.7 | 2361 | hCoV-19/USA/PA-VSP2824/2021 | OK246534 |
| VSP2825 | B.1.526 | 5/18/21 | surveillance | 99.1 | 1261 | hCoV-19/USA/PA-VSP2825/2021 | OK246535 |
| VSP2826 | P.1 | 5/18/21 | surveillance | 99.5 | 1007 | hCoV-19/USA/PA-VSP2826/2021 | OK246536 |
| VSP2827 | B.1.1.7 | 5/18/21 | surveillance | 99.7 | 992 | hCoV-19/USA/PA-VSP2827/2021 | OK246537 |
| VSP2828 | B.1.526 | 5/18/21 | surveillance | 99 | 129 | hCoV-19/USA/PA-VSP2828/2021 | OK246538 |
| VSP2829 | B.1.1.7 | 5/18/21 | surveillance | 99.6 | 930 | hCoV-19/USA/PA-VSP2829/2021 | OK246539 |
| VSP2830 | P.1 | 5/18/21 | surveillance | 99.6 | 2790 | hCoV-19/USA/PA-VSP2830/2021 | OK246540 |
| VSP2831 | B.1.1.7 | 5/18/21 | surveillance | 99.1 | 681 | hCoV-19/USA/PA-VSP2831/2021 | OK246541 |
| VSP2832 | B.1.1.7 | 5/18/21 | surveillance | 99.7 | 999 | hCoV-19/USA/PA-VSP2832/2021 | OK246542 |
| VSP2833 | B.1.526 | 5/18/21 | surveillance | 99.7 | 3923 | hCoV-19/USA/PA-VSP2833/2021 | OK246543 |
| VSP2834 | B.1.1.7 | 5/18/21 | surveillance | 99.7 | 2907 | hCoV-19/USA/PA-VSP2834/2021 | OK246544 |
| VSP2836 | P.1.10 | 5/18/21 | surveillance | 99.7 | 734 | hCoV-19/USA/PA-VSP2836/2021 | OK246545 |
| VSP2837 | B.1.526 | 5/18/21 | surveillance | 99.7 | 3925 | hCoV-19/USA/PA-VSP2837/2021 | OK246546 |
| VSP2838 | B.1.1.7 | 5/18/21 | surveillance | 99.1 | 298 | hCoV-19/USA/PA-VSP2838/2021 | OK246547 |

| | | | | | | | |
|---|---|---|---|---|---|---|---|
| VSP2839 | B.1.1.7 | 5/18/21 | surveillance | 96.7 | 238 | hCoV-19/USA/PA-VSP2839/2021 | OK246548 |
| VSP2840 | B.1.637 | 5/18/21 | surveillance | 98.7 | 4398 | hCoV-19/USA/PA-VSP2840/2021 | OK246549 |
| VSP2842 | B.1.2 | 5/18/21 | surveillance | 99.7 | 3857 | hCoV-19/USA/PA-VSP2842/2021 | OK246550 |
| VSP2843 | B.1.526 | 5/18/21 | surveillance | 99.7 | 2748 | hCoV-19/USA/PA-VSP2843/2021 | OK246551 |
| VSP2844 | B.1.526 | 5/18/21 | surveillance | 98.5 | 860 | hCoV-19/USA/PA-VSP2844/2021 | |
| VSP2845 | B.1.1.7 | 5/18/21 | surveillance | 99.7 | 1835 | hCoV-19/USA/PA-VSP2845/2021 | OK246552 |
| VSP2846 | B.1.525 | 5/18/21 | surveillance | 99.4 | 5486 | hCoV-19/USA/PA-VSP2846/2021 | |
| VSP2847 | B.1.526 | 5/18/21 | surveillance | 99.7 | 1538 | hCoV-19/USA/PA-VSP2847/2021 | OK246553 |
| VSP2848 | B.1.1.7 | 5/18/21 | surveillance | 99.7 | 882 | hCoV-19/USA/PA-VSP2848/2021 | OK246554 |
| VSP2849 | B.1.1.7 | 5/18/21 | surveillance | 99.1 | 206 | hCoV-19/USA/PA-VSP2849/2021 | OK246555 |
| VSP2850 | B.1.525 | 5/18/21 | surveillance | 99.5 | 2231 | hCoV-19/USA/PA-VSP2850/2021 | |
| VSP2851 | B.1.526 | 5/18/21 | surveillance | 99.7 | 4653 | hCoV-19/USA/PA-VSP2851/2021 | OK246556 |
| VSP2853 | B.1.526 | 5/18/21 | surveillance | 99.6 | 1906 | hCoV-19/USA/PA-VSP2853/2021 | OK246557 |
| VSP2854 | B.1.1.7 | 5/18/21 | surveillance | 99.1 | 2055 | hCoV-19/USA/PA-VSP2854/2021 | OK246558 |
| VSP2857 | B.1.1.7 | 5/18/21 | surveillance | 99.7 | 2430 | hCoV-19/USA/PA-VSP2857/2021 | OK246559 |
| VSP2858 | B.1.526 | 5/18/21 | surveillance | 99.7 | 68262 | hCoV-19/USA/PA-VSP2858/2021 | OK246560 |
| VSP2859 | B.1.1.7 | 5/18/21 | surveillance | 99.7 | 57833 | hCoV-19/USA/PA-VSP2859/2021 | OK246561 |
| VSP2860 | P.1.2 | 5/18/21 | surveillance | 99.7 | 4359 | hCoV-19/USA/PA-VSP2860/2021 | OK246562 |
| VSP2861 | B.1.637 | 5/18/21 | surveillance | 99.7 | 3271 | hCoV-19/USA/PA-VSP2861/2021 | OK246563 |
| VSP2863 | B.1.1.7 | 5/18/21 | surveillance | 99.7 | 2271 | hCoV-19/USA/PA-VSP2863/2021 | OK246564 |
| VSP2864 | B.1.1.7 | 5/18/21 | surveillance | 99.7 | 3689 | hCoV-19/USA/PA-VSP2864/2021 | OK246565 |
| VSP2865 | B.1.637 | 5/18/21 | surveillance | 99.7 | 2398 | hCoV-19/USA/PA-VSP2865/2021 | OK246566 |
| VSP2866 | B.1.621 | 5/18/21 | surveillance | 99.7 | 1759 | hCoV-19/USA/PA-VSP2866/2021 | OK246567 |
| VSP2867 | B.1.526 | 5/18/21 | surveillance | 99.7 | 2669 | hCoV-19/USA/PA-VSP2867/2021 | OK246568 |
| VSP2868 | B.1.1.7 | 5/18/21 | surveillance | 99.7 | 673 | hCoV-19/USA/PA-VSP2868/2021 | OK246569 |
| VSP2869 | B.1.1.7 | 5/18/21 | surveillance | 99.7 | 2995 | hCoV-19/USA/PA-VSP2869/2021 | OK246570 |
| VSP2870 | B.1.1.7 | 5/18/21 | surveillance | 99.7 | 3680 | hCoV-19/USA/PA-VSP2870/2021 | OK246571 |
| VSP2871 | B.1.1.7 | 5/18/21 | accine breakthroug | 99.7 | 5156 | hCoV-19/USA/PA-VSP2871/2021 | OK246572 |
| VSP2872 | AY.10 | 5/18/21 | surveillance | 99.7 | 4945 | hCoV-19/USA/PA-VSP2872/2021 | OK246573 |
| VSP2873 | B.1.1.7 | 5/18/21 | surveillance | 99.7 | 1985 | hCoV-19/USA/PA-VSP2873/2021 | OK246574 |
| VSP2874 | B.1.1.7 | 5/18/21 | surveillance | 99.7 | 3740 | hCoV-19/USA/PA-VSP2874/2021 | OK246575 |
| VSP2875 | B.1.1.7 | 5/18/21 | surveillance | 99.7 | 4041 | hCoV-19/USA/PA-VSP2875/2021 | OK246576 |
| VSP2876 | B.1.1.7 | 5/18/21 | surveillance | 99.7 | 2990 | hCoV-19/USA/PA-VSP2876/2021 | OK246577 |
| VSP2877 | B.1.1.7 | 5/18/21 | surveillance | 99.7 | 3248 | hCoV-19/USA/PA-VSP2877/2021 | OK246578 |
| VSP2878 | B.1.1.7 | 5/18/21 | surveillance | 99.7 | 4013 | hCoV-19/USA/PA-VSP2878/2021 | OK246579 |
| VSP2879 | B.1.1.7 | 5/18/21 | surveillance | 99.7 | 2456 | hCoV-19/USA/PA-VSP2879/2021 | OK246580 |
| VSP2880 | B.1.1.7 | 5/18/21 | surveillance | 99.7 | 1846 | hCoV-19/USA/PA-VSP2880/2021 | OK246581 |
| VSP2885 | B.1.1.7 | 5/24/21 | surveillance | 99.7 | 2014 | hCoV-19/USA/VSP2885/2021 | MZ512918 |
| VSP2886 | B.1.1.7 | 5/24/21 | surveillance | 99.7 | 1331 | hCoV-19/USA/VSP2886/2021 | MZ512919 |
| VSP2887 | B.1.526 | 5/24/21 | surveillance | 99.7 | 2194 | hCoV-19/USA/VSP2887/2021 | MZ512920 |
| VSP2888 | B.1.1.7 | 5/24/21 | surveillance | 99.7 | 2132 | hCoV-19/USA/VSP2888/2021 | MZ512921 |
| VSP2889 | B.1.637 | 5/24/21 | surveillance | 99.3 | 329 | hCoV-19/USA/VSP2889/2021 | MZ512922 |
| VSP2890 | B.1.1.7 | 5/24/21 | surveillance | 99.7 | 1960 | hCoV-19/USA/VSP2890/2021 | MZ512923 |
| VSP2891 | B.1.1.7 | 5/24/21 | surveillance | 99.7 | 1989 | hCoV-19/USA/VSP2891/2021 | MZ512924 |
| VSP2892 | B.1.1.7 | 5/24/21 | surveillance | 99.7 | 1791 | hCoV-19/USA/VSP2892/2021 | MZ512925 |
| VSP2893 | B.1.1.7 | 5/24/21 | surveillance | 99.7 | 2797 | hCoV-19/USA/VSP2893/2021 | MZ512926 |
| VSP2894 | B.1.1.7 | 5/24/21 | surveillance | 99.1 | 3043 | hCoV-19/USA/VSP2894/2021 | MZ512927 |

| | | | | | | | |
|---|---|---|---|---|---|---|---|
| VSP2895 | B.1.1.7 | 5/24/21 | surveillance | 99.7 | 2727 | hCoV-19/USA/VSP2895/2021 | MZ512928 |
| VSP2896 | B.1.1.7 | 5/24/21 | surveillance | 99.3 | 726 | hCoV-19/USA/VSP2896/2021 | MZ512929 |
| VSP2897 | B.1.1.7 | 5/24/21 | surveillance | 99.3 | 538 | hCoV-19/USA/VSP2897/2021 | MZ512930 |
| VSP2898 | B.1.1.7 | 5/24/21 | surveillance | 99.7 | 1081 | hCoV-19/USA/VSP2898/2021 | MZ512931 |
| VSP2899 | R.1 | 5/24/21 | accine breakthroug | 99.7 | 2294 | hCoV-19/USA/VSP2899/2021 | MZ512932 |
| VSP2900 | P.1 | 5/24/21 | surveillance | 99.7 | 1191 | hCoV-19/USA/VSP2900/2021 | MZ512933 |
| VSP2901 | B.1.1.7 | 5/24/21 | surveillance | 99.7 | 1568 | hCoV-19/USA/VSP2901/2021 | MZ512934 |
| VSP2902 | B.1.1.7 | 5/24/21 | surveillance | 99.7 | 2456 | hCoV-19/USA/VSP2902/2021 | MZ512935 |
| VSP2903 | P.1.2 | 5/24/21 | surveillance | 99.7 | 1510 | hCoV-19/USA/VSP2903/2021 | MZ512936 |
| VSP2904 | B.1.1.7 | 5/24/21 | surveillance | 99.7 | 2293 | hCoV-19/USA/VSP2904/2021 | MZ512937 |
| VSP2906 | B.1.1.7 | 5/24/21 | surveillance | 99.7 | 2312 | hCoV-19/USA/VSP2906/2021 | MZ512938 |
| VSP2907 | B.1.1.7 | 5/24/21 | surveillance | 99.7 | 2309 | hCoV-19/USA/VSP2907/2021 | MZ512939 |
| VSP2908 | B.1.526 | 5/24/21 | surveillance | 99.7 | 2671 | hCoV-19/USA/VSP2908/2021 | MZ512940 |
| VSP2909 | B.1.1.7 | 5/24/21 | surveillance | 99.3 | 463 | hCoV-19/USA/VSP2909/2021 | MZ512941 |
| VSP2910 | P.1 | 5/24/21 | surveillance | 99.6 | 2069 | hCoV-19/USA/VSP2910/2021 | MZ512942 |
| VSP2911 | B.1.1.7 | 5/24/21 | surveillance | 99.7 | 1933 | hCoV-19/USA/VSP2911/2021 | MZ512943 |
| VSP2912 | B.1.526 | 5/24/21 | surveillance | 99.7 | 2699 | hCoV-19/USA/VSP2912/2021 | MZ512944 |
| VSP2914 | B.1.526 | 5/24/21 | surveillance | 99.7 | 892 | hCoV-19/USA/VSP2914/2021 | MZ512945 |
| VSP2915 | P.1 | 5/24/21 | surveillance | 99.7 | 2285 | hCoV-19/USA/VSP2915/2021 | MZ512946 |
| VSP2916 | B.1.1.7 | 5/24/21 | surveillance | 99.7 | 1675 | hCoV-19/USA/VSP2916/2021 | MZ512947 |
| VSP2917 | B.1.525 | 5/24/21 | surveillance | 99.5 | 2331 | hCoV-19/USA/VSP2917/2021 | MZ512948 |
| VSP2918 | B.1.526 | 5/24/21 | surveillance | 99.3 | 797 | hCoV-19/USA/VSP2918/2021 | MZ512949 |
| VSP2921 | B.1.1.7 | 5/24/21 | surveillance | 99.6 | 1302 | hCoV-19/USA/VSP2921/2021 | MZ512950 |
| VSP2922 | B.1.526 | 5/24/21 | surveillance | 99.5 | 519 | hCoV-19/USA/VSP2922/2021 | MZ512951 |
| VSP2923 | B.1.1.7 | 5/24/21 | surveillance | 99.7 | 1008 | hCoV-19/USA/VSP2923/2021 | MZ512952 |
| VSP2924 | B.1.1.7 | 5/24/21 | surveillance | 99.6 | 1200 | hCoV-19/USA/VSP2924/2021 | MZ512953 |
| VSP2925 | B.1.637 | 5/24/21 | surveillance | 98.3 | 208 | hCoV-19/USA/VSP2925/2021 | MZ512954 |
| VSP2926 | B.1.1.7 | 5/24/21 | surveillance | 99.7 | 1453 | hCoV-19/USA/VSP2926/2021 | MZ512955 |
| VSP2929 | B.1.1.7 | 5/24/21 | surveillance | 99.7 | 1427 | hCoV-19/USA/VSP2929/2021 | MZ512956 |
| VSP2930 | P.1.10 | 5/24/21 | surveillance | 99.7 | 3092 | hCoV-19/USA/VSP2930/2021 | MZ512957 |
| VSP2931 | B.1.1.7 | 5/24/21 | surveillance | 99.7 | 1023 | hCoV-19/USA/VSP2931/2021 | MZ512958 |
| VSP2932 | B.1.1.7 | 5/24/21 | surveillance | 99.7 | 535 | hCoV-19/USA/VSP2932/2021 | MZ512959 |
| VSP2933 | B.1.526 | 5/24/21 | surveillance | 99.7 | 2195 | hCoV-19/USA/VSP2933/2021 | MZ512960 |
| VSP2934 | B.1.637 | 5/24/21 | surveillance | 98.9 | 2653 | hCoV-19/USA/VSP2934/2021 | MZ512961 |
| VSP2936 | B.1.617.2 | 5/24/21 | surveillance | 99.7 | 3212 | hCoV-19/USA/VSP2936/2021 | MZ512963 |
| VSP2937 | B.1.1.7 | 5/24/21 | surveillance | 99.7 | 1934 | hCoV-19/USA/VSP2937/2021 | MZ512964 |
| VSP2938 | B.1.617.2 | 5/24/21 | surveillance | 97.8 | 848 | hCoV-19/USA/VSP2938/2021 | MZ512965 |
| VSP2939 | B.1.1.7 | 5/24/21 | surveillance | 99.7 | 2317 | hCoV-19/USA/VSP2939/2021 | MZ512966 |
| VSP2940 | P.1 | 5/24/21 | surveillance | 99.7 | 3097 | hCoV-19/USA/VSP2940/2021 | MZ512967 |
| VSP2942 | B.1.1.7 | 5/24/21 | surveillance | 99.7 | 2535 | hCoV-19/USA/VSP2942/2021 | MZ512968 |
| VSP2943 | B.1.1.7 | 5/24/21 | surveillance | 98.9 | 765 | hCoV-19/USA/VSP2943/2021 | MZ512969 |
| VSP2944 | B.1.1.7 | 5/24/21 | surveillance | 99.7 | 2580 | hCoV-19/USA/VSP2944/2021 | MZ512970 |
| VSP2945 | B.1.214.2 | 5/24/21 | surveillance | 99.7 | 2289 | hCoV-19/USA/VSP2945/2021 | MZ512971 |
| VSP2946 | B.1.1.7 | 5/24/21 | surveillance | 99.7 | 3268 | hCoV-19/USA/VSP2946/2021 | MZ512972 |
| VSP2947 | B.1.526 | 5/24/21 | surveillance | 99.6 | 842 | hCoV-19/USA/VSP2947/2021 | MZ512973 |
| VSP2948 | B.1.526 | 5/24/21 | surveillance | 99.7 | 4074 | hCoV-19/USA/VSP2948/2021 | MZ512974 |
| VSP2949 | B.1.1.7 | 5/24/21 | surveillance | 99.7 | 2808 | hCoV-19/USA/VSP2949/2021 | MZ512975 |

| | | | | | | | |
|---|---|---|---|---|---|---|---|
| VSP2950 | P.1 | 5/24/21 | surveillance | 99.7 | 2552 | hCoV-19/USA/VSP2950/2021 | MZ512976 |
| VSP2951 | B.1.1.7 | 5/24/21 | surveillance | 99.7 | 3538 | hCoV-19/USA/VSP2951/2021 | MZ512977 |
| VSP2954 | B.1.1.7 | 5/24/21 | surveillance | 99.7 | 3229 | hCoV-19/USA/VSP2954/2021 | MZ512978 |
| VSP2955 | B.1.617.2 | 5/24/21 | surveillance | 99.6 | 2543 | hCoV-19/USA/VSP2955/2021 | MZ512979 |
| VSP2956 | B.1.1.7 | 5/24/21 | surveillance | 99.7 | 3467 | hCoV-19/USA/VSP2956/2021 | MZ512980 |
| VSP2957 | B.1.1.7 | 5/24/21 | surveillance | 99.7 | 987 | hCoV-19/USA/VSP2957/2021 | MZ512981 |
| VSP2958 | B.1.1.7 | 5/24/21 | surveillance | 99.7 | 874 | hCoV-19/USA/VSP2958/2021 | MZ512982 |
| VSP2959 | B.1.526 | 5/24/21 | surveillance | 99.7 | 3003 | hCoV-19/USA/VSP2959/2021 | MZ512983 |
| VSP2960 | B.1.1.7 | 5/24/21 | surveillance | 99.7 | 1220 | hCoV-19/USA/VSP2960/2021 | MZ512984 |
| VSP2962 | B.1.1.7 | 5/24/21 | surveillance | 99.1 | 2657 | hCoV-19/USA/VSP2962/2021 | MZ512985 |
| VSP2963 | B.1.1.7 | 5/24/21 | surveillance | 99.7 | 2625 | hCoV-19/USA/VSP2963/2021 | MZ512986 |
| VSP2965 | B.1.1.7 | 5/24/21 | surveillance | 99.7 | 1477 | hCoV-19/USA/VSP2965/2021 | MZ512987 |
| VSP3001 | B.1.311 | 2/3/21 | surveillance | 98.8 | 1535 | hCoV-19/USA/PA-VSP3001/2021 | OK246582 |
| VSP3004 | B.1.2 | 2/3/21 | surveillance | 97.4 | 2264 | hCoV-19/USA/NY-VSP3004/2021 | |
| VSP3005 | B.1.1 | 2/3/21 | surveillance | 99.1 | 1611 | hCoV-19/USA/PA-VSP3005/2021 | OK246583 |
| VSP3006 | B.1.2 | 2/3/21 | surveillance | 99.7 | 1674 | hCoV-19/USA/PA-VSP3006/2021 | OK246584 |
| VSP3007 | B.1.588 | 2/3/21 | surveillance | 99.4 | 1997 | hCoV-19/USA/PA-VSP3007/2021 | OK246585 |
| VSP3008 | B.1.1 | 2/3/21 | surveillance | 99.4 | 1638 | hCoV-19/USA/PA-VSP3008/2021 | OK246586 |
| VSP3009 | B.1.588 | 2/3/21 | surveillance | 99.7 | 1315 | hCoV-19/USA/PA-VSP3009/2021 | OK246587 |
| VSP3010 | B.1 | 2/3/21 | surveillance | 98.7 | 310 | hCoV-19/USA/PA-VSP3010/2021 | OK246588 |
| VSP3011 | B.1.596 | 2/3/21 | surveillance | 99.2 | 1292 | hCoV-19/USA/PA-VSP3011/2021 | OK246589 |
| VSP3012 | B.1 | 2/3/21 | surveillance | 99.7 | 1379 | hCoV-19/USA/PA-VSP3012/2021 | OK246590 |
| VSP3013 | B.1.2 | 2/3/21 | surveillance | 98.9 | 2046 | hCoV-19/USA/PA-VSP3013/2021 | OK246591 |
| VSP3014 | B.1.1.348 | 2/3/21 | surveillance | 99.4 | 973 | hCoV-19/USA/PA-VSP3014/2021 | OK246592 |
| VSP3015 | B.1 | 2/3/21 | surveillance | 99.7 | 2033 | hCoV-19/USA/PA-VSP3015/2021 | OK246593 |
| VSP3016 | B.1.1.519 | 2/3/21 | surveillance | 99.4 | 1721 | hCoV-19/USA/PA-VSP3016/2021 | OK246594 |
| VSP3017 | B.1.1 | 2/3/21 | surveillance | 99.3 | 2497 | hCoV-19/USA/PA-VSP3017/2021 | OK246595 |
| VSP3018 | B.1.1 | 2/3/21 | surveillance | 99 | 1732 | hCoV-19/USA/PA-VSP3018/2021 | OK246596 |
| VSP3019 | B.1.1.7 | 2/10/21 | surveillance | 99.5 | 1146 | hCoV-19/USA/PA-VSP3019/2021 | OK246597 |
| VSP3020 | B.1.1.519 | 2/10/21 | surveillance | 99.7 | 1575 | hCoV-19/USA/PA-VSP3020/2021 | OK246598 |
| VSP3021 | B.1 | 2/10/21 | surveillance | 99.6 | 1452 | hCoV-19/USA/PA-VSP3021/2021 | OK246599 |
| VSP3022 | B.1.2 | 2/10/21 | surveillance | 99.7 | 2095 | hCoV-19/USA/PA-VSP3022/2021 | OK246600 |
| VSP3023 | B.1.526 | 2/10/21 | surveillance | 99.6 | 1364 | hCoV-19/USA/PA-VSP3023/2021 | OK246601 |
| VSP3024 | B.1 | 2/10/21 | surveillance | 96.6 | 1508 | hCoV-19/USA/NJ-VSP3024/2021 | OK246602 |
| VSP3025 | B.1.1 | 2/10/21 | surveillance | 99.6 | 1743 | hCoV-19/USA/PA-VSP3025/2021 | OK246603 |
| VSP3026 | B.1.526 | 2/10/21 | surveillance | 99.5 | 2387 | hCoV-19/USA/PA-VSP3026/2021 | OK246604 |
| VSP3027 | B.1.1.519 | 2/10/21 | surveillance | 99.7 | 2163 | hCoV-19/USA/PA-VSP3027/2021 | OK246605 |
| VSP3028 | R.1 | 2/10/21 | surveillance | 99.5 | 1446 | hCoV-19/USA/PA-VSP3028/2021 | OK246606 |
| VSP3029 | B.1.311 | 2/10/21 | surveillance | 99.7 | 1844 | hCoV-19/USA/PA-VSP3029/2021 | OK246607 |
| VSP3031 | B.1.243 | 2/10/21 | surveillance | 99.7 | 3004 | hCoV-19/USA/PA-VSP3031/2021 | OK246608 |
| VSP3032 | B.1.637 | 3/3/21 | surveillance | 99.4 | 8999 | hCoV-19/USA/PA-VSP3032/2021 | OK246609 |
| VSP3034 | B.1.2 | 3/3/21 | surveillance | 99.6 | 1473 | hCoV-19/USA/PA-VSP3034/2021 | OK246610 |
| VSP3035 | B.1.2 | 3/3/21 | surveillance | 99.7 | 32628 | hCoV-19/USA/PA-VSP3035/2021 | OK246611 |
| VSP3036 | B.1.1 | 3/3/21 | surveillance | 99.6 | 401 | hCoV-19/USA/PA-VSP3036/2021 | OK246612 |
| VSP3037 | B.1.1.7 | 3/3/21 | surveillance | 99.6 | 5819 | hCoV-19/USA/PA-VSP3037/2021 | OK246613 |
| VSP3038 | B.1.1.7 | 3/3/21 | surveillance | 99.4 | 4969 | hCoV-19/USA/PA-VSP3038/2021 | OK246614 |
| VSP3039 | B.1.2 | 3/3/21 | surveillance | 99.7 | 11612 | hCoV-19/USA/PA-VSP3039/2021 | OK246615 |

| | | | | | | | |
|---|---|---|---|---|---|---|---|
| VSP3040 | B.1.1.7 | 3/3/21 | surveillance | 99.6 | 7386 | hCoV-19/USA/PA-VSP3040/2021 | OK246616 |
| VSP3042 | B.1.1 | 3/17/21 | surveillance | 99.7 | 2247 | hCoV-19/USA/PA-VSP3042/2021 | OK246617 |
| VSP3043 | B.1.1.7 | 3/17/21 | surveillance | 99.2 | 855 | hCoV-19/USA/PA-VSP3043/2021 | OK246618 |
| VSP3044 | B.1.637 | 3/17/21 | surveillance | 99.4 | 2247 | hCoV-19/USA/PA-VSP3044/2021 | OK246619 |
| VSP3045 | B.1.2 | 3/17/21 | surveillance | 99.5 | 1892 | hCoV-19/USA/PA-VSP3045/2021 | OK246620 |
| VSP3046 | R.1 | 3/17/21 | surveillance | 99.5 | 781 | hCoV-19/USA/PA-VSP3046/2021 | OK246621 |
| VSP3047 | B.1.1.7 | 3/16/21 | surveillance | 99.2 | 742 | hCoV-19/USA/PA-VSP3047/2021 | OK246622 |
| VSP3048 | B.1.1.7 | 3/16/21 | surveillance | 99.2 | 844 | hCoV-19/USA/PA-VSP3048/2021 | OK246623 |
| VSP3049 | B.1.1.7 | 3/17/21 | surveillance | 99.1 | 1405 | hCoV-19/USA/PA-VSP3049/2021 | OK246624 |
| VSP3050 | B.1.526 | 3/17/21 | surveillance | 99.5 | 1639 | hCoV-19/USA/PA-VSP3050/2021 | OK246625 |
| VSP3051 | B.1.1.7 | 3/17/21 | surveillance | 99 | 1073 | hCoV-19/USA/PA-VSP3051/2021 | OK246626 |
| VSP3052 | B.1.1.7 | 3/16/21 | surveillance | 99.3 | 578 | hCoV-19/USA/PA-VSP3052/2021 | OK246627 |
| VSP3053 | B.1.526 | 3/17/21 | surveillance | 99.3 | 290 | hCoV-19/USA/PA-VSP3053/2021 | OK246628 |
| VSP3054 | B.1.526 | 3/17/21 | surveillance | 98.9 | 677 | hCoV-19/USA/PA-VSP3054/2021 | OK246629 |
| VSP3055 | R.1 | 3/17/21 | surveillance | 98.7 | 132 | hCoV-19/USA/PA-VSP3055/2021 | OK246630 |
| VSP3056 | B.1.2 | 3/17/21 | surveillance | 99.7 | 1715 | hCoV-19/USA/PA-VSP3056/2021 | OK246631 |
| VSP3057 | B.1.1.7 | 3/17/21 | surveillance | 98.6 | 712 | hCoV-19/USA/PA-VSP3057/2021 | OK246632 |
| VSP3058 | B.1.1.7 | 3/24/21 | surveillance | 99.2 | 1519 | hCoV-19/USA/PA-VSP3058/2021 | OK246633 |
| VSP3059 | B.1.1.7 | 3/24/21 | surveillance | 99.2 | 1663 | hCoV-19/USA/PA-VSP3059/2021 | OK246634 |
| VSP3060 | B.1.1 | 3/24/21 | surveillance | 99.4 | 632 | hCoV-19/USA/PA-VSP3060/2021 | OK246635 |
| VSP3061 | B.1.429 | 3/24/21 | surveillance | 99.4 | 798 | hCoV-19/USA/PA-VSP3061/2021 | OK246636 |
| VSP3062 | B.1.526 | 3/24/21 | surveillance | 99.5 | 1628 | hCoV-19/USA/PA-VSP3062/2021 | OK246637 |
| VSP3063 | B.1.1.7 | 3/24/21 | surveillance | 99.3 | 1489 | hCoV-19/USA/PA-VSP3063/2021 | OK246638 |
| VSP3064 | B.1.526 | 3/24/21 | surveillance | 99.7 | 1616 | hCoV-19/USA/PA-VSP3064/2021 | OK246639 |
| VSP3065 | B.1.1.7 | 3/24/21 | surveillance | 99.3 | 563 | hCoV-19/USA/PA-VSP3065/2021 | OK246640 |
| VSP3066 | B.1.1.7 | 3/24/21 | surveillance | 99.5 | 1180 | hCoV-19/USA/PA-VSP3066/2021 | OK246641 |
| VSP3067 | B.1.243 | 3/24/21 | surveillance | 99.7 | 1218 | hCoV-19/USA/PA-VSP3067/2021 | OK246642 |
| VSP3068 | B.1.1.7 | 4/7/21 | surveillance | 99.3 | 1134 | hCoV-19/USA/PA-VSP3068/2021 | OK246643 |
| VSP3069 | B.1.1.7 | 4/7/21 | surveillance | 99 | 734 | hCoV-19/USA/PA-VSP3069/2021 | OK246644 |
| VSP3070 | B.1.1.7 | 4/7/21 | surveillance | 98.5 | 379 | hCoV-19/USA/PA-VSP3070/2021 | OK246645 |
| VSP3071 | B.1.526 | 4/7/21 | surveillance | 98.5 | 183 | hCoV-19/USA/PA-VSP3071/2021 | OK246646 |
| VSP3072 | P.1 | 4/14/21 | surveillance | 99 | 648 | hCoV-19/USA/PA-VSP3072/2021 | OK246647 |
| VSP3073 | B.1.1.7 | 4/14/21 | surveillance | 99.1 | 768 | hCoV-19/USA/PA-VSP3073/2021 | OK246648 |
| VSP3074 | B.1.1.7 | 4/14/21 | surveillance | 99.2 | 415 | hCoV-19/USA/PA-VSP3074/2021 | OK246649 |
| VSP3075 | B.1.525 | 4/14/21 | surveillance | 98.2 | 635 | hCoV-19/USA/PA-VSP3075/2021 | |
| VSP3076 | B.1.1.7 | 4/14/21 | surveillance | 99 | 548 | hCoV-19/USA/PA-VSP3076/2021 | OK246650 |
| VSP3077 | B.1.526 | 4/14/21 | surveillance | 99.5 | 732 | hCoV-19/USA/PA-VSP3077/2021 | OK246651 |
| VSP3078 | B.1.1.7 | 4/14/21 | surveillance | 99 | 694 | hCoV-19/USA/PA-VSP3078/2021 | OK246652 |
| VSP3079 | B.1.1.7 | 4/14/21 | surveillance | 98.9 | 503 | hCoV-19/USA/PA-VSP3079/2021 | OK246653 |
| VSP3080 | B.1.1.7 | 4/14/21 | surveillance | 99.2 | 459 | hCoV-19/USA/PA-VSP3080/2021 | OK246654 |
| VSP3082 | B.1.1.7 | 4/14/21 | surveillance | 98.7 | 616 | hCoV-19/USA/PA-VSP3082/2021 | OK246655 |
| VSP3083 | B.1.1.7 | 4/14/21 | surveillance | 99 | 426 | hCoV-19/USA/PA-VSP3083/2021 | OK246656 |
| VSP3084 | B.1.2 | 4/14/21 | surveillance | 99.7 | 759 | hCoV-19/USA/PA-VSP3084/2021 | OK246657 |
| VSP3085 | B.1.1.7 | 4/14/21 | surveillance | 98.9 | 366 | hCoV-19/USA/PA-VSP3085/2021 | OK246658 |
| VSP3086 | B.1.1.7 | 4/14/21 | surveillance | 99.3 | 375 | hCoV-19/USA/PA-VSP3086/2021 | OK246659 |
| VSP3087 | B.1.526 | 4/14/21 | surveillance | 99.3 | 824 | hCoV-19/USA/PA-VSP3087/2021 | OK246660 |
| VSP3089 | B.1.1.7 | 4/27/21 | surveillance | 99.7 | 3858 | hCoV-19/USA/PA-VSP3089/2021 | OK246661 |

| | | | | | | | |
|---|---|---|---|---|---|---|---|
| VSP3090 | B.1.526 | 4/27/21 | surveillance | 99.7 | 4076 | hCoV-19/USA/PA-VSP3090/2021 | OK246662 |
| VSP3091 | B.1.1.7 | 4/27/21 | surveillance | 99.7 | 2999 | hCoV-19/USA/PA-VSP3091/2021 | OK246663 |
| VSP3092 | B.1.1.7 | 4/27/21 | surveillance | 99.7 | 3158 | hCoV-19/USA/PA-VSP3092/2021 | OK246664 |
| VSP3093 | B.1.1.7 | 4/27/21 | surveillance | 99.7 | 4710 | hCoV-19/USA/PA-VSP3093/2021 | OK246665 |
| VSP3094 | B.1.1.7 | 4/27/21 | surveillance | 99.7 | 4186 | hCoV-19/USA/PA-VSP3094/2021 | OK246666 |
| VSP3095 | B.1.1.7 | 4/27/21 | surveillance | 99.7 | 3400 | hCoV-19/USA/PA-VSP3095/2021 | OK246667 |
| VSP3096 | P.1.2 | 4/27/21 | surveillance | 99.7 | 3877 | hCoV-19/USA/PA-VSP3096/2021 | OK246668 |
| VSP3097 | B.1.1.7 | 4/27/21 | surveillance | 99.7 | 3026 | hCoV-19/USA/PA-VSP3097/2021 | OK246669 |
| VSP3098 | B.1.526 | 4/27/21 | surveillance | 99.7 | 3626 | hCoV-19/USA/PA-VSP3098/2021 | OK246670 |
| VSP3099 | B.1.526 | 4/28/21 | surveillance | 98.8 | 322 | hCoV-19/USA/PA-VSP3099/2021 | OK246671 |
| VSP3100 | B.1.1.7 | 4/28/21 | surveillance | 99.7 | 3810 | hCoV-19/USA/PA-VSP3100/2021 | OK246672 |
| VSP3101 | B.1.1.7 | 4/28/21 | surveillance | 99.7 | 2964 | hCoV-19/USA/PA-VSP3101/2021 | OK246673 |
| VSP3102 | B.1.1.7 | 6/18/21 | surveillance | 99.6 | 762 | hCoV-19/USA/PA-VSP3102/2021 | OK246674 |
| VSP3103 | B.1.1.7 | 6/18/21 | surveillance | 98.9 | 180 | hCoV-19/USA/PA-VSP3103/2021 | OK246675 |
| VSP3104 | B.1.526 | 6/18/21 | surveillance | 99.7 | 626 | hCoV-19/USA/PA-VSP3104/2021 | OK246676 |
| VSP3105 | P.1 | 6/18/21 | surveillance | 99.6 | 358 | hCoV-19/USA/PA-VSP3105/2021 | OK246677 |
| VSP3106 | B.1.1.7 | 6/18/21 | surveillance | 99.4 | 271 | hCoV-19/USA/PA-VSP3106/2021 | OK246678 |
| VSP3107 | P.1 | 6/18/21 | surveillance | 99.6 | 553 | hCoV-19/USA/PA-VSP3107/2021 | OK246679 |
| VSP3114 | B.1.1.434 | 12/29/20 | surveillance | 99.7 | 3324 | hCoV-19/USA/PA-VSP3114/2020 | OK246683 |
| VSP3115 | B.1.1.434 | 1/4/21 | surveillance | 99.7 | 3216 | hCoV-19/USA/PA-VSP3115/2021 | OK246684 |
| VSP3117 | B.1.243 | 1/4/21 | surveillance | 98.7 | 462 | hCoV-19/USA/PA-VSP3117/2021 | OK246685 |
| VSP3118 | B.1.243 | 12/29/20 | surveillance | 95.3 | 3274 | hCoV-19/USA/PA-VSP3118/2020 | |
| VSP3120 | B.1.243 | 1/7/21 | surveillance | 99.8 | 4508 | hCoV-19/USA/PA-VSP3120/2021 | OK246686 |
| VSP3121 | B.1.243 | 12/29/20 | surveillance | 98.8 | 508 | hCoV-19/USA/PA-VSP3121/2020 | |
| VSP3122 | B.1.2 | 1/6/21 | surveillance | 99.7 | 2904 | hCoV-19/USA/PA-VSP3122/2021 | OK246687 |
| VSP3128 | B.1.582 | 12/24/20 | surveillance | 97.8 | 1035 | hCoV-19/USA/PA-VSP3128/2020 | OK246689 |
| VSP3130 | B.1.243 | 1/5/21 | surveillance | 95.7 | 1590 | hCoV-19/USA/PA-VSP3130/2021 | OK246690 |
| VSP3131 | B.1 | 12/30/20 | surveillance | 97.8 | 2341 | hCoV-19/USA/PA-VSP3131/2020 | OK246691 |
| VSP3132 | B.1.409 | 12/24/20 | surveillance | 98 | 1762 | hCoV-19/USA/PA-VSP3132/2020 | OK246692 |
| VSP3135 | B.1.243 | 12/30/20 | surveillance | 97.3 | 834 | hCoV-19/USA/PA-VSP3135/2020 | OK246693 |
| VSP3137 | B.1 | 12/30/20 | surveillance | 99.3 | 822 | hCoV-19/USA/PA-VSP3137/2020 | OK246694 |
| VSP3138 | B.1.2 | 12/30/20 | surveillance | 99.7 | 1684 | hCoV-19/USA/PA-VSP3138/2020 | OK246695 |
| VSP3140 | B.1 | 1/5/21 | surveillance | 97.2 | 1019 | hCoV-19/USA/PA-VSP3140/2021 | OK246696 |
| VSP3141 | B.1.243 | 1/4/21 | surveillance | 99.7 | 2530 | hCoV-19/USA/PA-VSP3141/2021 | OK246697 |
| VSP3142 | B.1 | 1/5/21 | surveillance | 98.8 | 1113 | hCoV-19/USA/PA-VSP3142/2021 | OK246698 |
| VSP3143 | B.1.1 | 12/29/20 | surveillance | 95.2 | 1696 | hCoV-19/USA/PA-VSP3143/2020 | |
| VSP3145 | B.1.1 | 12/24/20 | surveillance | 97.5 | 1028 | hCoV-19/USA/PA-VSP3145/2020 | OK246699 |
| VSP3146 | B.1.243 | 2/3/21 | surveillance | 99.7 | 1655 | hCoV-19/USA/PA-VSP3146/2021 | OK246700 |
| VSP3147 | B.1.596 | 2/3/21 | surveillance | 99.7 | 1451 | hCoV-19/USA/PA-VSP3147/2021 | OK246701 |
| VSP3148 | B.1.243 | 1/3/21 | surveillance | 99.7 | 1292 | hCoV-19/USA/PA-VSP3148/2021 | OK246702 |
| VSP3149 | B.1.596 | 2/3/21 | surveillance | 99.7 | 1116 | hCoV-19/USA/PA-VSP3149/2021 | OK246703 |
| VSP3150 | B.1.596 | 1/20/21 | surveillance | 97.8 | 206 | hCoV-19/USA/PA-VSP3150/2021 | OK246704 |
| VSP3151 | B.1.369 | 5/5/20 | surveillance | 99.4 | 567 | hCoV-19/USA/PA-VSP3151/2020 | OK246705 |
| VSP3152 | B.1.369 | 5/27/20 | surveillance | 99.7 | 287 | hCoV-19/USA/PA-VSP3152/2020 | OK246706 |
| VSP3153 | B.1 | 5/27/20 | surveillance | 99 | 137 | hCoV-19/USA/PA-VSP3153/2020 | OK246707 |
| VSP3154 | B.1 | 5/27/20 | surveillance | 99.7 | 493 | hCoV-19/USA/PA-VSP3154/2020 | OK246708 |
| VSP3155 | B.1.520 | 5/4/20 | surveillance | 99.5 | 374 | hCoV-19/USA/PA-VSP3155/2020 | OK246709 |

| | | | | | | | |
|---|---|---|---|---|---|---|---|
| VSP3156 | B.1.564 | 5/5/20 | surveillance | 99.7 | 295 | hCoV-19/USA/PA-VSP3156/2020 | OK246710 |
| VSP3157 | B.1.520 | 5/4/20 | surveillance | 99.5 | 256 | hCoV-19/USA/PA-VSP3157/2020 | OK246711 |
| VSP3158 | B.1 | 4/23/20 | surveillance | 99.7 | 498 | hCoV-19/USA/PA-VSP3158/2020 | OK246712 |
| VSP3159 | B.1 | 5/5/20 | surveillance | 99.7 | 269 | hCoV-19/USA/PA-VSP3159/2020 | OK246713 |
| VSP3160 | B.1.369 | 4/23/20 | surveillance | 99 | 614 | hCoV-19/USA/PA-VSP3160/2020 | OK246714 |
| VSP3161 | B.1.520 | 4/23/20 | surveillance | 99.1 | 334 | hCoV-19/USA/PA-VSP3161/2020 | OK246715 |
| VSP3162 | B.1.311 | 2/5/21 | surveillance | 99.4 | 659 | hCoV-19/USA/PA-VSP3162/2021 | OK246716 |
| VSP3163 | B.1.361 | 2/5/21 | surveillance | 99.7 | 2011 | hCoV-19/USA/PA-VSP3163/2021 | OK246717 |
| VSP3164 | B.1 | 2/5/21 | surveillance | 99.5 | 441 | hCoV-19/USA/PA-VSP3164/2021 | OK246718 |
| VSP3169 | B.1.311 | 3/16/21 | surveillance | 99.4 | 793 | hCoV-19/USA/PA-VSP3169/2021 | OK246721 |
| VSP3170 | B.1.1.7 | 4/1/21 | surveillance | 97.4 | 833 | hCoV-19/USA/PA-VSP3170/2021 | OK246722 |
| VSP3181 | B.1.1.7 | 3/24/21 | surveillance | 99.3 | 557 | hCoV-19/USA/PA-VSP3181/2021 | OK246732 |
| VSP3182 | B.1.349 | 4/5/21 | surveillance | 98.9 | 151 | hCoV-19/USA/PA-VSP3182/2021 | OK246733 |
| VSP3183 | B.1.2 | 4/6/21 | surveillance | 99.7 | 402 | hCoV-19/USA/PA-VSP3183/2021 | OK246734 |
| VSP3187 | B.1.1.7 | 5/5/21 | surveillance | 99.6 | 794 | hCoV-19/USA/PA-VSP3187/2021 | OK246736 |
| VSP3188 | B.1.1.7 | 5/5/21 | surveillance | 99.2 | 438 | hCoV-19/USA/PA-VSP3188/2021 | OK246737 |
| VSP3189 | B.1.1.7 | 5/5/21 | surveillance | 99.6 | 1060 | hCoV-19/USA/PA-VSP3189/2021 | OK246738 |
| VSP3190 | B.1.1.7 | 5/5/21 | surveillance | 99.7 | 1004 | hCoV-19/USA/PA-VSP3190/2021 | OK246739 |
| VSP3191 | P.1 | 5/5/21 | surveillance | 97.5 | 1058 | hCoV-19/USA/PA-VSP3191/2021 | OK246740 |
| VSP3193 | B.1.1.7 | 5/5/21 | surveillance | 99.7 | 606 | hCoV-19/USA/PA-VSP3193/2021 | OK246741 |
| VSP3194 | B.1.1.7 | 5/5/21 | surveillance | 99.7 | 952 | hCoV-19/USA/PA-VSP3194/2021 | OK246742 |
| VSP3195 | B.1.1.7 | 5/5/21 | surveillance | 99.7 | 1015 | hCoV-19/USA/PA-VSP3195/2021 | OK246743 |
| VSP3196 | B.1.1.7 | 5/5/21 | surveillance | 99.7 | 1208 | hCoV-19/USA/PA-VSP3196/2021 | OK246744 |
| VSP3197 | P.1 | 5/5/21 | surveillance | 99.7 | 941 | hCoV-19/USA/PA-VSP3197/2021 | OK246745 |
| VSP3198 | P.1 | 5/5/21 | surveillance | 99.7 | 1060 | hCoV-19/USA/PA-VSP3198/2021 | OK246746 |
| VSP3199 | B.1.1.7 | 5/5/21 | surveillance | 99.7 | 1153 | hCoV-19/USA/PA-VSP3199/2021 | OK246747 |
| VSP3200 | B.1.1.7 | 5/5/21 | surveillance | 99.7 | 1260 | hCoV-19/USA/PA-VSP3200/2021 | OK246748 |
| VSP3201 | B.1.1.7 | 5/12/21 | surveillance | 99.7 | 1048 | hCoV-19/USA/PA-VSP3201/2021 | OK246749 |
| VSP3202 | B.1.1.7 | 5/12/21 | surveillance | 99.7 | 1177 | hCoV-19/USA/PA-VSP3202/2021 | OK246750 |
| VSP3203 | B.1.1.7 | 5/12/21 | surveillance | 98.5 | 141 | hCoV-19/USA/PA-VSP3203/2021 | OK246751 |
| VSP3204 | B.1.1.7 | 5/12/21 | surveillance | 97 | 233 | hCoV-19/USA/PA-VSP3204/2021 | OK246752 |
| VSP3205 | B.1.1.7 | 5/12/21 | surveillance | 99.5 | 399 | hCoV-19/USA/PA-VSP3205/2021 | OK246753 |
| VSP3206 | B.1.1.7 | 5/12/21 | surveillance | 95.5 | 1174 | hCoV-19/USA/PA-VSP3206/2021 | OK246754 |
| VSP3207 | B.1.1.7 | 5/12/21 | surveillance | 99.4 | 339 | hCoV-19/USA/PA-VSP3207/2021 | OK246755 |
| VSP3208 | B.1.637 | 5/11/21 | surveillance | 98.4 | 932 | hCoV-19/USA/PA-VSP3208/2021 | OK246756 |
| VSP3209 | B.1.1.7 | 5/12/21 | surveillance | 99.7 | 1137 | hCoV-19/USA/PA-VSP3209/2021 | OK246757 |
| VSP3210 | B.1.1.7 | 5/12/21 | surveillance | 99.3 | 603 | hCoV-19/USA/PA-VSP3210/2021 | OK246758 |
| VSP3211 | B.1.526 | 5/12/21 | surveillance | 99.6 | 1120 | hCoV-19/USA/PA-VSP3211/2021 | OK246759 |
| VSP3212 | B.1.1.7 | 5/11/21 | surveillance | 99.6 | 1175 | hCoV-19/USA/PA-VSP3212/2021 | OK246760 |
| VSP3214 | B.1.1.7 | 5/11/21 | surveillance | 99.3 | 1189 | hCoV-19/USA/PA-VSP3214/2021 | OK246761 |
| VSP3215 | B.1.1.7 | 5/11/21 | surveillance | 99.2 | 1270 | hCoV-19/USA/PA-VSP3215/2021 | OK246762 |
| VSP3216 | B.1.1.7 | 5/11/21 | surveillance | 99.7 | 1226 | hCoV-19/USA/PA-VSP3216/2021 | OK246763 |
| VSP3217 | B.1.637 | 5/11/21 | surveillance | 97.8 | 1107 | hCoV-19/USA/PA-VSP3217/2021 | OK246764 |
| VSP3218 | B.1.1.7 | 5/11/21 | surveillance | 99.6 | 1054 | hCoV-19/USA/PA-VSP3218/2021 | OK246765 |
| VSP3219 | B.1.1.7 | 5/11/21 | surveillance | 97.5 | 406 | hCoV-19/USA/PA-VSP3219/2021 | |
| VSP3220 | B.1.1.7 | 5/11/21 | surveillance | 99.7 | 1002 | hCoV-19/USA/PA-VSP3220/2021 | OK246766 |
| VSP3221 | None | 5/19/21 | surveillance | 97.6 | 388 | hCoV-19/USA/PA-VSP3221/2021 | OK246767 |

| | | | | | | | |
|---|---|---|---|---|---|---|---|
| VSP3222 | B.1.617.2 | 5/19/21 | surveillance | 99.4 | 3361 | hCoV-19/USA/PA-VSP3222/2021 | OK246768 |
| VSP3223 | B.1.621 | 5/19/21 | surveillance | 98.8 | 1894 | hCoV-19/USA/PA-VSP3223/2021 | OK246769 |
| VSP3322 | B.1.526 | 4/21/21 | surveillance | 97.8 | 132 | hCoV-19/USA/PA-VSP3322/2021 | OK246770 |
| VSP3323 | B.1.526 | 3/31/21 | surveillance | 99.4 | 4477 | hCoV-19/USA/PA-VSP3323/2021 | OK246771 |
| VSP3325 | AY.25 | 6/26/21 | surveillance | 99.6 | 2621 | hCoV-19/USA/PA-VSP3325/2021 | OK246772 |
| VSP3327 | B.1.1.7 | 6/25/21 | surveillance | 99.3 | 993 | hCoV-19/USA/PA-VSP3327/2021 | OK246773 |
| VSP3328 | P.1 | 6/26/21 | surveillance | 99.4 | 1138 | hCoV-19/USA/PA-VSP3328/2021 | OK246774 |
| VSP3329 | B.1.1.7 | 6/25/21 | surveillance | 99.1 | 721 | hCoV-19/USA/PA-VSP3329/2021 | OK246775 |
| VSP3330 | B.1.1.7 | 6/28/21 | surveillance | 99.3 | 991 | hCoV-19/USA/PA-VSP3330/2021 | OK246776 |
| VSP3331 | AY.25 | 6/29/21 | surveillance | 99.5 | 1215 | hCoV-19/USA/PA-VSP3331/2021 | OK246777 |
| VSP3332 | AY.25 | 6/29/21 | surveillance | 99.5 | 745 | hCoV-19/USA/PA-VSP3332/2021 | OK246778 |
| VSP3336 | AY.25 | 6/30/21 | surveillance | 98 | 1282 | hCoV-19/USA/PA-VSP3336/2021 | OK246779 |
| VSP3337 | AY.25 | 6/30/21 | surveillance | 99.3 | 1039 | hCoV-19/USA/PA-VSP3337/2021 | OK246780 |
| VSP3338 | AY.19 | 6/22/21 | surveillance | 99.5 | 1079 | hCoV-19/USA/PA-VSP3338/2021 | OK246781 |
| VSP3339 | B.1.1.7 | 4/28/21 | surveillance | 99.6 | 5417 | hCoV-19/USA/PA-VSP3339/2021 | OK246782 |
| VSP3340 | B.1.1.7 | 4/28/21 | surveillance | 99.5 | 6714 | hCoV-19/USA/PA-VSP3340/2021 | OK246783 |
| VSP3341 | B.1.427 | 4/28/21 | surveillance | 99.7 | 5224 | hCoV-19/USA/PA-VSP3341/2021 | OK246784 |
| VSP3342 | B.1.1.7 | 4/28/21 | surveillance | 99.4 | 2151 | hCoV-19/USA/PA-VSP3342/2021 | OK246785 |
| VSP3343 | B.1.637 | 4/7/21 | surveillance | 99.6 | 5376 | hCoV-19/USA/PA-VSP3343/2021 | OK246786 |
| VSP3344 | B.1.1.7 | 3/31/21 | surveillance | 99.2 | 1016 | hCoV-19/USA/PA-VSP3344/2021 | OK246787 |
| VSP3345 | B.1.1.7 | 3/31/21 | surveillance | 96.5 | 93 | hCoV-19/USA/PA-VSP3345/2021 | OK246788 |
| VSP3346 | B.1.1.7 | 3/31/21 | surveillance | 99.2 | 674 | hCoV-19/USA/PA-VSP3346/2021 | OK246789 |
| VSP3347 | B.1.1.7 | 3/31/21 | surveillance | 99.3 | 1510 | hCoV-19/USA/PA-VSP3347/2021 | OK246790 |
| VSP3351 | B.1.1.7 | 7/4/21 | surveillance | 97.8 | 2030 | hCoV-19/USA/PA-VSP3351/2021 | OK246791 |
| VSP3353 | AY.14 | 7/14/21 | surveillance | 97.1 | 222 | hCoV-19/USA/PA-VSP3353/2021 | OK246792 |
| VSP3358 | AY.14 | 7/14/21 | surveillance | 95.7 | 273 | hCoV-19/USA/PA-VSP3358/2021 | OK246793 |
| VSP3359 | AY.12 | 7/3/21 | surveillance | 97.7 | 1362 | hCoV-19/USA/PA-VSP3359/2021 | OK246794 |
| VSP3360 | B.1.630 | 7/11/21 | surveillance | 96.7 | 1319 | hCoV-19/USA/PA-VSP3360/2021 | OK246795 |
| VSP3361 | P.1 | 7/12/21 | surveillance | 98.1 | 1741 | hCoV-19/USA/PA-VSP3361/2021 | OK246796 |
| VSP3363 | AY.3 | 7/8/21 | surveillance | 95.9 | 2098 | hCoV-19/USA/PA-VSP3363/2021 | OK246797 |
| VSP3367 | AY.20 | 7/12/21 | surveillance | 99 | 803 | hCoV-19/USA/PA-VSP3367/2021 | OK246798 |
| VSP3369 | B.1.1.7 | 3/7/21 | accine breakthroug | 97.9 | 232 | hCoV-19/USA/PA-VSP3369/2021 | OK246799 |
| VSP3370 | B.1.1.7 | 3/3/21 | accine breakthroug | 96.6 | 204 | hCoV-19/USA/PA-VSP3370/2021 | OK246800 |
| VSP3371 | B.1.1.7 | 3/4/21 | accine breakthroug | 99.3 | 460 | hCoV-19/USA/PA-VSP3371/2021 | OK246801 |
| VSP3372 | B.1.588 | 3/2/21 | accine breakthroug | 99.2 | 234 | hCoV-19/USA/PA-VSP3372/2021 | OK246802 |
| VSP3373 | B.1.588 | 3/1/21 | accine breakthroug | 98.7 | 162 | hCoV-19/USA/PA-VSP3373/2021 | OK246803 |
| VSP3374 | B.1.1.7 | 3/3/21 | accine breakthroug | 98.5 | 695 | hCoV-19/USA/PA-VSP3374/2021 | OK246804 |
| VSP3377 | B.1.2 | 3/10/21 | accine breakthroug | 99.7 | 930 | hCoV-19/USA/PA-VSP3377/2021 | OK246805 |
| VSP3378 | B.1.1.7 | 3/18/21 | accine breakthroug | 96.6 | 164 | hCoV-19/USA/PA-VSP3378/2021 | OK246806 |
| VSP3380 | B.1.1.7 | 3/16/21 | accine breakthroug | 99.1 | 431 | hCoV-19/USA/PA-VSP3380/2021 | OK246807 |
| VSP3381 | B.1.526 | 3/23/21 | accine breakthroug | 98.8 | 636 | hCoV-19/USA/PA-VSP3381/2021 | OK246808 |
| VSP3382 | B.1.1.7 | 3/24/21 | accine breakthroug | 99.5 | 2567 | hCoV-19/USA/PA-VSP3382/2021 | OK246809 |
| VSP3383 | B.1.240 | 3/16/21 | accine breakthroug | 95.7 | 104 | hCoV-19/USA/PA-VSP3383/2021 | OK246810 |
| VSP3384 | B.1.243 | 3/24/21 | accine breakthroug | 98.9 | 688 | hCoV-19/USA/PA-VSP3384/2021 | OK246811 |
| VSP3385 | None | 3/30/21 | accine breakthroug | 98 | 770 | hCoV-19/USA/PA-VSP3385/2021 | OK246812 |
| VSP3387 | B.1.1.7 | 3/28/21 | accine breakthroug | 97.9 | 1951 | hCoV-19/USA/PA-VSP3387/2021 | OK246813 |
| VSP3388 | B.1.1.7 | 4/1/21 | accine breakthroug | 96.4 | 1021 | hCoV-19/USA/PA-VSP3388/2021 | OK246814 |

| | | | | | | | |
|---|---|---|---|---|---|---|---|
| VSP3389 | P.1 | 3/25/21 | accine breakthroug | 99.2 | 445 | hCoV-19/USA/PA-VSP3389/2021 | OK246815 |
| VSP3390 | B.1 | 3/29/21 | accine breakthroug | 98.7 | 149 | hCoV-19/USA/PA-VSP3390/2021 | |
| VSP3391 | B.1.526 | 4/7/21 | accine breakthroug | 99 | 726 | hCoV-19/USA/PA-VSP3391/2021 | OK246816 |
| VSP3392 | P.1 | 4/2/21 | accine breakthroug | 98.2 | 103 | hCoV-19/USA/PA-VSP3392/2021 | OK246817 |
| VSP3395 | B.1.1.7 | 4/4/21 | accine breakthroug | 99.2 | 1026 | hCoV-19/USA/PA-VSP3395/2021 | OK246818 |
| VSP3396 | B.1.1.7 | 4/9/21 | accine breakthroug | 98 | 972 | hCoV-19/USA/PA-VSP3396/2021 | |
| VSP3397 | B.1.1.7 | 4/13/21 | accine breakthroug | 97.3 | 887 | hCoV-19/USA/PA-VSP3397/2021 | OK246819 |
| VSP3398 | B.1.1.7 | 4/1/21 | accine breakthroug | 95.9 | 79 | hCoV-19/USA/PA-VSP3398/2021 | OK246820 |
| VSP3399 | B.1.526 | 4/13/21 | accine breakthroug | 99.4 | 736 | hCoV-19/USA/PA-VSP3399/2021 | OK246821 |
| VSP3400 | B.1.1.7 | 4/14/21 | accine breakthroug | 99.2 | 893 | hCoV-19/USA/PA-VSP3400/2021 | OK246822 |
| VSP3402 | B.1.1.7 | 4/9/21 | accine breakthroug | 98.9 | 1248 | hCoV-19/USA/PA-VSP3402/2021 | OK246823 |
| VSP3403 | B.1.1.7 | 4/15/21 | accine breakthroug | 99.2 | 1053 | hCoV-19/USA/PA-VSP3403/2021 | OK246824 |
| VSP3404 | B.1.1.7 | 4/17/21 | accine breakthroug | 98.9 | 1238 | hCoV-19/USA/PA-VSP3404/2021 | OK246825 |
| VSP3405 | P.1.2 | 4/18/21 | accine breakthroug | 99.1 | 966 | hCoV-19/USA/PA-VSP3405/2021 | OK246826 |
| VSP3406 | B.1.617.2 | 4/21/21 | accine breakthroug | 98.8 | 1351 | hCoV-19/USA/PA-VSP3406/2021 | OK246827 |
| VSP3408 | P.1 | 4/21/21 | accine breakthroug | 99.4 | 280 | hCoV-19/USA/PA-VSP3408/2021 | OK246828 |
| VSP3409 | B.1.1.7 | 4/22/21 | accine breakthroug | 99.7 | 3061 | hCoV-19/USA/PA-VSP3409/2021 | OK246829 |
| VSP3410 | B.1.617.1 | 4/28/21 | accine breakthroug | 98 | 3684 | hCoV-19/USA/PA-VSP3410/2021 | OK246830 |
| VSP3411 | B.1.1.7 | 4/26/21 | accine breakthroug | 99 | 3515 | hCoV-19/USA/PA-VSP3411/2021 | OK246831 |
| VSP3412 | B.1.1.7 | 4/26/21 | accine breakthroug | 99.7 | 3459 | hCoV-19/USA/PA-VSP3412/2021 | OK246832 |
| VSP3413 | B.1.1.7 | 4/22/21 | accine breakthroug | 99.4 | 4785 | hCoV-19/USA/PA-VSP3413/2021 | OK246833 |
| VSP3414 | B.1.1.7 | 4/23/21 | accine breakthroug | 98.8 | 3828 | hCoV-19/USA/PA-VSP3414/2021 | OK246834 |
| VSP3415 | B.1.1.7 | 4/26/21 | accine breakthroug | 99.7 | 4035 | hCoV-19/USA/PA-VSP3415/2021 | OK246835 |
| VSP3416 | B.1.1.7 | 4/13/21 | accine breakthroug | 99.7 | 4052 | hCoV-19/USA/PA-VSP3416/2021 | OK246836 |
| VSP3420 | B.1.1.7 | 4/19/21 | accine breakthroug | 97.8 | 60 | hCoV-19/USA/PA-VSP3420/2021 | OK246837 |
| VSP3421 | P.1.2 | 5/5/21 | accine breakthroug | 98.8 | 1102 | hCoV-19/USA/PA-VSP3421/2021 | OK246838 |
| VSP3423 | B.1.1.7 | 5/15/21 | accine breakthroug | 99.1 | 467 | hCoV-19/USA/PA-VSP3423/2021 | OK246839 |
| VSP3424 | B.1.621 | 4/30/21 | accine breakthroug | 99.7 | 885 | hCoV-19/USA/PA-VSP3424/2021 | OK246840 |
| VSP3425 | B.1.1.7 | 5/11/21 | accine breakthroug | 99.2 | 250 | hCoV-19/USA/PA-VSP3425/2021 | OK246841 |
| VSP3426 | B.1.1.7 | 5/11/21 | accine breakthroug | 99 | 700 | hCoV-19/USA/PA-VSP3426/2021 | OK246842 |
| VSP3427 | B.1.1.7 | 5/11/21 | accine breakthroug | 99.4 | 1854 | hCoV-19/USA/PA-VSP3427/2021 | OK246843 |
| VSP3428 | B.1.1.7 | 5/20/21 | accine breakthroug | 99.4 | 1527 | hCoV-19/USA/PA-VSP3428/2021 | OK246844 |
| VSP3429 | B.1.1.7 | 5/12/21 | accine breakthroug | 99.7 | 1980 | hCoV-19/USA/PA-VSP3429/2021 | OK246845 |
| VSP3430 | B.1.637 | 6/4/21 | accine breakthroug | 99.6 | 1390 | hCoV-19/USA/PA-VSP3430/2021 | OK246846 |
| VSP5006 | B.1.1.7 | 5/18/21 | accine breakthroug | 99.6 | 566 | hCoV-19/USA/PA-VSP5006/2021 | OK246851 |
| VSP5007 | B.1.2 | 5/21/21 | surveillance | 99.7 | 605 | hCoV-19/USA/PA-VSP5007/2021 | OK246852 |
| VSP5008 | B.1.1.7 | 5/23/21 | surveillance | 98.1 | 83 | hCoV-19/USA/PA-VSP5008/2021 | OK246853 |
| VSP5010 | B.1.621 | 5/24/21 | surveillance | 99.7 | 2894 | hCoV-19/USA/PA-VSP5010/2021 | OK246854 |
| VSP5011 | B.1.621 | 5/24/21 | surveillance | 99.7 | 6351 | hCoV-19/USA/PA-VSP5011/2021 | OK246855 |
| VSP5012 | B.1.1.7 | 5/24/21 | surveillance | 99 | 3205 | hCoV-19/USA/NJ-VSP5012/2021 | OK246856 |
| VSP5013 | R.1 | 5/24/21 | surveillance | 98.7 | 447 | hCoV-19/USA/PA-VSP5013/2021 | OK246857 |
| VSP5014 | B.1.1.7 | 5/25/21 | surveillance | 99.7 | 1941 | hCoV-19/USA/PA-VSP5014/2021 | OK246858 |
| VSP5015 | B.1.621 | 5/25/21 | surveillance | 99.7 | 5540 | hCoV-19/USA/PA-VSP5015/2021 | OK246859 |
| VSP5016 | B.1.1.7 | 5/25/21 | surveillance | 99.7 | 4460 | hCoV-19/USA/PA-VSP5016/2021 | OK246860 |
| VSP5018 | P.1.2 | 5/25/21 | surveillance | 99.7 | 2795 | hCoV-19/USA/PA-VSP5018/2021 | OK246861 |
| VSP5019 | P.1 | 5/25/21 | surveillance | 99.7 | 1538 | hCoV-19/USA/PA-VSP5019/2021 | OK246862 |
| VSP5020 | B.1.1.7 | 5/25/21 | surveillance | 99.4 | 956 | hCoV-19/USA/PA-VSP5020/2021 | OK246863 |

| | | | | | | | |
|---|---|---|---|---|---|---|---|
| VSP5021 | B.1.1.7 | 5/25/21 | surveillance | 99.7 | 2462 | hCoV-19/USA/PA-VSP5021/2021 | OK246864 |
| VSP5022 | B.1.1.7 | 5/25/21 | surveillance | 99.7 | 787 | hCoV-19/USA/PA-VSP5022/2021 | OK246865 |
| VSP5023 | B.1.1.7 | 5/26/21 | surveillance | 99.7 | 10548 | hCoV-19/USA/PA-VSP5023/2021 | OK246866 |
| VSP5024 | B.1.1.7 | 5/26/21 | surveillance | 99.7 | 5345 | hCoV-19/USA/PA-VSP5024/2021 | OK246867 |
| VSP5026 | B.1.526 | 5/26/21 | surveillance | 99.7 | 8728 | hCoV-19/USA/PA-VSP5026/2021 | OK246868 |
| VSP5028 | B.1.1.7 | 5/26/21 | surveillance | 99.7 | 5350 | hCoV-19/USA/PA-VSP5028/2021 | OK246869 |
| VSP5029 | P.1.10 | 5/26/21 | surveillance | 99.7 | 13664 | hCoV-19/USA/NJ-VSP5029/2021 | OK246870 |
| VSP5030 | B.1.1.7 | 5/27/21 | surveillance | 99.4 | 642 | hCoV-19/USA/PA-VSP5030/2021 | OK246871 |
| VSP5032 | B.1.1.7 | 5/28/21 | surveillance | 99.7 | 1234 | hCoV-19/USA/PA-VSP5032/2021 | OK246872 |
| VSP5033 | B.1.1.7 | 5/28/21 | surveillance | 99.7 | 2543 | hCoV-19/USA/PA-VSP5033/2021 | OK246873 |
| VSP5034 | B.1.1.7 | 5/28/21 | surveillance | 99.1 | 3893 | hCoV-19/USA/PA-VSP5034/2021 | OK246874 |
| VSP5035 | B.1.1.7 | 5/28/21 | surveillance | 99.7 | 436 | hCoV-19/USA/PA-VSP5035/2021 | OK246875 |
| VSP5036 | B.1.621 | 5/29/21 | surveillance | 98.5 | 2240 | hCoV-19/USA/PA-VSP5036/2021 | OK246876 |
| VSP5037 | B.1.1.7 | 5/29/21 | surveillance | 99.7 | 5155 | hCoV-19/USA/PA-VSP5037/2021 | OK246877 |
| VSP5038 | B.1.1.7 | 5/30/21 | surveillance | 99.7 | 2273 | hCoV-19/USA/PA-VSP5038/2021 | OK246878 |
| VSP5039 | B.1.526 | 6/1/21 | surveillance | 99.4 | 954 | hCoV-19/USA/PA-VSP5039/2021 | OK246879 |
| VSP5040 | B.1.1.7 | 6/2/21 | surveillance | 99.7 | 2516 | hCoV-19/USA/PA-VSP5040/2021 | OK246880 |
| VSP5041 | B.1.526 | 5/11/21 | surveillance | 99.7 | 5152 | hCoV-19/USA/PA-VSP5041/2021 | OK246881 |
| VSP5042 | B.1.1.7 | 5/11/21 | surveillance | 99.7 | 4252 | hCoV-19/USA/PA-VSP5042/2021 | OK246882 |
| VSP5043 | B.1.1.7 | 5/15/21 | surveillance | 99.1 | 379 | hCoV-19/USA/PA-VSP5043/2021 | OK246883 |
| VSP5044 | B.1.1.7 | 5/15/21 | surveillance | 95.8 | 1691 | hCoV-19/USA/PA-VSP5044/2021 | |
| VSP5045 | B.1.526 | 5/16/21 | surveillance | 99.1 | 4430 | hCoV-19/USA/PA-VSP5045/2021 | OK246884 |
| VSP5046 | B.1.1.7 | 5/17/21 | surveillance | 99.7 | 1642 | hCoV-19/USA/PA-VSP5046/2021 | OK246885 |
| VSP5047 | B.1.1.7 | 5/17/21 | surveillance | 98.1 | 167 | hCoV-19/USA/PA-VSP5047/2021 | OK246886 |
| VSP5048 | B.1.526 | 5/17/21 | surveillance | 99.7 | 2539 | hCoV-19/USA/PA-VSP5048/2021 | OK246887 |
| VSP5049 | B.1.1.7 | 5/20/21 | surveillance | 99.7 | 6131 | hCoV-19/USA/PA-VSP5049/2021 | OK246888 |
| VSP5050 | B.1.1.7 | 5/20/21 | surveillance | 99.7 | 3633 | hCoV-19/USA/PA-VSP5050/2021 | OK246889 |
| VSP5051 | B.1.1.7 | 5/21/21 | surveillance | 99.1 | 1869 | hCoV-19/USA/PA-VSP5051/2021 | OK246890 |
| VSP5052 | B.1.637 | 5/21/21 | surveillance | 99.5 | 3315 | hCoV-19/USA/PA-VSP5052/2021 | OK246891 |
| VSP5053 | B.1.526 | 5/21/21 | surveillance | 99.7 | 20211 | hCoV-19/USA/PA-VSP5053/2021 | OK246892 |
| VSP5054 | B.1.1.7 | 5/21/21 | surveillance | 99.7 | 4107 | hCoV-19/USA/PA-VSP5054/2021 | OK246893 |
| VSP5055 | B.1.1.7 | 5/21/21 | surveillance | 99.7 | 3478 | hCoV-19/USA/PA-VSP5055/2021 | OK246894 |
| VSP5056 | B.1.1.7 | 5/22/21 | surveillance | 99 | 8259 | hCoV-19/USA/PA-VSP5056/2021 | OK246895 |
| VSP5059 | B.1.1.7 | 5/22/21 | surveillance | 99.7 | 4864 | hCoV-19/USA/PA-VSP5059/2021 | OK246896 |
| VSP5060 | B.1.1.7 | 5/23/21 | surveillance | 99.7 | 2443 | hCoV-19/USA/PA-VSP5060/2021 | OK246897 |
| VSP5061 | B.1.1.7 | 5/23/21 | surveillance | 99.7 | 726 | hCoV-19/USA/PA-VSP5061/2021 | OK246898 |
| VSP5062 | B.1.526 | 5/24/21 | surveillance | 99.7 | 2697 | hCoV-19/USA/PA-VSP5062/2021 | OK246899 |
| VSP5063 | B.1.1.7 | 5/24/21 | surveillance | 97.7 | 84 | hCoV-19/USA/PA-VSP5063/2021 | OK246900 |
| VSP5065 | P.1 | 5/25/21 | surveillance | 99.7 | 7422 | hCoV-19/USA/PA-VSP5065/2021 | OK246901 |
| VSP5066 | B.1.526 | 5/29/21 | surveillance | 99.7 | 4722 | hCoV-19/USA/PA-VSP5066/2021 | OK246902 |
| VSP5067 | B.1.526 | 5/29/21 | surveillance | 99.7 | 5798 | hCoV-19/USA/PA-VSP5067/2021 | OK246903 |
| VSP5068 | B.1.1.7 | 5/29/21 | surveillance | 99.7 | 2579 | hCoV-19/USA/PA-VSP5068/2021 | OK246904 |
| VSP5069 | B.1.526 | 5/30/21 | surveillance | 99.7 | 8162 | hCoV-19/USA/PA-VSP5069/2021 | OK246905 |
| VSP5070 | B.1.1.7 | 5/31/21 | surveillance | 99.7 | 10298 | hCoV-19/USA/PA-VSP5070/2021 | OK246906 |
| VSP5071 | B.1.526 | 5/21/21 | accine breakthroug | 99.1 | 1701 | hCoV-19/USA/PA-VSP5071/2021 | OK246907 |
| VSP5079 | B.1.1.7 | 6/3/21 | surveillance | 99.7 | 6974 | hCoV-19/USA/PA-VSP5079/2021 | OK246909 |
| VSP5080 | B.1.621 | 6/4/21 | surveillance | 99.7 | 2197 | hCoV-19/USA/PA-VSP5080/2021 | OK246910 |

| | | | | | | | |
|---|---|---|---|---|---|---|---|
| VSP5081 | B.1.526 | 6/4/21 | surveillance | 99.7 | 3892 | hCoV-19/USA/PA-VSP5081/2021 | OK246911 |
| VSP5082 | B.1.1.7 | 6/5/21 | surveillance | 99.7 | 2785 | hCoV-19/USA/PA-VSP5082/2021 | OK246912 |
| VSP5083 | P.1 | 6/7/21 | surveillance | 99.7 | 3601 | hCoV-19/USA/PA-VSP5083/2021 | OK246913 |
| VSP5084 | P.1 | 6/7/21 | surveillance | 99.7 | 4861 | hCoV-19/USA/PA-VSP5084/2021 | OK246914 |
| VSP5085 | R.1 | 6/8/21 | surveillance | 99.6 | 4179 | hCoV-19/USA/PA-VSP5085/2021 | OK246915 |
| VSP5086 | AY.4 | 6/8/21 | accine breakthroug | 99.7 | 1229 | hCoV-19/USA/NJ-VSP5086/2021 | OK246916 |
| VSP5088 | B.1.1.7 | 6/9/21 | surveillance | 99.7 | 1613 | hCoV-19/USA/NJ-VSP5088/2021 | OK246917 |
| VSP5089 | B.1.1.7 | 6/10/21 | surveillance | 99.7 | 997 | hCoV-19/USA/NJ-VSP5089/2021 | OK246918 |
| VSP5090 | B.1.1.7 | 5/26/21 | surveillance | 99.5 | 595 | hCoV-19/USA/PA-VSP5090/2021 | OK246919 |
| VSP5091 | B.1.526 | 5/27/21 | surveillance | 99.6 | 840 | hCoV-19/USA/PA-VSP5091/2021 | OK246920 |
| VSP5092 | B.1.1.7 | 5/27/21 | surveillance | 99.7 | 1007 | hCoV-19/USA/PA-VSP5092/2021 | OK246921 |
| VSP5093 | B.1.1.7 | 5/28/21 | surveillance | 99.7 | 884 | hCoV-19/USA/PA-VSP5093/2021 | OK246922 |
| VSP5094 | B.1.1.7 | 5/28/21 | surveillance | 99.6 | 321 | hCoV-19/USA/PA-VSP5094/2021 | OK246923 |
| VSP5095 | B.1.1.7 | 6/2/21 | surveillance | 99.4 | 679 | hCoV-19/USA/PA-VSP5095/2021 | OK246924 |
| VSP5096 | B.1.1.7 | 6/2/21 | surveillance | 99.4 | 602 | hCoV-19/USA/PA-VSP5096/2021 | OK246925 |
| VSP5097 | B.1.1.7 | 6/2/21 | surveillance | 99.2 | 466 | hCoV-19/USA/PA-VSP5097/2021 | OK246926 |
| VSP5098 | P.1.2 | 5/31/21 | surveillance | 99.7 | 2141 | hCoV-19/USA/PA-VSP5098/2021 | OK246927 |
| VSP5099 | B.1.526 | 5/29/21 | surveillance | 99.7 | 4528 | hCoV-19/USA/PA-VSP5099/2021 | OK246928 |
| VSP5100 | B.1.617.2 | 5/30/21 | surveillance | 99.5 | 1053 | hCoV-19/USA/PA-VSP5100/2021 | OK246929 |
| VSP5101 | B.1.1.7 | 5/3/21 | surveillance | 99.7 | 2144 | hCoV-19/USA/PA-VSP5101/2021 | OK246930 |
| VSP5102 | B.1.1.7 | 6/4/21 | surveillance | 99.6 | 1151 | hCoV-19/USA/PA-VSP5102/2021 | OK246931 |
| VSP5103 | B.1.1.7 | 6/7/21 | surveillance | 99.6 | 1876 | hCoV-19/USA/PA-VSP5103/2021 | OK246932 |
| VSP5104 | B.1.1.7 | 6/8/21 | surveillance | 98.8 | 179 | hCoV-19/USA/PA-VSP5104/2021 | OK246933 |
| VSP5105 | B.1.1.7 | 6/9/21 | surveillance | 99.7 | 1812 | hCoV-19/USA/PA-VSP5105/2021 | OK246934 |
| VSP5107 | B.1.1.7 | 6/9/21 | surveillance | 99.7 | 793 | hCoV-19/USA/PA-VSP5107/2021 | OK246935 |
| VSP5110 | B.1.1.7 | 6/10/21 | surveillance | 99 | 326 | hCoV-19/USA/PA-VSP5110/2021 | OK246936 |
| VSP5112 | B.1.1.7 | 6/12/21 | accine breakthroug | 99.1 | 222 | hCoV-19/USA/PA-VSP5112/2021 | OK246937 |
| VSP5113 | B.1.1.7 | 6/13/21 | surveillance | 99.6 | 3050 | hCoV-19/USA/PA-VSP5113/2021 | OK246938 |
| VSP5114 | B.1.617.2 | 6/14/21 | surveillance | 99 | 1387 | hCoV-19/USA/NJ-VSP5114/2021 | OK246939 |
| VSP5115 | None | 6/14/21 | surveillance | 86.6 | 423 | hCoV-19/USA/PA-VSP5115/2021 | OK246940 |
| VSP5119 | B.1.1.7 | 6/16/21 | surveillance | 99.7 | 4195 | hCoV-19/USA/PA-VSP5119/2021 | OK246942 |
| VSP5120 | B.1.621 | 6/17/21 | surveillance | 99.7 | 4073 | hCoV-19/USA/PA-VSP5120/2021 | OK246943 |
| VSP5121 | B.1.617.2 | 6/17/21 | accine breakthroug | 99.7 | 2898 | hCoV-19/USA/PA-VSP5121/2021 | OK246944 |
| VSP5122 | B.1.617.2 | 5/24/21 | surveillance | 99.3 | 1391 | hCoV-19/USA/PA-VSP5122/2021 | OK246945 |
| VSP5123 | B.1.1.7 | 6/1/21 | surveillance | 99.7 | 3452 | hCoV-19/USA/PA-VSP5123/2021 | OK246946 |
| VSP5124 | B.1.1.7 | 6/3/21 | surveillance | 99.7 | 2681 | hCoV-19/USA/PA-VSP5124/2021 | OK246947 |
| VSP5125 | P.1 | 6/5/21 | surveillance | 99.7 | 3875 | hCoV-19/USA/PA-VSP5125/2021 | OK246948 |
| VSP5126 | B.1.1.7 | 6/6/21 | surveillance | 99.6 | 2323 | hCoV-19/USA/PA-VSP5126/2021 | OK246949 |
| VSP5127 | P.1.2 | 6/7/21 | surveillance | 99.7 | 937 | hCoV-19/USA/PA-VSP5127/2021 | OK246950 |
| VSP5128 | B.1.1.7 | 6/8/21 | surveillance | 99.4 | 2257 | hCoV-19/USA/PA-VSP5128/2021 | OK246951 |
| VSP5129 | B.1.1.7 | 6/9/21 | surveillance | 99 | 880 | hCoV-19/USA/PA-VSP5129/2021 | OK246952 |
| VSP5130 | B.1.526 | 6/12/21 | surveillance | 99.6 | 593 | hCoV-19/USA/PA-VSP5130/2021 | OK246953 |
| VSP5131 | B.1.1.7 | 6/12/21 | surveillance | 99.7 | 2837 | hCoV-19/USA/PA-VSP5131/2021 | OK246954 |
| VSP5133 | B.1.1.7 | 6/15/21 | surveillance | 99.7 | 4222 | hCoV-19/USA/PA-VSP5133/2021 | OK246956 |
| VSP5134 | B.1.617.2 | 6/16/21 | surveillance | 99.7 | 4122 | hCoV-19/USA/PA-VSP5134/2021 | OK246957 |
| VSP5135 | AY.25 | 6/17/21 | surveillance | 99.7 | 3905 | hCoV-19/USA/PA-VSP5135/2021 | OK246958 |
| VSP5136 | B.1.637 | 6/18/21 | surveillance | 99.7 | 554 | hCoV-19/USA/PA-VSP5136/2021 | |

| | | | | | | | |
|---|---|---|---|---|---|---|---|
| VSP5137 | P.1 | 6/19/21 | surveillance | 99.7 | 3066 | hCoV-19/USA/PA-VSP5137/2021 | OK246959 |
| VSP5138 | B.1.1.7 | 6/21/21 | surveillance | 99.7 | 4484 | hCoV-19/USA/PA-VSP5138/2021 | OK246960 |
| VSP5139 | R.1 | 6/23/21 | surveillance | 99.4 | 428 | hCoV-19/USA/PA-VSP5139/2021 | OK246961 |
| VSP5145 | B.1.526 | 6/14/21 | surveillance | 99.7 | 3028 | hCoV-19/USA/NC-VSP5145/2021 | OK246963 |
| VSP5146 | AY.25 | 6/19/21 | surveillance | 99.7 | 4159 | hCoV-19/USA/PA-VSP5146/2021 | OK246964 |
| VSP5147 | B.1.621 | 6/21/21 | surveillance | 98 | 141 | hCoV-19/USA/NJ-VSP5147/2021 | OK246965 |
| VSP5148 | B.1.617.2 | 6/22/21 | surveillance | 97 | 355 | hCoV-19/USA/PA-VSP5148/2021 | OK246966 |
| VSP5151 | B.1.1.7 | 6/24/21 | surveillance | 99.7 | 757 | hCoV-19/USA/NJ-VSP5151/2021 | OK246967 |
| VSP5154 | B.1.1.7 | 6/26/21 | surveillance | 99.7 | 1170 | hCoV-19/USA/PA-VSP5154/2021 | OK246968 |
| VSP5155 | B.1.1.7 | 6/26/21 | accine breakthroug | 99.7 | 213 | hCoV-19/USA/PA-VSP5155/2021 | OK246969 |
| VSP5156 | B.1.1.7 | 6/26/21 | surveillance | 99.3 | 209 | hCoV-19/USA/PA-VSP5156/2021 | OK246970 |
| VSP5158 | B.1.1.7 | 6/15/21 | surveillance | 99.7 | 7334 | hCoV-19/USA/PA-VSP5158/2021 | OK246972 |
| VSP5159 | B.1.1.7 | 6/18/21 | surveillance | 99.1 | 2301 | hCoV-19/USA/PA-VSP5159/2021 | OK246973 |
| VSP5160 | B.1.1.7 | 6/19/21 | surveillance | 99.7 | 6611 | hCoV-19/USA/PA-VSP5160/2021 | OK246974 |
| VSP5161 | B.1.617.2 | 6/25/21 | surveillance | 99.7 | 10083 | hCoV-19/USA/PA-VSP5161/2021 | OK246975 |
| VSP5162 | B.1.617.2 | 6/26/21 | surveillance | 99.6 | 5017 | hCoV-19/USA/PA-VSP5162/2021 | OK246976 |
| VSP5164 | None | 6/28/21 | surveillance | 95.9 | 4890 | hCoV-19/USA/PA-VSP5164/2021 | OK246977 |
| VSP5165 | B.1.617.2 | 6/24/21 | surveillance | 99.1 | 399 | hCoV-19/USA/PA-VSP5165/2021 | OK246978 |
| VSP5166 | B.1.617.2 | 6/19/21 | surveillance | 99.3 | 1241 | hCoV-19/USA/PA-VSP5166/2021 | OK246979 |
| VSP5167 | B.1.1.7 | 6/13/21 | surveillance | 99.7 | 2571 | hCoV-19/USA/PA-VSP5167/2021 | OK246980 |
| VSP5169 | AY.12 | 6/16/21 | surveillance | 99.6 | 618 | hCoV-19/USA/PA-VSP5169/2021 | OK246981 |
| VSP5170 | AY.3 | 6/21/21 | surveillance | 99.1 | 2147 | hCoV-19/USA/NJ-VSP5170/2021 | OK246982 |
| VSP5173 | AY.25 | 6/26/21 | surveillance | 99.7 | 3157 | hCoV-19/USA/PA-VSP5173/2021 | OK246983 |
| VSP5174 | AY.25 | 6/26/21 | surveillance | 99.3 | 2359 | hCoV-19/USA/PA-VSP5174/2021 | OK246984 |
| VSP5175 | B.1.617.2 | 6/29/21 | surveillance | 99 | 1355 | hCoV-19/USA/PA-VSP5175/2021 | OK246985 |
| VSP5176 | B.1.1.7 | 6/30/21 | surveillance | 99.7 | 2979 | hCoV-19/USA/PA-VSP5176/2021 | OK246986 |
| VSP5178 | B.1.1.7 | 7/2/21 | surveillance | 99.7 | 1021 | hCoV-19/USA/PA-VSP5178/2021 | OK246987 |
| VSP5179 | B.1.617.2 | 7/2/21 | accine breakthroug | 98.9 | 1737 | hCoV-19/USA/PA-VSP5179/2021 | OK246988 |
| VSP5183 | B.1.617.2 | 7/4/21 | surveillance | 99.4 | 2243 | hCoV-19/USA/TX-VSP5183/2021 | OK246989 |
| VSP5184 | B.1.1.7 | 7/5/21 | surveillance | 99.7 | 596 | hCoV-19/USA/PA-VSP5184/2021 | OK246990 |
| VSP5185 | AY.14 | 7/6/21 | surveillance | 96.7 | 1272 | hCoV-19/USA/NJ-VSP5185/2021 | OK246991 |
| VSP5186 | AY.25 | 7/6/21 | surveillance | 98.3 | 579 | hCoV-19/USA/PA-VSP5186/2021 | OK246992 |
| VSP5187 | B.1.617.2 | 7/7/21 | surveillance | 96.2 | 477 | hCoV-19/USA/CO-VSP5187/2021 | OK246993 |
| VSP5188 | B.1.617.2 | 7/7/21 | surveillance | 99.2 | 7101 | hCoV-19/USA/KS-VSP5188/2021 | OK246994 |
| VSP5192 | B.1.617.2 | 7/9/21 | surveillance | 99.7 | 9123 | hCoV-19/USA/PA-VSP5192/2021 | OK246996 |
| VSP5193 | AY.25 | 6/25/21 | surveillance | 99.7 | 6559 | hCoV-19/USA/PA-VSP5193/2021 | OK246997 |
| VSP5194 | B.1.1.7 | 6/26/21 | surveillance | 99.6 | 5669 | hCoV-19/USA/PA-VSP5194/2021 | OK246998 |
| VSP5198 | AY.25 | 7/9/21 | surveillance | 99.1 | 7272 | hCoV-19/USA/PA-VSP5198/2021 | OK246999 |
| VSP5200 | B.1.617.2 | 7/10/21 | surveillance | 97.8 | 4248 | hCoV-19/USA/OH-VSP5200/2021 | OK247000 |
| VSP5201 | None | 7/10/21 | surveillance | 95.2 | 2310 | hCoV-19/USA/PA-VSP5201/2021 | OK247001 |
| VSP5202 | AY.12 | 7/10/21 | surveillance | 96.1 | 1322 | hCoV-19/USA/PA-VSP5202/2021 | OK247002 |
| VSP5206 | B.1.617.2 | 7/10/21 | surveillance | 99.7 | 7970 | hCoV-19/USA/PA-VSP5206/2021 | OK247003 |
| VSP5207 | B.1.621 | 7/11/21 | surveillance | 99.7 | 3729 | hCoV-19/USA/NJ-VSP5207/2021 | OK247004 |
| VSP5209 | B.1.617.2 | 7/11/21 | surveillance | 97.8 | 4618 | hCoV-19/USA/PA-VSP5209/2021 | OK247005 |
| VSP5214 | B.1.617.2 | 7/13/21 | surveillance | 99.7 | 7528 | hCoV-19/USA/PA-VSP5214/2021 | OK247006 |
| VSP5217 | AY.24 | 7/13/21 | surveillance | 99.7 | 7690 | hCoV-19/USA/PA-VSP5217/2021 | OK247007 |
| VSP5219 | B.1.617.2 | 7/14/21 | surveillance | 98.7 | 3750 | hCoV-19/USA/PA-VSP5219/2021 | OK247008 |

| | | | | | | | |
|---|---|---|---|---|---|---|---|
| VSP5221 | B.1.617.2 | 7/15/21 | surveillance | 99.7 | 4243 | hCoV-19/USA/PA-VSP5221/2021 | OK247009 |
| VSP5222 | B.1.617.2 | 7/15/21 | surveillance | 97.1 | 2190 | hCoV-19/USA/PA-VSP5222/2021 | OK247010 |
| VSP5223 | AY.14 | 7/15/21 | surveillance | 99.6 | 8230 | hCoV-19/USA/PA-VSP5223/2021 | OK247011 |
| VSP5224 | AY.25 | 7/15/21 | surveillance | 99.6 | 2912 | hCoV-19/USA/PA-VSP5224/2021 | OK247012 |
| VSP5225 | AY.25 | 7/15/21 | surveillance | 99.6 | 869 | hCoV-19/USA/PA-VSP5225/2021 | OK247013 |
| VSP5226 | B.1.617.2 | 7/15/21 | surveillance | 99.1 | 2216 | hCoV-19/USA/PA-VSP5226/2021 | OK247014 |
| VSP5227 | B.1.617.2 | 7/15/21 | surveillance | 99.6 | 3741 | hCoV-19/USA/PA-VSP5227/2021 | OK247015 |
| VSP5228 | AY.25 | 7/15/21 | surveillance | 99.1 | 8295 | hCoV-19/USA/PA-VSP5228/2021 | OK247016 |
| VSP5229 | B.1.1.7 | 7/16/21 | surveillance | 99.7 | 3075 | hCoV-19/USA/PA-VSP5229/2021 | OK247017 |
| VSP5230 | AY.12 | 7/16/21 | surveillance | 99.6 | 5139 | hCoV-19/USA/PA-VSP5230/2021 | OK247018 |
| VSP5231 | B.1.617.2 | 7/16/21 | surveillance | 97.5 | 2201 | hCoV-19/USA/PA-VSP5231/2021 | OK247019 |
| VSP5232 | B.1.617.2 | 7/16/21 | surveillance | 99.1 | 1289 | hCoV-19/USA/PA-VSP5232/2021 | OK247020 |
| VSP5235 | AY.21 | 7/10/21 | surveillance | 97.5 | 2028 | hCoV-19/USA/PA-VSP5235/2021 | OK247021 |
| VSP5242 | B.1.628 | 7/16/21 | surveillance | 99.5 | 2353 | hCoV-19/USA/PA-VSP5242/2021 | OK247022 |
| VSP5243 | B.1.1.528 | 7/16/21 | surveillance | 97.6 | 253 | hCoV-19/USA/PA-VSP5243/2021 | OK247023 |
| VSP5244 | B.1.617.2 | 7/17/21 | surveillance | 99.4 | 2449 | hCoV-19/USA/PA-VSP5244/2021 | OK247024 |
| VSP5245 | B.1.617.2 | 7/17/21 | surveillance | 99.7 | 10098 | hCoV-19/USA/PA-VSP5245/2021 | OK247025 |
| VSP5246 | None | 7/17/21 | surveillance | 96.2 | 370 | hCoV-19/USA/PA-VSP5246/2021 | OK247026 |
| VSP5247 | AY.3 | 7/17/21 | surveillance | 99.7 | 7041 | hCoV-19/USA/PA-VSP5247/2021 | OK247027 |
| VSP5248 | B.1.617.2 | 7/17/21 | surveillance | 99.7 | 4841 | hCoV-19/USA/PA-VSP5248/2021 | OK247028 |
| VSP5249 | B.1.617.2 | 7/17/21 | surveillance | 99.7 | 8336 | hCoV-19/USA/PA-VSP5249/2021 | OK247029 |
| VSP5251 | B.1.617.2 | 7/17/21 | surveillance | 99.7 | 7288 | hCoV-19/USA/PA-VSP5251/2021 | OK247030 |
| VSP5252 | B.1.617.2 | 7/17/21 | surveillance | 99.7 | 3249 | hCoV-19/USA/PA-VSP5252/2021 | OK247031 |
| VSP5253 | B.1.617.2 | 7/17/21 | surveillance | 99.6 | 2439 | hCoV-19/USA/GA-VSP5253/2021 | OK247032 |
| VSP5254 | AY.24 | 7/19/21 | surveillance | 99.6 | 1320 | hCoV-19/USA/PA-VSP5254/2021 | OK247033 |
| VSP5255 | B.1.617.2 | 7/19/21 | surveillance | 95.8 | 1064 | hCoV-19/USA/PA-VSP5255/2021 | OK247034 |
| VSP5256 | B.1.617.2 | 7/19/21 | surveillance | 99.7 | 8039 | hCoV-19/USA/PA-VSP5256/2021 | OK247035 |
| VSP5257 | B.1.617.2 | 7/19/21 | surveillance | 99.7 | 2217 | hCoV-19/USA/PA-VSP5257/2021 | OK247036 |
| VSP5258 | AY.12 | 7/20/21 | surveillance | 99.7 | 12343 | hCoV-19/USA/PA-VSP5258/2021 | OK247037 |
| VSP5259 | B.1.617.2 | 7/20/21 | surveillance | 93.8 | 217 | hCoV-19/USA/PA-VSP5259/2021 | OK247038 |
| VSP5260 | B.1.617.2 | 7/20/21 | surveillance | 99.7 | 2235 | hCoV-19/USA/CA-VSP5260/2021 | OK247039 |
| VSP5270 | AY.3 | 7/22/21 | surveillance | 99.5 | 4220 | hCoV-19/USA/PA-VSP5270/2021 | OK247040 |
| VSP5272 | AY.12 | 7/22/21 | surveillance | 99.7 | 4194 | hCoV-19/USA/PA-VSP5272/2021 | OK247041 |
| VSP5292 | B.1.617.2 | 7/15/21 | surveillance | 99.7 | 4353 | hCoV-19/USA/PA-VSP5292/2021 | OK247042 |
| VSP5293 | B.1.617.2 | 7/14/21 | surveillance | 99.7 | 8512 | hCoV-19/USA/PA-VSP5293/2021 | OK247043 |
| VSP5294 | AY.14 | 7/23/21 | surveillance | 99.7 | 2489 | hCoV-19/USA/NJ-VSP5294/2021 | OK247044 |
| VSP5295 | B.1.617.2 | 7/23/21 | surveillance | 99.6 | 3219 | hCoV-19/USA/PA-VSP5295/2021 | OK247045 |
| VSP5296 | B.1.617.2 | 7/23/21 | surveillance | 99.3 | 1833 | hCoV-19/USA/PA-VSP5296/2021 | OK247046 |
| VSP5298 | B.1.617.2 | 7/23/21 | surveillance | 99.7 | 2967 | hCoV-19/USA/PA-VSP5298/2021 | OK247047 |
| VSP5299 | AY.20 | 7/23/21 | surveillance | 99.7 | 3346 | hCoV-19/USA/PA-VSP5299/2021 | OK247048 |
| VSP5300 | B.1.617.2 | 7/23/21 | surveillance | 98.8 | 395 | hCoV-19/USA/PA-VSP5300/2021 | OK247049 |
| VSP5301 | B.1.617.2 | 7/24/21 | surveillance | 99.7 | 1265 | hCoV-19/USA/PA-VSP5301/2021 | OK247050 |
| VSP5302 | B.1.617.2 | 7/24/21 | surveillance | 99.7 | 1653 | hCoV-19/USA/PA-VSP5302/2021 | OK247051 |
| VSP5303 | B.1.617.2 | 7/26/21 | surveillance | 99.5 | 561 | hCoV-19/USA/PA-VSP5303/2021 | OK247052 |
| VSP5304 | B.1.617.2 | 7/26/21 | surveillance | 99.6 | 3561 | hCoV-19/USA/PA-VSP5304/2021 | OK247053 |
| VSP5305 | B.1.617.2 | 7/26/21 | surveillance | 99.7 | 4399 | hCoV-19/USA/PA-VSP5305/2021 | OK247054 |
| VSP5306 | AY.25 | 7/26/21 | surveillance | 97.9 | 3644 | hCoV-19/USA/PA-VSP5306/2021 | OK247055 |

| | | | | | | | |
|---|---|---|---|---|---|---|---|
| VSP5372 | B.1.617.2 | 7/19/21 | surveillance | 99.7 | 1867 | hCoV-19/USA/PA-VSP5372/2021 | OK247056 |
| VSP5373 | AY.25 | 7/17/21 | surveillance | 99.7 | 3441 | hCoV-19/USA/PA-VSP5373/2021 | OK247057 |
| VSP5374 | B.1.617.2 | 7/18/21 | surveillance | 99.7 | 14180 | hCoV-19/USA/PA-VSP5374/2021 | OK247058 |
| VSP5375 | B.1.617.2 | 7/18/21 | surveillance | 99.6 | 927 | hCoV-19/USA/PA-VSP5375/2021 | OK247059 |
| VSP5376 | AY.24 | 7/21/21 | surveillance | 99.6 | 3411 | hCoV-19/USA/PA-VSP5376/2021 | OK247060 |
| VSP5377 | AY.25 | 7/24/21 | surveillance | 99.7 | 2685 | hCoV-19/USA/PA-VSP5377/2021 | OK247061 |
| VSP5378 | AY.3 | 7/26/21 | surveillance | 99.6 | 3481 | hCoV-19/USA/PA-VSP5378/2021 | OK247062 |
| VSP5379 | AY.20 | 7/27/21 | surveillance | 98.9 | 2565 | hCoV-19/USA/PA-VSP5379/2021 | OK247063 |
| VSP5380 | B.1.617.2 | 7/27/21 | surveillance | 99.7 | 2688 | hCoV-19/USA/PA-VSP5380/2021 | OK247064 |
| VSP5381 | AY.12 | 7/27/21 | surveillance | 99.7 | 3164 | hCoV-19/USA/PA-VSP5381/2021 | OK247065 |
| VSP5382 | AY.25 | 7/28/21 | surveillance | 99.7 | 3428 | hCoV-19/USA/PA-VSP5382/2021 | OK247066 |
| VSP5383 | AY.24 | 7/29/21 | surveillance | 99.6 | 5426 | hCoV-19/USA/PA-VSP5383/2021 | OK247067 |
| VSP5385 | B.1.617.2 | 7/29/21 | surveillance | 99.4 | 1193 | hCoV-19/USA/PA-VSP5385/2021 | OK247068 |
| VSP5386 | AY.3 | 7/29/21 | surveillance | 99.7 | 4040 | hCoV-19/USA/PA-VSP5386/2021 | OK247069 |
| VSP5389 | B.1.617.2 | 7/30/21 | surveillance | 99.7 | 2918 | hCoV-19/USA/PA-VSP5389/2021 | OK247070 |
| VSP5395 | B.1.617.2 | 7/30/21 | surveillance | 99.7 | 2256 | hCoV-19/USA/PA-VSP5395/2021 | OK247071 |
| VSP5399 | AY.3 | 7/31/21 | surveillance | 99.6 | 3059 | hCoV-19/USA/PA-VSP5399/2021 | OK247072 |
| VSP5443 | B.1.617.2 | 8/4/21 | surveillance | 99.7 | 3239 | hCoV-19/USA/PA-VSP5443/2021 | OK247073 |
| VSP5444 | AY.4 | 8/4/21 | surveillance | 99.7 | 4316 | hCoV-19/USA/PA-VSP5444/2021 | OK247074 |
| VSP5445 | AY.3 | 8/4/21 | surveillance | 99.4 | 3887 | hCoV-19/USA/PA-VSP5445/2021 | OK247075 |
| VSP5446 | AY.12 | 8/4/21 | surveillance | 99.7 | 5185 | hCoV-19/USA/NJ-VSP5446/2021 | OK247076 |
| VSP5447 | B.1.617.2 | 8/4/21 | surveillance | 99.7 | 3500 | hCoV-19/USA/PA-VSP5447/2021 | OK247077 |
| VSP5448 | B.1.617.2 | 8/5/21 | surveillance | 99.7 | 5292 | hCoV-19/USA/PA-VSP5448/2021 | OK247078 |
| VSP5449 | AY.24 | 8/5/21 | surveillance | 99.1 | 796 | hCoV-19/USA/PA-VSP5449/2021 | OK247079 |
| VSP5450 | B.1.617.2 | 8/5/21 | surveillance | 98.8 | 627 | hCoV-19/USA/PA-VSP5450/2021 | OK247080 |
| VSP5451 | B.1.617.2 | 8/5/21 | surveillance | 99.7 | 3640 | hCoV-19/USA/NJ-VSP5451/2021 | OK247081 |
| VSP5452 | B.1.617.2 | 8/5/21 | surveillance | 99.7 | 3726 | hCoV-19/USA/PA-VSP5452/2021 | OK247082 |
| VSP5453 | B.1.617.2 | 8/5/21 | surveillance | 99.6 | 3470 | hCoV-19/USA/PA-VSP5453/2021 | OK247083 |
| VSP5454 | AY.3 | 8/5/21 | surveillance | 99.1 | 1326 | hCoV-19/USA/PA-VSP5454/2021 | OK247084 |
| VSP5455 | AY.25 | 8/5/21 | surveillance | 98.9 | 1479 | hCoV-19/USA/NJ-VSP5455/2021 | OK247085 |
| VSP5456 | AY.12 | 8/5/21 | surveillance | 99.7 | 4372 | hCoV-19/USA/NJ-VSP5456/2021 | OK247086 |
| VSP5458 | AY.25 | 8/6/21 | surveillance | 99.7 | 4384 | hCoV-19/USA/PA-VSP5458/2021 | OK247088 |
| VSP5460 | AY.25 | 8/6/21 | surveillance | 99.7 | 3964 | hCoV-19/USA/PA-VSP5460/2021 | OK247090 |
| VSP5461 | AY.3 | 8/6/21 | surveillance | 99.6 | 3585 | hCoV-19/USA/MD-VSP5461/2021 | OK247091 |
| VSP5462 | B.1.617.2 | 8/6/21 | surveillance | 99.7 | 3901 | hCoV-19/USA/PA-VSP5462/2021 | OK247092 |
| VSP5465 | AY.10 | 8/6/21 | surveillance | 97 | 214 | hCoV-19/USA/PA-VSP5465/2021 | OK247093 |
| VSP5466 | AY.12 | 8/6/21 | surveillance | 98.1 | 414 | hCoV-19/USA/PA-VSP5466/2021 | OK247094 |
| VSP5467 | B.1.617.2 | 7/21/21 | surveillance | 99.7 | 3135 | hCoV-19/USA/PA-VSP5467/2021 | OK247095 |
| VSP5468 | B.1.617.2 | 7/21/21 | surveillance | 98.5 | 3581 | hCoV-19/USA/PA-VSP5468/2021 | OK247096 |
| VSP5471 | AY.4 | 7/24/21 | surveillance | 95.8 | 1200 | hCoV-19/USA/PA-VSP5471/2021 | OK247097 |
| VSP5472 | B.1.617.2 | 7/25/21 | surveillance | 97.4 | 1341 | hCoV-19/USA/PA-VSP5472/2021 | OK247098 |
| VSP5473 | B.1.617.2 | 7/25/21 | surveillance | 99.3 | 982 | hCoV-19/USA/PA-VSP5473/2021 | OK247099 |
| VSP5474 | AY.25 | 7/25/21 | surveillance | 99.6 | 3808 | hCoV-19/USA/PA-VSP5474/2021 | OK247100 |
| VSP5477 | B.1.617.2 | 7/26/21 | surveillance | 99.5 | 2098 | hCoV-19/USA/PA-VSP5477/2021 | OK247101 |
| VSP5478 | B.1.617.2 | 7/26/21 | surveillance | 99.7 | 3560 | hCoV-19/USA/PA-VSP5478/2021 | OK247102 |
| VSP5479 | AY.21 | 7/26/21 | surveillance | 99.7 | 3483 | hCoV-19/USA/PA-VSP5479/2021 | OK247103 |
| VSP5481 | B.1.617.2 | 7/26/21 | surveillance | 96.2 | 1926 | hCoV-19/USA/PA-VSP5481/2021 | OK247104 |

| | | | | | | | |
|---|---|---|---|---|---|---|---|
| VSP5484 | B.1.617.2 | 7/29/21 | surveillance | 96.4 | 3963 | hCoV-19/USA/PA-VSP5484/2021 | OK247105 |
| VSP5485 | B.1.617.2 | 7/29/21 | surveillance | 99.4 | 3618 | hCoV-19/USA/PA-VSP5485/2021 | OK247106 |
| VSP5490 | B.1.617.2 | 8/1/21 | surveillance | 97.9 | 474 | hCoV-19/USA/PA-VSP5490/2021 | OK247107 |
| VSP5491 | AY.14 | 8/1/21 | surveillance | 98.1 | 1003 | hCoV-19/USA/PA-VSP5491/2021 | OK247108 |
| VSP5495 | B.1.617.2 | 8/2/21 | surveillance | 97.2 | 3723 | hCoV-19/USA/PA-VSP5495/2021 | OK247109 |
| VSP5496 | AY.20 | 8/3/21 | surveillance | 99.5 | 13645 | hCoV-19/USA/PA-VSP5496/2021 | OK247110 |
| VSP5503 | B.1.617.2 | 8/7/21 | surveillance | 99.5 | 3451 | hCoV-19/USA/PA-VSP5503/2021 | OK247111 |
| VSP5504 | B.1.617.2 | 8/7/21 | surveillance | 99.7 | 3429 | hCoV-19/USA/PA-VSP5504/2021 | OK247112 |
| VSP5505 | AY.3 | 8/7/21 | surveillance | 99.7 | 3456 | hCoV-19/USA/PA-VSP5505/2021 | OK247113 |
| VSP5507 | AY.14 | 8/7/21 | surveillance | 95.6 | 1314 | hCoV-19/USA/PA-VSP5507/2021 | OK247114 |
| VSP5508 | B.1.617.2 | 8/7/21 | surveillance | 99.6 | 6431 | hCoV-19/USA/PA-VSP5508/2021 | OK247115 |
| VSP5509 | AY.4 | 8/7/21 | surveillance | 96.2 | 4197 | hCoV-19/USA/PA-VSP5509/2021 | OK247116 |
| VSP5510 | AY.25 | 8/7/21 | surveillance | 98.8 | 5726 | hCoV-19/USA/PA-VSP5510/2021 | OK247117 |
| VSP5511 | AY.3 | 8/8/21 | surveillance | 99.2 | 5716 | hCoV-19/USA/PA-VSP5511/2021 | OK247118 |
| VSP5512 | AY.3 | 8/8/21 | surveillance | 95.8 | 2319 | hCoV-19/USA/PA-VSP5512/2021 | OK247119 |
| VSP5513 | AY.3 | 8/8/21 | surveillance | 99.7 | 4328 | hCoV-19/USA/PA-VSP5513/2021 | OK247120 |
| VSP5514 | AY.25 | 8/8/21 | surveillance | 99.4 | 4281 | hCoV-19/USA/PA-VSP5514/2021 | OK247121 |
| VSP5515 | AY.3 | 8/8/21 | surveillance | 98.9 | 3866 | hCoV-19/USA/PA-VSP5515/2021 | OK247122 |
| VSP5516 | B.1.617.2 | 8/9/21 | surveillance | 99.7 | 3408 | hCoV-19/USA/PA-VSP5516/2021 | OK247123 |
| VSP5517 | AY.25 | 8/9/21 | surveillance | 99.7 | 4109 | hCoV-19/USA/PA-VSP5517/2021 | OK247124 |
| VSP5518 | B.1.617.2 | 8/9/21 | surveillance | 99.6 | 1611 | hCoV-19/USA/PA-VSP5518/2021 | OK247125 |
| VSP5519 | B.1.617.2 | 8/9/21 | surveillance | 99.7 | 4541 | hCoV-19/USA/PA-VSP5519/2021 | OK247126 |
| VSP5520 | B.1.617.2 | 8/9/21 | surveillance | 99.6 | 2115 | hCoV-19/USA/PA-VSP5520/2021 | OK247127 |
| VSP5521 | AY.25 | 8/9/21 | surveillance | 99.7 | 4860 | hCoV-19/USA/NJ-VSP5521/2021 | OK247128 |
| VSP5525 | B.1.617.2 | 8/9/21 | surveillance | 99.1 | 2954 | hCoV-19/USA/PA-VSP5525/2021 | OK247129 |
| VSP5526 | B.1.617.2 | 8/9/21 | surveillance | 99.7 | 3508 | hCoV-19/USA/PA-VSP5526/2021 | OK247130 |
| VSP5527 | AY.10 | 8/9/21 | surveillance | 99.3 | 5238 | hCoV-19/USA/PA-VSP5527/2021 | OK247131 |
| VSP5528 | AY.14 | 8/9/21 | surveillance | 99.7 | 5282 | hCoV-19/USA/PA-VSP5528/2021 | OK247132 |
| VSP5529 | AY.25 | 8/9/21 | surveillance | 99.6 | 3376 | hCoV-19/USA/PA-VSP5529/2021 | OK247133 |
| VSP5530 | B.1.617.2 | 8/9/21 | surveillance | 99.7 | 2782 | hCoV-19/USA/CA-VSP5530/2021 | OK247134 |
| VSP5531 | B.1.617.2 | 8/9/21 | surveillance | 99.4 | 4261 | hCoV-19/USA/PA-VSP5531/2021 | OK247135 |
| VSP5532 | AY.15 | 8/9/21 | surveillance | 99.7 | 4230 | hCoV-19/USA/PA-VSP5532/2021 | OK247136 |
| VSP5533 | B.1.617.2 | 8/9/21 | surveillance | 99.7 | 3827 | hCoV-19/USA/PA-VSP5533/2021 | OK247137 |
| VSP5534 | B.1.617.2 | 8/1/21 | surveillance | 99.6 | 3252 | hCoV-19/USA/PA-VSP5534/2021 | OK247138 |
| VSP5535 | B.1.617.2 | 8/1/21 | surveillance | 99.1 | 2266 | hCoV-19/USA/PA-VSP5535/2021 | OK247139 |
| VSP5536 | B.1.617.2 | 8/3/21 | surveillance | 98 | 1733 | hCoV-19/USA/PA-VSP5536/2021 | OK247140 |
| VSP5537 | B.1.617.2 | 8/4/21 | surveillance | 99.7 | 3214 | hCoV-19/USA/PA-VSP5537/2021 | OK247141 |
| VSP5539 | AY.13 | 8/6/21 | surveillance | 99.6 | 4420 | hCoV-19/USA/NJ-VSP5539/2021 | OK247142 |
| VSP5540 | B.1.617.2 | 8/6/21 | surveillance | 98.4 | 580 | hCoV-19/USA/PA-VSP5540/2021 | OK247143 |
| VSP5542 | P.1 | 8/6/21 | surveillance | 99.7 | 2732 | hCoV-19/USA/PA-VSP5542/2021 | OK247144 |
| VSP5543 | B.1.617.2 | 8/6/21 | surveillance | 99.7 | 3808 | hCoV-19/USA/NJ-VSP5543/2021 | OK247145 |
| VSP5544 | AY.3 | 8/7/21 | surveillance | 99.7 | 8082 | hCoV-19/USA/PA-VSP5544/2021 | OK247146 |
| VSP5545 | AY.20 | 8/7/21 | surveillance | 99.7 | 5226 | hCoV-19/USA/PA-VSP5545/2021 | OK247147 |
| VSP5546 | B.1.617.2 | 8/7/21 | surveillance | 99.7 | 4381 | hCoV-19/USA/NJ-VSP5546/2021 | OK247148 |
| VSP5548 | B.1.617.2 | 8/7/21 | surveillance | 99.6 | 4924 | hCoV-19/USA/NJ-VSP5548/2021 | OK247149 |
| VSP5549 | B.1.617.2 | 8/7/21 | surveillance | 99.4 | 1441 | hCoV-19/USA/PA-VSP5549/2021 | OK247150 |
| VSP5550 | B.1.617.2 | 8/7/21 | surveillance | 99.6 | 2143 | hCoV-19/USA/NJ-VSP5550/2021 | OK247151 |

| | | | | | | | |
|---|---|---|---|---|---|---|---|
| VSP5551 | AY.12 | 8/7/21 | surveillance | 98.4 | 436 | hCoV-19/USA/PA-VSP5551/2021 | OK247152 |
| VSP5552 | AY.25 | 8/8/21 | surveillance | 99.4 | 1533 | hCoV-19/USA/NJ-VSP5552/2021 | OK247153 |
| VSP5553 | AY.24 | 8/8/21 | surveillance | 99.6 | 2059 | hCoV-19/USA/PA-VSP5553/2021 | OK247154 |
| VSP5554 | AY.4 | 8/9/21 | surveillance | 98.9 | 4803 | hCoV-19/USA/PA-VSP5554/2021 | OK247155 |
| VSP5555 | B.1.617.2 | 8/9/21 | surveillance | 99.6 | 2557 | hCoV-19/USA/PA-VSP5555/2021 | OK247156 |
| VSP5556 | AY.1 | 8/9/21 | surveillance | 99.6 | 6629 | hCoV-19/USA/NJ-VSP5556/2021 | OK247157 |
| VSP5557 | B.1.617.2 | 8/9/21 | surveillance | 99.7 | 6338 | hCoV-19/USA/PA-VSP5557/2021 | OK247158 |
| VSP5558 | AY.25 | 8/9/21 | surveillance | 98.9 | 1413 | hCoV-19/USA/NJ-VSP5558/2021 | OK247159 |
| VSP5559 | AY.1 | 8/9/21 | surveillance | 99.6 | 3557 | hCoV-19/USA/PA-VSP5559/2021 | OK247160 |
| VSP5560 | B.1.617.2 | 8/9/21 | surveillance | 99.4 | 1361 | hCoV-19/USA/PA-VSP5560/2021 | OK247161 |
| VSP5561 | B.1.617.2 | 8/9/21 | surveillance | 99.2 | 1320 | hCoV-19/USA/PA-VSP5561/2021 | OK247162 |
| VSP5562 | AY.20 | 8/9/21 | surveillance | 98.9 | 2104 | hCoV-19/USA/NJ-VSP5562/2021 | OK247163 |
| VSP5563 | B.1.617.2 | 8/10/21 | surveillance | 99.5 | 3357 | hCoV-19/USA/PA-VSP5563/2021 | OK247164 |
| VSP5564 | AY.20 | 8/10/21 | surveillance | 99.4 | 1432 | hCoV-19/USA/PA-VSP5564/2021 | OK247165 |
| VSP5566 | AY.24 | 8/10/21 | surveillance | 97.4 | 177 | hCoV-19/USA/PA-VSP5566/2021 | OK247166 |
| VSP5568 | AY.4 | 8/10/21 | surveillance | 98.8 | 2148 | hCoV-19/USA/PA-VSP5568/2021 | OK247167 |
| VSP5569 | AY.25 | 8/10/21 | surveillance | 99.7 | 2581 | hCoV-19/USA/NJ-VSP5569/2021 | OK247168 |
| VSP5570 | AY.4 | 8/10/21 | surveillance | 98 | 845 | hCoV-19/USA/PA-VSP5570/2021 | OK247169 |
| VSP5571 | AY.24 | 8/10/21 | surveillance | 99.4 | 2051 | hCoV-19/USA/PA-VSP5571/2021 | OK247170 |
| VSP5572 | B.1.621.1 | 8/10/21 | surveillance | 99.6 | 782 | hCoV-19/USA/PA-VSP5572/2021 | OK247171 |
| VSP5573 | AY.25 | 8/10/21 | surveillance | 99.6 | 4820 | hCoV-19/USA/PA-VSP5573/2021 | OK247172 |
| VSP5575 | B.1.617.2 | 8/10/21 | surveillance | 99.7 | 3889 | hCoV-19/USA/PA-VSP5575/2021 | OK247173 |
| VSP5576 | AY.25 | 8/10/21 | surveillance | 98 | 2944 | hCoV-19/USA/PA-VSP5576/2021 | OK247174 |
| VSP5577 | AY.14 | 8/10/21 | surveillance | 99.7 | 3582 | hCoV-19/USA/NJ-VSP5577/2021 | OK247175 |
| VSP5578 | AY.3 | 8/10/21 | surveillance | 98.6 | 784 | hCoV-19/USA/PA-VSP5578/2021 | OK247176 |
| VSP5580 | AY.4 | 8/10/21 | surveillance | 98.1 | 3143 | hCoV-19/USA/PA-VSP5580/2021 | OK247177 |
| VSP5581 | B.1.617.2 | 8/10/21 | surveillance | 99.6 | 3061 | hCoV-19/USA/PA-VSP5581/2021 | OK247178 |
| VSP5582 | AY.25 | 8/10/21 | surveillance | 99.3 | 2118 | hCoV-19/USA/PA-VSP5582/2021 | OK247179 |
| VSP5583 | AY.24 | 8/10/21 | surveillance | 99.6 | 2055 | hCoV-19/USA/PA-VSP5583/2021 | OK247180 |
| VSP5584 | B.1.617.2 | 8/10/21 | surveillance | 99.7 | 3742 | hCoV-19/USA/NJ-VSP5584/2021 | OK247181 |
| VSP5585 | AY.14 | 8/10/21 | surveillance | 99.1 | 1041 | hCoV-19/USA/NJ-VSP5585/2021 | OK247182 |
| VSP5586 | AY.12 | 8/10/21 | surveillance | 99.4 | 2231 | hCoV-19/USA/PA-VSP5586/2021 | OK247183 |
| VSP5587 | B.1.617.2 | 8/11/21 | surveillance | 99.7 | 7646 | hCoV-19/USA/PA-VSP5587/2021 | OK247184 |
| VSP5589 | B.1.617.2 | 8/11/21 | surveillance | 99.2 | 1696 | hCoV-19/USA/NJ-VSP5589/2021 | OK247185 |
| VSP5590 | AY.25 | 8/11/21 | surveillance | 98.5 | 2635 | hCoV-19/USA/PA-VSP5590/2021 | OK247186 |
| VSP5592 | AY.4 | 8/11/21 | surveillance | 97.5 | 1837 | hCoV-19/USA/PA-VSP5592/2021 | OK247187 |
| VSP5596 | AY.3 | 8/11/21 | surveillance | 98.1 | 1996 | hCoV-19/USA/PA-VSP5596/2021 | OK247188 |
| VSP5598 | AY.15 | 8/11/21 | surveillance | 99.7 | 2861 | hCoV-19/USA/PA-VSP5598/2021 | OK247189 |
| VSP5599 | B.1.617.2 | 8/11/21 | surveillance | 99.6 | 3714 | hCoV-19/USA/PA-VSP5599/2021 | OK247190 |
| VSP5600 | B.1.617.2 | 8/11/21 | surveillance | 99.7 | 2390 | hCoV-19/USA/PA-VSP5600/2021 | OK247191 |
| VSP5601 | AY.20 | 8/11/21 | surveillance | 99.6 | 1487 | hCoV-19/USA/PA-VSP5601/2021 | OK247192 |
| VSP5604 | AY.12 | 8/11/21 | surveillance | 97.8 | 756 | hCoV-19/USA/VA-VSP5604/2021 | OK247193 |
| VSP5605 | B.1.617.2 | 8/11/21 | surveillance | 99.1 | 1351 | hCoV-19/USA/NJ-VSP5605/2021 | OK247194 |
| VSP5606 | B.1.617.2 | 8/11/21 | surveillance | 98.3 | 3852 | hCoV-19/USA/PA-VSP5606/2021 | OK247195 |
| VSP5609 | B.1.617.2 | 8/12/21 | surveillance | 95.2 | 697 | hCoV-19/USA/NJ-VSP5609/2021 | OK247196 |
| VSP5610 | B.1.617.2 | 8/12/21 | surveillance | 99.5 | 3719 | hCoV-19/USA/PA-VSP5610/2021 | OK247197 |
| VSP5611 | B.1.617.2 | 8/12/21 | surveillance | 96.3 | 887 | hCoV-19/USA/PA-VSP5611/2021 | OK247198 |

| | | | | | | | |
|---|---|---|---|---|---|---|---|
| VSP5612 | B.1.617.2 | 8/12/21 | surveillance | 98.3 | 2628 | hCoV-19/USA/NJ-VSP5612/2021 | OK247199 |
| VSP5614 | B.1.617.2 | 8/12/21 | surveillance | 98.8 | 530 | hCoV-19/USA/PA-VSP5614/2021 | OK247200 |
| VSP5615 | AY.4 | 8/12/21 | surveillance | 98.8 | 3541 | hCoV-19/USA/PA-VSP5615/2021 | OK247201 |
| VSP5616 | AY.14 | 8/12/21 | surveillance | 99.7 | 3042 | hCoV-19/USA/NJ-VSP5616/2021 | OK247202 |
| VSP5617 | B.1.617.2 | 8/12/21 | surveillance | 99.7 | 2958 | hCoV-19/USA/NJ-VSP5617/2021 | OK247203 |
| VSP5618 | AY.3 | 8/12/21 | surveillance | 98.5 | 713 | hCoV-19/USA/PA-VSP5618/2021 | OK247204 |
| VSP5619 | AY.2 | 8/12/21 | surveillance | 98.9 | 2864 | hCoV-19/USA/PA-VSP5619/2021 | OK247205 |
| VSP5620 | B.1.617.2 | 8/12/21 | surveillance | 99.5 | 2301 | hCoV-19/USA/PA-VSP5620/2021 | OK247206 |
| VSP5621 | B.1.617.2 | 8/12/21 | surveillance | 99.3 | 1289 | hCoV-19/USA/SC-VSP5621/2021 | OK247207 |
| VSP5622 | AY.25 | 8/12/21 | surveillance | 98.8 | 815 | hCoV-19/USA/PA-VSP5622/2021 | OK247208 |
| VSP5623 | B.1.617.2 | 8/12/21 | surveillance | 99.3 | 1681 | hCoV-19/USA/NJ-VSP5623/2021 | OK247209 |
| VSP5625 | B.1.617.2 | 8/12/21 | surveillance | 99.3 | 3672 | hCoV-19/USA/PA-VSP5625/2021 | OK247210 |
| VSP5627 | B.1.617.2 | 8/12/21 | surveillance | 99.7 | 2141 | hCoV-19/USA/NJ-VSP5627/2021 | OK247211 |
| VSP5628 | AY.25 | 8/12/21 | surveillance | 98.7 | 673 | hCoV-19/USA/NJ-VSP5628/2021 | OK247212 |
| VSP5629 | B.1.617.2 | 8/12/21 | surveillance | 98 | 4430 | hCoV-19/USA/PA-VSP5629/2021 | OK247213 |
| VSP5630 | AY.3 | 8/12/21 | surveillance | 98.7 | 1457 | hCoV-19/USA/NJ-VSP5630/2021 | OK247214 |
| VSP5631 | AY.3 | 8/13/21 | surveillance | 99.1 | 834 | hCoV-19/USA/PA-VSP5631/2021 | OK247215 |
| VSP5632 | B.1.617.2 | 8/13/21 | surveillance | 99.5 | 1168 | hCoV-19/USA/PA-VSP5632/2021 | OK247216 |
| VSP5633 | B.1.617.2 | 8/13/21 | surveillance | 99.6 | 1371 | hCoV-19/USA/PA-VSP5633/2021 | OK247217 |
| VSP5634 | AY.25 | 8/13/21 | surveillance | 99.7 | 10558 | hCoV-19/USA/PA-VSP5634/2021 | OK247218 |
| VSP5635 | B.1.617.2 | 8/13/21 | surveillance | 99.6 | 1857 | hCoV-19/USA/PA-VSP5635/2021 | OK247219 |
| VSP5636 | B.1.617.2 | 8/13/21 | surveillance | 99.5 | 1323 | hCoV-19/USA/PA-VSP5636/2021 | OK247220 |
| VSP5637 | AY.25 | 8/13/21 | surveillance | 99 | 947 | hCoV-19/USA/PA-VSP5637/2021 | OK247221 |
| VSP5639 | AY.25 | 8/13/21 | surveillance | 98.8 | 1188 | hCoV-19/USA/PA-VSP5639/2021 | OK247223 |
| VSP5640 | AY.3 | 8/13/21 | surveillance | 98.8 | 830 | hCoV-19/USA/PA-VSP5640/2021 | OK247224 |
| VSP5641 | B.1.617.2 | 8/13/21 | surveillance | 99.1 | 1558 | hCoV-19/USA/PA-VSP5641/2021 | OK247225 |
| VSP5642 | B.1.617.2 | 8/13/21 | surveillance | 98.7 | 251 | hCoV-19/USA/PA-VSP5642/2021 | OK247226 |
| VSP5643 | AY.25 | 8/13/21 | surveillance | 99.5 | 757 | hCoV-19/USA/PA-VSP5643/2021 | OK247227 |
| VSP5644 | AY.4 | 8/13/21 | surveillance | 99.6 | 930 | hCoV-19/USA/NJ-VSP5644/2021 | OK247228 |
| VSP5645 | B.1.617.2 | 8/13/21 | surveillance | 98.8 | 823 | hCoV-19/USA/PA-VSP5645/2021 | OK247229 |
| VSP5646 | B.1.617.2 | 8/13/21 | surveillance | 99.7 | 8406 | hCoV-19/USA/PA-VSP5646/2021 | OK247230 |
| VSP5647 | B.1.617.2 | 8/13/21 | surveillance | 99.5 | 1175 | hCoV-19/USA/PA-VSP5647/2021 | OK247231 |
| VSP5648 | AY.15 | 8/13/21 | surveillance | 99.6 | 4054 | hCoV-19/USA/PA-VSP5648/2021 | OK247232 |
| VSP5649 | B.1.617.2 | 8/13/21 | surveillance | 99.2 | 1030 | hCoV-19/USA/PA-VSP5649/2021 | OK247233 |
| VSP5650 | B.1.617.2 | 8/13/21 | surveillance | 99.4 | 1783 | hCoV-19/USA/PA-VSP5650/2021 | OK247234 |
| VSP5651 | B.1.617.2 | 8/13/21 | surveillance | 99.1 | 1649 | hCoV-19/USA/PA-VSP5651/2021 | OK247235 |
| VSP5652 | AY.25 | 8/13/21 | surveillance | 98.9 | 528 | hCoV-19/USA/PA-VSP5652/2021 | OK247236 |
| VSP5653 | AY.3 | 8/13/21 | surveillance | 98.8 | 1440 | hCoV-19/USA/NJ-VSP5653/2021 | OK247237 |
| VSP5654 | B.1.617.2 | 8/13/21 | surveillance | 98.8 | 870 | hCoV-19/USA/NJ-VSP5654/2021 | OK247238 |
| VSP5656 | AY.25 | 8/14/21 | surveillance | 98.1 | 116 | hCoV-19/USA/PA-VSP5656/2021 | OK247240 |
| VSP5658 | AY.3 | 8/14/21 | surveillance | 99.6 | 3621 | hCoV-19/USA/PA-VSP5658/2021 | OK247242 |
| VSP5659 | AY.25 | 8/14/21 | surveillance | 99.2 | 573 | hCoV-19/USA/PA-VSP5659/2021 | OK247243 |
| VSP5660 | B.1.617.2 | 8/14/21 | surveillance | 99.7 | 2280 | hCoV-19/USA/PA-VSP5660/2021 | OK247244 |
| VSP5661 | B.1.617.2 | 8/14/21 | surveillance | 99.1 | 941 | hCoV-19/USA/PA-VSP5661/2021 | OK247245 |
| VSP5662 | B.1.617.2 | 8/14/21 | surveillance | 99.1 | 990 | hCoV-19/USA/PA-VSP5662/2021 | OK247246 |
| VSP5663 | AY.15 | 8/14/21 | surveillance | 99.6 | 2718 | hCoV-19/USA/PA-VSP5663/2021 | OK247247 |
| VSP5664 | B.1.617.2 | 8/14/21 | surveillance | 99.7 | 2116 | hCoV-19/USA/NJ-VSP5664/2021 | OK247248 |

| | | | | | | | |
|---|---|---|---|---|---|---|---|
| VSP5665 | B.1.617.2 | 8/14/21 | surveillance | 99.4 | 1450 | hCoV-19/USA/PA-VSP5665/2021 | OK247249 |
| VSP5666 | B.1.617.2 | 8/14/21 | surveillance | 99.4 | 1670 | hCoV-19/USA/PA-VSP5666/2021 | OK247250 |
| VSP5667 | None | 8/14/21 | surveillance | 99.7 | 716 | hCoV-19/USA/PA-VSP5667/2021 | OK247251 |
| VSP5668 | AY.25 | 8/14/21 | surveillance | 99 | 657 | hCoV-19/USA/PA-VSP5668/2021 | OK247252 |
| VSP5672 | AY.25 | 8/15/21 | surveillance | 99.7 | 7891 | hCoV-19/USA/PA-VSP5672/2021 | OK247256 |
| VSP5673 | B.1.617.2 | 8/15/21 | surveillance | 98.7 | 372 | hCoV-19/USA/PA-VSP5673/2021 | OK247257 |
| VSP5674 | AY.25 | 8/15/21 | surveillance | 99.4 | 1354 | hCoV-19/USA/PA-VSP5674/2021 | OK247258 |
| VSP5676 | B.1.617.2 | 8/15/21 | surveillance | 99.1 | 612 | hCoV-19/USA/ME-VSP5676/2021 | OK247259 |
| VSP5677 | AY.25 | 8/16/21 | surveillance | 99.3 | 1363 | hCoV-19/USA/PA-VSP5677/2021 | OK247260 |
| VSP5678 | B.1.617.2 | 8/16/21 | surveillance | 99 | 1962 | hCoV-19/USA/PA-VSP5678/2021 | OK247261 |
| VSP5679 | B.1.617.2 | 8/16/21 | surveillance | 98.9 | 865 | hCoV-19/USA/PA-VSP5679/2021 | OK247262 |
| VSP5680 | AY.3 | 8/16/21 | surveillance | 99.4 | 887 | hCoV-19/USA/PA-VSP5680/2021 | OK247263 |
| VSP5681 | AY.3 | 8/16/21 | surveillance | 99.4 | 1480 | hCoV-19/USA/PA-VSP5681/2021 | OK247264 |
| VSP5682 | AY.25 | 8/16/21 | surveillance | 99 | 1693 | hCoV-19/USA/PA-VSP5682/2021 | OK247265 |
| VSP5683 | B.1.617.2 | 8/16/21 | surveillance | 99.7 | 1090 | hCoV-19/USA/PA-VSP5683/2021 | OK247266 |
| VSP5684 | AY.3 | 8/16/21 | surveillance | 99.6 | 2033 | hCoV-19/USA/PA-VSP5684/2021 | OK247267 |
| VSP5685 | AY.25 | 8/16/21 | surveillance | 99.1 | 2168 | hCoV-19/USA/PA-VSP5685/2021 | OK247268 |
| VSP5686 | AY.3 | 8/16/21 | surveillance | 98.7 | 619 | hCoV-19/USA/PA-VSP5686/2021 | OK247269 |
| VSP5708 | AY.25 | 7/30/21 | surveillance | 99.5 | 1728 | hCoV-19/USA/PA-VSP5708/2021 | OK247272 |
| VSP5709 | AY.21 | 7/30/21 | surveillance | 99.7 | 3445 | hCoV-19/USA/PA-VSP5709/2021 | OK247273 |
| VSP5710 | B.1.617.2 | 7/31/21 | surveillance | 99.7 | 4914 | hCoV-19/USA/PA-VSP5710/2021 | OK247274 |
| VSP5712 | B.1.617.2 | 8/2/21 | surveillance | 99.6 | 4079 | hCoV-19/USA/PA-VSP5712/2021 | OK247275 |
| VSP5713 | B.1.617.2 | 8/2/21 | surveillance | 99.7 | 2800 | hCoV-19/USA/PA-VSP5713/2021 | OK247276 |
| VSP5714 | B.1.617.2 | 8/2/21 | surveillance | 98.7 | 1808 | hCoV-19/USA/PA-VSP5714/2021 | OK247277 |
| VSP5715 | AY.12 | 8/2/21 | surveillance | 99.6 | 3575 | hCoV-19/USA/PA-VSP5715/2021 | OK247278 |
| VSP5716 | AY.25 | 8/2/21 | surveillance | 99.7 | 5007 | hCoV-19/USA/PA-VSP5716/2021 | OK247279 |
| VSP5717 | B.1.617.2 | 8/2/21 | surveillance | 99.4 | 2438 | hCoV-19/USA/PA-VSP5717/2021 | OK247280 |
| VSP5718 | B.1.617.2 | 8/3/21 | surveillance | 99.7 | 2279 | hCoV-19/USA/PA-VSP5718/2021 | OK247281 |
| VSP5719 | B.1.617.2 | 8/4/21 | surveillance | 98.9 | 3087 | hCoV-19/USA/PA-VSP5719/2021 | OK247282 |
| VSP5721 | AY.25 | 8/4/21 | surveillance | 98.2 | 581 | hCoV-19/USA/PA-VSP5721/2021 | OK247283 |
| VSP5722 | B.1.617.2 | 8/4/21 | surveillance | 99.7 | 5170 | hCoV-19/USA/PA-VSP5722/2021 | OK247284 |
| VSP5723 | AY.3 | 8/4/21 | surveillance | 99.4 | 1664 | hCoV-19/USA/PA-VSP5723/2021 | OK247285 |
| VSP5724 | AY.3 | 8/5/21 | surveillance | 99 | 987 | hCoV-19/USA/PA-VSP5724/2021 | OK247286 |
| VSP5725 | B.1.617.2 | 8/5/21 | surveillance | 99.7 | 3843 | hCoV-19/USA/PA-VSP5725/2021 | OK247287 |
| VSP5727 | B.1.617.2 | 8/7/21 | surveillance | 99.7 | 4915 | hCoV-19/USA/PA-VSP5727/2021 | OK247288 |
| VSP5729 | B.1.617.2 | 8/8/21 | surveillance | 99.5 | 1528 | hCoV-19/USA/PA-VSP5729/2021 | OK247289 |
| VSP5738 | B.1.617.2 | 8/6/21 | surveillance | 98.8 | 1052 | hCoV-19/USA/PA-VSP5738/2021 | OK247290 |
| VSP5739 | B.1.617.2 | 8/9/21 | surveillance | 98.9 | 1322 | hCoV-19/USA/PA-VSP5739/2021 | OK247291 |
| VSP5740 | AY.20 | 8/9/21 | surveillance | 98.8 | 1468 | hCoV-19/USA/MI-VSP5740/2021 | OK247292 |
| VSP5742 | B.1.617.2 | 8/7/21 | surveillance | 99.6 | 1598 | hCoV-19/USA/PA-VSP5742/2021 | OK247293 |
| VSP5743 | AY.20 | 8/7/21 | surveillance | 99.5 | 1692 | hCoV-19/USA/PA-VSP5743/2021 | OK247294 |
| VSP5744 | B.1.617.2 | 8/7/21 | surveillance | 99.7 | 950 | hCoV-19/USA/PA-VSP5744/2021 | OK247295 |
| VSP5745 | AY.2 | 8/10/21 | surveillance | 99.7 | 10300 | hCoV-19/USA/PA-VSP5745/2021 | OK247296 |
| VSP5746 | AY.25 | 8/10/21 | surveillance | 98.8 | 710 | hCoV-19/USA/PA-VSP5746/2021 | OK247297 |
| VSP5747 | AY.1 | 8/10/21 | surveillance | 98.8 | 317 | hCoV-19/USA/PA-VSP5747/2021 | OK247298 |
| VSP5748 | AY.24 | 8/11/21 | surveillance | 99.7 | 16340 | hCoV-19/USA/PA-VSP5748/2021 | OK247299 |
| VSP5749 | AY.25 | 8/8/21 | surveillance | 99.6 | 2267 | hCoV-19/USA/PA-VSP5749/2021 | OK247300 |

| | | | | | | | |
|---|---|---|---|---|---|---|---|
| VSP5908 | B.1.617.2 | 8/24/21 | surveillance | 97 | 150 | hCoV-19/USA/NJ-VSP5908/2021 | OK247301 |
| VSP5910 | AY.3 | 8/24/21 | surveillance | 99.6 | 3040 | hCoV-19/USA/NJ-VSP5910/2021 | OK247302 |
| VSP5912 | B.1.617.2 | 8/26/21 | surveillance | 98.8 | 1415 | hCoV-19/USA/NJ-VSP5912/2021 | OK247303 |
| VSP5920 | B.1.617.2 | 8/28/21 | surveillance | 99.7 | 26703 | hCoV-19/USA/PA-VSP5920/2021 | OK247304 |
| VSP5928 | AY.3 | 8/28/21 | surveillance | 99.6 | 2019 | hCoV-19/USA/PA-VSP5928/2021 | OK247305 |
| VSP5929 | AY.4 | 8/28/21 | surveillance | 96.5 | 70 | hCoV-19/USA/PA-VSP5929/2021 | OK247306 |
| VSP5931 | AY.25 | 8/28/21 | surveillance | 98.6 | 573 | hCoV-19/USA/PA-VSP5931/2021 | OK247307 |
| VSP5932 | AY.25 | 8/28/21 | surveillance | 98.8 | 1252 | hCoV-19/USA/PA-VSP5932/2021 | OK247308 |
| VSP5935 | AY.3 | 8/28/21 | accine breakthroug | 99.4 | 840 | hCoV-19/USA/PA-VSP5935/2021 | OK247309 |
| VSP5936 | B.1.617.2 | 8/28/21 | surveillance | 98.5 | 2020 | hCoV-19/USA/PA-VSP5936/2021 | OK247310 |
| VSP5942 | B.1.617.2 | 8/29/21 | surveillance | 98.8 | 686 | hCoV-19/USA/PA-VSP5942/2021 | OK247311 |
| VSP5946 | B.1.617.2 | 8/29/21 | surveillance | 98.9 | 948 | hCoV-19/USA/PA-VSP5946/2021 | OK247312 |
| VSP5947 | AY.3 | 8/29/21 | surveillance | 99 | 1243 | hCoV-19/USA/PA-VSP5947/2021 | OK247313 |
| VSP5948 | AY.3 | 8/29/21 | surveillance | 97.9 | 901 | hCoV-19/USA/PA-VSP5948/2021 | OK247314 |
| VSP5949 | AY.3 | 8/29/21 | surveillance | 99 | 1095 | hCoV-19/USA/PA-VSP5949/2021 | OK247315 |
| VSP5953 | B.1.617.2 | 8/30/21 | surveillance | 98.4 | 773 | hCoV-19/USA/PA-VSP5953/2021 | OK247316 |
| VSP5954 | B.1.617.2 | 8/30/21 | surveillance | 97.8 | 101 | hCoV-19/USA/PA-VSP5954/2021 | OK247317 |
| VSP5957 | B.1.617.2 | 8/30/21 | surveillance | 99.5 | 2165 | hCoV-19/USA/NJ-VSP5957/2021 | OK247318 |
| VSP5958 | B.1.617.2 | 8/30/21 | surveillance | 98.3 | 480 | hCoV-19/USA/PA-VSP5958/2021 | OK247319 |
| VSP5959 | B.1.617.2 | 8/30/21 | surveillance | 97.6 | 147 | hCoV-19/USA/PA-VSP5959/2021 | OK247320 |
| VSP5960 | AY.3.1 | 8/30/21 | surveillance | 97.7 | 797 | hCoV-19/USA/PA-VSP5960/2021 | OK247321 |
| VSP5961 | AY.24 | 8/30/21 | surveillance | 99 | 5902 | hCoV-19/USA/PA-VSP5961/2021 | OK247322 |
| VSP5968 | B.1.617.2 | 8/31/21 | surveillance | 97.3 | 626 | hCoV-19/USA/PA-VSP5968/2021 | OK247323 |
| VSP5971 | B.1.617.2 | 8/31/21 | surveillance | 97.2 | 253 | hCoV-19/USA/PA-VSP5971/2021 | OK247324 |
| VSP5980 | AY.3 | 8/31/21 | surveillance | 99.6 | 2017 | hCoV-19/USA/PA-VSP5980/2021 | OK247325 |
| VSP5983 | B.1.617.2 | 9/1/21 | surveillance | 99 | 1005 | hCoV-19/USA/PA-VSP5983/2021 | OK247326 |
| VSP5986 | B.1.617.2 | 9/1/21 | surveillance | 98.9 | 975 | hCoV-19/USA/PA-VSP5986/2021 | OK247327 |
| VSP5987 | B.1.617.2 | 9/1/21 | surveillance | 98.9 | 874 | hCoV-19/USA/PA-VSP5987/2021 | OK247328 |
| VSP5991 | AY.25 | 9/1/21 | surveillance | 99.1 | 1684 | hCoV-19/USA/PA-VSP5991/2021 | OK247329 |
| VSP5992 | AY.24 | 9/1/21 | accine breakthroug | 98.4 | 267 | hCoV-19/USA/PA-VSP5992/2021 | OK247330 |
| VSP5996 | B.1.617.2 | 9/1/21 | surveillance | 98 | 236 | hCoV-19/USA/PA-VSP5996/2021 | OK247331 |
| VSP5997 | B.1.617.2 | 9/1/21 | surveillance | 98.1 | 379 | hCoV-19/USA/NJ-VSP5997/2021 | OK247332 |
| VSP6000 | AY.12 | 9/1/21 | surveillance | 97.6 | 130 | hCoV-19/USA/PA-VSP6000/2021 | OK247333 |
| VSP6004 | B.1.617.2 | 9/2/21 | surveillance | 98.1 | 616 | hCoV-19/USA/NJ-VSP6004/2021 | OK247334 |
| VSP6005 | B.1.617.2 | 9/2/21 | surveillance | 98.8 | 584 | hCoV-19/USA/NJ-VSP6005/2021 | OK247335 |
| VSP6007 | B.1.617.2 | 9/2/21 | surveillance | 99.7 | 3512 | hCoV-19/USA/PA-VSP6007/2021 | OK247336 |
| VSP6008 | AY.4 | 9/2/21 | surveillance | 95.2 | 63 | hCoV-19/USA/NJ-VSP6008/2021 | OK247337 |
| VSP6009 | B.1.617.2 | 9/2/21 | surveillance | 98.9 | 955 | hCoV-19/USA/PA-VSP6009/2021 | OK247338 |
| VSP6011 | AY.24 | 9/2/21 | accine breakthroug | 99.6 | 1192 | hCoV-19/USA/PA-VSP6011/2021 | OK247339 |
| VSP6013 | AY.25 | 9/2/21 | surveillance | 99 | 1563 | hCoV-19/USA/PA-VSP6013/2021 | OK247340 |
| VSP6015 | B.1.617.2 | 9/2/21 | accine breakthroug | 98.2 | 433 | hCoV-19/USA/PA-VSP6015/2021 | OK247341 |
| VSP6018 | AY.20 | 9/2/21 | surveillance | 98.1 | 238 | hCoV-19/USA/PA-VSP6018/2021 | OK247342 |
| VSP6022 | B.1.617.2 | 9/2/21 | surveillance | 98.1 | 203 | hCoV-19/USA/PA-VSP6022/2021 | OK247343 |
| VSP6023 | B.1.617.2 | 9/2/21 | surveillance | 98.6 | 553 | hCoV-19/USA/PA-VSP6023/2021 | OK247344 |
| VSP6027 | AY.25 | 9/2/21 | surveillance | 99.7 | 2976 | hCoV-19/USA/PA-VSP6027/2021 | OK247345 |
| VSP6033 | AY.24 | 9/2/21 | surveillance | 99 | 1458 | hCoV-19/USA/PA-VSP6033/2021 | OK247346 |
| VSP6034 | B.1.617.2 | 9/2/21 | surveillance | 98.8 | 1304 | hCoV-19/USA/NJ-VSP6034/2021 | OK247347 |

| | | | | | | | |
|---|---|---|---|---|---|---|---|
| VSP6035 | B.1.617.2 | 9/2/21 | surveillance | 97.7 | 250 | hCoV-19/USA/NJ-VSP6035/2021 | OK247348 |
| VSP6038 | AY.25 | 9/2/21 | surveillance | 98.7 | 1006 | hCoV-19/USA/PA-VSP6038/2021 | OK247349 |
| VSP6039 | B.1.617.2 | 9/3/21 | surveillance | 99 | 606 | hCoV-19/USA/PA-VSP6039/2021 | OK247350 |
| VSP6040 | B.1.617.2 | 9/3/21 | surveillance | 97.6 | 446 | hCoV-19/USA/PA-VSP6040/2021 | OK247351 |
| VSP6042 | B.1.617.2 | 9/3/21 | accine breakthroug | 97.5 | 160 | hCoV-19/USA/PA-VSP6042/2021 | OK247352 |
| VSP6043 | AY.3 | 9/3/21 | surveillance | 98.6 | 825 | hCoV-19/USA/PA-VSP6043/2021 | OK247353 |
| VSP6045 | B.1.617.2 | 9/3/21 | surveillance | 99.7 | 11265 | hCoV-19/USA/PA-VSP6045/2021 | OK247354 |
| VSP6047 | B.1.617.2 | 9/3/21 | surveillance | 99.7 | 12390 | hCoV-19/USA/NJ-VSP6047/2021 | OK247355 |
| VSP6049 | B.1.617.2 | 9/3/21 | surveillance | 98.7 | 550 | hCoV-19/USA/PA-VSP6049/2021 | OK247356 |
| VSP6050 | AY.24 | 9/3/21 | surveillance | 99.4 | 1550 | hCoV-19/USA/PA-VSP6050/2021 | OK247357 |
| VSP6051 | AY.24 | 9/3/21 | surveillance | 99.7 | 19048 | hCoV-19/USA/PA-VSP6051/2021 | OK247358 |
| VSP6053 | B.1.617.2 | 9/3/21 | surveillance | 98.9 | 1832 | hCoV-19/USA/PA-VSP6053/2021 | OK247359 |
| VSP6054 | B.1.617.2 | 9/3/21 | surveillance | 98.8 | 569 | hCoV-19/USA/PA-VSP6054/2021 | OK247360 |
| VSP6056 | B.1.617.2 | 9/3/21 | surveillance | 98.9 | 772 | hCoV-19/USA/NJ-VSP6056/2021 | OK247361 |
| VSP6057 | B.1.617.2 | 9/4/21 | surveillance | 98.6 | 219 | hCoV-19/USA/NJ-VSP6057/2021 | OK247362 |
| VSP6058 | B.1.617.2 | 9/4/21 | surveillance | 98.8 | 616 | hCoV-19/USA/PA-VSP6058/2021 | OK247363 |
| VSP6061 | B.1.617.2 | 9/4/21 | surveillance | 97.8 | 237 | hCoV-19/USA/PA-VSP6061/2021 | OK247364 |
| VSP6063 | B.1.617.2 | 9/4/21 | surveillance | 99.5 | 1802 | hCoV-19/USA/PA-VSP6063/2021 | OK247365 |
| VSP6064 | AY.24 | 9/4/21 | surveillance | 99.7 | 6099 | hCoV-19/USA/PA-VSP6064/2021 | OK247366 |
| VSP6067 | AY.3 | 9/4/21 | surveillance | 99.3 | 2412 | hCoV-19/USA/PA-VSP6067/2021 | OK247367 |
| VSP6068 | AY.24 | 9/4/21 | surveillance | 98.9 | 1611 | hCoV-19/USA/PA-VSP6068/2021 | OK247368 |
| VSP6075 | B.1.617.2 | 9/4/21 | surveillance | 99.2 | 2177 | hCoV-19/USA/PA-VSP6075/2021 | OK247369 |
| VSP6108 | AY.19 | 8/21/21 | surveillance | 99.5 | 1090 | hCoV-19/USA/PA-VSP6108/2021 | OK247370 |
| VSP6112 | AY.25 | 8/21/21 | surveillance | 98.8 | 553 | hCoV-19/USA/PA-VSP6112/2021 | OK247371 |
| VSP6114 | B.1.617.2 | 8/22/21 | surveillance | 98.7 | 282 | hCoV-19/USA/PA-VSP6114/2021 | OK247372 |
| VSP6115 | B.1.617.2 | 8/22/21 | surveillance | 99 | 1424 | hCoV-19/USA/PA-VSP6115/2021 | OK247373 |
| VSP6118 | AY.19 | 8/24/21 | surveillance | 98.6 | 408 | hCoV-19/USA/PA-VSP6118/2021 | OK247374 |
| VSP6122 | B.1.617.2 | 8/25/21 | surveillance | 99 | 701 | hCoV-19/USA/PA-VSP6122/2021 | |
| VSP6132 | B.1.617.2 | 9/2/21 | surveillance | 99.6 | 878 | hCoV-19/USA/PA-VSP6132/2021 | OK247375 |
| VSP6133 | B.1.617.2 | 9/2/21 | surveillance | 99.7 | 2171 | hCoV-19/USA/PA-VSP6133/2021 | OK247376 |
| VSP6136 | AY.3 | 8/23/21 | surveillance | 98.4 | 500 | hCoV-19/USA/PA-VSP6136/2021 | OK247377 |
| VSP6145 | AY.25 | 8/31/21 | surveillance | 99.3 | 6995 | hCoV-19/USA/PA-VSP6145/2021 | OK247378 |
| VSP6149 | AY.25 | 9/1/21 | surveillance | 99.6 | 1613 | hCoV-19/USA/PA-VSP6149/2021 | OK247379 |
| VSP6152 | B.1.617.2 | 8/29/21 | surveillance | 98.7 | 981 | hCoV-19/USA/PA-VSP6152/2021 | OK247380 |
| VSP8001 | B.1.409 | 2/21/21 | surveillance | 99.8 | 148 | hCoV-19/USA/S25-ILLM/2021 | |
| VSP8002 | B.1.526 | 2/21/21 | surveillance | 99.8 | 666 | hCoV-19/USA/S26-ILLM/2021 | |
| VSP8003 | B.1.637 | 2/26/21 | surveillance | 99.8 | 10156 | hCoV-19/USA/S1-ILLM/2021 | |
| VSP8004 | B.1.596 | 2/26/21 | surveillance | 99.8 | 95 | hCoV-19/USA/S2-ILLM/2021 | |
| VSP8005 | B.1.234 | 2/26/21 | surveillance | 99.8 | 10992 | hCoV-19/USA/S3-ILLM/2021 | |
| VSP8006 | B.1.637 | 2/25/21 | surveillance | 99.8 | 2398 | hCoV-19/USA/S4-ILLM/2021 | |
| VSP8007 | B.1.526 | 2/26/21 | surveillance | 99.8 | 233 | hCoV-19/USA/S5-ILLM/2021 | |
| VSP8008 | B.1.2 | 2/26/21 | surveillance | 99.8 | 168 | hCoV-19/USA/S6-ILLM/2021 | |
| VSP8009 | B.1.526 | 2/27/21 | surveillance | 99.8 | 1121 | hCoV-19/USA/S7-ILLM/2021 | |
| VSP8010 | R.1 | 2/26/21 | surveillance | 99.8 | 3793 | hCoV-19/USA/S8-ILLM/2021 | |
| VSP8011 | B.1.575 | 2/26/21 | surveillance | 99.8 | 233 | hCoV-19/USA/S9-ILLM/2021 | |
| VSP8012 | B.1.1.7 | 2/26/21 | surveillance | 99.8 | 12488 | hCoV-19/USA/S10-ILLM/2021 | |
| VSP8013 | B.1.1.519 | 2/27/21 | surveillance | 99.9 | 3592 | hCoV-19/USA/S11-ILLM/2021 | |

| | | | | | | |
|---|---|---|---|---|---|---|
| VSP8014 | B.1.243 | 2/27/21 | surveillance | 99.8 | 8862 | hCoV-19/USA/S12-ILLM/2021 |
| VSP8015 | B.1.243 | 2/27/21 | surveillance | 99.8 | 2523 | hCoV-19/USA/S13-ILLM/2021 |
| VSP8016 | B.1.595 | 3/1/21 | surveillance | 99.8 | 5257 | hCoV-19/USA/S14-ILLM/2021 |
| VSP8017 | B.1.1.7 | 3/1/21 | surveillance | 99.8 | 1083 | hCoV-19/USA/S15-ILLM/2021 |
| VSP8018 | B.1.1.7 | 3/1/21 | surveillance | 99.8 | 5087 | hCoV-19/USA/S16-ILLM/2021 |
| VSP8019 | B.1.1.7 | 3/1/21 | surveillance | 99.8 | 3108 | hCoV-19/USA/S17-ILLM/2021 |
| VSP8020 | B.1.1.7 | 3/1/21 | surveillance | 99.8 | 81 | hCoV-19/USA/S18-ILLM/2021 |
| VSP8021 | B.1.2 | 3/1/21 | surveillance | 99.8 | 144 | hCoV-19/USA/S19-ILLM/2021 |
| VSP8022 | B.1.1.7 | 3/1/21 | surveillance | 99.8 | 1205 | hCoV-19/USA/S20-ILLM/2021 |
| VSP8023 | B.1.526 | 3/1/21 | surveillance | 99.8 | 10718 | hCoV-19/USA/S21-ILLM/2021 |
| VSP8024 | B.1.596 | 3/1/21 | surveillance | 99.8 | 225 | hCoV-19/USA/S22-ILLM/2021 |
| VSP8025 | B.1.2 | 3/1/21 | surveillance | 99.4 | 25 | hCoV-19/USA/S23-ILLM/2021 |
| VSP8026 | B.1.526 | 3/1/21 | surveillance | 99.8 | 4109 | hCoV-19/USA/S24-ILLM/2021 |
| VSP8027 | B.1.243 | 1/31/21 | surveillance | 99.8 | 3144 | hCoV-19/USA/NJ-S27-ILLM/2021 |
| VSP8028 | B.1.243 | 1/31/21 | surveillance | 99.8 | 2621 | hCoV-19/USA/NJ-S28-ILLM/2021 |
| VSP8029 | B.1.526 | 2/2/21 | surveillance | 99.9 | 9926 | hCoV-19/USA/NJ-S29-ILLM/2021 |
| VSP8030 | B.1.1.7 | 2/3/21 | surveillance | 99.8 | 394 | hCoV-19/USA/NJ-S30-ILLM/2021 |
| VSP8031 | B.1.575 | 2/5/21 | surveillance | 99.8 | 915 | hCoV-19/USA/NJ-S31-ILLM/2021 |
| VSP8032 | B.1.575 | 2/6/21 | surveillance | 98.6 | 17 | hCoV-19/USA/NJ-S32-ILLM/2021 |
| VSP8033 | B.1.526 | 2/8/21 | surveillance | 99.9 | 12517 | hCoV-19/USA/NJ-S33-ILLM/2021 |
| VSP8034 | B.1.1.7 | 2/10/21 | surveillance | 99.8 | 8962 | hCoV-19/USA/NJ-S34-ILLM/2021 |
| VSP8035 | B.1.526 | 2/10/21 | surveillance | 99.8 | 5638 | hCoV-19/USA/NJ-S35-ILLM/2021 |
| VSP8036 | B.1.575 | 2/10/21 | surveillance | 99.8 | 7241 | hCoV-19/USA/NJ-S36-ILLM/2021 |
| VSP8037 | B.1.427 | 2/10/21 | surveillance | 99.8 | 3753 | hCoV-19/USA/NJ-S37-ILLM/2021 |
| VSP8038 | B.1.2 | 2/10/21 | surveillance | 99.8 | 4089 | hCoV-19/USA/NJ-S38-ILLM/2021 |
| VSP8039 | B.1.243 | 2/10/21 | surveillance | 99.8 | 13373 | hCoV-19/USA/NJ-S39-ILLM/2021 |
| VSP8040 | B.1.575 | 2/12/21 | surveillance | 99.8 | 2493 | hCoV-19/USA/NJ-S40-ILLM/2021 |
| VSP8041 | B.1.243 | 2/13/21 | surveillance | 99.8 | 3293 | hCoV-19/USA/NJ-S41-ILLM/2021 |
| VSP8042 | B.1.595 | 2/13/21 | surveillance | 99.8 | 1209 | hCoV-19/USA/NJ-S42-ILLM/2021 |
| VSP8043 | B.1.1 | 2/16/21 | surveillance | 99.8 | 4770 | hCoV-19/USA/NJ-S43-ILLM/2021 |
| VSP8044 | B.1.1.7 | 2/19/21 | surveillance | 99.8 | 548 | hCoV-19/USA/NJ-S44-ILLM/2021 |
| VSP8045 | B.1.234 | 2/19/21 | surveillance | 99.8 | 1108 | hCoV-19/USA/NJ-S45-ILLM/2021 |
| VSP8046 | B.1.234 | 2/23/21 | surveillance | 99.8 | 8211 | hCoV-19/USA/NJ-S46-ILLM/2021 |
| VSP8047 | B.1.1.7 | 3/3/21 | surveillance | 99.8 | 369 | hCoV-19/USA/NJ-S47-ILLM/2021 |
| VSP8048 | B.1.1.7 | 3/3/21 | surveillance | 99.9 | 10880 | hCoV-19/USA/NJ-S48-ILLM/2021 |
| VSP8049 | R.1 | 3/3/21 | surveillance | 97.6 | 14 | hCoV-19/USA/NJ-S49-ILLM/2021 |
| VSP8050 | B.1.575 | 3/4/21 | surveillance | 99.8 | 4329 | hCoV-19/USA/NJ-S50-ILLM/2021 |
| VSP8065 | B.1.1.7 | 4/27/21 | surveillance | 99.7 | 2480 | hCoV-19/USA/PA-Jefferson_20210601_COVIDSeq2_S15/2021 |
| VSP8066 | B.1.1.7 | 4/27/21 | surveillance | 99.6 | 2467 | hCoV-19/USA/PA-Jefferson_20210601_COVIDSeq2_S16/2021 |
| VSP8067 | B.1.1.7 | 4/27/21 | surveillance | 99.7 | 2903 | hCoV-19/USA/PA-Jefferson_20210601_COVIDSeq2_S17/2021 |
| VSP8073 | P.1.2 | 4/28/21 | surveillance | 99.6 | 2484 | hCoV-19/USA/PA-Jefferson_20210601_COVIDSeq2_S23/2021 |
| VSP8074 | B.1.1.7 | 4/28/21 | surveillance | 99.7 | 2899 | hCoV-19/USA/PA-Jefferson_20210601_COVIDSeq2_S24/2021 |
| VSP8075 | B.1.1.7 | 4/21/21 | accine breakthroug | 99.7 | 2854 | hCoV-19/USA/PA-Jefferson_20210601_COVIDSeq2_S25/2021 |
| VSP8077 | B.1.1.7 | 4/12/21 | accine breakthroug | 99.7 | 2377 | hCoV-19/USA/PA-Jefferson_20210601_COVIDSeq2_S28/2021 |
| VSP8078 | B.1.1.7 | 4/13/21 | accine breakthroug | 99.7 | 2319 | hCoV-19/USA/PA-Jefferson_20210601_COVIDSeq2_S29/2021 |
| VSP8080 | B.1.526 | 4/19/21 | accine breakthroug | 99.7 | 2717 | hCoV-19/USA/PA-Jefferson_20210601_COVIDSeq2_S31/2021 |
| VSP8081 | B.1.1.7 | 4/19/21 | accine breakthroug | 99.7 | 2826 | hCoV-19/USA/PA-Jefferson_20210601_COVIDSeq2_S32/2021 |

| | | | | | | |
|---|---|---|---|---|---|---|
| VSP8082 | B.1.1.7 | 4/21/21 | accine breakthroug | 99.7 | 2386 | hCoV-19/USA/PA-Jefferson_20210601_COVIDSeq2_S33/2021 |
| VSP8083 | B.1.1.7 | 4/23/21 | accine breakthroug | 99.7 | 2432 | hCoV-19/USA/PA-Jefferson_20210601_COVIDSeq2_S34/2021 |
| VSP8084 | B.1.1.7 | 4/23/21 | accine breakthroug | 99.7 | 2520 | hCoV-19/USA/PA-Jefferson_20210601_COVIDSeq2_S35/2021 |
| VSP8087 | B.1.526 | 5/6/21 | accine breakthroug | 98.6 | 1600 | hCoV-19/USA/PA-Jefferson_20210601_COVIDSeq2_S39/2021 |
| VSP8088 | B.1.1.7 | 5/7/21 | accine breakthroug | 99.2 | 2522 | hCoV-19/USA/PA-Jefferson_20210601_COVIDSeq2_S40/2021 |
| VSP8089 | B.1.1.7 | 5/7/21 | accine breakthroug | 99.7 | 3005 | hCoV-19/USA/PA-Jefferson_20210601_COVIDSeq2_S41/2021 |
| VSP8092 | B.1.1.7 | 5/10/21 | accine breakthroug | 99.4 | 2746 | hCoV-19/USA/PA-Jefferson_20210601_COVIDSeq2_S44/2021 |
| VSP8094 | B.1.1.7 | 5/13/21 | accine breakthroug | 99.7 | 2549 | hCoV-19/USA/PA-Jefferson_20210601_COVIDSeq2_S46/2021 |
| VSP8095 | B.1.1.7 | 4/7/21 | surveillance | 99.7 | 2575 | hCoV-19/USA/PA-Jefferson_20210605_COVIDSeq3_S01/2021 |
| VSP8096 | B.1.1.7 | 4/7/21 | surveillance | 99.7 | 2204 | hCoV-19/USA/PA-Jefferson_20210605_COVIDSeq3_S02/2021 |
| VSP8097 | B.1.1.7 | 4/7/21 | surveillance | 99.7 | 2046 | hCoV-19/USA/PA-Jefferson_20210605_COVIDSeq3_S03/2021 |
| VSP8098 | B.1.575 | 4/7/21 | surveillance | 99.6 | 2684 | hCoV-19/USA/PA-Jefferson_20210605_COVIDSeq3_S04/2021 |
| VSP8099 | B.1.1.7 | 4/7/21 | surveillance | 99.8 | 2567 | hCoV-19/USA/PA-Jefferson_20210605_COVIDSeq3_S05/2021 |
| VSP8100 | B.1.1.7 | 4/9/21 | surveillance | 99.7 | 2704 | hCoV-19/USA/PA-Jefferson_20210605_COVIDSeq3_S06/2021 |
| VSP8101 | B.1.1.7 | 4/9/21 | surveillance | 99.7 | 2898 | hCoV-19/USA/PA-Jefferson_20210605_COVIDSeq3_S07/2021 |
| VSP8102 | B.1.1.7 | 4/10/21 | surveillance | 99.7 | 2213 | hCoV-19/USA/PA-Jefferson_20210605_COVIDSeq3_S08/2021 |
| VSP8103 | B.1.1.7 | 4/10/21 | surveillance | 99.7 | 2988 | hCoV-19/USA/PA-Jefferson_20210605_COVIDSeq3_S09/2021 |
| VSP8105 | B.1.1.7 | 4/10/21 | surveillance | 99.7 | 2475 | hCoV-19/USA/PA-Jefferson_20210605_COVIDSeq3_S11/2021 |
| VSP8106 | B.1.1.7 | 4/12/21 | surveillance | 99.7 | 2416 | hCoV-19/USA/PA-Jefferson_20210605_COVIDSeq3_S12/2021 |
| VSP8107 | B.1.526 | 4/13/21 | surveillance | 99.7 | 2743 | hCoV-19/USA/PA-Jefferson_20210605_COVIDSeq3_S13/2021 |
| VSP8108 | B.1.1.7 | 4/13/21 | surveillance | 99.7 | 2397 | hCoV-19/USA/PA-Jefferson_20210605_COVIDSeq3_S14/2021 |
| VSP8109 | B.1.1.7 | 4/13/21 | surveillance | 99.7 | 2683 | hCoV-19/USA/PA-Jefferson_20210605_COVIDSeq3_S15/2021 |
| VSP8110 | Q.1 | 4/15/21 | surveillance | 99.4 | 1605 | hCoV-19/USA/PA-Jefferson_20210605_COVIDSeq3_S16/2021 |
| VSP8111 | B.1.1.7 | 4/14/21 | surveillance | 99.7 | 2421 | hCoV-19/USA/PA-Jefferson_20210605_COVIDSeq3_S17/2021 |
| VSP8112 | B.1.1.7 | 4/14/21 | surveillance | 99.7 | 2068 | hCoV-19/USA/PA-Jefferson_20210605_COVIDSeq3_S18/2021 |
| VSP8113 | B.1.526 | 4/13/21 | surveillance | 99.7 | 2810 | hCoV-19/USA/PA-Jefferson_20210605_COVIDSeq3_S19/2021 |
| VSP8114 | B.1.1.7 | 4/15/21 | surveillance | 99.7 | 2124 | hCoV-19/USA/PA-Jefferson_20210605_COVIDSeq3_S20/2021 |
| VSP8115 | B.1.1.7 | 4/15/21 | surveillance | 99.8 | 2762 | hCoV-19/USA/PA-Jefferson_20210605_COVIDSeq3_S21/2021 |
| VSP8116 | B.1.1.7 | 4/15/21 | surveillance | 99.7 | 2461 | hCoV-19/USA/PA-Jefferson_20210605_COVIDSeq3_S22/2021 |
| VSP8117 | B.1.1.7 | 4/15/21 | surveillance | 99.7 | 2461 | hCoV-19/USA/PA-Jefferson_20210605_COVIDSeq3_S23/2021 |
| VSP8118 | B.1.1.7 | 4/16/21 | surveillance | 99.8 | 2604 | hCoV-19/USA/PA-Jefferson_20210605_COVIDSeq3_S24/2021 |
| VSP8119 | B.1.526 | 4/16/21 | surveillance | 99.6 | 2735 | hCoV-19/USA/PA-Jefferson_20210605_COVIDSeq3_S25/2021 |
| VSP8120 | B.1.1.7 | 4/15/21 | surveillance | 99.7 | 2528 | hCoV-19/USA/PA-Jefferson_20210605_COVIDSeq3_S26/2021 |
| VSP8121 | B.1.1.7 | 4/15/21 | surveillance | 99.7 | 2476 | hCoV-19/USA/PA-Jefferson_20210605_COVIDSeq3_S27/2021 |
| VSP8122 | B.1.1.7 | 4/17/21 | surveillance | 99.7 | 2767 | hCoV-19/USA/PA-Jefferson_20210605_COVIDSeq3_S28/2021 |
| VSP8123 | B.1.1.7 | 4/17/21 | surveillance | 99.7 | 2845 | hCoV-19/USA/PA-Jefferson_20210605_COVIDSeq3_S29/2021 |
| VSP8124 | B.1.1.7 | 4/17/21 | surveillance | 99.7 | 2426 | hCoV-19/USA/PA-Jefferson_20210605_COVIDSeq3_S30/2021 |
| VSP8125 | B.1.1.7 | 4/17/21 | surveillance | 99.7 | 2452 | hCoV-19/USA/PA-Jefferson_20210605_COVIDSeq3_S31/2021 |
| VSP8126 | B.1.1.7 | 4/16/21 | surveillance | 99.7 | 2319 | hCoV-19/USA/PA-Jefferson_20210605_COVIDSeq3_S32/2021 |
| VSP8127 | B.1.1.7 | 4/17/21 | surveillance | 99.7 | 1664 | hCoV-19/USA/PA-Jefferson_20210605_COVIDSeq3_S33/2021 |
| VSP8128 | B.1.1.7 | 4/17/21 | surveillance | 99.1 | 1735 | hCoV-19/USA/PA-Jefferson_20210605_COVIDSeq3_S34/2021 |
| VSP8129 | B.1.1.7 | 4/18/21 | surveillance | 99.7 | 2216 | hCoV-19/USA/PA-Jefferson_20210605_COVIDSeq3_S35/2021 |
| VSP8130 | B.1.1.7 | 4/17/21 | surveillance | 99.8 | 2797 | hCoV-19/USA/PA-Jefferson_20210605_COVIDSeq3_S36/2021 |
| VSP8131 | B.1.1.7 | 4/17/21 | surveillance | 99.8 | 2837 | hCoV-19/USA/PA-Jefferson_20210605_COVIDSeq3_S37/2021 |
| VSP8132 | B.1.351 | 4/17/21 | surveillance | 99.5 | 3132 | hCoV-19/USA/PA-Jefferson_20210605_COVIDSeq3_S38/2021 |
| VSP8133 | B.1.575 | 4/18/21 | surveillance | 99.7 | 2533 | hCoV-19/USA/PA-Jefferson_20210605_COVIDSeq3_S39/2021 |
| VSP8134 | B.1.637 | 4/18/21 | surveillance | 99.7 | 2431 | hCoV-19/USA/PA-Jefferson_20210605_COVIDSeq3_S40/2021 |

| | | | | | | |
|---|---|---|---|---|---|---|
| VSP8135 | B.1.1.7 | 4/19/21 | surveillance | 99.7 | 2942 | hCoV-19/USA/PA-Jefferson_20210605_COVIDSeq3_S41/2021 |
| VSP8136 | B.1.1.7 | 4/19/21 | surveillance | 99.7 | 2567 | hCoV-19/USA/PA-Jefferson_20210605_COVIDSeq3_S42/2021 |
| VSP8137 | Q.6 | 4/19/21 | surveillance | 99.7 | 2562 | hCoV-19/USA/PA-Jefferson_20210605_COVIDSeq3_S43/2021 |
| VSP8138 | B.1.1.7 | 4/19/21 | surveillance | 99.7 | 2721 | hCoV-19/USA/PA-Jefferson_20210605_COVIDSeq3_S44/2021 |
| VSP8139 | B.1.526 | 4/20/21 | surveillance | 99.7 | 2755 | hCoV-19/USA/PA-Jefferson_20210605_COVIDSeq3_S45/2021 |
| VSP8140 | B.1.526 | 4/20/21 | surveillance | 99.7 | 3437 | hCoV-19/USA/PA-Jefferson_20210605_COVIDSeq3_S46/2021 |
| VSP8141 | B.1.1.7 | 5/9/21 | accine breakthroug | 99.7 | 2418 | hCoV-19/USA/PA-Jefferson_20210614_COVIDSeq10_S01/2021 |
| VSP8142 | B.1.1.7 | 5/16/21 | accine breakthroug | 99.7 | 2295 | hCoV-19/USA/PA-Jefferson_20210614_COVIDSeq10_S02/2021 |
| VSP8143 | B.1.617.2 | 4/29/21 | accine breakthroug | 99.7 | 2605 | hCoV-19/USA/PA-Jefferson_20210614_COVIDSeq10_S03/2021 |
| VSP8144 | B.1.1.7 | 5/7/21 | accine breakthroug | 99.7 | 2703 | hCoV-19/USA/PA-Jefferson_20210614_COVIDSeq10_S04/2021 |
| VSP8147 | B.1.1.7 | 4/20/21 | surveillance | 99.6 | 2103 | hCoV-19/USA/PA-Jefferson_20210614_COVIDSeq10_S07/2021 |
| VSP8148 | B.1.1.7 | 4/20/21 | surveillance | 99.7 | 1876 | hCoV-19/USA/PA-Jefferson_20210614_COVIDSeq10_S08/2021 |
| VSP8149 | B.1.1.7 | 4/20/21 | surveillance | 99.2 | 1450 | hCoV-19/USA/PA-Jefferson_20210614_COVIDSeq10_S09/2021 |
| VSP8150 | B.1.1.7 | 4/21/21 | surveillance | 99.7 | 2885 | hCoV-19/USA/PA-Jefferson_20210614_COVIDSeq10_S10/2021 |
| VSP8151 | B.1.1.7 | 4/21/21 | surveillance | 99.7 | 1559 | hCoV-19/USA/PA-Jefferson_20210614_COVIDSeq10_S11/2021 |
| VSP8152 | B.1.1.7 | 4/21/21 | surveillance | 99.7 | 2194 | hCoV-19/USA/PA-Jefferson_20210614_COVIDSeq10_S12/2021 |
| VSP8153 | B.1.1.7 | 4/21/21 | surveillance | 99.3 | 2455 | hCoV-19/USA/PA-Jefferson_20210614_COVIDSeq10_S13/2021 |
| VSP8154 | B.1.1.7 | 4/21/21 | surveillance | 99.7 | 2125 | hCoV-19/USA/PA-Jefferson_20210614_COVIDSeq10_S14/2021 |
| VSP8155 | B.1.1.7 | 4/22/21 | surveillance | 99 | 2344 | hCoV-19/USA/PA-Jefferson_20210614_COVIDSeq10_S15/2021 |
| VSP8156 | B.1.1.7 | 4/22/21 | surveillance | 99.7 | 1421 | hCoV-19/USA/PA-Jefferson_20210614_COVIDSeq10_S16/2021 |
| VSP8158 | B.1.1.519 | 4/21/21 | surveillance | 98.6 | 1159 | hCoV-19/USA/PA-Jefferson_20210614_COVIDSeq10_S18/2021 |
| VSP8159 | B.1.1.7 | 4/24/21 | surveillance | 99.2 | 1555 | hCoV-19/USA/PA-Jefferson_20210614_COVIDSeq10_S19/2021 |
| VSP8160 | B.1.526 | 4/23/21 | surveillance | 99.7 | 2588 | hCoV-19/USA/PA-Jefferson_20210614_COVIDSeq10_S20/2021 |
| VSP8161 | B.1.1.7 | 4/23/21 | surveillance | 99.1 | 1500 | hCoV-19/USA/PA-Jefferson_20210614_COVIDSeq10_S21/2021 |
| VSP8162 | B.1.526 | 4/24/21 | surveillance | 99.7 | 2712 | hCoV-19/USA/PA-Jefferson_20210614_COVIDSeq10_S22/2021 |
| VSP8163 | B.1.1.7 | 4/25/21 | surveillance | 99.7 | 2575 | hCoV-19/USA/PA-Jefferson_20210614_COVIDSeq10_S23/2021 |
| VSP8164 | B.1.1.7 | 4/25/21 | surveillance | 99.7 | 2568 | hCoV-19/USA/PA-Jefferson_20210614_COVIDSeq10_S24/2021 |
| VSP8165 | B.1.1.7 | 4/25/21 | surveillance | 99.7 | 2661 | hCoV-19/USA/PA-Jefferson_20210614_COVIDSeq10_S25/2021 |
| VSP8166 | B.1.1.7 | 4/26/21 | surveillance | 99.7 | 3096 | hCoV-19/USA/PA-Jefferson_20210614_COVIDSeq10_S26/2021 |
| VSP8167 | B.1.1.7 | 4/26/21 | surveillance | 99.7 | 2827 | hCoV-19/USA/PA-Jefferson_20210614_COVIDSeq10_S27/2021 |
| VSP8168 | B.1.1.7 | 4/26/21 | surveillance | 99.7 | 3466 | hCoV-19/USA/PA-Jefferson_20210614_COVIDSeq10_S28/2021 |
| VSP8169 | B.1.1.7 | 4/27/21 | surveillance | 99.7 | 2951 | hCoV-19/USA/PA-Jefferson_20210614_COVIDSeq10_S29/2021 |
| VSP8170 | B.1.526 | 4/27/21 | surveillance | 99.8 | 3181 | hCoV-19/USA/PA-Jefferson_20210614_COVIDSeq10_S30/2021 |
| VSP8171 | B.1.1.7 | 4/27/21 | surveillance | 99.7 | 3205 | hCoV-19/USA/PA-Jefferson_20210614_COVIDSeq10_S31/2021 |
| VSP8176 | B.1.637 | 4/29/21 | surveillance | 99.7 | 2206 | |
| VSP8177 | B.1.1.7 | 4/29/21 | surveillance | 99.7 | 2720 | hCoV-19/USA/PA-Jefferson_20210614_COVIDSeq10_S37/2021 |
| VSP8178 | B.1.526 | 4/29/21 | surveillance | 99.7 | 2894 | hCoV-19/USA/PA-Jefferson_20210614_COVIDSeq10_S38/2021 |
| VSP8179 | B.1.526 | 1/5/21 | surveillance | 99.7 | 3160 | hCoV-19/USA/PA-Jefferson_20210614_COVIDSeq10_S39/2021 |
| VSP8180 | B.1.1.7 | 4/30/21 | surveillance | 99.7 | 3156 | hCoV-19/USA/PA-Jefferson_20210614_COVIDSeq10_S40/2021 |
| VSP8181 | B.1.621 | 4/30/21 | surveillance | 99.7 | 3187 | hCoV-19/USA/PA-Jefferson_20210614_COVIDSeq10_S41/2021 |
| VSP8185 | B.1.1.7 | 5/1/21 | surveillance | 99.7 | 2288 | hCoV-19/USA/PA-Jefferson_20210614_COVIDSeq10_S45/2021 |
| VSP8186 | B.1.1.7 | 5/1/21 | surveillance | 99.7 | 2872 | hCoV-19/USA/PA-Jefferson_20210614_COVIDSeq10_S46/2021 |
| VSP8187 | B.1.1.7 | 5/6/21 | surveillance | 99.8 | 2295 | hCoV-19/USA/PA-Jefferson_20210621_COVIDSeq11_S01/2021 |
| VSP8188 | B.1.1.7 | 5/6/21 | surveillance | 99.3 | 2194 | hCoV-19/USA/PA-Jefferson_20210621_COVIDSeq11_S02/2021 |
| VSP8189 | B.1.1.7 | 5/6/21 | surveillance | 99.2 | 1246 | hCoV-19/USA/PA-Jefferson_20210621_COVIDSeq11_S03/2021 |
| VSP8190 | B.1.1.7 | 5/6/21 | surveillance | 99.7 | 2136 | hCoV-19/USA/PA-Jefferson_20210621_COVIDSeq11_S04/2021 |
| VSP8191 | B.1.1.7 | 5/7/21 | surveillance | 99.7 | 2267 | hCoV-19/USA/PA-Jefferson_20210621_COVIDSeq11_S05/2021 |

| | | | | | | |
|---|---|---|---|---|---|---|
| VSP8192 | B.1.1.7 | 5/8/21 | surveillance | 99.8 | 2655 | hCoV-19/USA/PA-Jefferson_20210621_COVIDSeq11_S06/2021 |
| VSP8193 | B.1.1.7 | 5/8/21 | surveillance | 99.7 | 1908 | hCoV-19/USA/PA-Jefferson_20210621_COVIDSeq11_S07/2021 |
| VSP8194 | B.1.1.7 | 5/9/21 | surveillance | 99.7 | 1692 | hCoV-19/USA/PA-Jefferson_20210621_COVIDSeq11_S08/2021 |
| VSP8195 | B.1.1.7 | 5/10/21 | surveillance | 99.7 | 2358 | hCoV-19/USA/PA-Jefferson_20210621_COVIDSeq11_S09/2021 |
| VSP8196 | B.1.1.7 | 5/10/21 | surveillance | 99.7 | 1976 | hCoV-19/USA/PA-Jefferson_20210621_COVIDSeq11_S10/2021 |
| VSP8197 | B.1.1.7 | 5/10/21 | surveillance | 99.7 | 2383 | hCoV-19/USA/PA-Jefferson_20210621_COVIDSeq11_S11/2021 |
| VSP8198 | B.1.526 | 5/10/21 | surveillance | 99.7 | 1666 | hCoV-19/USA/PA-Jefferson_20210621_COVIDSeq11_S12/2021 |
| VSP8199 | B.1.1.7 | 5/10/21 | surveillance | 99.7 | 1886 | hCoV-19/USA/PA-Jefferson_20210621_COVIDSeq11_S13/2021 |
| VSP8200 | B.1.621 | 5/12/21 | surveillance | 99.7 | 2776 | hCoV-19/USA/PA-Jefferson_20210621_COVIDSeq11_S14/2021 |
| VSP8201 | B.1.637 | 5/11/21 | surveillance | 99.7 | 2742 | hCoV-19/USA/PA-Jefferson_20210621_COVIDSeq11_S15/2021 |
| VSP8202 | B.1.1.7 | 5/11/21 | surveillance | 99.7 | 2188 | hCoV-19/USA/PA-Jefferson_20210621_COVIDSeq11_S16/2021 |
| VSP8203 | B.1.1.7 | 5/12/21 | surveillance | 99.7 | 1841 | hCoV-19/USA/PA-Jefferson_20210621_COVIDSeq11_S17/2021 |
| VSP8204 | B.1.1.7 | 5/13/21 | surveillance | 99.7 | 2617 | hCoV-19/USA/PA-Jefferson_20210621_COVIDSeq11_S18/2021 |
| VSP8205 | B.1.1.7 | 5/13/21 | surveillance | 99.7 | 2230 | hCoV-19/USA/PA-Jefferson_20210621_COVIDSeq11_S19/2021 |
| VSP8206 | B.1.1.7 | 5/13/21 | surveillance | 99.7 | 1730 | hCoV-19/USA/PA-Jefferson_20210621_COVIDSeq11_S20/2021 |
| VSP8207 | B.1.1.7 | 5/13/21 | surveillance | 99.7 | 2141 | hCoV-19/USA/PA-Jefferson_20210621_COVIDSeq11_S21/2021 |
| VSP8208 | B.1.1.7 | 5/13/21 | surveillance | 99.7 | 2038 | hCoV-19/USA/PA-Jefferson_20210621_COVIDSeq11_S22/2021 |
| VSP8209 | B.1.1.7 | 5/2/21 | surveillance | 99.7 | 2050 | hCoV-19/USA/PA-Jefferson_20210621_COVIDSeq11_S24/2021 |
| VSP8210 | B.1.1.7 | 5/2/21 | surveillance | 99.7 | 1866 | hCoV-19/USA/PA-Jefferson_20210621_COVIDSeq11_S25/2021 |
| VSP8211 | B.1.1.7 | 5/2/21 | surveillance | 99.7 | 2651 | hCoV-19/USA/PA-Jefferson_20210621_COVIDSeq11_S26/2021 |
| VSP8212 | B.1.617.2 | 5/3/21 | surveillance | 99.7 | 2301 | hCoV-19/USA/PA-Jefferson_20210621_COVIDSeq11_S27/2021 |
| VSP8213 | B.1.1.7 | 5/3/21 | surveillance | 99.7 | 2692 | hCoV-19/USA/PA-Jefferson_20210621_COVIDSeq11_S28/2021 |
| VSP8214 | B.1.1.7 | 5/3/21 | surveillance | 99.7 | 2628 | hCoV-19/USA/PA-Jefferson_20210621_COVIDSeq11_S29/2021 |
| VSP8215 | B.1.1.7 | 5/3/21 | surveillance | 99.4 | 2705 | hCoV-19/USA/PA-Jefferson_20210621_COVIDSeq11_S30/2021 |
| VSP8216 | B.1.525 | 5/3/21 | surveillance | 97.1 | 895 | hCoV-19/USA/PA-Jefferson_20210621_COVIDSeq11_S31/2021 |
| VSP8217 | B.1.1.7 | 5/3/21 | surveillance | 99.7 | 2421 | hCoV-19/USA/PA-Jefferson_20210621_COVIDSeq11_S32/2021 |
| VSP8218 | B.1.1.7 | 5/4/21 | surveillance | 99.7 | 1628 | hCoV-19/USA/PA-Jefferson_20210621_COVIDSeq11_S33/2021 |
| VSP8219 | B.1.1.7 | 5/4/21 | surveillance | 99.7 | 2273 | hCoV-19/USA/PA-Jefferson_20210621_COVIDSeq11_S34/2021 |
| VSP8220 | B.1.1.7 | 5/4/21 | surveillance | 98.7 | 1248 | hCoV-19/USA/PA-Jefferson_20210621_COVIDSeq11_S35/2021 |
| VSP8221 | B.1.1.7 | 5/4/21 | surveillance | 99.7 | 2265 | hCoV-19/USA/PA-Jefferson_20210621_COVIDSeq11_S36/2021 |
| VSP8222 | B.1.1.7 | 5/13/21 | surveillance | 99.7 | 1946 | hCoV-19/USA/PA-Jefferson_20210621_COVIDSeq11_S37/2021 |
| VSP8223 | B.1.1.7 | 5/13/21 | surveillance | 99.7 | 2291 | hCoV-19/USA/PA-Jefferson_20210621_COVIDSeq11_S38/2021 |
| VSP8224 | B.1.1.7 | 5/15/21 | surveillance | 99.7 | 2134 | hCoV-19/USA/PA-Jefferson_20210621_COVIDSeq11_S39/2021 |
| VSP8225 | B.1.1.7 | 5/15/21 | surveillance | 99.7 | 2613 | hCoV-19/USA/PA-Jefferson_20210621_COVIDSeq11_S40/2021 |
| VSP8226 | B.1.1.7 | 5/17/21 | surveillance | 99.7 | 2172 | hCoV-19/USA/PA-Jefferson_20210621_COVIDSeq11_S41/2021 |
| VSP8227 | B.1.1.7 | 5/17/21 | surveillance | 99.7 | 2200 | hCoV-19/USA/PA-Jefferson_20210621_COVIDSeq11_S42/2021 |
| VSP8228 | B.1.526 | 5/18/21 | surveillance | 99.7 | 2432 | hCoV-19/USA/PA-Jefferson_20210621_COVIDSeq11_S43/2021 |
| VSP8229 | B.1.1.7 | 5/18/21 | surveillance | 99.7 | 2386 | hCoV-19/USA/PA-Jefferson_20210621_COVIDSeq11_S44/2021 |
| VSP8230 | B.1.1.7 | 5/19/21 | surveillance | 99.7 | 2433 | hCoV-19/USA/PA-Jefferson_20210621_COVIDSeq11_S45/2021 |
| VSP8231 | B.1.526 | 5/19/21 | surveillance | 99.7 | 2372 | hCoV-19/USA/PA-Jefferson_20210621_COVIDSeq11_S46/2021 |
| VSP8232 | B.1.526 | 5/19/21 | surveillance | 99.7 | 3061 | hCoV-19/USA/PA-Jefferson_20210627_COVIDSeq12_S01/2021 |
| VSP8233 | B.1.1.7 | 5/19/21 | surveillance | 97.7 | 2059 | hCoV-19/USA/PA-Jefferson_20210627_COVIDSeq12_S02/2021 |
| VSP8234 | B.1.526 | 5/21/21 | surveillance | 99.5 | 1860 | hCoV-19/USA/PA-Jefferson_20210627_COVIDSeq12_S03/2021 |
| VSP8235 | B.1.1.7 | 5/21/21 | surveillance | 99.7 | 2627 | hCoV-19/USA/PA-Jefferson_20210627_COVIDSeq12_S04/2021 |
| VSP8236 | B.1.1.7 | 5/21/21 | surveillance | 99.7 | 2781 | hCoV-19/USA/PA-Jefferson_20210627_COVIDSeq12_S05/2021 |
| VSP8237 | B.1.1.7 | 5/22/21 | surveillance | 99.7 | 1428 | hCoV-19/USA/PA-Jefferson_20210627_COVIDSeq12_S06/2021 |
| VSP8238 | B.1.1.7 | 5/22/21 | surveillance | 99.5 | 1610 | hCoV-19/USA/PA-Jefferson_20210627_COVIDSeq12_S07/2021 |

| | | | | | | |
|---|---|---|---|---|---|---|
| VSP8239 | B.1.1.7 | 5/24/21 | surveillance | 99.7 | 3051 | hCoV-19/USA/PA-Jefferson_20210627_COVIDSeq12_S08/2021 |
| VSP8240 | B.1.1.7 | 5/25/21 | surveillance | 99.7 | 2763 | hCoV-19/USA/PA-Jefferson_20210627_COVIDSeq12_S09/2021 |
| VSP8241 | B.1.526 | 5/24/21 | surveillance | 99.7 | 1819 | hCoV-19/USA/PA-Jefferson_20210627_COVIDSeq12_S10/2021 |
| VSP8242 | B.1.525 | 5/26/21 | surveillance | 99.5 | 2949 | hCoV-19/USA/PA-Jefferson_20210627_COVIDSeq12_S11/2021 |
| VSP8243 | B.1.526 | 5/26/21 | surveillance | 99.7 | 2796 | hCoV-19/USA/PA-Jefferson_20210627_COVIDSeq12_S12/2021 |
| VSP8244 | B.1.1.7 | 5/26/21 | surveillance | 99.7 | 2890 | hCoV-19/USA/PA-Jefferson_20210627_COVIDSeq12_S13/2021 |
| VSP8245 | B.1.1.7 | 5/28/21 | surveillance | 99.8 | 2826 | hCoV-19/USA/PA-Jefferson_20210627_COVIDSeq12_S14/2021 |
| VSP8246 | B.1.1.7 | 5/28/21 | surveillance | 99.8 | 2696 | hCoV-19/USA/PA-Jefferson_20210627_COVIDSeq12_S15/2021 |
| VSP8247 | B.1.526 | 5/30/21 | surveillance | 99.7 | 2658 | hCoV-19/USA/PA-Jefferson_20210627_COVIDSeq12_S16/2021 |
| VSP8248 | B.1.1.7 | 5/26/21 | surveillance | 99.7 | 3293 | hCoV-19/USA/PA-Jefferson_20210627_COVIDSeq12_S17/2021 |
| VSP8249 | B.1.1.7 | 6/3/21 | surveillance | 99.7 | 2559 | hCoV-19/USA/PA-Jefferson_20210627_COVIDSeq12_S18/2021 |
| VSP8250 | P.1 | 5/25/21 | surveillance | 99.7 | 2310 | hCoV-19/USA/PA-Jefferson_20210627_COVIDSeq12_S19/2021 |
| VSP8251 | B.1.1.7 | 6/3/21 | surveillance | 99.7 | 2741 | hCoV-19/USA/PA-Jefferson_20210627_COVIDSeq12_S20/2021 |
| VSP8252 | P.1 | 6/4/21 | surveillance | 99.7 | 2651 | hCoV-19/USA/PA-Jefferson_20210627_COVIDSeq12_S21/2021 |
| VSP8253 | B.1.1.7 | 6/6/21 | surveillance | 99.7 | 2953 | hCoV-19/USA/PA-Jefferson_20210627_COVIDSeq12_S22/2021 |
| VSP8254 | B.1.1.7 | 6/5/21 | surveillance | 99.7 | 2553 | hCoV-19/USA/PA-Jefferson_20210627_COVIDSeq12_S23/2021 |
| VSP8255 | B.1.1.7 | 6/9/21 | surveillance | 99.7 | 1944 | hCoV-19/USA/PA-Jefferson_20210627_COVIDSeq12_S24/2021 |
| VSP8256 | B.1.1.7 | 6/10/21 | surveillance | 99.7 | 2806 | hCoV-19/USA/PA-Jefferson_20210627_COVIDSeq12_S25/2021 |
| VSP8257 | B.1.526 | 6/11/21 | surveillance | 99.7 | 2918 | hCoV-19/USA/PA-Jefferson_20210627_COVIDSeq12_S26/2021 |
| VSP8258 | B.1.1.7 | 6/15/21 | surveillance | 99.2 | 2083 | hCoV-19/USA/PA-Jefferson_20210627_COVIDSeq12_S27/2021 |
| VSP8259 | AY.3 | 6/23/21 | surveillance | 99.6 | 3089 | hCoV-19/USA/PA-Jefferson_20210627_COVIDSeq12_S28/2021 |
| VSP8260 | B.1.1.7 | 6/22/21 | surveillance | 99.7 | 2928 | hCOV-19/USA/PA-Jefferson_20210727_COVIDSeq13_01/2021 |
| VSP8261 | AY.25 | 6/25/21 | surveillance | 98.9 | 1335 | hCOV-19/USA/PA-Jefferson_20210727_COVIDSeq13_02/2021 |
| VSP8262 | B.1.1.7 | 6/26/21 | surveillance | 99.7 | 3020 | hCOV-19/USA/PA-Jefferson_20210727_COVIDSeq13_03/2021 |
| VSP8263 | B.1.621 | 6/28/21 | surveillance | 98.9 | 2613 | hCOV-19/USA/PA-Jefferson_20210727_COVIDSeq13_04/2021 |
| VSP8264 | B.1.617.2 | 6/28/21 | surveillance | 99.5 | 2499 | hCOV-19/USA/PA-Jefferson_20210727_COVIDSeq13_05/2021 |
| VSP8265 | AY.5 | 6/29/21 | surveillance | 96.9 | 931 | hCOV-19/USA/PA-Jefferson_20210727_COVIDSeq13_06/2021 |
| VSP8266 | B.1.621 | 7/2/21 | surveillance | 99.7 | 2613 | hCOV-19/USA/PA-Jefferson_20210727_COVIDSeq13_07/2021 |
| VSP8267 | B.1.1.7 | 7/6/21 | surveillance | 99.7 | 2736 | hCOV-19/USA/PA-Jefferson_20210727_COVIDSeq13_08/2021 |
| VSP8268 | AY.6 | 7/7/21 | surveillance | 99.1 | 2516 | hCOV-19/USA/PA-Jefferson_20210727_COVIDSeq13_09/2021 |
| VSP8269 | B.1.617.2 | 7/9/21 | surveillance | 99.4 | 1932 | hCOV-19/USA/PA-Jefferson_20210727_COVIDSeq13_10/2021 |
| VSP8270 | B.1.617.2 | 7/9/21 | surveillance | 99 | 2393 | hCOV-19/USA/PA-Jefferson_20210727_COVIDSeq13_11/2021 |
| VSP8271 | B.1.617.2 | 7/11/21 | surveillance | 98.6 | 1407 | hCOV-19/USA/PA-Jefferson_20210727_COVIDSeq13_12/2021 |
| VSP8272 | AY.12 | 7/10/21 | surveillance | 99.3 | 1943 | hCOV-19/USA/PA-Jefferson_20210727_COVIDSeq13_13/2021 |
| VSP8273 | B.1.617.2 | 7/12/21 | surveillance | 99.3 | 1190 | hCOV-19/USA/PA-Jefferson_20210727_COVIDSeq13_14/2021 |
| VSP8274 | AY.3 | 7/12/21 | surveillance | 99.1 | 3062 | hCOV-19/USA/PA-Jefferson_20210727_COVIDSeq13_15/2021 |
| VSP8275 | AY.6 | 7/13/21 | surveillance | 99.1 | 2432 | hCOV-19/USA/PA-Jefferson_20210727_COVIDSeq13_16/2021 |
| VSP8276 | AY.3 | 7/13/21 | surveillance | 98.7 | 3282 | hCOV-19/USA/PA-Jefferson_20210727_COVIDSeq13_17/2021 |
| VSP8277 | AY.25 | 7/14/21 | surveillance | 99.3 | 2750 | hCOV-19/USA/PA-Jefferson_20210727_COVIDSeq13_18/2021 |
| VSP8278 | B.1.1.7 | 7/14/21 | surveillance | 99.7 | 3217 | hCOV-19/USA/PA-Jefferson_20210727_COVIDSeq13_19/2021 |
| VSP8279 | B.1.617.2 | 7/16/21 | surveillance | 98.9 | 3235 | hCOV-19/USA/PA-Jefferson_20210727_COVIDSeq13_20/2021 |
| VSP8280 | B.1.1.7 | 7/15/21 | surveillance | 99.7 | 3716 | hCOV-19/USA/PA-Jefferson_20210727_COVIDSeq13_21/2021 |
| VSP8281 | B.1.617.2 | 7/16/21 | surveillance | 99 | 2268 | hCOV-19/USA/PA-Jefferson_20210727_COVIDSeq13_22/2021 |
| VSP8282 | B.1.617.2 | 7/19/21 | accine breakthroug | 98.7 | 2618 | hCOV-19/USA/PA-Jefferson_20210727_COVIDSeq13_23/2021 |
| VSP8284 | B.1.526 | 7/20/21 | accine breakthroug | 99.7 | 3393 | hCOV-19/USA/PA-Jefferson_20210727_COVIDSeq13_25/2021 |
| VSP8285 | AY.12 | 7/20/21 | accine breakthroug | 97.7 | 1824 | hCOV-19/USA/PA-Jefferson_20210727_COVIDSeq13_26/2021 |
| VSP8286 | AY.3 | 7/19/21 | surveillance | 98.5 | 2815 | hCOV-19/USA/PA-Jefferson_20210727_COVIDSeq13_27/2021 |

| | | | | | | |
|---|---|---|---|---|---|---|
| VSP8287 | AY.3 | 7/19/21 | accine breakthroug | 96.4 | 1898 | hCOV-19/USA/PA-Jefferson_20210727_COVIDSeq13_28/2021 |
| VSP8288 | AY.25 | 7/20/21 | surveillance | 99.1 | 2754 | hCOV-19/USA/PA-Jefferson_20210727_COVIDSeq13_29/2021 |
| VSP8289 | AY.24 | 7/19/21 | surveillance | 99.7 | 3631 | hCOV-19/USA/PA-Jefferson_20210727_COVIDSeq13_30/2021 |
| VSP8290 | AY.25 | 7/19/21 | surveillance | 99.4 | 2130 | hCOV-19/USA/PA-Jefferson_20210727_COVIDSeq13_31/2021 |
| VSP8291 | B.1.617.2 | 7/19/21 | surveillance | 99.5 | 2532 | hCOV-19/USA/PA-Jefferson_20210727_COVIDSeq13_32/2021 |
| VSP8292 | B.1.617.2 | 7/20/21 | accine breakthroug | 99.6 | 2295 | hCOV-19/USA/PA-Jefferson_20210727_COVIDSeq13_33/2021 |
| VSP8294 | B.1.617.2 | 7/21/21 | accine breakthroug | 98.7 | 1561 | hCOV-19/USA/PA-Jefferson_20210727_COVIDSeq13_35/2021 |
| VSP8295 | AY.14 | 7/21/21 | accine breakthroug | 98.4 | 2319 | hCOV-19/USA/PA-Jefferson_20210727_COVIDSeq13_36/2021 |
| VSP8296 | B.1.617.2 | 7/21/21 | accine breakthroug | 98.9 | 2799 | hCOV-19/USA/PA-Jefferson_20210727_COVIDSeq13_37/2021 |
| VSP8297 | B.1.617.2 | 7/21/21 | surveillance | 99.3 | 2515 | hCOV-19/USA/PA-Jefferson_20210727_COVIDSeq13_38/2021 |
| VSP8298 | AY.14 | 7/22/21 | surveillance | 99.5 | 2559 | hCOV-19/USA/PA-Jefferson_20210727_COVIDSeq13_39/2021 |
| VSP8299 | B.1.617.2 | 7/22/21 | surveillance | 99.7 | 3239 | hCOV-19/USA/PA-Jefferson_20210727_COVIDSeq13_40/2021 |
| VSP8301 | AY.25 | 7/23/21 | surveillance | 99.4 | 2618 | hCOV-19/USA/PA-Jefferson_20210727_COVIDSeq13_42/2021 |
| VSP8302 | AY.25 | 7/23/21 | surveillance | 98.7 | 2246 | hCOV-19/USA/PA-Jefferson_20210727_COVIDSeq13_43/2021 |
| VSP8303 | B.1.617.2 | 7/23/21 | surveillance | 99.1 | 3292 | hCOV-19/USA/PA-Jefferson_20210727_COVIDSeq13_44/2021 |
| VSP8304 | B.1.617.2 | 7/23/21 | surveillance | 99.4 | 3000 | hCOV-19/USA/PA-Jefferson_20210727_COVIDSeq13_45/2021 |
| VSP8305 | B.1.617.2 | 7/24/21 | surveillance | 99.4 | 3783 | hCOV-19/USA/PA-Jefferson_20210727_COVIDSeq13_46/2021 |
| VSP8375 | AY.12 | 7/23/21 | surveillance | 99.3 | 1819 | hCOV-19/USA/PA-Jefferson_20210803_COVIDSeq14_01/2021 |
| VSP8376 | AY.25 | 7/24/21 | surveillance | 98.8 | 1224 | hCOV-19/USA/PA-Jefferson_20210803_COVIDSeq14_02/2021 |
| VSP8377 | B.1.617.2 | 7/25/21 | surveillance | 99.7 | 1845 | hCOV-19/USA/PA-Jefferson_20210803_COVIDSeq14_03/2021 |
| VSP8378 | B.1.621 | 7/24/21 | surveillance | 99.8 | 2044 | hCOV-19/USA/PA-Jefferson_20210803_COVIDSeq14_04/2021 |
| VSP8379 | B.1.617.2 | 7/25/21 | accine breakthroug | 99.7 | 2967 | hCOV-19/USA/PA-Jefferson_20210803_COVIDSeq14_05/2021 |
| VSP8380 | B.1.617.2 | 7/26/21 | accine breakthroug | 99.7 | 990 | hCOV-19/USA/PA-Jefferson_20210803_COVIDSeq14_06/2021 |
| VSP8381 | B.1.617.2 | 7/26/21 | accine breakthroug | 99.7 | 1757 | hCOV-19/USA/PA-Jefferson_20210803_COVIDSeq14_07/2021 |
| VSP8382 | B.1.617.2 | 7/26/21 | surveillance | 99.7 | 1445 | hCOV-19/USA/PA-Jefferson_20210803_COVIDSeq14_08/2021 |
| VSP8383 | AY.25 | 7/26/21 | surveillance | 96.6 | 1015 | hCOV-19/USA/PA-Jefferson_20210803_COVIDSeq14_09/2021 |
| VSP8384 | B.1.617.2 | 7/27/21 | surveillance | 99.7 | 2493 | hCOV-19/USA/PA-Jefferson_20210803_COVIDSeq14_10/2021 |
| VSP8385 | B.1.617.2 | 7/26/21 | surveillance | 99.7 | 2458 | hCOV-19/USA/PA-Jefferson_20210803_COVIDSeq14_11/2021 |
| VSP8388 | B.1.617.2 | 7/27/21 | surveillance | 99.7 | 2249 | hCOV-19/USA/PA-Jefferson_20210803_COVIDSeq14_14/2021 |
| VSP8389 | AY.13 | 7/27/21 | surveillance | 96.2 | 993 | hCOV-19/USA/PA-Jefferson_20210803_COVIDSeq14_15/2021 |
| VSP8390 | AY.25 | 7/27/21 | surveillance | 99.7 | 3273 | hCOV-19/USA/PA-Jefferson_20210803_COVIDSeq14_16/2021 |
| VSP8391 | AY.15 | 7/27/21 | accine breakthroug | 99.7 | 2896 | hCOV-19/USA/PA-Jefferson_20210803_COVIDSeq14_17/2021 |
| VSP8392 | AY.25 | 7/27/21 | surveillance | 99.7 | 2818 | hCOV-19/USA/PA-Jefferson_20210803_COVIDSeq14_18/2021 |
| VSP8393 | AY.25 | 7/28/21 | accine breakthroug | 99.7 | 2168 | hCOV-19/USA/PA-Jefferson_20210803_COVIDSeq14_19/2021 |
| VSP8394 | AY.13 | 7/28/21 | surveillance | 99.7 | 1215 | hCOV-19/USA/PA-Jefferson_20210803_COVIDSeq14_20/2021 |
| VSP8395 | AY.14 | 7/28/21 | surveillance | 99.7 | 2199 | hCOV-19/USA/PA-Jefferson_20210803_COVIDSeq14_21/2021 |
| VSP8396 | B.1.617.2 | 7/28/21 | surveillance | 99.7 | 1494 | hCOV-19/USA/PA-Jefferson_20210803_COVIDSeq14_22/2021 |
| VSP8397 | B.1.617.2 | 7/27/21 | surveillance | 99.6 | 2225 | hCOV-19/USA/PA-Jefferson_20210803_COVIDSeq14_23/2021 |
| VSP8398 | AY.3 | 7/29/21 | surveillance | 99.6 | 2206 | hCOV-19/USA/PA-Jefferson_20210803_COVIDSeq14_24/2021 |
| VSP8399 | AY.25 | 7/29/21 | accine breakthroug | 99.7 | 2262 | hCOV-19/USA/PA-Jefferson_20210803_COVIDSeq14_25/2021 |
| VSP8400 | B.1.617.2 | 7/28/21 | surveillance | 99.7 | 1432 | hCOV-19/USA/PA-Jefferson_20210803_COVIDSeq14_26/2021 |
| VSP8401 | B.1.617.2 | 7/28/21 | surveillance | 99.7 | 2438 | hCOV-19/USA/PA-Jefferson_20210803_COVIDSeq14_27/2021 |
| VSP8402 | B.1.617.2 | 7/28/21 | accine breakthroug | 99.7 | 1804 | hCOV-19/USA/PA-Jefferson_20210803_COVIDSeq14_28/2021 |
| VSP8403 | B.1.617.2 | 7/28/21 | accine breakthroug | 99.7 | 1659 | hCOV-19/USA/PA-Jefferson_20210803_COVIDSeq14_29/2021 |
| VSP8404 | B.1.617.2 | 7/29/21 | accine breakthroug | 99.7 | 2332 | hCOV-19/USA/PA-Jefferson_20210803_COVIDSeq14_30/2021 |
| VSP8405 | AY.25 | 7/29/21 | surveillance | 99.7 | 1202 | hCOV-19/USA/PA-Jefferson_20210803_COVIDSeq14_31/2021 |
| VSP8406 | B.1.617.2 | 7/29/21 | surveillance | 99.7 | 2127 | hCOV-19/USA/PA-Jefferson_20210803_COVIDSeq14_32/2021 |

| | | | | | | |
|---|---|---|---|---|---|---|
| VSP8407 | AY.25 | 7/29/21 | surveillance | 99.7 | 2337 | hCOV-19/USA/PA-Jefferson_20210803_COVIDSeq14_33/2021 |
| VSP8408 | AY.20 | 7/29/21 | surveillance | 98.2 | 1276 | hCOV-19/USA/PA-Jefferson_20210803_COVIDSeq14_34/2021 |
| VSP8409 | B.1.617.2 | 7/29/21 | surveillance | 99.7 | 2303 | hCOV-19/USA/PA-Jefferson_20210803_COVIDSeq14_35/2021 |
| VSP8410 | B.1.617.2 | 7/29/21 | surveillance | 99.7 | 1761 | hCOV-19/USA/PA-Jefferson_20210803_COVIDSeq14_36/2021 |
| VSP8411 | B.1.617.2 | 7/30/21 | surveillance | 99.7 | 2100 | hCOV-19/USA/PA-Jefferson_20210803_COVIDSeq14_37/2021 |
| VSP8412 | AY.25 | 7/29/21 | surveillance | 96.9 | 1267 | hCOV-19/USA/PA-Jefferson_20210803_COVIDSeq14_38/2021 |
| VSP8413 | B.1.1.7 | 7/29/21 | surveillance | 96.3 | 868 | hCOV-19/USA/PA-Jefferson_20210803_COVIDSeq14_39/2021 |
| VSP8414 | B.1.617.2 | 7/29/21 | surveillance | 99.7 | 2412 | hCOV-19/USA/PA-Jefferson_20210803_COVIDSeq14_40/2021 |
| VSP8415 | B.1.617.2 | 7/30/21 | accine breakthroug | 99.7 | 2451 | hCOV-19/USA/PA-Jefferson_20210803_COVIDSeq14_41/2021 |
| VSP8416 | AY.3 | 7/30/21 | surveillance | 99.3 | 2089 | hCOV-19/USA/PA-Jefferson_20210803_COVIDSeq14_42/2021 |
| VSP8417 | AY.10 | 7/30/21 | surveillance | 98.5 | 1286 | hCOV-19/USA/PA-Jefferson_20210810_COVIDSeq15_01/2021 |
| VSP8418 | AY.3 | 7/30/21 | surveillance | 98.9 | 1749 | hCOV-19/USA/PA-Jefferson_20210810_COVIDSeq15_02/2021 |
| VSP8420 | B.1.617.2 | 7/31/21 | surveillance | 99.7 | 2341 | hCOV-19/USA/PA-Jefferson_20210810_COVIDSeq15_04/2021 |
| VSP8421 | B.1.617.2 | 7/31/21 | surveillance | 99.7 | 2182 | hCOV-19/USA/PA-Jefferson_20210810_COVIDSeq15_05/2021 |
| VSP8422 | B.1.617.2 | 8/1/21 | accine breakthroug | 99.7 | 1911 | hCOV-19/USA/PA-Jefferson_20210810_COVIDSeq15_06/2021 |
| VSP8423 | B.1.617.2 | 8/1/21 | surveillance | 99.1 | 1776 | hCOV-19/USA/PA-Jefferson_20210810_COVIDSeq15_07/2021 |
| VSP8424 | AY.3 | 7/30/21 | surveillance | 99.1 | 1864 | hCOV-19/USA/PA-Jefferson_20210810_COVIDSeq15_08/2021 |
| VSP8425 | AY.3 | 7/31/21 | surveillance | 99.2 | 2131 | hCOV-19/USA/PA-Jefferson_20210810_COVIDSeq15_09/2021 |
| VSP8426 | B.1.617.2 | 8/2/21 | surveillance | 99.7 | 1961 | hCOV-19/USA/PA-Jefferson_20210810_COVIDSeq15_10/2021 |
| VSP8427 | B.1.617.2 | 8/2/21 | accine breakthroug | 99.7 | 2567 | hCOV-19/USA/PA-Jefferson_20210810_COVIDSeq15_11/2021 |
| VSP8428 | B.1.617.2 | 8/2/21 | surveillance | 99.7 | 2871 | hCOV-19/USA/PA-Jefferson_20210810_COVIDSeq15_12/2021 |
| VSP8429 | B.1.617.2 | 8/2/21 | surveillance | 99.7 | 2861 | hCOV-19/USA/PA-Jefferson_20210810_COVIDSeq15_13/2021 |
| VSP8430 | B.1.617.2 | 8/2/21 | accine breakthroug | 99.7 | 1968 | hCOV-19/USA/PA-Jefferson_20210810_COVIDSeq15_14/2021 |
| VSP8431 | B.1.617.2 | 8/2/21 | surveillance | 99.7 | 1685 | hCOV-19/USA/PA-Jefferson_20210810_COVIDSeq15_15/2021 |
| VSP8433 | AY.25 | 8/2/21 | surveillance | 99.7 | 2123 | hCOV-19/USA/PA-Jefferson_20210810_COVIDSeq15_17/2021 |
| VSP8434 | B.1.617.2 | 8/2/21 | surveillance | 99.7 | 2030 | hCOV-19/USA/PA-Jefferson_20210810_COVIDSeq15_18/2021 |
| VSP8435 | B.1.617.2 | 7/21/21 | surveillance | 99.7 | 2698 | hCOV-19/USA/PA-Jefferson_20210810_COVIDSeq15_19/2021 |
| VSP8436 | B.1.617.2 | 8/2/21 | accine breakthroug | 99.7 | 1299 | hCOV-19/USA/PA-Jefferson_20210810_COVIDSeq15_20/2021 |
| VSP8439 | B.1.617.2 | 8/3/21 | surveillance | 99.7 | 2359 | hCOV-19/USA/PA-Jefferson_20210810_COVIDSeq15_23/2021 |
| VSP8441 | B.1.617.2 | 8/4/21 | accine breakthroug | 99.6 | 1378 | hCOV-19/USA/PA-Jefferson_20210810_COVIDSeq15_25/2021 |
| VSP8442 | B.1.617.2 | 8/4/21 | accine breakthroug | 98.2 | 1736 | hCOV-19/USA/PA-Jefferson_20210810_COVIDSeq15_26/2021 |
| VSP8444 | AY.25 | 8/5/21 | accine breakthroug | 99.7 | 1483 | hCOV-19/USA/PA-Jefferson_20210810_COVIDSeq15_28/2021 |
| VSP8445 | AY.3 | 8/5/21 | accine breakthroug | 99.1 | 1785 | hCOV-19/USA/PA-Jefferson_20210810_COVIDSeq15_29/2021 |
| VSP8446 | AY.25 | 8/7/21 | accine breakthroug | 99.1 | 1547 | hCOV-19/USA/PA-Jefferson_20210810_COVIDSeq15_30/2021 |
| VSP8447 | B.1.617.2 | 8/6/21 | accine breakthroug | 99.7 | 2377 | hCOV-19/USA/PA-Jefferson_20210810_COVIDSeq15_31/2021 |
| VSP8448 | AY.4 | 8/9/21 | accine breakthroug | 99.7 | 2087 | hCOV-19/USA/PA-Jefferson_20210810_COVIDSeq15_32/2021 |
| VSP8451 | AY.25 | 8/8/21 | surveillance | 96.7 | 1401 | hCOV-19/USA/PA-Jefferson_20210810_COVIDSeq15_35/2021 |
| VSP8452 | AY.3 | 8/8/21 | surveillance | 99.2 | 2359 | hCOV-19/USA/PA-Jefferson_20210810_COVIDSeq15_36/2021 |
| VSP8455 | AY.20 | 8/8/21 | surveillance | 99.7 | 1486 | hCOV-19/USA/PA-Jefferson_20210810_COVIDSeq15_39/2021 |
| VSP8456 | B.1.617.2 | 8/8/21 | surveillance | 98.9 | 1032 | hCOV-19/USA/PA-Jefferson_20210810_COVIDSeq15_40/2021 |
| VSP8457 | B.1.617.2 | 8/7/21 | surveillance | 99.7 | 2425 | hCOV-19/USA/PA-Jefferson_20210810_COVIDSeq15_41/2021 |
| VSP8458 | B.1.617.2 | 8/7/21 | surveillance | 99.7 | 1873 | hCOV-19/USA/PA-Jefferson_20210810_COVIDSeq15_42/2021 |
| VSP8460 | B.1.617.2 | 8/9/21 | accine breakthroug | 99.7 | 961 | hCOV-19/USA/PA-Jefferson_20210810_COVIDSeq15_44/2021 |
| VSP8461 | B.1.617.2 | 8/7/21 | surveillance | 99.7 | 2392 | hCOV-19/USA/PA-Jefferson_20210810_COVIDSeq15_45/2021 |
| VSP8462 | B.1.617.2 | 8/7/21 | surveillance | 99.7 | 2545 | hCOV-19/USA/PA-Jefferson_20210810_COVIDSeq15_46/2021 |
| VSP8463 | AY.25 | 8/6/21 | surveillance | 99 | 1498 | hCOV-19/USA/PA-Jefferson_20210816_COVIDSeq16_01/2021 |
| VSP8464 | AY.2 | 8/6/21 | surveillance | 99.7 | 3084 | hCOV-19/USA/PA-Jefferson_20210816_COVIDSeq16_02/2021 |

| | | | | | | |
|---|---|---|---|---|---|---|
| VSP8465 | AY.12 | 8/7/21 | surveillance | 97.9 | 1325 | hCOV-19/USA/PA-Jefferson_20210816_COVIDSeq16_03/2021 |
| VSP8466 | B.1.617.2 | 8/10/21 | accine breakthroug | 99.7 | 2462 | hCOV-19/USA/PA-Jefferson_20210816_COVIDSeq16_04/2021 |
| VSP8467 | AY.3 | 8/9/21 | accine breakthroug | 99.1 | 3176 | hCOV-19/USA/PA-Jefferson_20210816_COVIDSeq16_05/2021 |
| VSP8468 | B.1.617.2 | 8/10/21 | accine breakthroug | 99.7 | 1756 | hCOV-19/USA/PA-Jefferson_20210816_COVIDSeq16_06/2021 |
| VSP8469 | B.1.617.2 | 8/10/21 | accine breakthroug | 99.7 | 1991 | hCOV-19/USA/PA-Jefferson_20210816_COVIDSeq16_07/2021 |
| VSP8471 | B.1.617.2 | 8/10/21 | surveillance | 99.7 | 2839 | hCOV-19/USA/PA-Jefferson_20210816_COVIDSeq16_09/2021 |
| VSP8472 | B.1.617.2 | 8/10/21 | surveillance | 99.7 | 2434 | hCOV-19/USA/PA-Jefferson_20210816_COVIDSeq16_10/2021 |
| VSP8473 | AY.25 | 8/10/21 | surveillance | 99.7 | 2794 | hCOV-19/USA/PA-Jefferson_20210816_COVIDSeq16_11/2021 |
| VSP8474 | B.1.617.2 | 8/10/21 | surveillance | 99.7 | 2627 | hCOV-19/USA/PA-Jefferson_20210816_COVIDSeq16_12/2021 |
| VSP8475 | B.1.617.2 | 8/9/21 | surveillance | 99.7 | 2690 | hCOV-19/USA/PA-Jefferson_20210816_COVIDSeq16_13/2021 |
| VSP8476 | B.1.617.2 | 8/11/21 | accine breakthroug | 99.7 | 2504 | hCOV-19/USA/PA-Jefferson_20210816_COVIDSeq16_14/2021 |
| VSP8477 | AY.3 | 8/11/21 | accine breakthroug | 99.6 | 2880 | hCOV-19/USA/PA-Jefferson_20210816_COVIDSeq16_15/2021 |
| VSP8478 | B.1.617.2 | 8/11/21 | surveillance | 99.7 | 2882 | hCOV-19/USA/PA-Jefferson_20210816_COVIDSeq16_16/2021 |
| VSP8479 | AY.20 | 8/10/21 | surveillance | 99.7 | 2787 | hCOV-19/USA/PA-Jefferson_20210816_COVIDSeq16_17/2021 |
| VSP8480 | AY.14 | 8/11/21 | surveillance | 99.8 | 3235 | hCOV-19/USA/PA-Jefferson_20210816_COVIDSeq16_18/2021 |
| VSP8481 | AY.3 | 8/11/21 | surveillance | 99.1 | 2230 | hCOV-19/USA/PA-Jefferson_20210816_COVIDSeq16_19/2021 |
| VSP8482 | B.1.617.2 | 8/11/21 | surveillance | 99.7 | 3115 | hCOV-19/USA/PA-Jefferson_20210816_COVIDSeq16_20/2021 |
| VSP8483 | B.1.617.2 | 8/12/21 | accine breakthroug | 99.7 | 2461 | hCOV-19/USA/PA-Jefferson_20210816_COVIDSeq16_21/2021 |
| VSP8484 | B.1.617.2 | 8/11/21 | accine breakthroug | 99.7 | 2427 | hCOV-19/USA/PA-Jefferson_20210816_COVIDSeq16_22/2021 |
| VSP8485 | B.1.617.2 | 8/13/21 | accine breakthroug | 96.8 | 1143 | hCOV-19/USA/PA-Jefferson_20210816_COVIDSeq16_23/2021 |
| VSP8488 | B.1.617.2 | 8/11/21 | accine breakthroug | 99.7 | 1641 | hCOV-19/USA/PA-Jefferson_20210816_COVIDSeq16_26/2021 |
| VSP8489 | B.1.617.2 | 8/13/21 | accine breakthroug | 99.7 | 3091 | hCOV-19/USA/PA-Jefferson_20210816_COVIDSeq16_27/2021 |
| VSP8491 | B.1.617.2 | 8/13/21 | accine breakthroug | 99.7 | 2429 | hCOV-19/USA/PA-Jefferson_20210816_COVIDSeq16_29/2021 |
| VSP8492 | B.1.617.2 | 8/13/21 | accine breakthroug | 99.7 | 3048 | hCOV-19/USA/PA-Jefferson_20210816_COVIDSeq16_30/2021 |
| VSP8493 | P.1 | 7/25/21 | accine breakthroug | 99.7 | 2349 | hCOV-19/USA/PA-Jefferson_20210816_COVIDSeq16_31/2021 |
| VSP8494 | B.1.617.2 | 8/15/21 | surveillance | 99.7 | 2347 | hCOV-19/USA/PA-Jefferson_20210816_COVIDSeq16_32/2021 |
| VSP8495 | AY.12 | 8/15/21 | surveillance | 99.7 | 2169 | hCOV-19/USA/PA-Jefferson_20210816_COVIDSeq16_33/2021 |
| VSP8496 | AY.25 | 8/15/21 | surveillance | 97.1 | 1214 | hCOV-19/USA/PA-Jefferson_20210816_COVIDSeq16_34/2021 |
| VSP8497 | AY.3 | 8/15/21 | accine breakthroug | 99.6 | 2716 | hCOV-19/USA/PA-Jefferson_20210816_COVIDSeq16_35/2021 |
| VSP8498 | B.1.1.7 | 8/14/21 | surveillance | 99.1 | 2607 | hCOV-19/USA/PA-Jefferson_20210816_COVIDSeq16_36/2021 |
| VSP8499 | B.1.617.2 | 8/14/21 | surveillance | 99.7 | 2912 | hCOV-19/USA/PA-Jefferson_20210816_COVIDSeq16_37/2021 |
| VSP8500 | AY.25 | 8/14/21 | surveillance | 99.7 | 3019 | hCOV-19/USA/PA-Jefferson_20210816_COVIDSeq16_38/2021 |
| VSP8501 | B.1.617.2 | 8/14/21 | surveillance | 99 | 1420 | hCOV-19/USA/PA-Jefferson_20210816_COVIDSeq16_39/2021 |
| VSP8502 | AY.25 | 8/14/21 | surveillance | 99.7 | 2490 | hCOV-19/USA/PA-Jefferson_20210816_COVIDSeq16_40/2021 |
| VSP8503 | AY.24 | 8/13/21 | surveillance | 99.6 | 2500 | hCOV-19/USA/PA-Jefferson_20210816_COVIDSeq16_41/2021 |
| VSP8504 | B.1.617.2 | 8/13/21 | surveillance | 99.7 | 2382 | hCOV-19/USA/PA-Jefferson_20210816_COVIDSeq16_42/2021 |
| VSP8505 | AY.25 | 8/13/21 | surveillance | 99.8 | 2844 | hCOV-19/USA/PA-Jefferson_20210816_COVIDSeq16_43/2021 |
| VSP8506 | AY.25 | 8/14/21 | surveillance | 99.5 | 2893 | hCOV-19/USA/PA-Jefferson_20210816_COVIDSeq16_44/2021 |
| VSP8507 | AY.3 | 8/14/21 | surveillance | 99.1 | 2882 | hCOV-19/USA/PA-Jefferson_20210816_COVIDSeq16_45/2021 |
| VSP8508 | B.1.617.2 | 8/14/21 | surveillance | 96.4 | 869 | hCOV-19/USA/PA-Jefferson_20210816_COVIDSeq16_46/2021 |
