# Supplementary material for: SARS-CoV-2 Variants Associated with Vaccine Breakthrough in the Delaware Valley through Summer 2021
Source: mBio. 2022 Feb 8;13(1):e03788-21. doi: 10.1128/mbio.03788-21 (PMC8942461; doi:10.1128/mbio.03788-21)
Supplement: TABLE S3 [file mbio.03788-21-st003.pdf]

Table S3 Estimated fold enrichment in odds of appearing in the spike gene target failu

	Mean	Lower 95% CrI	Upper 95% CrI
B.1.1.7	120	38	470
Other Alpha	0.93	0.046	14
Other Beta	0.91	0.05	12
AY.12	1	0.049	18
AY.14	0.99	0.048	20
AY.20	1	0.051	19
AY.24	0.99	0.052	19
AY.25	1	0.052	19
AY.3	0.98	0.048	20
AY.4	0.99	0.051	19
B.1.617.2	0.97	0.048	17
Other Delta	0.98	0.052	17
B.1.525	33	1.7	380
Other Gamma	0.9	0.045	12
P.1	0.78	0.041	7.3
P.1.2	0.95	0.05	15
B.1.526	0.39	0.018	2.4
Other Kappa	0.97	0.05	17
B.1	0.7	0.035	5.9
B.1.1	0.73	0.044	5.9
B.1.1.434	2.8	0.35	34
B.1.1.519	0.69	0.039	5.2
B.1.2	0.92	0.15	4.3
B.1.234	0.73	0.042	5.9
B.1.243	0.46	0.023	2.9
B.1.311	0.69	0.039	5.1
B.1.575	0.62	0.034	4.3
B.1.596	0.72	0.041	5.9
B.1.621	0.92	0.051	13
B.1.637	0.65	0.035	4.7
R.1	0.65	0.036	4.8
Other	2.3	0.49	14

re set for each SARS-CoV-2 lineage.
