# Supplementary material for: SARS-CoV-2 Variants Associated with Vaccine Breakthrough in the Delaware Valley through Summer 2021
Source: mBio. 2022 Feb 8;13(1):e03788-21. doi: 10.1128/mbio.03788-21 (PMC8942461; doi:10.1128/mbio.03788-21)
Supplement: TABLE S4 [file mbio.03788-21-st004.pdf]

Table S4 Estimated fold enrichment in odds of appearing in vaccine breakthrough sam

	Mean	Lower 95% CrI	Upper 95% CrI
Delta	1.6	0.49	7
Gamma	1.6	0.4	8.1
Non-VBM/VoC	0.69	0.22	1.9
B.1.1.7	1.4	0.57	3.9
Other Alpha	0.5	0.025	5.2
Other Beta	0.46	0.023	4
AY.12	1.2	0.17	7.4
AY.14	1.4	0.19	8.9
AY.20	0.63	0.028	5.7
AY.24	2	0.36	12
AY.25	1.1	0.25	4.9
AY.3	2.3	0.57	10
AY.4	2.4	0.43	15
B.1.617.2	3	0.89	11
Other Delta	0.65	0.095	3.5
B.1.525	0.47	0.025	2.8
Other Gamma	0.42	0.02	3.5
P.1	1.5	0.43	5.2
P.1.2	2.8	0.49	15
B.1.526	0.65	0.22	1.8
Other Kappa	4.3	0.23	230
B.1	1.1	0.14	8.8
B.1.1	0.43	0.02	3.8
B.1.1.434	0.48	0.022	4.5
B.1.1.519	0.59	0.09	2.9
B.1.2	0.4	0.078	1.6
B.1.234	0.88	0.12	5.3
B.1.243	0.75	0.15	3.3
B.1.311	0.37	0.018	3
B.1.575	0.22	0.0095	1.6
B.1.596	0.38	0.019	3
B.1.621	1.9	0.44	8.2
B.1.637	0.58	0.12	2.3
R.1	0.74	0.15	3.1
Other	0.96	0.25	3.6

ıples for each SARS-CoV-2 lineage.
