# Supplementary material for: SARS-CoV-2 Variants Associated with Vaccine Breakthrough in the Delaware Valley through Summer 2021
Source: mBio. 2022 Feb 8;13(1):e03788-21. doi: 10.1128/mbio.03788-21 (PMC8942461; doi:10.1128/mbio.03788-21)
Supplement: TABLE S6 [file mbio.03788-21-st006.pdf]

Table S6 Estimated fold enrichment in odds of appearing in the spike gene target failure set for each substitution or deletion studied.

Genomic Position	Gene	Protein	Mutation	Mean	Lower 95% CrI	Upper 95% CrI
820	ORF1ab	Nsp2	silent_820	3.4169831	2.223642775	4.999581057
913	ORF1ab	Nsp2	silent_913	110.133251	46.12986091	248.9815925
1059	ORF1ab	Nsp2	T265I	0.027341173	0.008692578	0.056896565
2110	ORF1ab	Nsp2	silent_2110	5.143735755	3.629610544	7.045590403
3037	ORF1ab	Nsp3	silent_3037	0.57863018	0.0066219	2.593720874
3267	ORF1ab	Nsp3	T1001I	117.0038585	47.64157222	269.5812541
4181	ORF1ab	Nsp3	A1306S	2.453096065	0.04548474	13.47005686
5388	ORF1ab	Nsp3	A1708D	81.43221553	37.52531916	169.174047
5986	ORF1ab	Nsp3	silent_5986	92.93347711	40.91961916	199.4979278
6402	ORF1ab	Nsp3	P2046L	2.567295381	0.046084352	13.54010167
6954	ORF1ab	Nsp3	I2230T	81.5124263	37.7273632	165.9670938
7042	ORF1ab	Nsp3	M2259I	5.64342404	3.614214376	8.445288805
7124	ORF1ab	Nsp3	P2287S	1.473865023	0.042935253	7.100740171
8986	ORF1ab	Nsp4	silent_8986	2.521796145	0.047590859	12.83777315
9053	ORF1ab	Nsp4	V2930L	1.748502105	0.044795588	8.550890752
9867	ORF1ab	Nsp4	L3201P	0.016084657	0.000395779	0.060730817
9891	ORF1ab	Nsp4	A3209V	0.106117604	0.002467382	0.399837717
10029	ORF1ab	Nsp4	T3255I	0.054541658	0.001312743	0.201759826
10319	ORF1ab	3CL-PRO	L3352F	0.07796607	0.015803928	0.190407057
11201	ORF1ab	Nsp6	T3646A	2.142131386	0.049184246	11.02203797
11288	ORF1ab	Nsp6	del_9_11288	60.9903687	21.03818496	159.0248525
11332	ORF1ab	Nsp6	silent_11332	2.346052737	0.049162021	12.30247315
14120	ORF1ab	Pol	P218L	5.600469973	3.931735133	7.747528481
14408	ORF1ab	Pol	P314L	1.134810112	0.28954804	3.362942035
14676	ORF1ab	Pol	silent_14676	109.7614966	45.63516585	246.1947389
15096	ORF1ab	Pol	silent_15096	3.313638366	2.217624188	4.739897476
15279	ORF1ab	Pol	silent_15279	115.9570956	47.17087253	265.8400144
15451	ORF1ab	Pol	G662S	1.133060367	0.038575826	4.637501271
15720	ORF1ab	Pol	silent_15720	1.172269071	0.633649636	1.961727723
16176	ORF1ab	Pol	silent_16176	110.7732772	46.97063918	249.2621181
16466	ORF1ab	Hel	P1000L	1.40915858	0.044026383	6.562087234
16500	ORF1ab	Hel	Q1011H	0.042241921	0.004966006	0.119555099
17615	ORF1ab	Hel	K1383R	2.668176659	1.684643474	3.981633884
18424	ORF1ab	ExoN	N1653D	0.088805381	0.017502498	0.220156691
19220	ORF1ab	ExoN	A1918V	2.600179593	0.05024288	14.60980137
20262	ORF1ab	Nsp15	silent_20262	0.040271351	0.004729179	0.114342534
21304	ORF1ab	Nsp16	R2613C	0.095418551	0.01902739	0.236078236
21575	S	Spike	L5F	0.261751089	0.138095842	0.429562623
21618	S	Spike	T19R	1.425454145	0.044362857	6.7160796
21765	S	Spike	del_6_21765	204.9412887	65.24688523	580.7747042
21846	S	Spike	T95I	0.042753581	0.005183494	0.118482277
21991	S	Spike	del_3_21991	96.47930698	38.60205317	229.9342997
22029	S	Spike	del_6_22029	1.440010864	0.042670578	6.753640322
22320	S	Spike	D253G	0.021573446	0.000526542	0.081117114
22917	S	Spike	L452R	0.093474175	0.00243089	0.353395493
22995	S	Spike	T478K	0.187514137	0.004332081	0.727596349
23012	S	Spike	E484K	0.073844955	0.02014935	0.161731114
23063	S	Spike	N501Y	84.08236256	36.80762043	181.2479778
23271	S	Spike	A570D	92.63852536	41.18301281	200.9122891
23403	S	Spike	D614G	10.21568067	0.105689761	22.12066297
23604	S	Spike	P681H	36.89190094	17.32144956	76.33502148
23604	S	Spike	P681R	1.076884534	0.034973012	4.300776899
23664	S	Spike	A701V	0.053880834	0.006563377	0.150664787
23709	S	Spike	T716I	68.82004724	30.51037793	146.3798711
24410	S	Spike	D950N	1.114349025	0.031995039	4.578417908
24506	S	Spike	S982A	94.4398387	41.13394376	204.9617562
24914	S	Spike	D1118H	92.83796364	40.71247625	201.2832695
26528	M	Membrane	silent_26528	2.56971927	1.220105429	4.65120653
26604	M	Membrane	F28L	0.161190261	0.003808663	0.614969257
26767	M	Membrane	I82T	0.43257257	0.085540329	1.043335024
28280	N	Nucleocapsid	D3L	110.4411058	45.52449224	248.9833403
28461	N	Nucleocapsid	D63G	1.42730005	0.044005276	6.560967987
28472	N	Nucleocapsid	P67S	0.090380236	0.018405603	0.221696127
28854	N	Nucleocapsid	S194L	0.057227229	0.001379343	0.216422501
28869	N	Nucleocapsid	P199L	0.048984571	0.013281455	0.108990337
28881	N	Nucleocapsid	R203K	32.80411121	16.56337894	62.80766322
28881	N	Nucleocapsid	R203M	1.369070102	0.039930912	6.38380742
28883	N	Nucleocapsid	G204R	32.7432095	16.45693171	62.39642202
28887	N	Nucleocapsid	T205I	0.156118675	0.040621359	0.347168753
28916	N	Nucleocapsid	G215C	2.526033886	0.047128757	13.86076845
28975	N	Nucleocapsid	M234I	0.019806226	0.000501329	0.074522047
28977	N	Nucleocapsid	S235F	71.99219236	34.41151267	143.6599875
29272	N	Nucleocapsid	silent_29272	3.276020462	2.143577196	4.777103474
29402	N	Nucleocapsid	D377Y	0.055489245	0.00147683	0.207724496
