# Supplementary material for: SARS-CoV-2 Variants Associated with Vaccine Breakthrough in the Delaware Valley through Summer 2021
Source: mBio. 2022 Feb 8;13(1):e03788-21. doi: 10.1128/mbio.03788-21 (PMC8942461; doi:10.1128/mbio.03788-21)
Supplement: TABLE S7 [file mbio.03788-21-st007.pdf]

Table S7 Estimated fold enrichment in odds of appearing in the vaccine breakthrough samples for each substitution or deletion studied.

Genomic Position	Gene	Protein	Mutation	Mean	Lower 95% CrI	Upper 95% CrI
820	ORF1ab	Nsp2	silent_820	0.810724	0.425987922	1.311655448
913	ORF1ab	Nsp2	silent_913	1.5855022	1.018622732	2.398031376
1059	ORF1ab	Nsp2	T265I	0.4762545	0.265890738	0.765807108
2110	ORF1ab	Nsp2	silent_2110	1.2810869	0.777396644	2.001655539
3037	ORF1ab	Nsp3	silent_3037	6.1248042	0.090070614	20.91676927
3267	ORF1ab	Nsp3	T1001I	1.5191445	0.987066153	2.279995511
4181	ORF1ab	Nsp3	A1306S	1.0448252	0.565388723	1.8055564
5388	ORF1ab	Nsp3	A1708D	1.4595427	0.961071014	2.166247955
5986	ORF1ab	Nsp3	silent_5986	1.5370474	1.001099921	2.283418664
6402	ORF1ab	Nsp3	P2046L	1.0326591	0.550010869	1.804972135
6954	ORF1ab	Nsp3	I2230T	1.5363607	0.995722314	2.306073715
7042	ORF1ab	Nsp3	M2259I	1.3250678	0.690644694	2.30880471
7124	ORF1ab	Nsp3	P2287S	0.965431	0.517040839	1.652767609
8986	ORF1ab	Nsp4	silent_8986	1.0406789	0.55399617	1.814140772
9053	ORF1ab	Nsp4	V2930L	1.0481489	0.561659857	1.839947282
9867	ORF1ab	Nsp4	L3201P	0.5355704	0.271296197	0.89092041
9891	ORF1ab	Nsp4	A3209V	1.1091302	0.620655862	1.819869618
10029	ORF1ab	Nsp4	T3255I	0.986886	0.58630171	1.546189381
10319	ORF1ab	3CL-PRO	L3352F	0.8251902	0.300257812	1.60285745
11201	ORF1ab	Nsp6	T3646A	1.1376408	0.612731638	2.006846346
11288	ORF1ab	Nsp6	del_9_11288	1.0468665	0.62645507	1.684079573
11332	ORF1ab	Nsp6	silent_11332	1.1348329	0.606839189	2.022338997
14120	ORF1ab	Pol	P218L	1.3328722	0.796418115	2.103012451
14408	ORF1ab	Pol	P314L	11.001201	0.484817413	45.88241582
14676	ORF1ab	Pol	silent_14676	1.5661209	1.010222617	2.35567343
15096	ORF1ab	Pol	silent_15096	0.8886217	0.510374108	1.367321013
15279	ORF1ab	Pol	silent_15279	1.5886246	1.023686527	2.401323285
15451	ORF1ab	Pol	G662S	1.8370109	0.722620381	4.252488304
15720	ORF1ab	Pol	silent_15720	1.5517575	0.849932487	2.59464604
16176	ORF1ab	Pol	silent_16176	1.5279447	0.994220928	2.284724616
16466	ORF1ab	Hel	P1000L	1.9453037	0.744274959	4.574104139
16500	ORF1ab	Hel	Q1011H	0.5603368	0.270820586	0.963896113
17615	ORF1ab	Hel	K1383R	1.4479914	0.832989251	2.355733499
18424	ORF1ab	ExoN	N1653D	0.6179103	0.164504149	1.316682584
19220	ORF1ab	ExoN	A1918V	1.0356321	0.548354144	1.819620733
20262	ORF1ab	Nsp15	silent_20262	0.5942825	0.311339814	0.979988783
21304	ORF1ab	Nsp16	R2613C	0.479472	0.096327986	1.127566379
21575	S	Spike	L5F	0.6635113	0.361682064	1.057407589
21618	S	Spike	T19R	1.4662188	0.49911954	3.641652632
21765	S	Spike	del_6_21765	1.5175732	0.984938631	2.27113005
21846	S	Spike	T95I	0.8368207	0.519596275	1.226270998
21991	S	Spike	del_3_21991	1.384252	0.911332839	2.075324262
22029	S	Spike	del_6_22029	1.0379389	0.415294229	2.222463047
22320	S	Spike	D253G	0.5021113	0.231594124	0.88737621
22917	S	Spike	L452R	1.2812442	0.66477337	2.269998708
22995	S	Spike	T478K	1.5155461	0.666514971	3.084620934
23012	S	Spike	E484K	0.9329095	0.566306197	1.393814533
23063	S	Spike	N501Y	2.0367664	1.250733322	3.176850638
23271	S	Spike	A570D	1.5162158	0.986862281	2.266557851
23403	S	Spike	D614G	5.6304256	0.111328207	20.08100693
23604	S	Spike	P681H	1.5102386	0.98334628	2.260187547
23604	S	Spike	P681R	2.2460476	0.734998695	5.611445632
23664	S	Spike	A701V	0.5914999	0.256206344	1.057296411
23709	S	Spike	T716I	1.4381836	0.945564293	2.134299568
24410	S	Spike	D950N	2.2638617	0.93667791	4.593211471
24506	S	Spike	S982A	1.5099331	0.975373497	2.272288423
24914	S	Spike	D1118H	1.557453	1.011756133	2.330004327
26528	M	Membrane	silent_26528	1.2827955	0.52386102	2.578098721
26604	M	Membrane	F28L	2.2795709	0.445633619	7.213825822
26767	M	Membrane	I82T	1.5711841	0.579970146	3.631962356
28280	N	Nucleocapsid	D3L	1.5841987	1.027720398	2.362042429
28461	N	Nucleocapsid	D63G	2.1535127	0.743873945	5.40271886
28472	N	Nucleocapsid	P67S	0.4678794	0.093415366	1.094718233
28854	N	Nucleocapsid	S194L	1.8241307	0.688697864	3.959481799
28869	N	Nucleocapsid	P199L	0.5017679	0.237226567	0.884768221
28881	N	Nucleocapsid	R203K	1.6064923	1.023526664	2.446534831
28881	N	Nucleocapsid	R203M	1.8875704	0.655434986	4.729497058
28883	N	Nucleocapsid	G204R	1.608691	1.024788616	2.446987963
28887	N	Nucleocapsid	T205I	0.6628609	0.254953495	1.207525664
28916	N	Nucleocapsid	G215C	0.9917648	0.530103627	1.71744875
28975	N	Nucleocapsid	M234I	0.6807963	0.347737916	1.113327871
28977	N	Nucleocapsid	S235F	1.4973654	0.978075393	2.247171563
29272	N	Nucleocapsid	silent_29272	0.9052396	0.498282598	1.42615733
29402	N	Nucleocapsid	D377Y	0.947974	0.401135827	1.805529482
