# Supplementary material for: SARS-CoV-2 Variants Associated with Vaccine Breakthrough in the Delaware Valley through Summer 2021
Source: mBio. 2022 Feb 8;13(1):e03788-21. doi: 10.1128/mbio.03788-21 (PMC8942461; doi:10.1128/mbio.03788-21)
Supplement: TABLE S8 [file mbio.03788-21-st008.pdf]

TableS8

Key reagents

Reagent or Resource	Source	Identifier
QIAmp 96 Viral RNA Kit	Qiagen, Hilden, Germany	5262
SuperScript III RT	Thermo Fisher Scientific, Waltham, USA	56575
SS III First Strand 5x Buffer	Thermo Fisher Scientific, Waltham, USA	Y02321
Random Hexamers	Thermo Fisher Scientific, Waltham, USA	51709
Dithiothreitol	Thermo Fisher Scientific, Waltham, USA	Y00122
Molecular Grade Water	Thermo Fisher Scientific, Waltham, USA	Y01138
Deoxynucleotide Mix	New England Biolabs, Ipswich, USA	N0447S
ARTIC Primer Pool 1	Integrated DNA Technologies, Coralville, USA	100006786
ARTIC Primer Pool 2	Integrated DNA Technologies, Coralville, USA	100006787
Q5 Hot Start Polymerase	New England Biolabs, Ipswich, USA	M0493L
Q5 5x Reaction Buffer	New England Biolabs, Ipswich, USA	B90275
AMPure XP	Beckman Coulter, Brea, USA	A63882
Qubit™ 1X dsDNA Kit	Invitrogen Corp., Waltham, USA	Q33230
Quant-iT PicoGreen Kit	Invitrogen Corp., Waltham, USA	P7589
IDT for Illumina DNA/RNA	Illumina Inc., San Diego, USA	20027213
UD Indexes A-D	Illumina Inc., San Diego, USA	20027214
	Illumina Inc., San Diego, USA	20027215
	Illumina Inc., San Diego, USA	20027216
Nextera XT DNA Library Preparation Kit	Illumina Inc., San Diego, USA	FC-131-1096
NextSeq 500/550 Mid Output Kit v2.5 (150 Cycles)	Illumina Inc., San Diego, USA	20024904
